# Non-Vitamin K Antagonist Oral Anticoagulants and Risk of Myocardial Infarction in Patients with Atrial Fibrillation with or without Percutaneous Coronary Interventions: A Meta-Analysis

**DOI:** 10.3390/jpm11101013

**Published:** 2021-10-09

**Authors:** Stefan Grajek, Marta Kałużna-Oleksy, Jolanta M. Siller-Matula, Maksymilian Grajek, Michał Michalak

**Affiliations:** 1 1st Department of Cardiology, Poznan University of Medical Sciences, 61-848 Poznan, Poland; stefan.grajek@skpp.edu.pl; 2 Department of Internal Medicine II, Division of Cardiology, Medical University of Vienna, 1090 Vienna, Austria; jolanta.siller-matula@skpp.edu.pl; 3 Center for Preclinical Research and Technology (CEPT), Department of Experimental and Clinical Pharmacology, Medical University of Warsaw, 02-097 Warsaw, Poland; 4 International School of Poznan, 60-147 Poznan, Poland; maksymilian.grajek@skpp.edu.pl; 5 Department of Computer Science and Statistics, Poznan University of Medical Sciences, 60-806 Poznan, Poland; michal@ump.edu.pl

**Keywords:** coronary artery disease, probability, VKA, warfarin, NOAC, percutaneous revascularization

## Abstract

The study aimed to assess the risk of myocardial infarction (MI) and major adverse cardiac events during non-vitamin K antagonist oral anticoagulants (NOAC) compared to warfarin therapy in patients with atrial fibrillation (AF), both treated and not treated with percutaneous coronary interventions (PCI). In a systematic search, we selected eight randomized clinical trials with a total of 81,943 patients. Dabigatran, compared to warfarin, significantly increased the risk of MI (relative risk [RR] 1.38, 95% CI 1.14–1.67), while the FXa inhibitors’ effect did not differ significantly from warfarin (RR 0.96, 95% CI 0.86–1.09). The RR comparison between analyzed subgroups (dabigatran vs. FXa inhibitors) showed a significant difference (Chi^2^ = 9.51, df = 1, *p* = 0.002). In a network meta-analysis, dabigatran 110 mg b.i.d. increased the risk of MI compared to warfarin, apixaban, edoxaban, and rivaroxaban. Also, dabigatran 150 mg b.i.d. increased the risk of MI compared to warfarin, apixaban, and rivaroxaban. Moreover, we tried to estimate the treatment ranking of the best therapy for MI prevention in patients with AF treated with PCI. Rivaroxaban had a 90% probability of being ranked the best therapy for MI prevention, whereas dabigatran 110 mg had an 8.2% probability. Dabigatran 150 mg was the most effective in stroke prevention (94% probability). Each NOAC is associated with a different risk of MI. Furthermore, we should consider FXa inhibitors as the first line NOACs in AF and coronary artery disease patients. PROSPERO ID CRD42020179808.

## 1. Introduction

For years, the standard triple antithrombotic therapy (TAT) containing vitamin K antagonist (VKA), P2Y12 inhibitor (mainly clopidogrel), and aspirin was recommended in patients with atrial fibrillation (AF) treated with percutaneous coronary interventions (PCI). However, the increase in bleeding complications is a serious limitation of this treatment. Two randomized controlled trials (RCTs) showed a significant reduction of hemorrhagic complications in patients treated with dual antithrombotic therapy (DAT) containing VKA and clopidogrel or aspirin [1,2]. The possibility of shortening TAT to 6 weeks with further DAT to 6 months was also pointed out [2]. The introduction of new non-VKA oral anticoagulants (NOAC) has changed the therapeutic strategy. Several RCTs compared DAT containing NOAC and P2Y12 inhibitor with standard TAT [3,4,5,6]. All studies showed a significant reduction in hemorrhagic complications with no apparent effect on thromboembolic complications and major adverse cardiac events (MACE). The results were confirmed in subsequent meta-analyses, favoring DAT over TAT [7,8,9,10,11]. However, two most recent meta-analyses (constructed on the same RCTs) have shown that on DAT, a significant reduction in the risk of hemorrhagic complications (about 40–50%) is accompanied by an increase in the risk of myocardial infarction (MI) and stent thrombosis (ST) [12,13].

The researchers suggest an individualized administration time of TAT (NOAC, P2Y12 inhibitor, and aspirin) directly after PCI with a transition to DAT during prolonged treatment [14,15]. After stent implantation, events such as stent thrombosis (ST) or myocardial infarction (MI) depend on the implantation technique and the type of a stent, the complexity of coronary artery lesions, and appropriate pharmacological support. Dual antiplatelet therapy (DAPT) compared to single antiplatelet therapy (SAPT) gives better protection against MACE, although it increases the risk of bleeding complications. Regardless of the PCI procedure, some NOACs in patients with AF may increase the risk of MI.

Many meta-analyses have shown that dabigatran treatment leads to a significant increase in the risk of MI [16,17,18,19,20,21,22]. However, these analyses were published before the publication of the RE-DUAL PCI study [4], included patients with AF as well as with sinus rhythm, and NOACs were used for different indications (prevention of thrombo-embolic events, deep vein thrombosis, acute coronary syndrome [ACS]). Furthermore, none of these mentioned above studies concerned AF patients qualified for PCI; therefore, DAT (containing NOAC) was not compared with standard TAT. Moreover, dabigatran was compared with different comparators (placebo/aspirin, warfarin, enoxaparin). Therefore, we present a meta-analysis assessing the risk of MI and MACE during NOAC therapy compared to VKA in patients with AF, both treated and not treated with PCI.

## 2. Materials and Methods

### 2.1. The Search Strategy and Selection Criteria

We conducted a systematic search of studies in PubMed, Embase, the Cochrane Library, and Web of Science until 22 March 2020. We used the following keywords: AF, PCI, ACS, chronic coronary syndrome (CCS), coronary stenting, warfarin, dabigatran, apixaban, rivaroxaban, edoxaban, VKA, “dual antithrombotic therapy”, and “triple antithrombotic therapy”. Further analyses included studies that fulfilled the following criteria [3,4,5,6,23,24,25,26]:

(a) Only Phase III RCTs in patients with AF treated with oral anticoagulants (OAC) containing two arms, NOAC vs. warfarin, were analyzed.
(b) Only RCTs with AF patients undergoing PCI for ACS or CCS and containing two arms, DAT (NOAC + P2Y12) vs. standard TAT, were analyzed.
(c) All studies with included information on at least three following endpoints: death, stroke, and MI. We analyzed in detail the data contained in the publication and the accompanying Supplementary Materials. Two co-authors (SG and MM) performed the review and qualification for the analysis, and the third co-author (MKO) completed the final evaluation.
(d) Clinical observational studies, data registers (a real-world registry-RWD), review papers, and comments were excluded.

### 2.2. Study Outcomes

Study outcomes were thrombo-ischemic complications (efficacy endpoints): death, stroke, MI, ST, trial-defined MACE, and cardiovascular death. In addition, the RE-LY study assessed vascular death or cardiac death [23]. Ischemic and thrombo-embolic complications defined as MACE are presented in Table S1B.

### 2.3. Data Synthesis and Analysis

The methodological quality of RCTs was assessed using the Cochrane Collaboration tool for assessing the risk of bias. For each clinical trial, we assessed bias qualitatively as low, unclear, or high (Supplementary Table S2). The assessment was made independently by two authors (SG and MM). A meta-analysis comparing the results of individual NOACs vs. warfarin was performed using a random model, which considered between-study variance-tau-squared. Random effects models are more conservative, leading the estimates with wider confidence intervals. In case τ^2^ was zero, the pooled estimate of the random model was corresponded to those from the fixed-effect model. As a measure of the effect, the Mantel-Haenszel relative risk (RR) was used with a 95% confidence interval (CI). A sensitivity analysis was performed by excluding the results of 30 mg edoxaban from the ENGAGE AF-TIMI study.

Furthermore, when we evaluated three-arm studies (two different doses and a control group), the analysis was performed twice. First, performing the analysis separately to different dosages, which required doubling the events and sample size of the control group. Due to this approach, we could get an estimate of a particular dose. In the second approach, we combined the results of different dosages into one group vs. control. This approach maintained a real number of events in the control group, but as a result, the obtained estimate reflected artificial dosage (non-existing one). Both results are presented in the Supplementary Materials. However, if results remained consistent across the different models, then we considered them robust. Additionally, we analyzed a difference between the effects obtained from the drug’s classes comparison-direct thrombin inhibitors (DTI) vs. warfarin and factor Xa inhibitors (FXa inhibitors) vs. warfarin. The calculations were performed using Review Manager (RevMan 5.3 Cochrane Community, Copenhagen: the Nordic Cochrane Centre, the Cochrane Collaboration, 2014).

For comparisons between individual drugs as well as each of them with warfarin, we used a network meta-analysis (network plot) (Supplementary Figure S44). We analyzed endpoints for which at least two direct studies of the particular drug vs. warfarin were available. Therefore, the network analysis was not used for edoxaban 30 mg and cardiovascular death and ST risk assessment. Calculations were performed three times; based on data from the original RELY study and after data correction in the intention-to-treat and on-treatment analysis [23,27].

The indirect analysis of the ‘star’ type network was performed using Busher’s method [28,29,30]. The network meta-analysis was performed with the mvmet command (STATA). We then estimated the relative probability of ranking each therapy and obtained a hierarchy of competing treatments using SUCRA (surface under the cumulative ranking) with the method proposed by G. Salanti [31], which required estimation of the probability of being the best for a particular therapy. The explanations about SUCRA are included in the Supplementary Materials. The probability was estimated based on the Bayesian model. We assumed uniform distribution as a priori distribution. As a result, we received a posterior normal distribution with mean and variance, where estimators of normal distribution parameters were estimated based on frequentist inference.

The terms “on-treatment” and “intention-to-treat” used in this work are based on the common rule: when the statistical analysis is performed with the recruited sample size, the analysis refers to the group called “intention-to-treat”, however when the statistical analysis is performed based on the number of patients who finished the trial, the study refers to the group called “on-treatment”.

Calculations were made with STATA 15.1 software (StataCorp LLC, College Station, TX, USA).

## 3. Results

### 3.1. Identified Studies Characteristics

**Figure 1:**
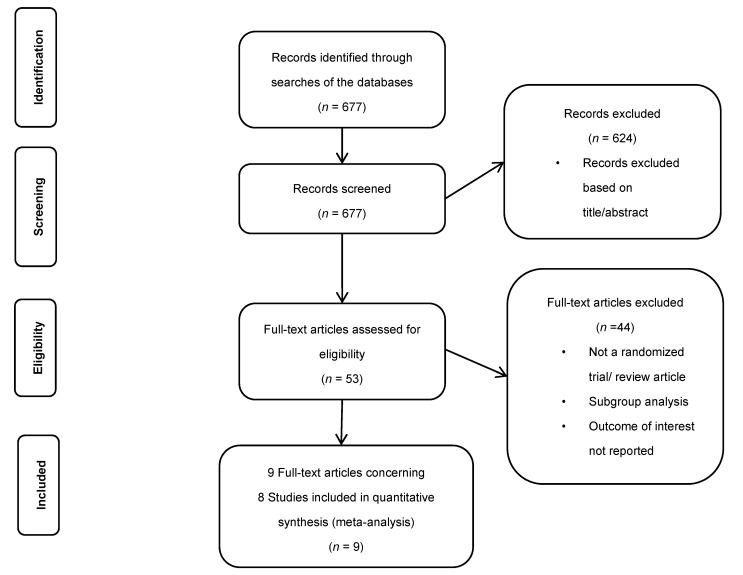
Flowchart of literature search.

A total of 677 studies were examined for eligibility, of which nine papers were finally selected for eight studies, with a total of 81,943 patients who met the inclusion criteria (Figure 1). 1653 (2.1%) patients had MI. Eight studies presenting two NOAC classes were selected: DTI: RE-LY [23] and RE-DUAL PCI [4]: dabigatran 150 mg *n* = 6839, dabigatran 110 mg *n* = 6996 (total 13,835), and FXa inhibitors: ROCKET rivaroxaban 20 mg [24], PIONEER AF-PCI [3] rivaroxaban 20/15 mg *n* = 7840, ARISTOTLE [25] and AUGUSTUS [5] apixaban *n* = 11,426 and ENGAGE AF-TIMI 48 [26] edoxaban 30 mg *n* = 7034, edoxaban 60 mg *n* = 7035 and ENTRUST-AF PCI [6] edoxaban 60 mg *n* = 751 (total 14,820). All NOACs were compared with warfarin. In PIONEER AF-PCI, we included in the analysis the comparison between the dose of rivaroxaban 15 mg + clopidogrel vs. warfarin + clopidogrel + aspirin [3]. In the AUGUSTUS study [5], we analyzed patients treated and untreated with PCI and randomized to apixaban or warfarin group independent of additional aspirin or placebo. All studies were characterized by the high quality of realization (Supplementary Table S2). Three key studies were not included in the analysis-WOEST [1], ISAR-TRIPLE [2], and AVERROES [32]. Warfarin was used in both arms of the first two studies. In the AVERROES study in AF patients, the efficacy of apixaban vs. aspirin was compared [32]. The study details are presented in Table 1 and Table 2.

We analyzed the following endpoints: overall mortality, stroke, MI, MACE, ST, and cardiovascular or cardiac death. The first three were available in all eight studies. For the MI data in the RE-LY study, we used data from the initial study and the later version after correcting data made by authors [23,33]. The original RE-LY results did not include MACE rates [23]. In the re-analysis, Hohnloser et al. presented MI, MACE, and CD results in the intention-to-treat and on-treatment analysis [27]. Thus, we performed three comparisons for MI and two comparisons for MACE and cardiac death. In the RE-DUAL PCI study, only overall mortality was presented [4]. In the RE-LY study, overall mortality and vascular death were reported, while in the re-analysis by Hohnloser et al., the cardiac death rate was also presented [23,27]. We obtained ST data only from four studies comparing DAT vs. TAT [3,4,5,6].

### 3.2. Results of the Standard Meta-Analysis

#### 3.2.1. Myocardial Infarction

**Figure 2:**
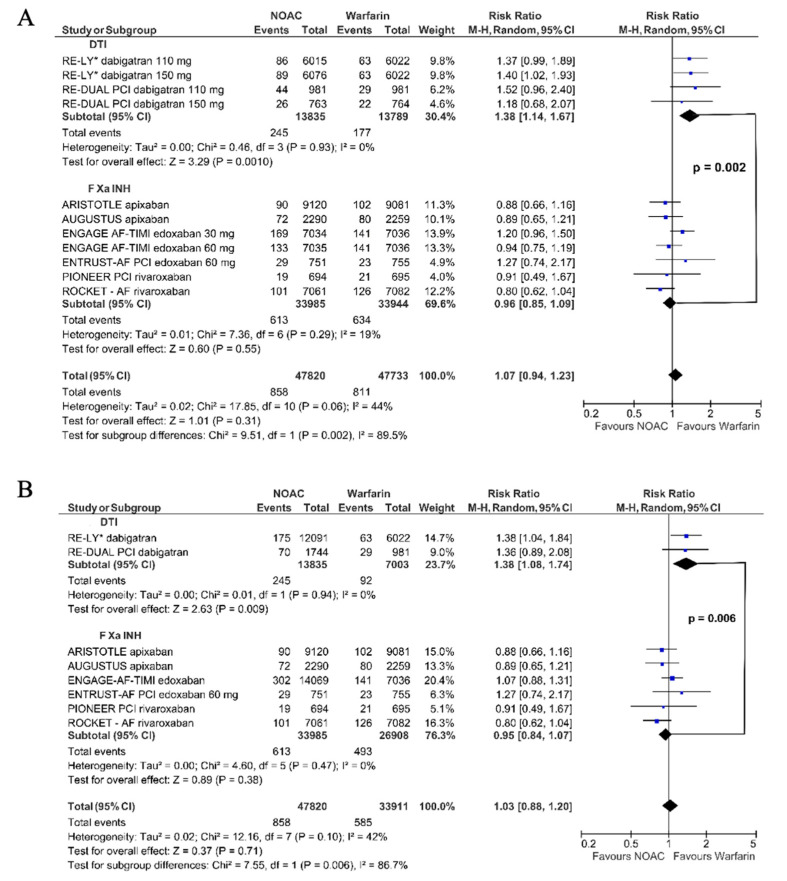
The meta-analysis results for myocardial infarction. * original data from RE-LY study [23]. (**A**)—dabigatran dose dependent analysis; (**B**)—dabigatran dose independent analysis.

Dabigatran compared to warfarin significantly increased the risk of MI (RR 1.38, 95% CI 1.14–1.67). The effect of FXa inhibitors did not differ significantly from warfarin (RR 0.96, 95% CI 0.86–1.09); however, the comparison of dabigatran vs. FXa inhibitors showed a significant difference (Chi^2^ = 9.51, df = 1, *p*_interaction_ = 0.002; Figure 2A). Similarly, in the model with combined dosages, the results remained consistent: dabigatran vs. warfarin (RR 1.38, 95% CI 1.08–1.74); FXa inhibitors vs. warfarin (RR 0.95, 95% CI 0.84–1.07); *p*_interaction_ for dabigatran vs. FXa inhibitors *p* = 0.006 (Figure 2B).

**Figure 3:**
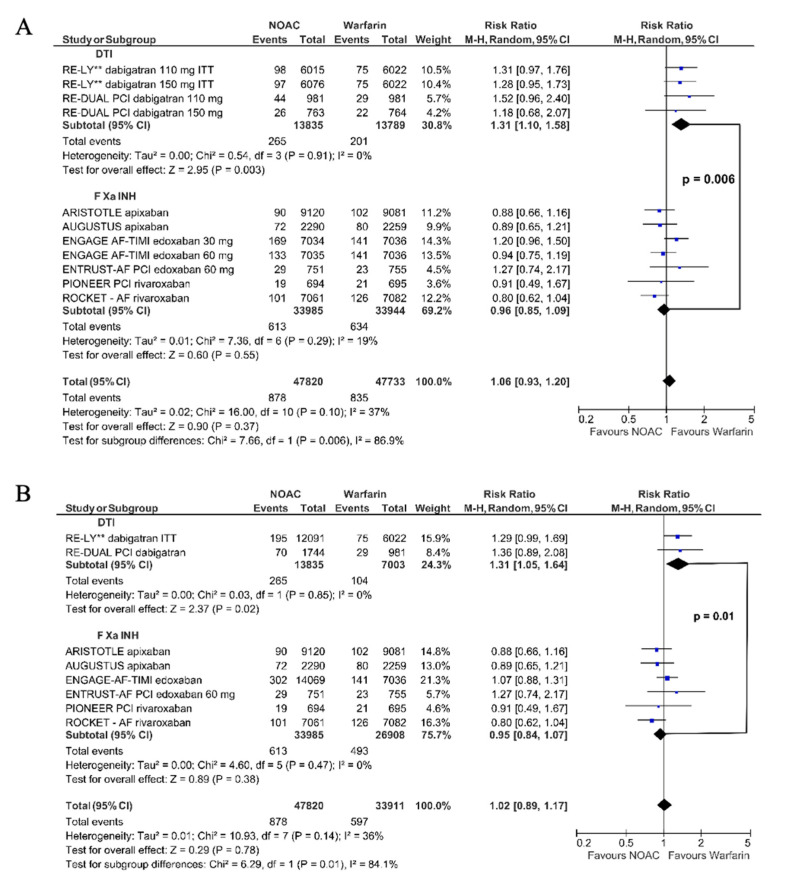
The meta-analysis results for myocardial infarction—the reanalysis RE-LY: intention to treat data. ** results from a reanalysis of the RE-LY study [27]; (**A**)—dabigatran dose dependent analysis; (**B**)—dabigatran dose independent analysis.

Considering the intention-to-treat analysis [30,31], the result was similar. Dabigatran increased the risk of MI (RR 1.31, 95% CI 1.10–1.58), but not FXa inhibitors (RR 0.96, 95% CI 0.85–1.09, Chi^2^ = 7.66 df = l (*p* = 0.006) (Figure 3A). The second approach yielded the same results: dabigatran vs. warfarin (RR 1.31, 95% CI 1.05–1.64); FXa inhibitors vs. warfarin (RR 0.95, 95% CI 0.84–1.07); *p*_interaction_: dabigatran vs. FXa inhibitors *p* = 0.01 (Figure 3B).

**Figure 4:**
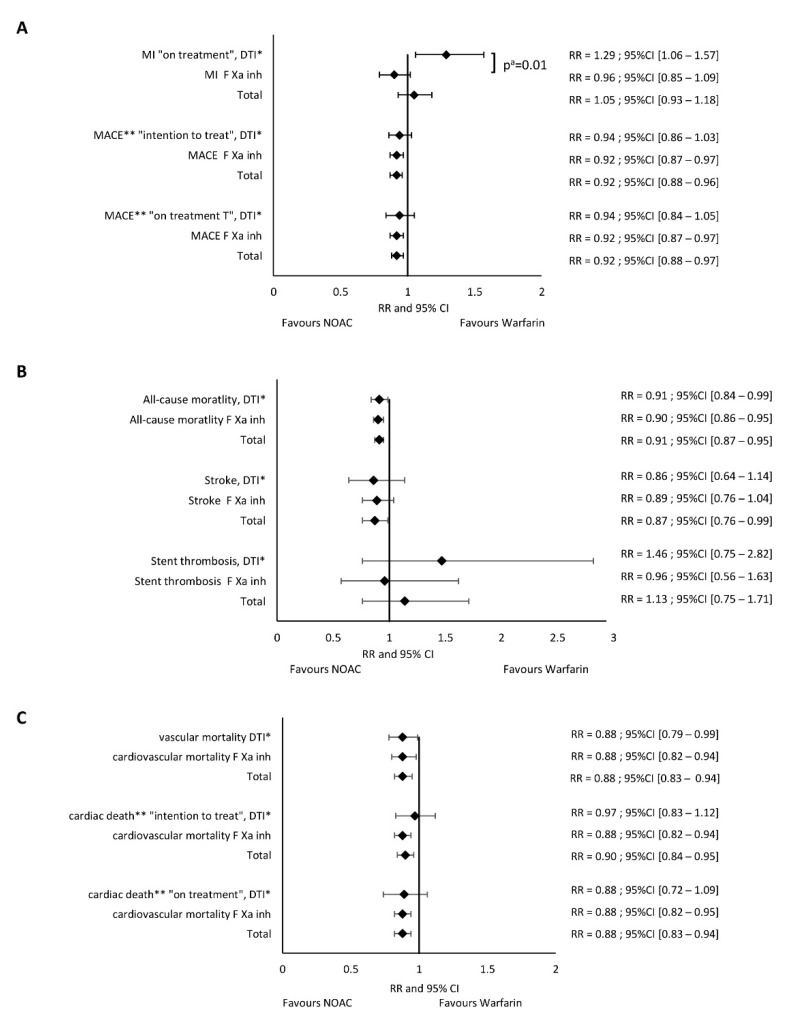
Forest plot of the relative risk of efficacy endpoints: (**A**): MI, MACE; (**B**): overall mortality, stroke, stent thrombosis; (**C**): cardiovascular mortality. ^a^—*p*-value for subgroup differences; * direct thrombin inhibitor–dabigatran; ** results from the reanalysis of the RE-LY study [27].

This result was confirmed in the on-treatment analysis: dabigatran (RR 1.29, 95% CI 1.06–1.57) vs. FXa inhibitors (RR 0.96, 95% CI 0.85–1.09, Chi^2^ 6.17, df = l, *p* = 0.01) (Figure 4A). The second model yielded the following results: dabigatran vs. warfarin (RR 1.29, 95% CI 1.01–1.64); FXa inhibitors vs. warfarin (RR 0.95, 95% CI 0.84–1.07); *p*_interaction_: dabigatran vs. FXa inhibitors *p* = 0.02 (Supplementary Figure S26A).

In the sensitivity analysis, after excluding edoxaban 30 mg, the risk of MI in patients treated with FXa inhibitors did not change (Supplementary Figures S20, S24 and S28). The results remained consistent also in the second model (combined dosages) (Supplementary Figures S20A, S24A and S28A).

#### 3.2.2. Major Adverse Cardiac Events

Dabigatran vs. warfarin did not significantly reduce the risk of MACE both in the intention-to-treat (RR 0.94, 95% CI 0.86–1.03) and in on-treatment analysis (RR 0.94, 95% CI 0.84–1.06, Figure 4A), while FXa inhibitors significantly reduced the risk of MACE (RR 0.92, 95% CI 0.87–0.97). The exclusion of 30 mg edoxaban did not significantly affect the results (Supplementary Figures S12 and S16). The pooled data showed that NOAC reduced the risk of MACE by 8% (RR 0.92, 95% CI 0.88–0.96, Figure 4A) compared to warfarin. We obtained the consistent results taking a second model in which the events of different dosages presented in one study were merged: intention-to-treat data: dabigatran vs. warfarin (RR 0.94, 95% CI 0.86–1.02); FXa inhibitors vs. warfarin (RR 0.91, 95% CI 0.87–0.96) (Supplementary Figure S10A); and on-treatment analysis: dabigatran vs. warfarin (RR 0.93, 95% CI 0.83–1.05); FXa inhibitors vs. warfarin (RR 0.91, 95% CI 0.87–0.96) (Supplementary Figure S14A); NOACs, all together vs. warfarin (RR 0.91, 95% CI 0.88–0.96).

#### 3.2.3. All-Cause Mortality

Both dabigatran and FXa inhibitors compared to warfarin significantly reduced overall mortality with RR 0.91, 95% CI 0.84–0.99 and RR 0.90, 95% CI 0.86–0.95, respectively (NOAC all together: RR 0.91 95% CI 0.87–0.96, Figure 4B). After excluding edoxaban 30 mg, the estimated risk indicators did not change significantly (Supplementary Figure S4). After combining data for different dosages, the results were as follows: dabigatran vs. warfarin-RR 0.91, 95% CI 0.82–1.01; FXa inhibitors vs. warfarin-RR 0.90, 95% CI 0.85–0.96; NOAC all together-RR 0.90 95% CI 0.86–0.95 (Supplementary Figure S2A). Further excluding data with edoxaban 30 mg led to the same results (Supplementary Figure S4A).

#### 3.2.4. Stroke

Compared to warfarin, both dabigatran and FXa inhibitors similarly reduced the risk of stroke: RR 0.86, 95% CI 0.65–1.14 and RR 0.89, 95% CI 0.76–1.04, respectively. We also confirmed this in the pooled analysis: NOACs vs. warfarin (RR 0.87, 95% CI 0.76–0.99, Figure 4B). After excluding edoxaban 30 mg, for the remaining FXa inhibitors vs. warfarin, RR for stroke was 0.84, 95% CI 0.76–0.92 (Supplementary Figure S8). The second model provided the following results: dabigatran vs. warfarin-RR 0.81, 95% CI 0.67–0.98; FXa inhibitors vs. warfarin-RR 0.86, 95% CI 0.74–1.01; NOAC together-RR 0.86 95% CI 0.76–0.97 (Supplementary Figure S6A). After exclusion of edoxaban 30 mg, we observed the significant stroke reduction for both types of NOAC: dabigatran vs. warfarin-RR 0.81, 95% CI 0.67–0.98; FXa inhibitors vs. warfarin-RR 0.84, 95% CI 0.76–0.92); and NOAC together-RR 0.83 95% CI 0.76–0.90 (Supplementary Figure S8A).

#### 3.2.5. Stent Thrombosis

ST was evaluated in only four studies: PIONEER [3], RE-DUAL PCI [4], AUGUSTUS [5], and ENTRUST-AF PCI [6]. In total, the use of NOACs compared to warfarin was associated with a similar risk of ST (RR 1.13, 95% CI 0.75–1.71). Dabigatran increased the risk of ST by 1.46-fold (RR 1.46, 95% CI 0.75–2.82), while FXa inhibitors decreased this risk (RR 0.96, 95% CI 0.67–1.62, Figure 4B). The second model provided similar results: dabigatran-RR 1.55, 95% CI 0.69–3.46; FXa inhibitors vs. warfarin-RR 0.96, 95% CI 0.56–1.63; NOAC all together-RR 1.11 95% CI 0.71–1.73 (Supplementary Figure S30A).

#### 3.2.6. Cardiovascular Death

Cardiovascular death was not reported in ARISTOTLE and REDUAL PCI, while RE-LY reported vascular death [23] and after data correction-cardiac death [27] (Supplementary Table S1C). Dabigatran vs. warfarin moderately (12%) but significantly reduced cardiovascular death risk (RR 0.88, 95% CI 0.79–0.99, Figure 4C). After data correction [27] in intention-to-treat and on-treatment analyses, RR for cardiovascular death was 0.97 (95% CI 0.83–1.12) and RR 0.88 (95% CI 0.72–1.09), respectively (Figure 4C). FXa inhibitors compared to warfarin also moderately reduced the risk of cardiovascular death (RR 0.88, 95% CI 0.82–0.94, Figure 4C). In all three comparisons, NOAC vs. warfarin significantly reduced the relative risk of cardiovascular death in the range of 10–12% (RR 0.88, 95% CI 0.83–0.94, RR 0.90, 95% CI 0.84–0.96 and RR 0.88, 95% CI 0.82–0.94, Figure 4C). The result was consistent after excluding edoxaban 30 mg (Supplementary Figures S34, S38 and S42). In the second model, dabigatran did not significantly reduce the cardiovascular death risk (RR 0.88, 95% CI 0.77–1.01, Supplementary Figure S32A), and after data correction [27] in intention-to-treat and on-treatment analyses, the results were as follows: RR 0.97, 95% CI 0.81–1.16 (Supplementary Figure S36A) and RR 0.89, 95% CI 0.71–1.10 (Supplementary Figure S34A), respectively. In all three comparisons, NOAC vs. warfarin significantly reduced the relative risk of cardiovascular death: RR 0.88, 95% CI 0.81–0.96 (Supplementary Figure S32A); RR 0.88, 95% CI 0.81–0.96 (Supplementary Figure S36A) and RR 0.89, 95% CI 0.82–0.96 (Supplementary Figure S40A). Similarly, the result was consistent after excluding edoxaban 30 mg (Supplementary Figures S34A, S38A and S42A).

### 3.3. Results of the Network Meta-Analysis

**Figure 5:**
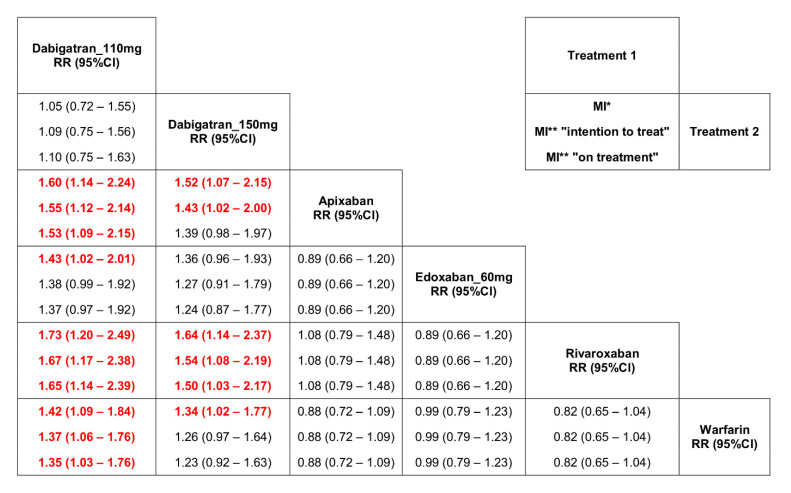
Direct and indirect comparison between warfarin and NOACs for myocardial infarction. * original data from RE-LY study [23], ** results from reanalysis RE-LY study [27]. The red colour shows significant differences.

Dabigatran at the dose of 110 mg b.i.d. as well as at the dose of 150 mg b.i.d. increased the risk of MI compared to warfarin, apixaban, and rivaroxaban (Figure 5). All analyzed drugs except dabigatran 110 mg b.i.d. significantly reduced the risk of MACE (Supplementary Tables S7 and S9). The estimated risk indicators for stroke and overall mortality were similar and did not differ between drugs (Supplementary Tables S13 and S15).

### 3.4. Results of the Analysis with SUCRA

**Figure 6:**
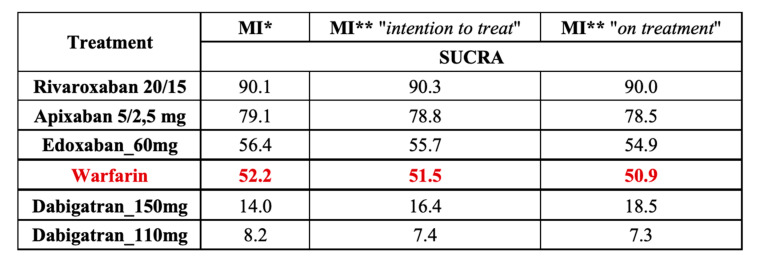
The surface under the cumulative ranking curve (SUCRA) of myocardial infarction. * original data from RE-LY study [23]; ** results from reanalysis RE-LY study [27]. The red numbers refer to warfarin as the comparator for all investigated drugs.

We also estimated a hierarchy of competitive treatments using SUCRA. Rivaroxaban doses 20 and 15 mg taken once daily showed the highest probability of being the most effective treatment in reducing the risk of MI (Figure 6), MACE, and overall mortality (Supplementary Tables S4, S6 and S16). Dabigatran 150 mg b.i.d. was the most effective in reducing the risk of stroke. Dabigatran 110 mg b.i.d. had the weakest effect on the ischemic events, whereas apixaban and edoxaban had a moderate impact.

## 4. Discussion

Dabigatran, in contrast to FXa inhibitors, compared to warfarin significantly increased the risk of MI by 1.38-fold. Comparing dabigatran vs. FXa inhibitors, a significant difference between the risk estimators was shown (*p* = 0.002) (Figure 2). After the correction of the RE-LY data [23,27,33], the observed effect of dabigatran on MI still significantly differed from that of warfarin or FXa inhibitors (Figure 3 and Figure 4A).

NOAC, compared to warfarin, had a favorable risk-benefit profile [34]. However, in the RE-LY study [23] in patients with AF, the number of patients with MI was higher in dabigatran than in the warfarin group. Many meta-analyses showed that dabigatran treatment led to an increased risk of MI [16,17,18,19,20,21,22]. These analyses included patients with AF and sinus rhythm at the same time. Dabigatran was used for various indications (prevention of thromboembolic events, deep vein thrombosis, ACS) and was compared with placebo/aspirin, warfarin, and enoxaparin. Therefore, it is questioned whether the results can be extrapolated to patients with AF undergoing PCI. Data from large RWD did not confirm an increase in the risk of MI during dabigatran treatment [35,36,37,38,39]. However, in patients with AF, the switch from warfarin to dabigatran treatment resulted in an increased risk of MI compared to naive patients [40]. The discrepancies between RCT and RWD findings result from different study designs and different confounding variables. Without questioning the informative value of RWD, RCTs still represent the ‘gold standard’ of clinical trials [41,42,43,44,45]. We excluded RWD from our analysis, and each of the four drugs was evaluated in two key phase III RCT: in patients with AF and patients with AF and CCS or ACS treated with PCI. In each study, warfarin was the comparator for NOAC. In this homogeneous group of patients, dabigatran in the direct comparison with warfarin significantly increased the risk of MI by about 30%. Moreover, the risk of MI was also significantly higher than the opposite effect of FXa inhibitors vs. warfarin. In our network meta-analysis, taking into account individual NOACs in recommended doses, only in patients treated with dabigatran 150 mg b.i.d. and especially with dabigatran 110 mg b.i.d., we found an increased risk of MI compared to warfarin (Figure 5). Our observations are consistent with the results of previously published network meta-analyses [18,19,46,47]. NOACs pooled together compared to warfarin significantly reduced the risk of overall mortality, cardiovascular mortality, stroke, and MACE. After considering the division into classes: DTI and FXa inhibitors, the directions of changes in both subgroups were consistent (Figure 4). The risk of MACE was significantly reduced only in the FXa inhibitors subgroup (Figure 4A). One might suppose that a lower but nonsignificant reduction of MACE and cardiac death in patients treated with dabigatran might have resulted from the increased number of patients with MI. Moreover, a higher risk of ST in these patients also deserves attention (Figure 4B). The final answer may be available from the randomized studies comparing FXa inhibitors and DTI in patients with AF and ACS treated with PCI.

Patients with AF treated with NOAC and undergoing PCI [3,4,5,6] differ from patients with AF treated on chronic NOAC therapy [23,24,25,26]. They characterize a higher risk of ACS and the necessity of DAPT use (usually clopidogrel + aspirin). However, independent of ACS risk and percentage of patients on chronic NOAC therapy treated with aspirin (29–40%) as well as in contrast to FXa inhibitors, dabigatran increased the risk of MI.

The mechanism of increased risk of MI during DTI treatment has not been fully understood. Warfarin suppresses thrombin generation more efficiently than dabigatran [48]. The effects of DTI depend on its plasma concentration, and its activity decreased at trough levels. When the concentrations of DTI decline below therapeutic ranges, the paradoxical impact (enhancement of thrombin generation) might occur.

The mechanism of the paradoxical coagulation activation by DTI may be suppressing the thrombin-thrombomodulin (TM)-induced negative feedback by inhibiting protein-C activation [49]. Artang et al. suggested that at DTI trough levels, the remaining enzymatically active thrombin dissociated from DTI molecules when exposed to tissue factor at the site of a ruptured atherosclerotic plaque, and thrombin generation increased [19]. Direct FXa inhibitors did not enhance thrombin generation in human plasma in the absence and the presence of thrombin-thrombomodulin and protein-C. Thus, FXa inhibitors are less prone to induce coagulation [49]. Some authors proved that dabigatran increased platelet reactivity by enhancing the thrombin receptor density (PAR-I PAR-4) on platelets [50]. Others suggested that DTIs therapy increased inflammatory markers in patients with MI [51]. These results provide arguments to justify the increased risk of MI in patients treated with dabigatran.

The benefit of dabigatran for stroke prevention supported the clinical opinion that it “seems to outweigh the small increase in the risk of MI” [41]. However, the situation changed after publishing the first RCT in patients with AF qualified for PCI and treated with standard TAT vs. DAT containing rivaroxaban (PIONEER AF-PCI) [3]. Subsequent studies based on a similar protocol were performed with dabigatran [4], apixaban [5], and edoxaban [6]. They all reported a significant reduction in hemorrhagic complications and the lack of substantial effect on MACE rates. These results started a debate on the optimal combination of OAC and antiplatelet therapy. The discussion focused on antiplatelet treatment and the recommendation of using NOAC over warfarin without considering differences between FXa inhibitors and DTI.

Therefore, we believe that in patients with AF and undergoing PCI, the choice of NOAC (FXa inhibitors vs. DTI) is as important as choosing the optimal antiplatelet therapy (DAPT vs. SAPT). Additionally, using the SUCRA score, we estimated the treatment ranking of the best therapy for MI prevention in patients with AF (Figure 6). Rivaroxaban had a 90% probability of being ranked the best therapy for MI prevention, whereas dabigatran 110 mg had only an 8.2% probability.

However, dabigatran 150 mg was the most effective in stroke prevention (a 94% probability). Conversely, dabigatran 110 mg was the worst in stroke prevention among all analyzed NOACs with a 24.5% probability. Rivaroxaban was ranked to be the best therapy with respect to MACE and overall mortality. More potent antiplatelet drugs (e.g., ticagrelor) may optimize the risk of MI related to dabigatran therapy, especially at a dose of 110 mg b.i.d., although this strategy needs to be confirmed in randomized trials.

The estimated number needed to harm (NNH) for both doses, including the original publication [23], is 219 (1057-122), while separately 184 and 231 for the 110 mg and 150 mg doses, respectively. The similar estimations from the RE-LY re-analysis [27] are 232 (1949-123) for both doses, and 184 and 268 for 110 mg and 150 mg, respectively. These results suggest that the risk of MI in patients treated with dabigatran, though low, is significant and that a higher risk is related to a dose of 110 mg.

The newest European Society of Cardiology guidelines on the management of patients with AF and non-ST elevation myocardial infarction [14,15] recommend the 110 mg dose of dabigatran in preference to 150 mg to mitigate the bleeding risk.

Our study proved an increased risk of MI in patients treated with both doses of dabigatran–110 mg and 150 mg. Therefore, we recommend that FXa inhibitors should be considered in the first line in patients with AF and concomitant coronary artery disease.

### Limitations

The limitation of our study was the different definitions of endpoints in individual studies-MACE, cardiovascular mortality, or vascular mortality. Another limitation is an indirect comparison of individual drugs through a common comparator–warfarin, because it was not possible to assess direct effects between the analyzed drugs. This is related to the impossibility of evaluating the consistency between direct and indirect effects, which is the basic assumption of the network meta-analysis. However, the advantage of our meta-analysis is that it only applies to randomized studies, which are free of bias. An additional advantage is that each of the analyzed arms was balanced and included patients with AF treated medically and patients with AF and ACS treated with PCI.

## 5. Conclusions

Each NOAC was associated with a different risk of MI. Dabigatran in both doses characterized a higher risk of MI compared to warfarin and FXa inhibitors. Furthermore, FXa inhibitors should be considered the first line NOACs in patients with AF and coronary artery disease.

## Figures and Tables

**Table 1:** Characteristics of patients in clinical trials included in the meta-analysis and not treated with PCI.

Characteristics	RE-LY			ROCKET AF		ENGAGE AF TIMI 48			ARISTOTLE	
Treatment/dose	Dabigatran 110 mg (*n* = 6015)	Dabigatran 150 mg (*n* = 6076)	Warfarin (*n* = 6022)	Rivaroxaban 20 mg or 15 mg daily if CrCl 30–49 mL/min (*n* = 7131)	Warfarin (*n* = 7133)	High Dose Edoxaban (*n* = 7035)	Low Dose Edoxaban (*n* = 7034)	Warfarin (*n* = 7036)	Apixaban 5 mg twice daily or 2.5 mg twice daily (*n* = 9120)	Warfarin (*n* = 9081)
Age, y, mean (SD) or median	Mean (SD) 71.4 (8.6)	Mean (SD) 71.5 (8.8)	Mean (SD) 71.6 (8.6)	Median 73	Median 73	Median 72	Median 72	Median 72	Median 70	Median 70
Female, *n* (%)	2150 (35.7)	2236 (36.8)	2213 (36.7)	2831 (39.7)	2832 (39.7)	2669 (37.9)	2730 (38.8)	2641 (37.5)	3234 (35.5)	3182 (35.0)
Renal function, *n* (%) or as indicated otherwise	NA	NA	NA	Median CrCl 67 mL/min	Median CrCl 67 mL/min	CrCl ≤ 50 mL/min 1379 (19.6)	CrCl ≤ 50 mL/min 1334 (19.0)	CrCl ≤ 50 mL/min 1361 (19.3)	CrCl ≤ 50 mL/min 1502 (16.5)	CrCl ≤ 50 mL/min 1515 (16.7)
CHA_2_DS_2_VASc, mean (SD)	2.1 (1.1)	2.2 (1.2)	2.1 (1.1)	3.48 (0.94)	3.46 (0.95)	2.8 (1.0)	2.8 (1.0)	2.8 (1.0)	2.1 (1.1)	2.1 (1.1)
HAS-BLED, mean (SD) or <3 vs. ≥3	NA	NA	NA	NA	NA	NA	NA	NA	NA	NA
Hypertension, *n* (%)	4738 (78.8)	4795 (78.9)	4750 (78.9)	6436 (90.3)	6474 (90.8)	6591 (93.7)	6575 (93.5)	6588 (93.6)	7962 (87.3)	7954 (87.6)
Diabetes mellitus, *n* (%)	1409 (23.4)	1402 (23.1)	1410 (23.4)	2878 (40.4)	2817 (39.5)	2559 (36.4)	2544 (36.2)	2521 (35.8)	2284 (25.0)	2263 (24.9)
History of stroke or TIA, *n* (%) or systemic embolism	1195 (19.9)	1233 (20.3)	1195 (19.8)	3916 (54.9)	3895 (54.6)	1976 (28.1)	2006 (28.5)	1991 (28.3)	1748 (19.2)	1790 (19.7)
History of myocardial infarction, *n* (%)	1008 (16.8)	1029 (16.9)	968 (16.1)	1182 (16.6)	1286 (18.0)	NA	NA	NA	1319 (14.5)	1266 (13.9)
History of CABG, *n* (%)	NA	NA	NA	NA	NA	NA	NA	NA	NA	NA
History of PCI, *n* (%)	NA	NA	NA	NA	NA	NA	NA	NA	NA	NA
ASA	2404 (40.0)	2352 (38.7)	2442 (40.6)	2586 (36.3)	2619 (36.7)	2070 (29.4)	2018 (28.7)	2092 (29.7)	2859 (31.3)	2773 (30.5)

ASA—aspirin, CABG—coronary artery bypass grafting, CrCl—creatinine clearance, PCI—percutaneous angioplasty, TIA—transient ischemic attack.

**Table 2:** Characteristics of patients in clinical trials included in the meta-analysis and treated with PCI.

Characteristics	PIONEER-AF PCI, 2016		RE-DUAL PCI, 2017				AUGUSTUS, 2019		ENTRUST-AF PCI, 2019	
Therapy	DT (*n* = 709) Rivaroxaban (15 mg) + P2Y_12_ inhibitor (clopidogrel, 75 mg, or ticagrelor, 2 × 90 mg, or prasugrel, 10 mg)	TT (*n* = 706) VKA + ASA (75–100 mg) + P2Y_12_ inhibitor (clopidogrel, 75 mg, or ticagrelor, 2 × 90 mg, or prasugrel, 10 mg)	DT (*n* = 981) Dabigatran (2 × 110 mg) + P2Y_12_ inhibitor (clopidogrel, 75 mg, or ticagrelor, 2 × 90 mg)	DT (*n* = 763) Dabigatran (2 × 150 mg) + P2Y_12_ inhibitor (clopidogrel, 75 mg, or ticagrelor, 2 × 90 mg)	TT (*n* = 981) VKA + ASA (<100 mg) + P2Y_12_ inhibitor (clopidogrel, 75 mg, or ticagrelor, 2 × 90 mg)	TT (*n* = 764) Corresponding TT VKA	DT/TT (*n* = 2306) Apixaban (2 × 5 mg or 2 × 2.5 mg) + P2Y_12_ inhibitor + ASA, 81 mg/placebo	DT/TT (*n* = 2308) VKA + P2Y_12_ inhibitor + ASA, 81 mg/placebo	DT (*n* = 751) Edoxaban (60 mg/or 30 mg in specific indication) + P2Y_12_ inhibitor	TT (*n* = 755) VKA + P2Y_12_ inhibitor + ASA (100 mg, for 1–12 months)
Age, y, mean (SD)	70.4 (9.1)	69.9 (8.7)	71.5 (8.9)	68.6 (7.7)	71.7 (8.9)	68.8 (7.7)	70.4	70.9	69	70
Female sex, *n* (%)	181 (25.5)	188 (26.6)	253 (25.8)	171 (22.4)	231 (23.5)	170 (22.3)	670 (29.1)	667 (28.9)	194 (26)	192 (25)
Renal function, *n* (%) or as indicated otherwise	Mean (SD) CrCl, 78.3 (31.3) mL/min	Mean (SD) CrCl, 80.7 (30) mL/min	Mean (SD) CrCl, 76.3 (28.9) mL/min	Mean (SD) CrCl, 83.7 (31) mL/min	Mean (SD) CrCl, 75.4 (29.1) mL/min	Mean (SD) CrCl, 81.3 (29.6) mL/min	creat ≥ 1.5 mg/dL 173 (7.6)	creat ≥ 1.5 mg/dL 207 (9.2)	Mean 71.8 mL/min	Mean 71.7 mL/min
Type of index event, *n* (%)	NSTEMI, 130 (18.5) STEMI, 86 (12.3) UA, 145 (20.7)	NSTEMI, 123 (17.8) STEMI, 74 (10.7) UA, 164 (23.7)	ACS, 509 (51.9) CCS, 433 (44.1) Staged PCI, 156 (15.9) Other 43 (4.4)	ACS, 391 (51.2) CCS, 320 (41.9) Staged PCI, 138 (18.1) Other, 65 (8.5)	ACS, 475 (48.4) CCS, 429 (43.7) Staged PCI, 168 (17.1) Other, 62 (6.3)	ACS, 369 (48.3) CCS, 339 (44.4) Staged PCI, 134 (17.5) other 50 (6.5)	ACS and PCI, 873 (38) ACS -medical therapy, 547 (23.8) Elective PCI, 877 (38.2)	ACS and PCI, 841 (36.6) ACS -medical therapy, 550 (23.9) Elective PCI, 907 (39.5)	ACS 388 (52), CCS 363 (48)	ACS 389 (52), CCS 366 (48)
CHA_2_DS_2_VASc, mean (SD)	3.7 (1.7)	3.8 (1.6)	3.7 (1.6)	3.3 (1.5)	3.8 (1.5)	3.5 (1.5)	3.9 (1.6)	4.0 (1.6)	4	4
HAS-BLED, mean (SD)	NA	NA	2.7 (0.7)	2.6 (0.7)	2.8 (0.8)	2.7 (0.8)	2.9 (1.0)	2.9 (0.9)	3	3
Hypertension, *n* (%)	NA	NA	NA	NA	NA	NA	2042 (88.6)	2031 (88)	674 (90)	687 (91)
Diabetes mellitus, *n* (%)	NA	NA	362 (36.9)	260 (34.1)	371 (37.9)	303 (39.7)	842 (36.5)	836 (36.2)	259 (34)	258 (34)
Stroke or TIA, *n* (%)	NA	NA	74 (7.5)	52 (6.8)	100 (10.2)	77 (10.1)	326 (14.2)	307 (13.4)	97 (13)	92 (12)
History of myocardial infarction, *n* (%)	20%	22%	237 (24.2)	194 (25.4)	268 (27.3)	211 (27.6)	NA	NA	188 (25)	177 (23)
History of CABG, *n* (%)	NA	NA	97 (9.9)	79 (10.4)	111 (11.3)	87 (11.4)	NA	NA	46 (6)	49 (6)
History of PCI, *n* (%)	NA	NA	326 (33.2)	239 (31.3)	347 (35.4)	272 (35.6)	NA	NA	199 (26)	195 (26)

ASA—aspirin, CABG—coronary artery bypass grafting, CrCl—creatinine clearance, PCI—percutaneous angioplasty, TIA—transient ischemic attack.

## Data Availability

The data that supports the findings of this study are available in the Supplementary Materials of this article.

## Supplementary Materials

The following are available online at https://www.mdpi.com/article/10.3390/jpm11101013/s1. Table S1A: The characteristics of studies included in meta-analysis, Table S1B: MACE (++) and thromboembolic or ischemic end points (+), Table S1C: Characteristics of patients in clinical trials included in the meta-analysis—RE-LY, ROCKET AF, ENGAGE AF TIMI 48, ARISTOTLE, Table S1D: Charcteristics of patients in clinical trials included in the meta-analysis—PIONEER-AF PCI, RE-DUAL PCI, AUGUSTUS, ENTRUST-AF-PCI, Table S2: The risk of bias of individual studies by Cochrane Risk Assessment Tool, Table S3: Direct and indirect comparison between Warfarin and NOAC’s—MACE**—inetntion-to-treat data, Table S4: Treatment hierarchy assessed by surface under the cumulative ranking (SUCRA) curves—MACE**—intention-to-treat data, Table S5: Direct and indirect comparison between Warfarin and NOAC’s—MACE**—on-treatment data, Table S6: Treatment hierarchy assessed by surface under the cumulative ranking (SUCRA) curves—MACE**—on-treatment data, Table S7: Direct and indirect comparison between Warfarin and NOAC’s—MI* data, Table S8: Treatment hierarchy assessed by surface under the cumulative ranking (SUCRA) curves—MI*, Table S9: Direct and indirect comparison between Warfarin and NOAC’s—MI**—intentio-to-treat data, Table S10: Treatment hierarchy assessed by surface under the cumulative ranking (SUCRA) curves—MI**—intentio-to-treat data, Table S11: Direct and indirect comparison between Warfarin and NOAC’s—MI*—on-treatment data, Table S12: Treatment hierarchy assessed by surface under the cumulative ranking (SUCRA) curves—MI**—on-treatment data, Table S13: Direct and indirect comparison between Warfarin and NOAC’s—stroke data, Table S14: Treatment hierarchy assessed by surface under the cumulative ranking (SUCRA) curves—stroke data, Table S15: Direct and indirect comparison between Warfarin and NOAC’s—overall mortality data, Table S16: Treatment hierarchy assessed by surface under the cumulative ranking (SUCRA) curves—overall mortality data, Figure S1: Flowchart of literature search, Figure S2: The meta-analysis results for all-cause mortality, Figure S2A: The meta-analysis results for all-cause mortality after combining study data with respect to doses, Figure S3: The funnel plot for all-cause mortality, Figure S4: The meta-analysis results for all-cause mortality after excluding ENGAGE AF-TIMI Edoxaban 30 mg, Figure S4A: The meta-analysis results for all-cause mortality after excluding ENGAGE AF-TIMI Edoxaban 30 mg and combining study data with respect to doses, Figure S5: The funnel plot for all-cause mortality after excluding ENGAGE AF-TIMI Edoxaban 30 mg, Figure S6: The meta-analysis results for stroke, Figure S6A: The meta-analysis results for stroke after combining study data with respect to doses, Figure S7: The funnel plot for stroke, Figure S8: The meta-analysis results for stroke after excluding ENGAGE AF-TIMI Edoxaban 30 mg, Figure S8A: The meta-analysis results for stroke after excluding ENGAGE AF-TIMI Edoxaban 30 mg and combining study data with respect to doses, Figure S9: The funnel plot for stroke after excluding ENGAGE AF-TIMI Edoxaban 30 mg, Figure S10: The meta-analysis results for MACE—reanalysis RE-LY—intention-to-treat data, Figure S10A: The meta-analysis results for MACE—reanalysis RE-LY—intention-to-treat data after comining study data with respect to doses, Figure S11: The funnel plot for MACE—reanalysis RE-LY– intention-to-treat data, Figure S12: The meta-analysis results for MACE—reanalysis RE-LY—intention-to-treat data after excluding ENGAGE AF-TIMI Edoxaban 30 mg, Figure S12A: The meta-analysis results for MACE—reanalysis RE-LY—intention-to-treat data after excluding ENGAGE AF-TIMI Edoxaban 30 mg and combining study data with respect to doses, Figure S13: The funnel plot for MACE—reanalysis RE-LY—intention-to-treat data after excluding ENGAGE AF-TIMI Edoxaban 30 mg, Figure S14: The meta-analysis results for MACE—reanalysis RE-LY—on-treatment data, Figure S14A: The meta-analysis results for MACE—reanalysis RE-LY—on-treatment data after combining study data with respect to doses, Figure S15: The funnel plot for MACE—reanalysis RE-LY—on-treatment data, Figure S16: The meta-analysis results for MACE—reanalysis RE-LY—on-treatment data after excluding ENGAGE AF-TIMI Edoxaban 30 mg, Figure S16A: The meta-analysis results for MACE—reanalysis RE-LY—on-treatment data after excluding ENGAGE AF-TIMI Edoxaban 30 mg and combining study data with respect to doses, Figure S17: The funnel plot for MACE—reanalysis RE-LY—on-treatment data after excluding ENGAGE AF-TIMI Edoxaban 30 mg, Figure S18: The meta-analysis results for MI, Figure S18A: The meta-analysis results for MI after combining study data with respect to doses, Figure S19: The funnal plot for MI, Figure S20: The meta-analysis results for MI after excluding ENGAGE AF-TIMI Edoxaban 30 mg, Figure S20A: The meta-analysis results for MI after excluding ENGAGE AF-TIMI Edoxaban 30 mg and combining study data with respect to doses, Figure S21: The funnel plot for MI after excluding ENGAGE AF-TIMI Edoxaban 30 mg, Figure S22: The meta-analysis results for MI—reanalysis RE-LY—intention-to-treat, Figure S22A: The meta-analysis results for MI—reanalysis RE-LY—intention-to-treat data after combining study data with respect to doses, Figure S23: The funnel plot for MI—reanalysis RE-LY—inetntion-to-treat data, Figure S24: The meta-analysis results for MI—reanalysis RE-LY—intentio-to-treat data after excluding ENGAGE AF-TIMI Edoxaban 30 mg, Figure S24A: The meta-analysis results for MI—reanalysis RE-LY—intention-to-treat data after excluding ENGAGE AF-TIMI Edoxaban 30 mg and combining study data with respect to doses, Figure S25: The funnel plot for MI—reanalysis RE-LY—intention-to-treat data after excluding ENGAGE AF-TIMI Edoxaban 30 mg, Figure S26: The meta-analysis results for MI—reanalysis RE-LY—on-treatment data, Figure S26A: The meta-analysis results for MI—reanalysis RE-LY—on-treatment data combining study data with respect to doses, Figure S27: The funnel plot for MI—reanalysis RE-LY—on-treatment data, Figure S28: The meta-analysis results for MI—reanalysis RE-LY—on-treatment data after excluding ENGAGE AF-TIMI Edoxaban 30 mg, Figure S28A: The meta-analysis results for MI—reanalysis RE-LY—on-treatment data after excluding ENGAGE AF-TIMI Edoxaban 30 mg and combining study data with respect to doses, Figure S29: The funnel plot for MI—reanalysis RE-LY—on-treatment data after excluding ENGAGE AF-TIMI Edoxaban 30 mg, Figure S30: The meta-analysis results for stent thrombosis, Figure S30A: The meta-analysis results for stent thrombosis after combining study data with respect to doses, Figure S31: The funnel plot for stent thrombosis, Figure S32 The meta-analysis results for cardiovascular mortality, Figure S32A: The meta-analysis results for cardiovascular mortality after combinig study data with respect to doses, Figure S33: The funnel plot for cardiovascular mortality, Figure S34: The meta-analysis results for cardiovascular mortality after excluding ENGAGE AF-TIMI Edoxaban 30 mg, Figure S34A: The meta-analysis results for cardiovascular mortality after excluding ENGAGE AF-TIMI Edoxaban 30 mg and combining study data with respect to doses, Figure S35: The funnel plot for cardiovascular mortality after excluding ENGAGE AF-TIMI Edoxaban 30 mg, Figure S36: The meta-analysis results for cardiovascular mortality—reanalysis RE-LY—intention-to-treat data, Figure S36A: The meta-analysis results for cardiovascular mortality—reanalysis RE-LY—intention-to-treat data after combining study data with respect to doses, Figure S37: The funnel plot for cardiovascular mortality—reanalysis RE-LY—intention-to-treat data, Figure S38: The meta-analysis results for cardiovascular mortality—reanalysis RE-LY—intention-to-treat data after excluding ENGAGE AF-TIMI Edoxaban 30 mg, Figure S38A: The meta-analysis results for cardiovascular mortality—reanalysis RE-LY—intention-to-treat data after excluding ENGAGE AF-TIMI Edoxaban 30 mg and combining study data with respect to doses, Figure S39: The funnel plot for cardiovascular mortality—reanalysis RE-LY—intention-to-treat data after excluding ENGAGE AF-TIMI Edoxaban 30 mg, Figure S40: The meta-analysis results for cardiovascular mortality—reanalysis RE-LY—on-treatment data, Figure S40A: The meta-analysis results for cardiovascular mortality—reanalysis RE-LY—on-treatment data after combining study data with respect to doses, Figure S41: The funnel plot for cardiovascular mortality—reanalysis RE-LY—on-treatment data, Figure S42: The meta-analysis results for cardiovascular mortality—reanalysis RE-LY—on-treatment data after excluding ENGAGE AF-TIMI Edoxaban 30 mg, Figure S42A: The meta-analysis results for cardiovascular mortality—reanalysis RE-LY—on-treatment data after excluding ENGAGE AF-TIMI Edoxaban 30 mg and combining study data with respect to doses, Figure S43: The funnel plot for cardiovascular mortality—reanalysis RE-LY—on-treatment data after excluding ENGAGE AF-TIMI Edoxaban 30 mg, Figure S44: The network plot of compared NOAC’s and Warfarin, Figure S45: Rankograms for the drugs network showing the probability every treatment being at particular order—MACE**—intention-to-treat data, Figure S46: Rankograms for the drugs network showing the probability every treatment being at particular order—MACE**—on-treatment data, Figure S47: Rankograms for the drugs network showing the probability every treatment being at particular order—MI* data, Figure S48: Rankograms for the drugs network showing the probability every treatment being at particular order—MI**—intention-to-treat data, Figure S49: Rankograms for the drugs network showing the probability every treatment being at particular order—MI**—on-treatment data, Figure S50: Rankograms for the drugs network showing the probability every treatment being at particular order—stroke data, Figure S51: Rankograms for the drugs network showing the probability every treatment being at particular order—overall moratlity data, Additional calculations necessary for the logical sequence of the meta-analysis are provided.
