# Supplementary material for: Non-Vitamin K Antagonist Oral Anticoagulants and Risk of Myocardial Infarction in Patients with Atrial Fibrillation with or without Percutaneous Coronary Interventions: A Meta-Analysis
Source: J Pers Med. 2021 Oct 9;11(10):1013. doi: 10.3390/jpm11101013 (PMC8539187; doi:10.3390/jpm11101013)

Supplementary material

FIGURE S1. FLOWCHART OF LITERATURE SEARCH. 6

DETAILED STATISTICAL METHODS: DATA SYNTHESIS AND ANALYSIS 7

TABLE S1A. THE CHARACTERISTICS OF STUDIES INCLUDED IN META-ANALYSIS. 8

TABLE S1B . MACE (++) AND THROMBOEMBOLIC OR ISCHEMIC END POINTS (+) 12

TABLE S1C. CHARACTERISTICS OF PATIENTS IN CLINICAL TRIALS INCLUDED IN THE META-ANALYSIS – RE-LY, ROCKET AF, ENGAGE AF TIMI 48, ARISTOTLE 14

TABLE S1D. CHARACTERISTICS OF PATIENTS IN CLINICAL TRIALS INCLUDED IN THE META-ANALYSIS – PIONEER-AF PCI, RE-DUAL PCI, AUGUSTUS, ENTRUST-AF-PCI 17

TABLE S2. THE RISK OF BIAS OF INDIVIDUAL STUDIES BY COCHRANE RISK ASSESSMENT TOOL. 20

FIGURE S2. THE META-ANALYSIS RESULTS FOR ALL-CAUSE MORTALITY. 22

FIGURE S2A. THE META-ANALYSIS RESULTS FOR ALL-CAUSE MORTALITY AFTER COMBINING STUDY DATA WITH RESPECT TO DOSES 23

FIGURE S3. THE FUNNEL PLOT FOR ALL-CAUSE MORTALITY. 24

FIGURE S4. THE META-ANALYSIS RESULTS FOR ALL-CAUSE MORTALITY AFTER EXCLUDING ENGAGE AF-TIMI EDOXABAN 30 MG. 25

FIGURE S4A. THE META-ANALYSIS RESULTS FOR ALL-CAUSE MORTALITY AFTER EXCLUDING ENGAGE AF-TIMI EDOXABAN 30 MG AND COMBINING STUDY DATA WITH RESPECT TO DOSES 26

FIGURE S5. THE FUNNEL PLOT FOR ALL-CAUSE MORTALITY AFTER EXCLUDING ENGAGE AF-TIMI EDOXABAN 30 MG. 27

FIGURE S6. THE META-ANALYSIS RESULTS FOR STROKE. 28

FIGURE S6A. THE META-ANALYSIS RESULTS FOR STROKE AFTER COMBINING STUDY DATA WITH RESPECT TO DOSES. 29

FIGURE S7. THE FUNNEL PLOT FOR STROKE. 30

FIGURE S8. THE META-ANALYSIS RESULTS FOR STROKE AFTER EXCLUDING ENGAGE AF-TIMI EDOXABAN 30 MG. 31

FIGURE S8A. THE META-ANALYSIS RESULTS FOR STROKE AFTER EXCLUDING ENGAGE AF-TIMI EDOXABAN 30 MG AND COMBINING STUDY DATA WITH RESPECT TO DOSES. 32

FIGURE S9. THE FUNNEL PLOT FOR STROKE AFTER EXCLUDING ENGAGE AF-TIMI EDOXABAN 30 MG. 33

FIGURE S10. THE META-ANALYSIS RESULTS FOR MACE – REANALYSIS RE-LY [27] -INTENTION TO TREAT DATA. 34

FIGURE S10A. THE META-ANALYSIS RESULTS FOR MACE – REANALYSIS RE-LY [27] -INTENTION TO TREAT DATA AFTER COMBINING STUDY DATA WITH RESPECT TO DOSES. 35

FIGURE S11. THE FUNNEL PLOT FOR MACE – RE-ANALYSIS RE-LY [27] -INTENTION TO TREAT DATA. 36

FIGURE S12. THE META-ANALYSIS RESULTS FOR MACE – REANALYSIS RE-LY [27] -INTENTION TO TREAT DATA AFTER EXCLUDING ENGAGE AF-TIMI EDOXABAN 30 MG ...37

FIGURE S12A. THE META-ANALYSIS RESULTS FOR MACE – REANALYSIS RE-LY [27] -INTENTION TO TREAT DATA AFTER EXCLUDING ENGAGE AF-TIMI EDOXABAN 30 MG AND COMBINING STUDY DATA WITH RESPECT TO DOSES. 38

FIGURE S13. THE FUNNEL PLOT FOR MACE – REANALYSIS RE-LY [27] -INTENTION TO TREAT DATA AFTER EXCLUDING ENGAGE AF-TIMI EDOXABAN 30 MG. 39

FIGURE S14. THE META-ANALYSIS RESULTS FOR MACE – REANALYSIS RE-LY [27] -ON TREATMENT DATA. 40

FIGURE S14A. THE META-ANALYSIS RESULTS FOR MACE – REANALYSIS RE-LY [27] -ON TREATMENT DATA AFTER COMBINING STUDY DATA WITH RESPECT TO DOSES. ...41

FIGURE S15. THE FUNNEL PLOT FOR MACE – REANALYSIS RE-LY [27] - ON TREATMENT DATA. 42

FIGURE S16. THE META-ANALYSIS RESULTS FOR MACE – REANALYSIS RE-LY [27] -ON TREATMENT DATA AFTER EXCLUDING ENGAGE AF-TIMI EDOXABAN 30 MG. 43

FIGURE S16A. THE META-ANALYSIS RESULTS FOR MACE – REANALYSIS RE-LY [27] -ON TREATMENT DATA AFTER EXCLUDING ENGAGE AF-TIMI EDOXABAN 30 MG AND COMBINING STUDY DATA WITH RESPECT TO DOSES. 44

FIGURE S17. THE FUNNEL PLOT FOR MACE – REANALYSIS RE-LY [27] ON TREATMENT DATA AFTER EXCLUDING ENGAGE AF-TIMI EDOXABAN 30 MG. 45

FIGURE S18. THE META-ANALYSIS RESULTS FOR MI. 46

FIGURE S18A. THE META-ANALYSIS RESULTS FOR MI AFTER COMBINING STUDY DATA WITH RESPECT TO DOSES. 47

FIGURE S19. THE FUNNEL PLOT FOR MI. 48

FIGURE S20. THE META-ANALYSIS RESULTS FOR MI AFTER EXCLUDING ENGAGE AF-TIMI EDOXABAN 30 MG. 49

FIGURE S20A. THE META-ANALYSIS RESULTS FOR MI AFTER EXCLUDING ENGAGE AF-TIMI EDOXABAN 30 MG AND COMBINING STUDY DATA WITH RESPECT TO DOSES. 50

FIGURE S21. THE FUNNEL PLOT FOR MI AFTER EXCLUDING ENGAGE AF-TIMI EDOXABAN 30 MG. 51

FIGURE S22. THE META-ANALYSIS RESULTS FOR MI – REANALYSIS RE-LY [27] -INTENTION TO TREAT DATA. 52

FIGURE S22A. THE META-ANALYSIS RESULTS FOR MI – REANALYSIS RE-LY [27] -INTENTION TO TREAT DATA AFTER COMBINING STUDY DATA WITH RESPECT TO DOSES. 53

FIGURE S23. THE FUNNEL PLOT FOR MI – REANALYSIS RE-LY [27] -INTENTION TO TREAT DATA. 54

FIGURE S24. THE META-ANALYSIS RESULTS FOR MI – REANALYSIS RE-LY [27] - INTENTION TO TREAT DATA AFTER EXCLUDING ENGAGE AF-TIMI EDOXABAN 30 MG........55

FIGURE S24A. THE META-ANALYSIS RESULTS FOR MI – REANALYSIS RE-LY [27] - INTENTION TO TREAT DATA AFTER EXCLUDING ENGAGE AF-TIMI EDOXABAN 30 MG AND COMBINING STUDY DATA WITH RESPECT TO DOSES........56

FIGURE S25. THE FUNNEL PLOT FOR MI – REANALYSIS RE-LY [27] -INTENTION TO TREAT DATA AFTER EXCLUDING ENGAGE AF-TIMI EDOXABAN 30 MG........57

FIGURE S26. THE META-ANALYSIS RESULTS FOR MI – REANALYSIS RE-LY [27] - ON TREATMENT DATA........58

FIGURE S26A. THE META-ANALYSIS RESULTS FOR MI – REANALYSIS RE-LY [27] - ON TREATMENT DATA AFTER COMBINING STUDY DATA WITH RESPECT TO DOSES........59

FIGURE S27. THE FUNNEL PLOT FOR MI – REANALYSIS RE-LY [27] - ON TREATMENT DATA........60

FIGURE S28. THE META-ANALYSIS RESULTS FOR MI – REANALYSIS RE-LY [27] - ON TREATMENT DATA AFTER EXCLUDING ENGAGE AF-TIMI EDOXABAN 30 MG........61

FIGURE S28A. THE META-ANALYSIS RESULTS FOR MI – REANALYSIS RE-LY [27] - ON TREATMENT DATA AFTER EXCLUDING ENGAGE AF-TIMI EDOXABAN 30 MG AND COMBINING STUDY DATA WITH RESPECT TO DOSES........62

FIGURE S29. THE FUNNEL PLOT FOR MI – REANALYSIS RE-LY [27] - ON TREATMENT DATA AFTER EXCLUDING ENGAGE AF-TIMI EDOXABAN 30 MG........63

FIGURE S30. THE META-ANALYSIS RESULTS FOR STENT THROMBOSIS........64

FIGURE S30A. THE META-ANALYSIS RESULTS FOR STENT THROMBOSIS AFTER COMBINING STUDY DATA WITH RESPECT TO DOSES........65

FIGURE S31. THE FUNNEL PLOT FOR STENT THROMBOSIS........66

FIGURE S32. THE META-ANALYSIS RESULTS FOR CARDIOVASCULAR MORTALITY........67

FIGURE S32A. THE META-ANALYSIS RESULTS FOR CARDIOVASCULAR MORTALITY AFTER COMBINING STUDY DATA WITH RESPECT TO DOSES........68

FIGURE S33. THE FUNNEL PLOT FOR CARDIOVASCULAR MORTALITY........69

FIGURE S34. THE META-ANALYSIS RESULTS FOR CARDIOVASCULAR MORTALITY AFTER EXCLUDING ENGAGE AF-TIMI EDOXABAN 30 MG........70

FIGURE S34A. THE META-ANALYSIS RESULTS FOR CARDIOVASCULAR MORTALITY AFTER EXCLUDING ENGAGE AF-TIMI EDOXABAN 30 MG AND COMBINING STUDY DATA WITH RESPECT TO DOSES........71

FIGURE S35. THE FUNNEL PLOT FOR CARDIOVASCULAR MORTALITY AFTER EXCLUDING ENGAGE AF-TIMI EDOXABAN 30 MG........72

FIGURE S36. THE META-ANALYSIS RESULTS FOR CARDIOVASCULAR MORTALITY REANALYSIS RE-LY [27]– INTENTION TO TREAT DATA........73

FIGURE S36A. THE META-ANALYSIS RESULTS FOR CARDIOVASCULAR MORTALITY REANALYSIS RE-LY [27]– INTENTION TO TREAT DATA AFTER COMBINING STUDY DATA WITH RESPECT TO DOSES........74

FIGURE S37. THE FUNNEL PLOT FOR CARDIOVASCULAR MORTALITY REANALYSIS RE-LY [27] – INTENTION TO TREAT DATA. 75

FIGURE S38. THE META-ANALYSIS RESULTS FOR CARDIOVASCULAR MORTALITY REANALYSIS RE-LY [27]– INTENTION TO TREAT DATA AFTER EXCLUDING ENGAGE AF-TIMI EDOXABAN 30 MG. 76

FIGURE S38A. THE META-ANALYSIS RESULTS FOR CARDIOVASCULAR MORTALITY REANALYSIS RE-LY [27]– INTENTION TO TREAT DATA AFTER EXCLUDING ENGAGE AF-TIMI EDOXABAN 30 MG AND COMBINING STUDY DATA WITH RESPECT TO DOSES. 77

FIGURE S39. THE FUNNEL PLOT FOR CARDIOVASCULAR MORTALITY REANALYSIS RE-LY [27] – INTENTION TO TREAT DATA AFTER EXCLUDING ENGAGE AF-TIMI EDOXABAN 30 MG. 78

FIGURE S40. THE META-ANALYSIS RESULTS FOR CARDIOVASCULAR MORTALITY REANALYSIS RE-LY [27] – ON TREATMENT DATA. 79

FIGURE S40A. THE META-ANALYSIS RESULTS FOR CARDIOVASCULAR MORTALITY REANALYSIS RE-LY [27] – ON TREATMENT DATA AFTER COMBINING STUDY DATA WITH RESPECT TO DOSES. 80

FIGURE S41. THE FUNNEL PLOT FOR CARDIOVASCULAR MORTALITY - RE-ANALYSIS RE-LY [27] – ON TREATMENT DATA. 81

FIGURE S42. THE META-ANALYSIS RESULTS FOR CARDIOVASCULAR MORTALITY REANALYSIS RE-LY [27] – ON TREATMENT DATA AFTER EXCLUDING ENGAGE AF-TIMI EDOXABAN 30 MG. 82

FIGURE S42A. THE META-ANALYSIS RESULTS FOR CARDIOVASCULAR MORTALITY REANALYSIS RE-LY [27] – ON TREATMENT DATA AFTER EXCLUDING ENGAGE AF-TIMI EDOXABAN 30 MG AND COMBINING STUDY DATA WITH RESPECT TO DOSES. 83

FIGURE S43. THE FUNNEL PLOT FOR CARDIOVASCULAR MORTALITY REANALYSIS RE-LY [27] – ON TREATMENT DATA AFTER EXCLUDING ENGAGE AF-TIMI EDOXABAN 30 MG. 84

FIGURE S44. THE NETWORK PLOT OF COMPARED NOAC'S AND WARFARIN. 85

TABLE S3. DIRECT AND INDIRECT COMPARISON BETWEEN WARFARIN AND NOAC'S – MACE**– INTENTION TO TREAT DATA. 86

TABLE S4. TREATMENT HIERARCHY ASSESSED BY SURFACE UNDER THE CUMULATIVE RANKING (SUCRA) CURVES, – MACE** 87

– INTENTION TO TREAT DATA. 87

FIGURE S45. RANKOGRAMS FOR THE DRUGS NETWORK SHOWING THE PROBABILITY EVERY TREATMENT BEING AT PARTICULAR ORDER– MACE** – INTENTION TO TREAT DATA. 88

TABLE S5. DIRECT AND INDIRECT COMPARISON BETWEEN WARFARIN AND NOAC'S – MACE** – ON TREATMENT DATA. 89

TABLE S6. TREATMENT HIERARCHY ASSESSED BY SURFACE UNDER THE CUMULATIVE RANKING (SUCRA) CURVES, – MACE** ON TREATMENT DATA. 90

FIGURE S46. RANKOGRAMS FOR THE DRUGS NETWORK SHOWING THE PROBABILITY EVERY TREATMENT BEING AT PARTICULAR ORDER– MACE**– ON TREATMENT DATA. 91

TABLE S7. DIRECT AND INDIRECT COMPARISON BETWEEN WARFARIN AND NOAC'S – MI* DATA. 92

TABLE S8. TREATMENT HIERARCHY ASSESSED BY SURFACE UNDER THE CUMULATIVE RANKING (SUCRA) CURVES, – MI* 93

DATA. 93

FIGURE S47. RANKOGRAMS FOR THE DRUGS NETWORK SHOWING THE PROBABILITY EVERY TREATMENT BEING AT PARTICULAR ORDER– MI* DATA. 94

TABLE S9. DIRECT AND INDIRECT COMPARISON BETWEEN WARFARIN AND NOAC'S – MI**- INTENTION TO TREAT DATA. 95

TABLE S10. TREATMENT HIERARCHY ASSESSED BY SURFACE UNDER THE CUMULATIVE RANKING (SUCRA) CURVES, – MI** - INTENTION TO TREAT DATA. 96

FIGURE S48. RANKOGRAMS FOR THE DRUGS NETWORK SHOWING THE PROBABILITY EVERY TREATMENT BEING AT PARTICULAR ORDER– MI** - INTENTION TO TREAT DATA. 97

TABLE S11. DIRECT AND INDIRECT COMPARISON BETWEEN WARFARIN AND NOAC'S – MI** – ON TREATMENT DATA. 98

TABLE S12. TREATMENT HIERARCHY ASSESSED BY SURFACE UNDER THE CUMULATIVE RANKING (SUCRA) CURVES, – MI** 99

– ON TREATMENT DATA. 99

FIGURE S49. RANKOGRAMS FOR THE DRUGS NETWORK SHOWING THE PROBABILITY EVERY TREATMENT BEING AT PARTICULAR ORDER– MI**– ON TREATMENT DATA. 100

TABLE S13. DIRECT AND INDIRECT COMPARISON BETWEEN WARFARIN AND NOAC'S – STROKE DATA. 101

TABLE S14. TREATMENT HIERARCHY ASSESSED BY SURFACE UNDER THE CUMULATIVE RANKING (SUCRA) CURVES, – STROKE 102

DATA. 102

FIGURE S50. RANKOGRAMS FOR THE DRUGS NETWORK SHOWING THE PROBABILITY EVERY TREATMENT BEING AT PARTICULAR ORDER - STROKE DATA. 103

TABLE S15. DIRECT AND INDIRECT COMPARISON BETWEEN WARFARIN AND NOAC'S – OVERALL MORTALITY DATA. 104

TABLE S16. TREATMENT HIERARCHY ASSESSED BY SURFACE UNDER THE CUMULATIVE RANKING (SUCRA) CURVES, – OVERALL MORTALITY DATA. 105

FIGURE S51. RANKOGRAMS FOR THE DRUGS NETWORK SHOWING THE PROBABILITY EVERY TREATMENT BEING AT PARTICULAR ORDER – OVERALL MORTALITY DATA. 106

Figure S1. Flowchart of literature search.

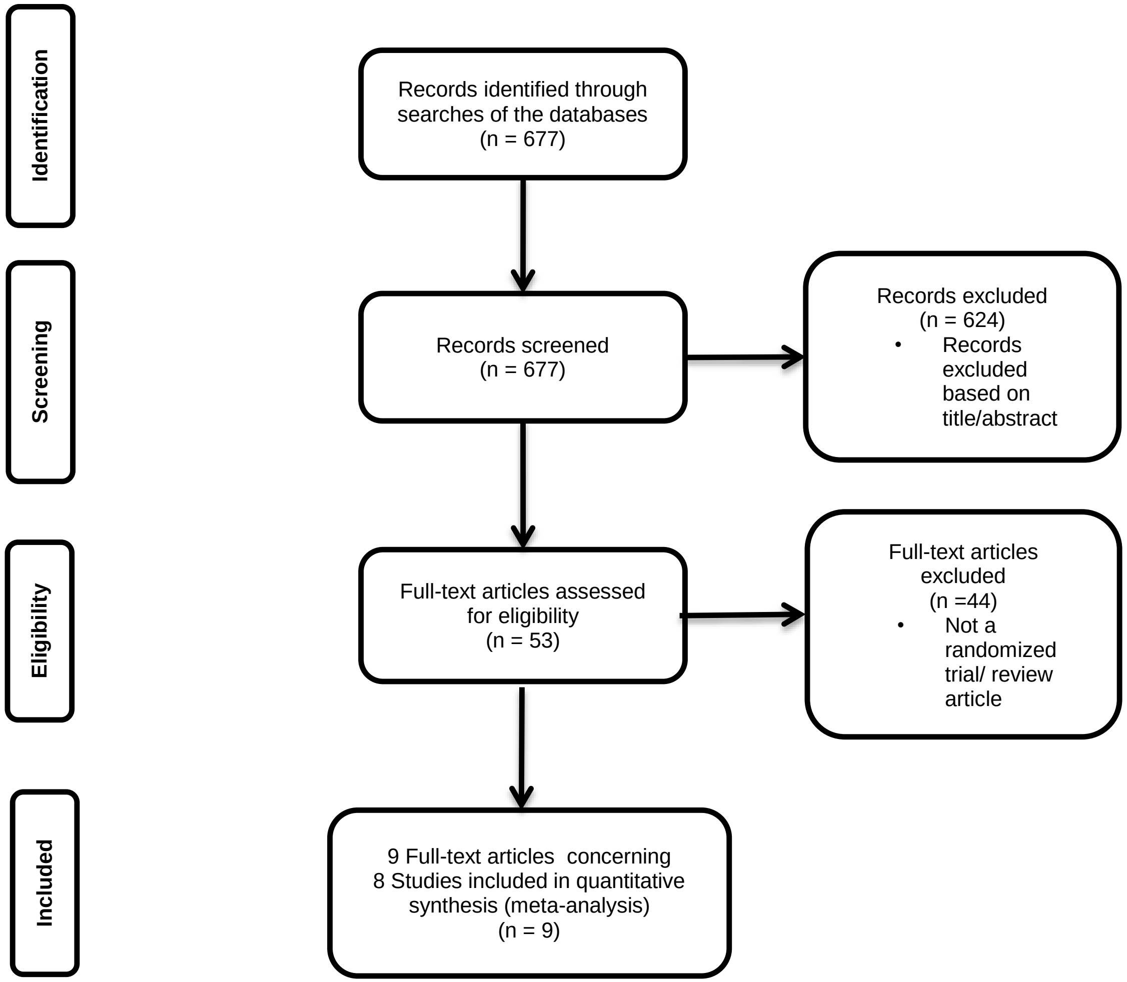

Detailed statistical methods: data synthesis and analysis

The methodological quality of randomized studies was assessed using the Cochrane Collaboration tool for assessing risk of bias. For each clinical trial, bias was assessed qualitatively as low, unclear or high (Table S2). The assessment was made independently by two authors (SG and MM). A meta-analysis comparing the results of individual NOACs vs. warfarin was performed using a random model, which considered between-study variance-tau-squared. Random effects models are more conservative, leading the estimates with wider confidence intervals. In case the τ^2 were zero, the pooled estimate of the random model would correspond to those from the fixed-effect model. As a measure of the effect, the Mantel-Haenszel relative risk (RR) with 95% confidence interval was used. A sensitivity analysis was performed by excluding the results of 30mg edoxaban from the ENGAGE AF-TIMI study [26].

Furthermore, in case we took under consideration three arms studies (two different doses and control group), the analysis was performed twice: first, performing the analysis separately to different dosages, which required doubling the events and sample size of the control group. Due to this approach, we could get an estimate of a particular dose. In the second approach, we combined the results of different dosages into one group vs. control. This approach maintained a real number of events in the control group, but as a result, the obtained estimate reflects artificial dosage (non-existing one). Both results are presented in the supplementary material. If results remain consistent across the different models then, the results can be considered robust. Additionally, a difference between the effects obtained from drug's classes comparison - Direct Thrombin Inhibitors (DTI) vs warfarin and factor Xa inhibitors (FXa inhibitors) vs warfarin - was analyzed. The calculations were performed using Review Manager (RevMan 5.3 Cochrane Community. Copenhagen: The Nordic Cochrane Centre, The Cochrane Collaboration, 2014).

For comparisons between individual drugs as well as each of them with warfarin, we used a network meta-analysis (network plot) (Fig.S44) We analyzed endpoints for which at least two direct studies of the particular drug vs. warfarin were available. Therefore, network analysis was not used for edoxaban 30mg and for risk assessment of CVD and ST. The RR values for MI are presented in Fig.5. Calculations were performed three times; based on data from the original RELY study [23] and after data correction in the intention-to-treat and on-treatment analysis [27].

Indirect analysis of the 'star' type network was performed using the Busher's method. Network meta-analysis was performed with mvmet command (STATA). We then estimated the relative probability of ranking each therapy and obtained a hierarchy of competing treatments using SUCRA (surface under the cumulative ranking) with the method proposed by G. Salanti [31], which requires estimation of the probability of being the best for a particular therapy. The probability is estimated based on the Bayesian model. We assume uniform distribution as a priori distribution. As a result, we get a posterior normal distribution with mean and variance, where estimators of normal distribution parameters are estimated based on frequentist inference.

The SUCRA (Surface Under the Cumulative Ranking) score is a metric to evaluate which treatment in a network is likely to be the most efficacious in the context of network meta-analyses. The SUCRA score is calculated in the function using the formula described by G. Salanti et al. [31]:

$$SUCRA_j = \frac{\sum_{b=1}^{a-1} cum_{jb}}{a-1}$$

Where j is some treatment, a are all competing treatments, b are the b=1,2,...,a−1 best treatments, and cum represents the cumulative probability of a treatment being among the b best treatments.

Calculations were made with STATA 15.1 software (StataCorp LLC, College Station, Texas, United States).

Table S1a. The characteristics of studies included in meta-analysis.

Study	RE-LY, 2009 [23] n=18113	ROCKET AF, 2011, [24] n=14264	ENGAGE AF-TIMI 48, 2013 [26] n=21105	ARISTOTLE, 2011 [25] n=18201	PIONEERAF-PCI, 2016 [3] n=2124	RE-DUAL PCI, 2017 [4] n=2725	AUGUSTUS, 2019 [5] n=4614	ENTRUST-AF - PCI, 2019 [6] n=1506
Inclusion criteria	AF and one of: previous stroke or transient	AF and history of stroke, TIA, or systemic	AF and CHADS2 >2, and anticoagulation	AF and one of : age of at least 75 years; previous stroke, TIA,	AF and PCI	AF and successful PCI	AF and planned use of OAC ; recent ACS or PCI; and planned use	AF and PCI for ACS or SCC

	ischemic attack, LVEF < 40%, NYHA II or higher, HF symptoms within 6 months before screening, and an age of at least 75 years or an age of 65 to 74 years plus DM, HA, or CAD	embolism or at least two of the following risk factors: HF or LVEF of 35% or less, HA, an age of 75 years or more, or the presence of DM	therapy planned for the duration of the trial	or systemic embolism; symptomatic heart failure within the previous 3 months or LVEF < 40%; DM or HA		within the previous 120 hours; ACS or SCC	of a P2Y12 inhibitor for at least 6 months	
Exclusion criteria	severe heart-valve disorder, stroke within 14 days or severe stroke within 6 months before screening, increased the risk of		reversible cause of AF; severe renal insufficiency; a high risk of bleeding; use of dual antiplatelet therapy; moderate-to severe	reversible cause of AF, moderate or severe mitral stenosis, conditions other than atrial fibrillation that required anticoagulation, stroke	history of stroke or transient ischemic attack, gastrointestinal bleeding , severe renal insufficiency, anemia, increased	bioprosthetic or mechanical heart valves, severe renal insufficiency or other major coexisting conditions.	OAC for other indication, severe renal insufficiency, intracranial hemorrhage, CABG, coagulopathy, bleeding, contraindication for study therapy	

	hemorrhage, severe renal insufficiency, active liver disease, and pregnancy.		mitral stenosis; other indications for anticoagulation therapy; ACS, coronary revascularization, or stroke within 30 days before randomization; and an inability to adhere to study procedures.	within the previous 7 days, a need for aspirin at a dose of >165 mg a day or for both aspirin and clopidogrel, and severe renal insufficiency	risk of bleeding			
Mean follow up	2 years	707 days	2.8 years	1.8 years	3 years	14 months	7 months	12 months
Primary Outcomes	Stroke or systemic embolism	Stroke and systemic embolism	Stroke or systemic embolism	Stroke or systemic embolism	Clinically significant bleeding	Major or clinically relevant bleeding	Major or clinically relevant bleeding	Major or clinically relevant bleeding,
Secondary Outcomes	Stroke, systemic embolism and death	Stroke, systemic embolism, death from	Stroke, systemic embolism, or death from cardiovascular	Death from any cause, MI	Bleeding, death from cardiovascular causes, MI, stroke, stent thrombosis	MI, stroke, systemic embolism, death, unplanned revascularization	Death, hospitalization, stroke, MI, sent thrombosis, urgent revascularization	Efficacy – cardiovascular death, stroke, systemic embolic

		cardiovascular causes, MI	causes, MI, death from any cause					event, MI definite stent thrombosis

*From Hohnloser SH et al. [27]

[a] From Connolly SJ et al. [23] and Hohnloser SH et al. [27]

AF – atrial fibrillation, ACS – acute coronary syndrome, CABG – coronary artery bypass grafting, CAD -coronary artery disease, DM – diabetes mellitus, HA – arterial hypertension, HF – heart failure, LVEF – left ventricle ejection fraction, MI – myocardial infarction, NYHA – New York Heart Association classification, OAC – oral anticoagulants, PCI – percutaneous angioplasty, SCC – stable coronary syndrome, TIA – transient ischaemic attack,

Table S1b . MACE (++) and thromboembolic or ischemic end points (+)

Study / Endpoint	RE-LY	ROCKET AF	ENGAGE AF TIMI 48	ARISTOTLE	PIONEER-AF PCI	RE-DUAL PCI	AUGUSTUS	ENTRUST-AF -PCI
Overall mortality	+	+	+	++	++	++	++	+
Cardiovascular mortality	+	++	++		+		+	++
Cardiac death	++*							
Cardiac arrest	++*							
Myocardial infarction	++ (a)	++	++	++	++	++	++	++
Unstable angina	++*							
Stroke	++	++	++	++	++	++	++	++
Stent thrombosis					+	+	+	++

Systemic embolism	++*	++	++	++		++		++
Revascularisation	++*					++	+	

*From Hohnloser SH et al. [27]

[a] From Connolly SJ et al. [23] and Hohnloser SH et al. [27]

Table S1c. Characteristics of patients in clinical trials included in the meta-analysis – RE-LY, ROCKET AF, ENGAGE AF TIMI 48, ARISTOTLE

Characteristics	RE-LY			ROCKET AF		ENGAGE AF TIMI 48			ARISTOTLE	
Treatment/dose	Dabigatran 110 mg (n =6015)	Dabigatran 150 mg (n =6076)	Warfarin (n=6022)	Rivaroxaban 20 mg or 15 mg daily if CrCl 30-49 ml/min (n =7131)	Warfarin (n =7133)	High Dose Edoxaban (n =7035)	Low Dose Edoxaban (n =7034)	Warfarin (n =7036)	Apixaban 5mg twice daily or 2.5mg twice daily (n=9120)	Warfarin (n =9081)
Age, y, mean (SD) or median	Mean (SD) 71.4 (8.6)	Mean (SD) 71.5 (8.8)	Mean (SD) 71.6 (8.6)	Median 73	Median 73	Median 72	Median 72	Median 72	Median 70	Median 70
Female, n (%)	2150 (35.7)	2236 (36.8)	2213 (36.7)	2831 (39.7)	2832 (39.7)	2669 (37.9)	2730 (38.8)	2641 (37.5)	3234 (35.5)	3182 (35.0)

Renal function, n (%) or as indicated otherwise	NA	NA	NA	Median CrCl 67 ml/min	Median CrCl 67 ml/min	CrCl ≤50 ml/min 1379 (19.6)	CrCl ≤50 ml/min 1334 (19.0)	CrCl ≤50 ml/min 1361 (19.3)	CrCl ≤50 ml/min 1502 (16.5)	CrCl ≤50 ml/min 1515 (16.7)
CHA_2DS_2VASc, mean (SD)	2.1 (1.1)	2.2 (1.2)	2.1 (1.1)	3.48 (0.94)	3.46 (0.95)	2.8 (1.0)	2.8 (1.0)	2.8 (1.0)	2.1 (1.1)	2.1 (1.1)
HAS-BLED, mean (SD) or <3 vs ≥3	NA	NA	NA	NA	NA	NA	NA	NA	NA	NA
Hypertension, n (%)	4738 (78.8)	4795 (78.9)	4750 (78.9)	6436 (90.3)	6474 (90.8)	6591 (93.7)	6575 (93.5)	6588 (93.6)	7962 (87.3)	7954 (87.6)
Diabetes mellitus, n (%)	1409 (23.4)	1402 (23.1)	1410 (23.4)	2878 (40.4)	2817 (39.5)	2559 (36.4)	2544 (36.2)	2521 (35.8)	2284 (25.0)	2263 (24.9)
History of stroke or TIA, n	1195 (19.9)	1233 (20.3)	1195 (19.8)	3916 (54.9)	3895 (54.6)	1976 (28.1)	2006 (28.5)	1991 (28.3)	1748 (19.2)	1790 (19.7)

(%) or systemic embolism										
History of myocardial infarction, n (%)	1008 (16.8)	1029 (16.9)	968 (16.1)	1182 (16.6)	1286 (18.0)	NA	NA	NA	1319 (14.5)	1266 (13.9)
History of CABG, n (%)	NA	NA	NA	NA	NA	NA	NA	NA	NA	NA
History of PCI, n (%)	NA	NA	NA	NA	NA	NA	NA	NA	NA	NA
ASA	2404 (40.0)	2352 (38.7)	2442 (40.6)	2586 (36.3)	2619 (36.7)	2070 (29.4)	2018 (28.7)	2092 (29.7)	2859 (31.3)	2773 (30.5)

ASA – aspirin, CABG – coronary artery bypass grafting, CrCl – creatinine clearence, PCI – percutaneous angioplasty, TIA – transient ischemic attack,

Table S1d. Characteristics of patients in clinical trials included in the meta-analysis – PIONEER-AF PCI, RE-DUAL PCI, AUGUSTUS, ENTRUST-AF-PCI

Characteristics	PIONEER-AF PCI, 2016		RE-DUAL PCI, 2017				AUGUSTUS, 2019		ENTRUST-AF –PCI 2019	
Therapy	DT (n = 709) Rivaroxaban (15 mg) + $P2Y_{12}$ inhibitor (clopidogrel, 75 mg, or ticagrelor, 2 × 90 mg, or prasugrel, 10 mg)	TT (n = 706) VKA + ASA (75–100 mg) + $P2Y_{12}$ inhibitor (clopidogrel, 75 mg, or ticagrelor, 2 × 90 mg, or prasugrel, 10 mg)	DT (n = 981) Dabigatran (2 × 110 mg) + $P2Y_{12}$ inhibitor (clopidogrel, 75 mg, or ticagrelor, 2 × 90 mg)	DT (n = 763) Dabigatran (2 × 150 mg) + $P2Y_{12}$ inhibitor (clopidogrel, 75 mg, or ticagrelor, 2 × 90 mg)	TT (n = 981) VKA + ASA (<100 mg) + $P2Y_{12}$ inhibitor (clopidogrel, 75 mg, or ticagrelor, 2 × 90 mg)	TT (n=764) Corresponding TT VKA	DT/TT (n = 2306) Apixaban (2 × 5 mg or 2 × 2.5 mg) + $P2Y_{12}$ inhibitor + ASA, 81 mg/placebo	DT/TT (n = 2308) VKA + $P2Y_{12}$ inhibitor + ASA, 81 mg/placebo	DT (n=751) Edoxaban (60mg/ or 30 mg in specific indication) + $P2Y_{12}$ inhibitor	TT (n=755) VKA + $P2Y_{12}$ inhibitor + ASA (100mg, for 1-12 months)
Age, y, mean (SD)	70.4 (9.1)	69.9 (8.7)	71.5 (8.9)	68.6 (7.7)	71.7 (8.9)	68.8 (7.7)	70.4	70.9	69	70

Female sex, n (%)	181 (25.5)	188 (26.6)	253 (25.8)	171 (22.4)	231 (23.5)	170 (22.3)	670 (29.1)	667 (28.9)	194 (26)	192 (25)
Renal function, n (%) or as indicated otherwise	Mean (SD) CrCl, 78.3 (31.3) ml/min	Mean (SD) CrCl, 80.7 (30) ml/min	Mean (SD) CrCl, 76.3 (28.9) ml/min	Mean (SD) CrCl, 83.7 (31) ml/min	Mean (SD) CrCl, 75.4 (29.1) ml/min	Mean (SD) CrCl, 81.3 (29.6) ml/min	creat ≥ 1.5 mg/dl 173 (7.6)	creat ≥ 1.5 mg/dl 207 (9.2)	Mean 71.8 ml/min	Mean 71.7 ml/min
Type of index event, n (%)	NSTEMI, 130 (18.5) STEMI, 86 (12.3) UA, 145 (20.7)	NSTEMI, 123 (17.8) STEMI, 74 (10.7) UA, 164 (23.7)	ACS, 509 (51.9) CCS, 433 (44.1) Staged PCI 156 (15.9) Other 43 (4.4)	ACS, 391 (51.2) CCS, 320 (41.9) Staged PCI, 138 (18.1) Other, 65 (8.5)	ACS, 475 (48.4) CCS, 429 (43.7) Staged PCI, 168 (17.1) Other, 62 (6.3)	ACS 369 (48.3), CCS 339 (44.4), Staged PCI 134 (17.5), other 50 (6.5)	ACS and PCI, 873 (38) ACS - medical therapy, 547 (23.8) Elective PCI, 877 (38.2)	ACS and PCI, 841 (36.6) ACS -medical therapy, 550 (23.9) Elective PCI, 907 (39.5)	ACS 388 (52), CCS 363 (48)	ACS 389 (52), CCS 366 (48)
CHA_2DS_2VASc, mean (SD)	3.7 (1.7)	3.8 (1.6)	3.7 (1.6)	3.3 (1.5)	3.8 (1.5)	3.5 (1.5)	3.9 (1.6)	4.0 (1.6)	4	4

HAS-BLED, mean (SD) or <3 vs ≥3	<3, 28<br>≥3, 72	<3, 29<br>≥3, 71	2.7 (0.7)	2.6 (0.7)	2.8 (0.8)	2.7 (0.8)	2.9 (1.0)	2.9 (0.9)	3	3
Hypertension, n (%)	NA	NA	NA	NA	NA	NA	2042 (88.6)	2031 (88)	674 (90)	687 (91)
Diabetes mellitus, n (%)	NA	NA	362 (36.9)	260 (34.1)	371 (37.9)	303 (39.7)	842 (36.5)	836 (36.2)	259 (34)	258 (34)
Stroke or TIA, n (%)	NA	NA	74 (7.5)	52 (6.8)	100 (10.2)	77 (10.1)	326 (14.2)	307 (13.4)	97 (13)	92 (12)
History of myocardial infarction, n (%)	20%	22%	237 (24.2)	194 (25.4)	268 (27.3)	211 (27.6)	NA	NA	188 (25)	177 (23)
History of CABG, n (%)	NA	NA	97 (9.9)	79 (10.4)	111 (11.3)	87 (11.4)	NA	NA	46 (6)	49 (6)

History of PCI, n (%)	NA	NA	326 (33.2)	239 (31.3)	347 (35.4)	272 (35.6)	NA	NA	199 (26)	195 (26)

ASA – aspirin, CABG – coronary artery bypass grafting, CrCl – creatinine clearence, PCI – percutaneous angioplasty, TIA – transient ischemic attack,

Table S2. The Risk of bias of individual studies by Cochrane risk assessment tool.

Study	Random sequence generation (Selection bias)	Allocation concealment *(Selection bias)*	Blinding of participants and personnel *(Performance bias)*	Blinding of outcome assessment personnel *(Detection bias)*	Incomplete outcome data *(Attrition bias)*	Selective reporting *(Reporting bias)*	Other sources of bias
RE-LY	+	+	-	-	+	+	+
RE-DUAL PCI	+	+	-	+	+	+	+
ARISTOTLE	+	+	+	+	+	+	+
AUGUSTUS	+	+	-	+	+	+	+
ENGAGE AF-TIMI	+	+	+	+	+	+	+
ENTRUST-AF PCI	+	+	-	+	+	+	+

PIONEER PCI	+	+	−	+	+	+	+
ROCKET - AF	+	+	+	+	+	+	+

\+ **low risk of bias** ? **unclear risk of bias** − **high risk of bias**

Figure S2. The meta-analysis results for all-cause mortality.

Study or Subgroup	NOAC Events	NOAC Total	Warfarin Events	Warfarin Total	Weight	Risk Ratio M-H, Random, 95% CI
**1.1.1 DTI**						
RE-LY* dabigatran 110 mg	446	6015	487	6022	12.8%	0.92 [0.81, 1.04]
RE-LY* dabigatran 150 mg	438	6076	487	6022	12.7%	0.89 [0.79, 1.01]
RE-DUAL PCI dabigatran 110 mg	55	981	48	981	1.4%	1.15 [0.79, 1.67]
RE-DUAL PCI dabigatran 150 mg	30	763	35	764	0.9%	0.86 [0.53, 1.38]
**Subtotal (95% CI)**		**13835**		**13789**	**27.7%**	**0.91 [0.84, 0.99]**
Total events	969		1057			
Heterogeneity: Tau² = 0.00; Chi² = 1.61, df = 3 (P = 0.66); I² = 0%						
Test for overall effect: Z = 2.12 (P = 0.03)						
**1.1.2 F Xa INH**						
ARISTOTLE apixaban	603	9120	669	9081	17.3%	0.90 [0.81, 1.00]
AUGUSTUS apixaban	77	2290	74	2259	2.0%	1.03 [0.75, 1.40]
ENGAGE AF-TIMI edoxaban 30 mg	737	7034	839	7036	22.4%	0.88 [0.80, 0.96]
ENGAGE AF-TIMI edoxaban 60 mg	773	7035	839	7036	23.0%	0.92 [0.84, 1.01]
ENTRUST-AF PCI edoxaban 60 mg	46	751	37	755	1.1%	1.25 [0.82, 1.90]
PIONEER PCI rivaroxaban	20	696	22	697	0.5%	0.91 [0.50, 1.65]
ROCKET - AF rivaroxaban	208	7061	250	7082	6.0%	0.83 [0.70, 1.00]
**Subtotal (95% CI)**		**33987**		**33946**	**72.3%**	**0.90 [0.86, 0.95]**
Total events	2464		2730			
Heterogeneity: Tau² = 0.00; Chi² = 4.19, df = 6 (P = 0.65); I² = 0%						
Test for overall effect: Z = 3.91 (P < 0.0001)						
**Total (95% CI)**		**47822**		**47735**	**100.0%**	**0.90 [0.87, 0.95]**
Total events	3433		3787			
Heterogeneity: Tau² = 0.00; Chi² = 5.86, df = 10 (P = 0.83); I² = 0%						
Test for overall effect: Z = 4.44 (P < 0.00001)						
Test for subgroup differences: Chi² = 0.06, df = 1 (P = 0.80), I² = 0%						

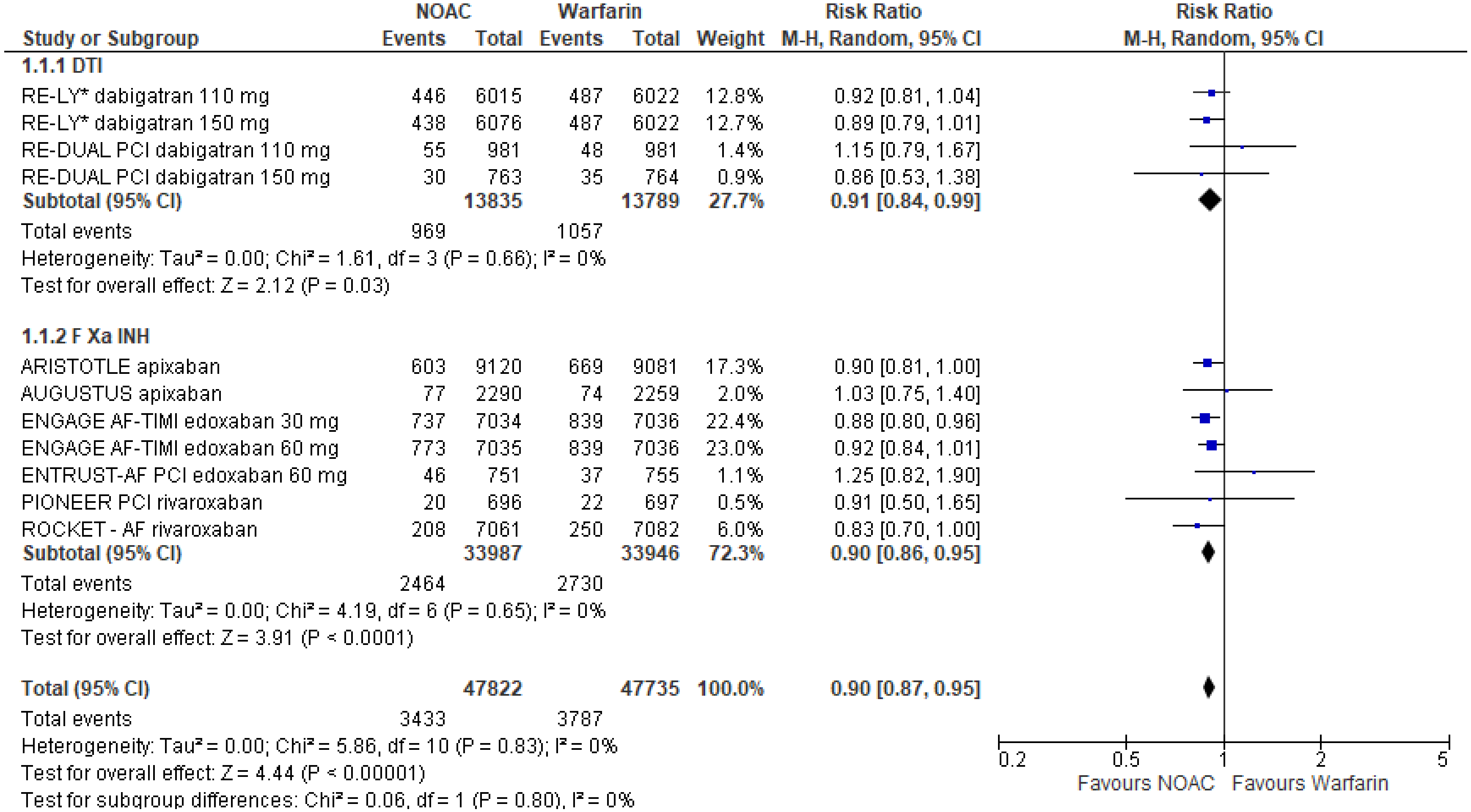

*- original data from RE-LY study [23]

Figure S2A. The meta-analysis results for all-cause mortality after combining study data with respect to doses

Study or Subgroup	NOAC Events	NOAC Total	Warfarin Events	Warfarin Total	Weight	Risk Ratio M-H, Random, 95% CI
**34.1.1 DTI**						
RE-LY* dabigatran	884	12091	487	6022	22.5%	0.90 [0.81, 1.01]
RE-DUAL PCI dabigatran	85	1764	48	981	2.1%	0.98 [0.70, 1.39]
**Subtotal (95% CI)**		**13855**		**7003**	**24.7%**	**0.91 [0.82, 1.01]**
Total events	969		535			
Heterogeneity: Tau² = 0.00; Chi² = 0.22, df = 1 (P = 0.64); I² = 0%						
Test for overall effect: Z = 1.80 (P = 0.07)						
**34.1.2 F Xa INH**						
ARISTOTLE apixaban	603	9120	669	9081	22.5%	0.90 [0.81, 1.00]
AUGUSTUS apixaban	77	2290	74	2259	2.6%	1.03 [0.75, 1.40]
ENGAGE-AF-TIMI edoxaban	1510	14069	839	7036	40.3%	0.90 [0.83, 0.97]
ENTRUST-AF PCI edoxaban 60 mg	46	751	37	755	1.4%	1.25 [0.82, 1.90]
PIONEER PCI rivaroxaban	20	696	22	697	0.7%	0.91 [0.50, 1.65]
ROCKET - AF rivaroxaban	208	7061	250	7082	7.8%	0.83 [0.70, 1.00]
**Subtotal (95% CI)**		**33987**		**26910**	**75.3%**	**0.90 [0.85, 0.96]**
Total events	2464		1891			
Heterogeneity: Tau² = 0.00; Chi² = 3.68, df = 5 (P = 0.60); I² = 0%						
Test for overall effect: Z = 3.48 (P = 0.0005)						
**Total (95% CI)**		**47842**		**33913**	**100.0%**	**0.90 [0.86, 0.95]**
Total events	3433		2426			
Heterogeneity: Tau² = 0.00; Chi² = 3.92, df = 7 (P = 0.79); I² = 0%						
Test for overall effect: Z = 3.92 (P < 0.0001)						
Test for subgroup differences: Chi² = 0.03, df = 1 (P = 0.87), I² = 0%						

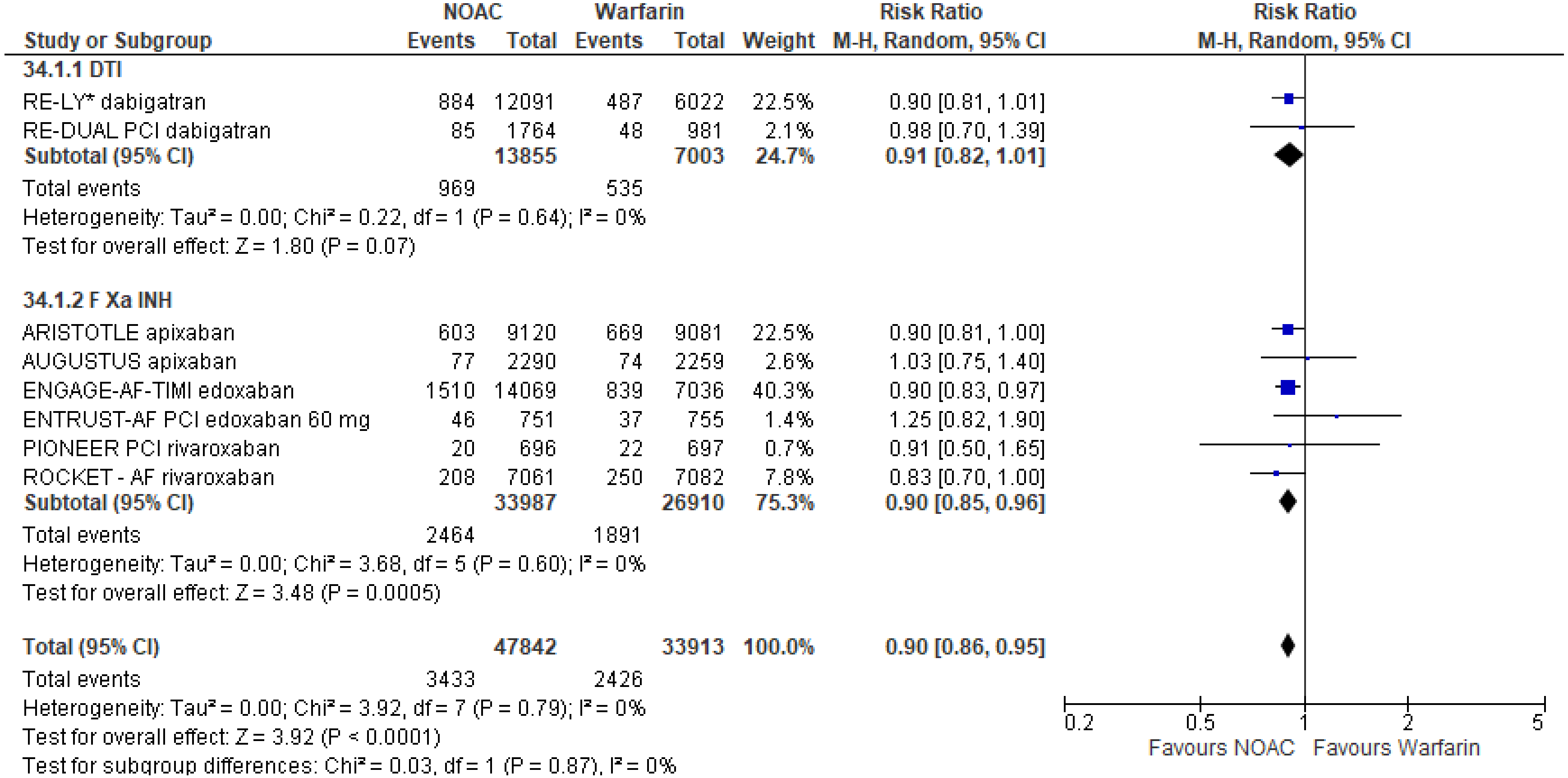

*- original data from RE-LY study [23]

Figure S3. The funnel plot for all-cause mortality.

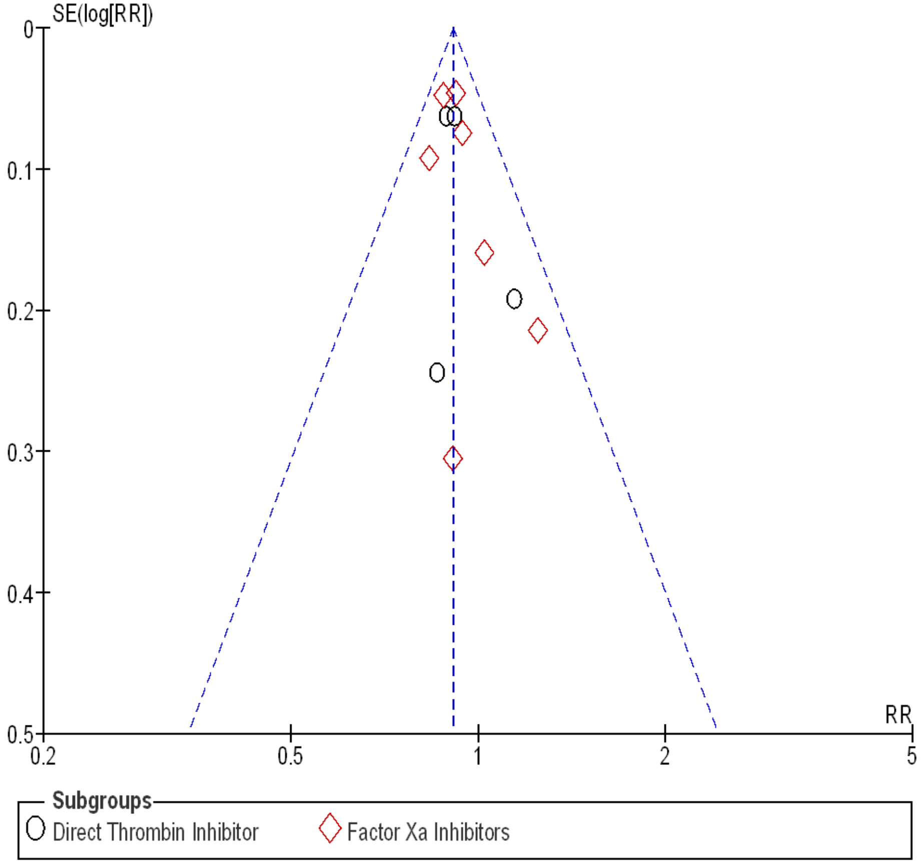

Figure S4. The meta-analysis results for all-cause mortality after excluding ENGAGE AF-TIMI edoxaban 30 mg.

Study or Subgroup	NOAC Events	NOAC Total	Warfarin Events	Warfarin Total	Weight	Risk Ratio M-H, Random, 95% CI
**2.1.1 DTI**						
RE-LY* dabigatran 110 mg	446	6015	487	6022	16.5%	0.92 [0.81, 1.04]
RE-LY* dabigatran 150 mg	438	6076	487	6022	16.3%	0.89 [0.79, 1.01]
RE-DUAL PCI dabigatran 110 mg	55	981	48	981	1.8%	1.15 [0.79, 1.67]
RE-DUAL PCI dabigatran 150 mg	30	763	35	764	1.1%	0.86 [0.53, 1.38]
**Subtotal (95% CI)**		**13835**		**13789**	**35.7%**	**0.91 [0.84, 0.99]**
Total events	969		1057			
Heterogeneity: Tau² = 0.00; Chi² = 1.61, df = 3 (P = 0.66); I² = 0%						
Test for overall effect: Z = 2.12 (P = 0.03)						
**2.1.2 F Xa INH**						
ARISTOTLE apixaban	603	9120	669	9081	22.3%	0.90 [0.81, 1.00]
AUGUSTUS apixaban	77	2290	74	2259	2.6%	1.03 [0.75, 1.40]
ENGAGE AF-TIMI edoxaban 60 mg	773	7035	839	7036	29.7%	0.92 [0.84, 1.01]
ENTRUST-AF PCI edoxaban 60 mg	46	751	37	755	1.4%	1.25 [0.82, 1.90]
PIONEER PCI rivaroxaban	20	696	22	697	0.7%	0.91 [0.50, 1.65]
ROCKET - AF rivaroxaban	208	7061	250	7082	7.7%	0.83 [0.70, 1.00]
**Subtotal (95% CI)**		**26953**		**26910**	**64.3%**	**0.91 [0.86, 0.97]**
Total events	1727		1891			
Heterogeneity: Tau² = 0.00; Chi² = 3.76, df = 5 (P = 0.58); I² = 0%						
Test for overall effect: Z = 2.88 (P = 0.004)						
**Total (95% CI)**		**40788**		**40699**	**100.0%**	**0.91 [0.87, 0.96]**
Total events	2696		2948			
Heterogeneity: Tau² = 0.00; Chi² = 5.37, df = 9 (P = 0.80); I² = 0%						
Test for overall effect: Z = 3.58 (P = 0.0003)						
Test for subgroup differences: Chi² = 0.00, df = 1 (P = 0.98), I² = 0%						

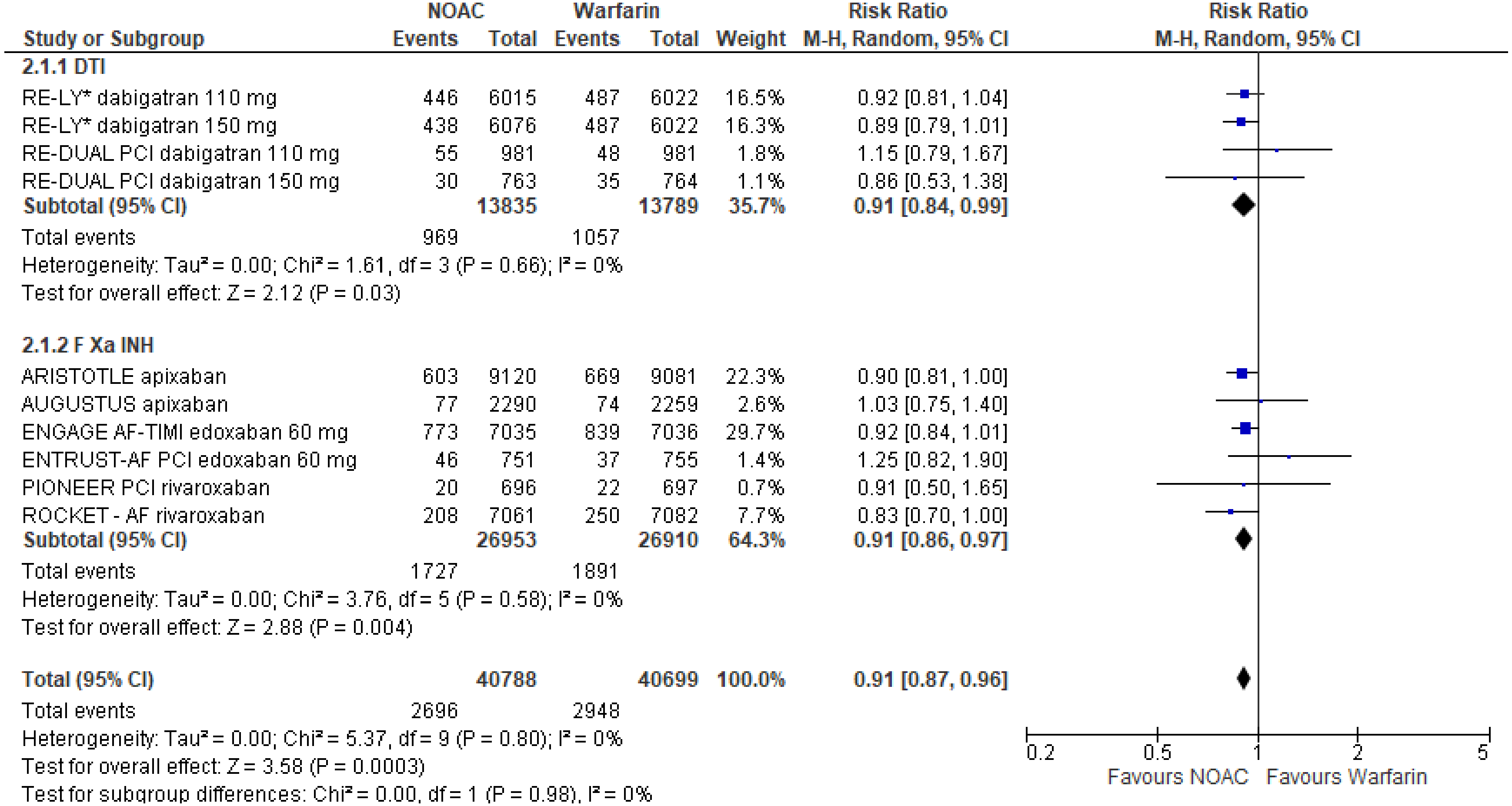

*- original data from RE-LY study [23]

Figure S4A. The meta-analysis results for all-cause mortality after excluding ENGAGE AF-TIMI edoxaban 30 mg and combining study data with respect to doses

Study or Subgroup	NOAC Events	NOAC Total	Warfarin Events	Warfarin Total	Weight	Risk Ratio M-H, Random, 95% CI
**35.1.1 DTI**						
RE-LY* dabigatran	884	12091	487	6022	25.1%	0.90 [0.81, 1.01]
RE-DUAL PCI dabigatran	85	1764	48	981	2.4%	0.98 [0.70, 1.39]
**Subtotal (95% CI)**		**13855**		**7003**	**27.5%**	**0.91 [0.82, 1.01]**
Total events	969		535			
Heterogeneity: Tau² = 0.00; Chi² = 0.22, df = 1 (P = 0.64); I² = 0%						
Test for overall effect: Z = 1.80 (P = 0.07)						
**35.1.2 F Xa INH**						
ARISTOTLE apixaban	603	9120	669	9081	25.1%	0.90 [0.81, 1.00]
AUGUSTUS apixaban	77	2290	74	2259	2.9%	1.03 [0.75, 1.40]
ENGAGE-AF-TIMI edoxaban	773	7035	839	7036	33.5%	0.92 [0.84, 1.01]
ENTRUST-AF PCI edoxaban 60 mg	46	751	37	755	1.6%	1.25 [0.82, 1.90]
PIONEER PCI rivaroxaban	20	696	22	697	0.8%	0.91 [0.50, 1.65]
ROCKET - AF rivaroxaban	208	7061	250	7082	8.6%	0.83 [0.70, 1.00]
**Subtotal (95% CI)**		**26953**		**26910**	**72.5%**	**0.91 [0.86, 0.97]**
Total events	1727		1891			
Heterogeneity: Tau² = 0.00; Chi² = 3.76, df = 5 (P = 0.58); I² = 0%						
Test for overall effect: Z = 2.88 (P = 0.004)						
**Total (95% CI)**		**40808**		**33913**	**100.0%**	**0.91 [0.86, 0.96]**
Total events	2696		2426			
Heterogeneity: Tau² = 0.00; Chi² = 3.98, df = 7 (P = 0.78); I² = 0%						
Test for overall effect: Z = 3.40 (P = 0.0007)						
Test for subgroup differences: Chi² = 0.00, df = 1 (P = 0.98), I² = 0%						

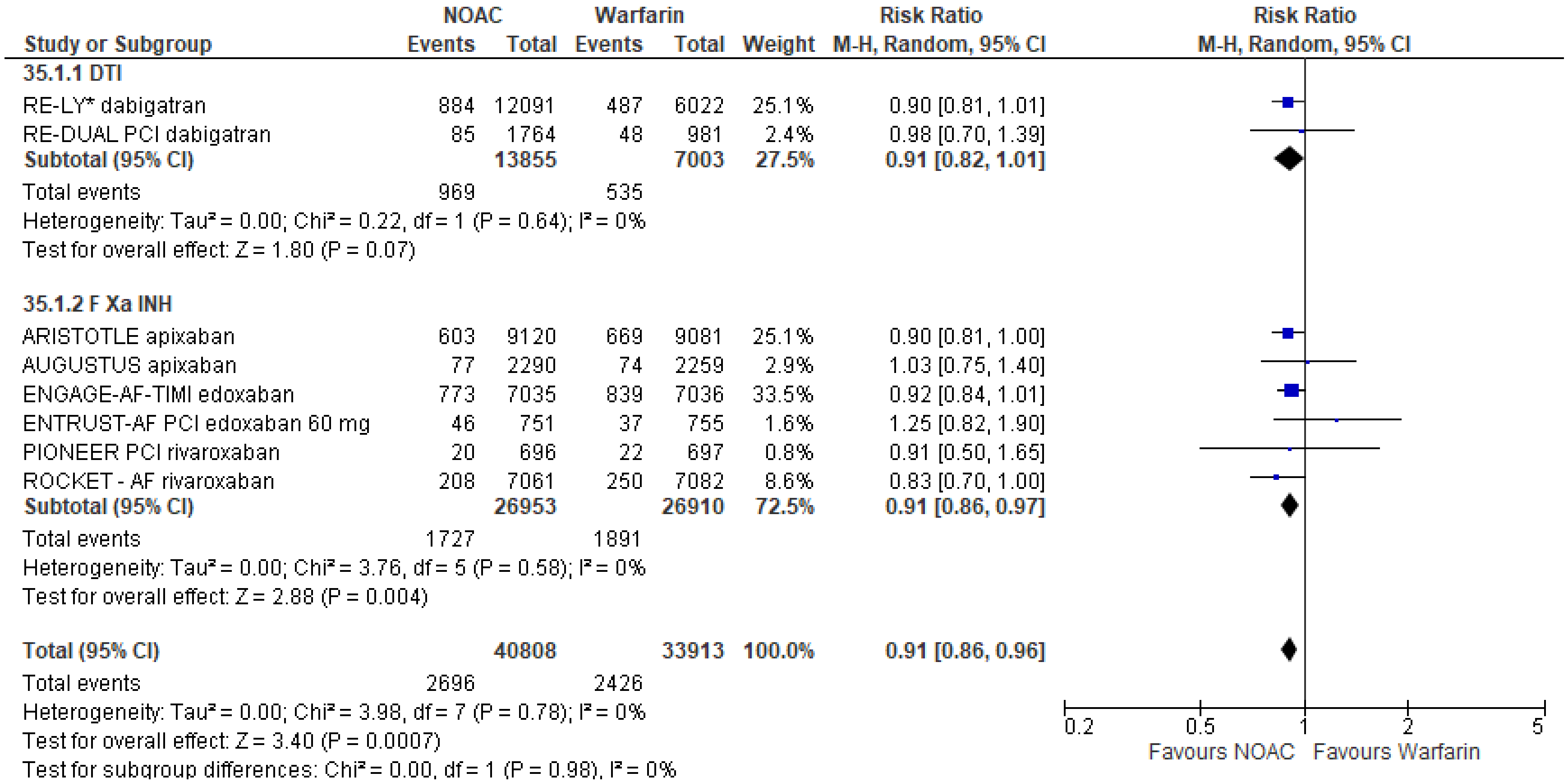

*- original data from RE-LY study [23]

Figure S5. The funnel plot for all-cause mortality after excluding ENGAGE AF-TIMI edoxaban 30 mg.

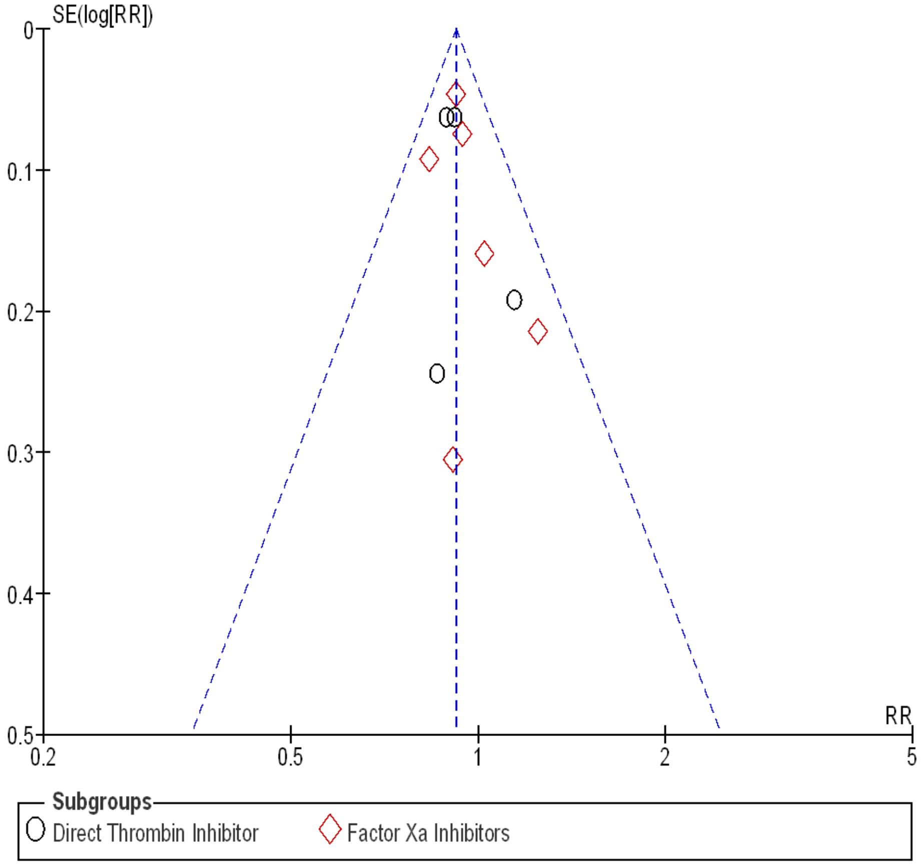

Figure S6. The meta-analysis results for stroke.

Study or Subgroup	NOAC Events	NOAC Total	Warfarin Events	Warfarin Total	Weight	Risk Ratio M-H, Random, 95% CI
**3.1.1 DTI**						
RE-LY* dabigatran 110 mg	171	6015	185	6022	13.8%	0.93 [0.75, 1.14]
RE-LY* dabigatran 150 mg	122	6076	185	6022	12.9%	0.65 [0.52, 0.82]
RE-DUAL PCI dabigatran 110 mg	17	981	13	981	3.0%	1.31 [0.64, 2.68]
RE-DUAL PCI dabigatran 150 mg	9	763	8	764	1.8%	1.13 [0.44, 2.90]
**Subtotal (95% CI)**		**13835**		**13789**	**31.5%**	**0.86 [0.64, 1.14]**
Total events	319		391			
Heterogeneity: Tau² = 0.04; Chi² = 7.27, df = 3 (P = 0.06); I² = 59%						
Test for overall effect: Z = 1.05 (P = 0.30)						
**3.1.2 F Xa INH**						
ARISTOTLE apixaban	199	9120	250	9081	14.7%	0.79 [0.66, 0.95]
AUGUSTUS apixaban	13	2290	26	2259	3.4%	0.49 [0.25, 0.96]
ENGAGE AF-TIMI edoxaban 30 mg	360	7034	317	7036	16.3%	1.14 [0.98, 1.32]
ENGAGE AF-TIMI edoxaban 60 mg	281	7035	317	7036	15.9%	0.89 [0.76, 1.04]
ENTRUST-AF PCI edoxaban 60 mg	10	751	12	755	2.3%	0.84 [0.36, 1.93]
PIONEER PCI rivaroxaban	8	694	7	695	1.6%	1.14 [0.42, 3.14]
ROCKET - AF rivaroxaban	184	7061	221	7082	14.3%	0.84 [0.69, 1.01]
**Subtotal (95% CI)**		**33985**		**33944**	**68.5%**	**0.89 [0.76, 1.04]**
Total events	1055		1150			
Heterogeneity: Tau² = 0.02; Chi² = 15.20, df = 6 (P = 0.02); I² = 61%						
Test for overall effect: Z = 1.51 (P = 0.13)						
**Total (95% CI)**		**47820**		**47733**	**100.0%**	**0.87 [0.76, 0.99]**
Total events	1374		1541			
Heterogeneity: Tau² = 0.02; Chi² = 24.45, df = 10 (P = 0.006); I² = 59%						
Test for overall effect: Z = 2.03 (P = 0.04)						
Test for subgroup differences: Chi² = 0.04, df = 1 (P = 0.84), I² = 0%						

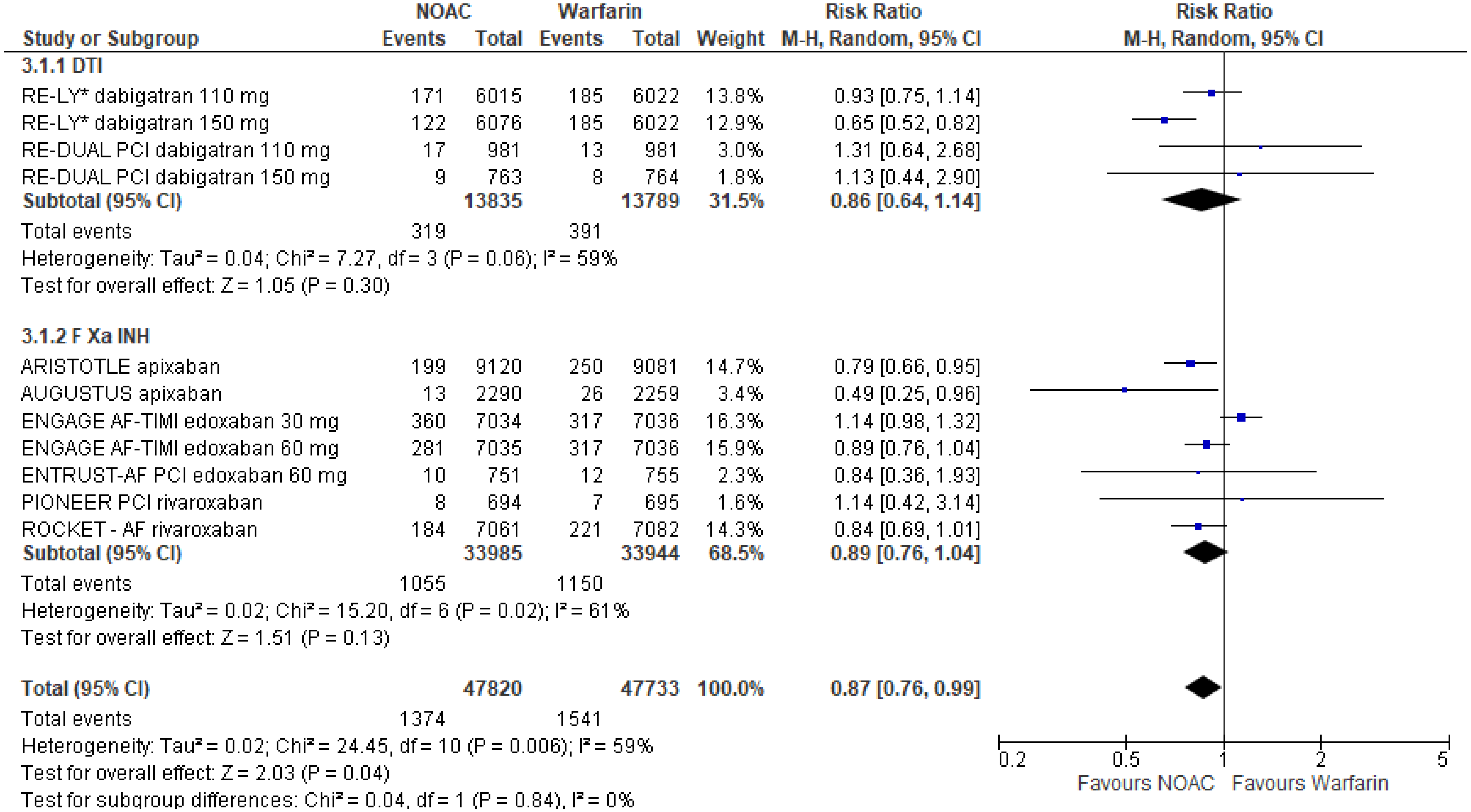

*- original data from RE-LY study [23]

Figure S6A. The meta-analysis results for stroke after combining study data with respect to doses.

Study or Subgroup	NOAC Events	NOAC Total	Warfarin Events	Warfarin Total	Weight	Risk Ratio M-H, Random, 95% CI
**36.1.1 DTI**						
RE-LY* dabigatran	293	12091	185	6022	21.4%	0.79 [0.66, 0.95]
RE-DUAL PCI dabigatran	26	1744	13	981	3.1%	1.13 [0.58, 2.18]
**Subtotal (95% CI)**		**13835**		**7003**	**24.5%**	**0.81 [0.67, 0.98]**
Total events	319		198			
Heterogeneity: Tau² = 0.00; Chi² = 1.03, df = 1 (P = 0.31); I² = 3%						
Test for overall effect: Z = 2.13 (P = 0.03)						
**36.1.2 F Xa INH**						
ARISTOTLE apixaban	199	9120	250	9081	21.1%	0.79 [0.66, 0.95]
AUGUSTUS apixaban	13	2290	26	2259	3.1%	0.49 [0.25, 0.96]
ENGAGE-AF-TIMI edoxaban	641	14069	317	7036	27.8%	1.01 [0.89, 1.15]
ENTRUST-AF PCI edoxaban 60 mg	10	751	12	755	2.0%	0.84 [0.36, 1.93]
PIONEER PCI rivaroxaban	8	694	7	695	1.4%	1.14 [0.42, 3.14]
ROCKET - AF rivaroxaban	184	7061	221	7082	20.1%	0.84 [0.69, 1.01]
**Subtotal (95% CI)**		**33985**		**26908**	**75.5%**	**0.86 [0.74, 1.01]**
Total events	1055		833			
Heterogeneity: Tau² = 0.01; Chi² = 8.84, df = 5 (P = 0.12); I² = 43%						
Test for overall effect: Z = 1.87 (P = 0.06)						
**Total (95% CI)**		**47820**		**33911**	**100.0%**	**0.86 [0.76, 0.97]**
Total events	1374		1031			
Heterogeneity: Tau² = 0.01; Chi² = 10.95, df = 7 (P = 0.14); I² = 36%						
Test for overall effect: Z = 2.48 (P = 0.01)						
Test for subgroup differences: Chi² = 0.24, df = 1 (P = 0.62), I² = 0%						

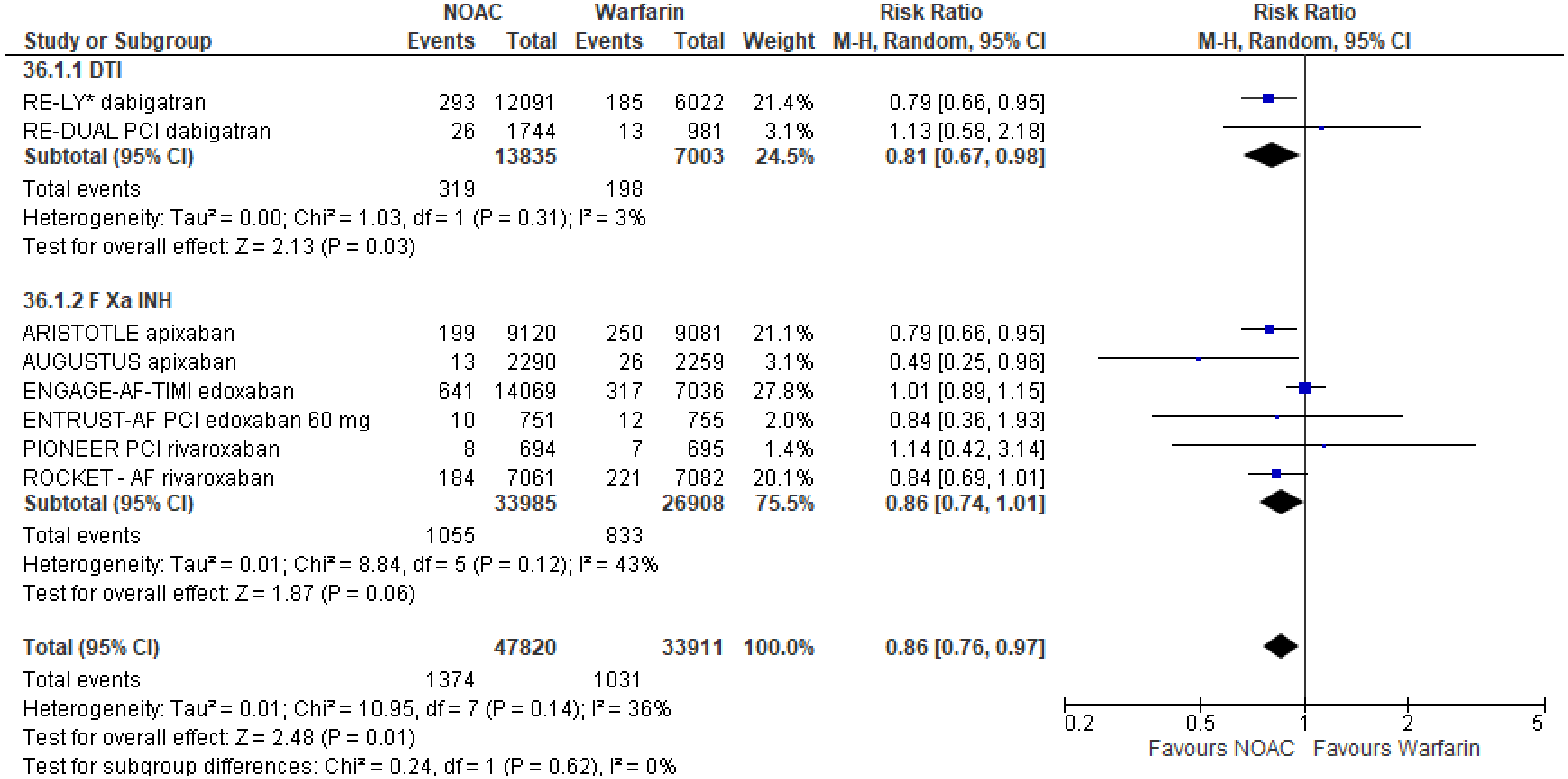

Figure S7. The funnel plot for stroke.

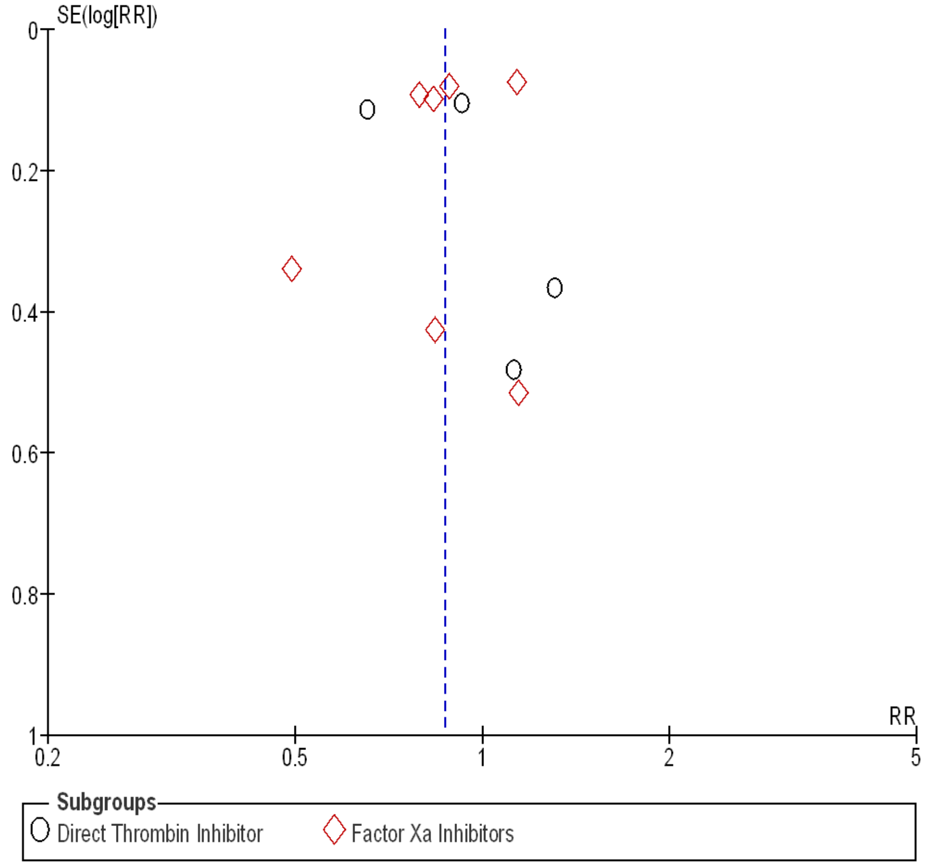

Figure S8. The meta-analysis results for stroke after excluding ENGAGE AF-TIMI edoxaban 30 mg.

	NOAC		Warfarin			Risk Ratio
**Study or Subgroup**	**Events**	**Total**	**Events**	**Total**	**Weight**	**M-H, Random, 95% CI**
**4.1.1 DTI**						
RE-LY* dabigatran 110 mg	171	6015	185	6022	16.7%	0.93 [0.75, 1.14]
RE-LY* dabigatran 150 mg	122	6076	185	6022	14.5%	0.65 [0.52, 0.82]
RE-DUAL PCI dabigatran 110 mg	17	981	13	981	1.9%	1.31 [0.64, 2.68]
RE-DUAL PCI dabigatran 150 mg	9	763	8	764	1.1%	1.13 [0.44, 2.90]
**Subtotal (95% CI)**		**13835**		**13789**	**34.2%**	**0.86 [0.64, 1.14]**
Total events	319		391			
Heterogeneity: Tau² = 0.04; Chi² = 7.27, df = 3 (P = 0.06); I² = 59%						
Test for overall effect: Z = 1.05 (P = 0.30)						
**4.1.2 F Xa INH**						
ARISTOTLE apixaban	199	9120	250	9081	19.4%	0.79 [0.66, 0.95]
AUGUSTUS apixaban	13	2290	26	2259	2.2%	0.49 [0.25, 0.96]
ENGAGE AF-TIMI edoxaban 60 mg	281	7035	317	7036	23.7%	0.89 [0.76, 1.04]
ENTRUST-AF PCI edoxaban 60 mg	10	751	12	755	1.4%	0.84 [0.36, 1.93]
PIONEER PCI rivaroxaban	8	694	7	695	0.9%	1.14 [0.42, 3.14]
ROCKET - AF rivaroxaban	184	7061	221	7082	18.2%	0.84 [0.69, 1.01]
**Subtotal (95% CI)**		**26951**		**26908**	**65.8%**	**0.84 [0.76, 0.92]**
Total events	695		833			
Heterogeneity: Tau² = 0.00; Chi² = 3.66, df = 5 (P = 0.60); I² = 0%						
Test for overall effect: Z = 3.56 (P = 0.0004)						
**Total (95% CI)**		**40786**		**40697**	**100.0%**	**0.83 [0.75, 0.91]**
Total events	1014		1224			
Heterogeneity: Tau² = 0.00; Chi² = 11.01, df = 9 (P = 0.28); I² = 18%						
Test for overall effect: Z = 3.78 (P = 0.0002)						
Test for subgroup differences: Chi² = 0.03, df = 1 (P = 0.87), I² = 0%						

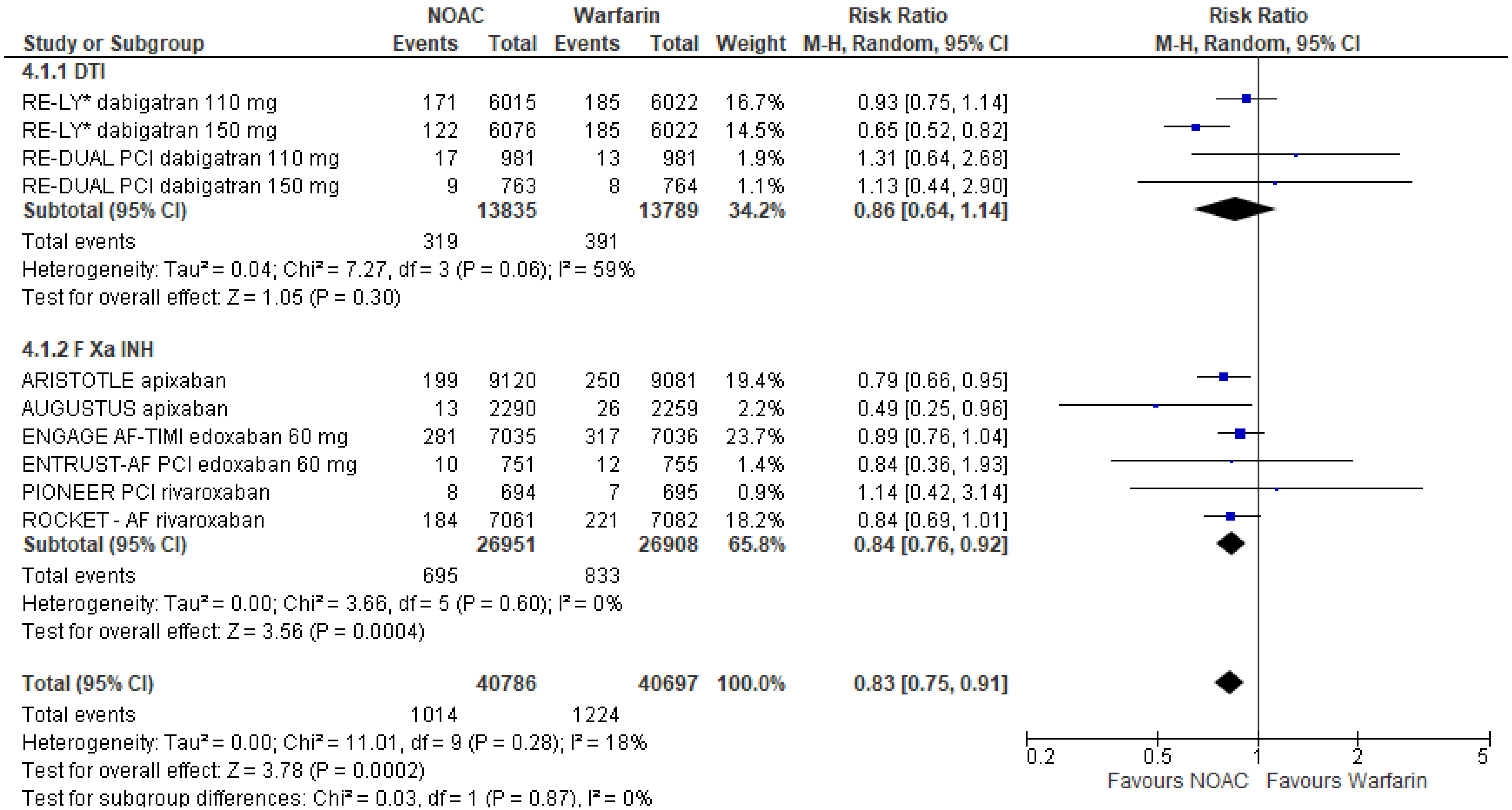

*- original data from RE-LY study [23]

Figure S8A. The meta-analysis results for stroke after excluding ENGAGE AF-TIMI edoxaban 30 mg and combining study data with respect to doses.

Study or Subgroup	NOAC Events	NOAC Total	Warfarin Events	Warfarin Total	Weight	Risk Ratio M-H, Random, 95% CI
**37.1.1 DTI**						
RE-LY* dabigatran	293	12091	185	6022	22.6%	0.79 [0.66, 0.95]
RE-DUAL PCI dabigatran	26	1744	13	981	1.7%	1.13 [0.58, 2.18]
**Subtotal (95% CI)**		**13835**		**7003**	**24.3%**	**0.81 [0.67, 0.98]**
Total events	319		198			
Heterogeneity: Tau² = 0.00; Chi² = 1.03, df = 1 (P = 0.31); I² = 3%						
Test for overall effect: Z = 2.13 (P = 0.03)						
**37.1.2 F Xa INH**						
ARISTOTLE apixaban	199	9120	250	9081	22.0%	0.79 [0.66, 0.95]
AUGUSTUS apixaban	13	2290	26	2259	1.7%	0.49 [0.25, 0.96]
ENGAGE-AF-TIMI edoxaban	281	7035	317	7036	30.1%	0.89 [0.76, 1.04]
ENTRUST-AF PCI edoxaban 60 mg	10	751	12	755	1.1%	0.84 [0.36, 1.93]
PIONEER PCI rivaroxaban	8	694	7	695	0.7%	1.14 [0.42, 3.14]
ROCKET - AF rivaroxaban	184	7061	221	7082	20.0%	0.84 [0.69, 1.01]
**Subtotal (95% CI)**		**26951**		**26908**	**75.7%**	**0.84 [0.76, 0.92]**
Total events	695		833			
Heterogeneity: Tau² = 0.00; Chi² = 3.66, df = 5 (P = 0.60); I² = 0%						
Test for overall effect: Z = 3.56 (P = 0.0004)						
**Total (95% CI)**		**40786**		**33911**	**100.0%**	**0.83 [0.76, 0.90]**
Total events	1014		1031			
Heterogeneity: Tau² = 0.00; Chi² = 4.79, df = 7 (P = 0.69); I² = 0%						
Test for overall effect: Z = 4.27 (P < 0.0001)						
Test for subgroup differences: Chi² = 0.06, df = 1 (P = 0.80), I² = 0%						

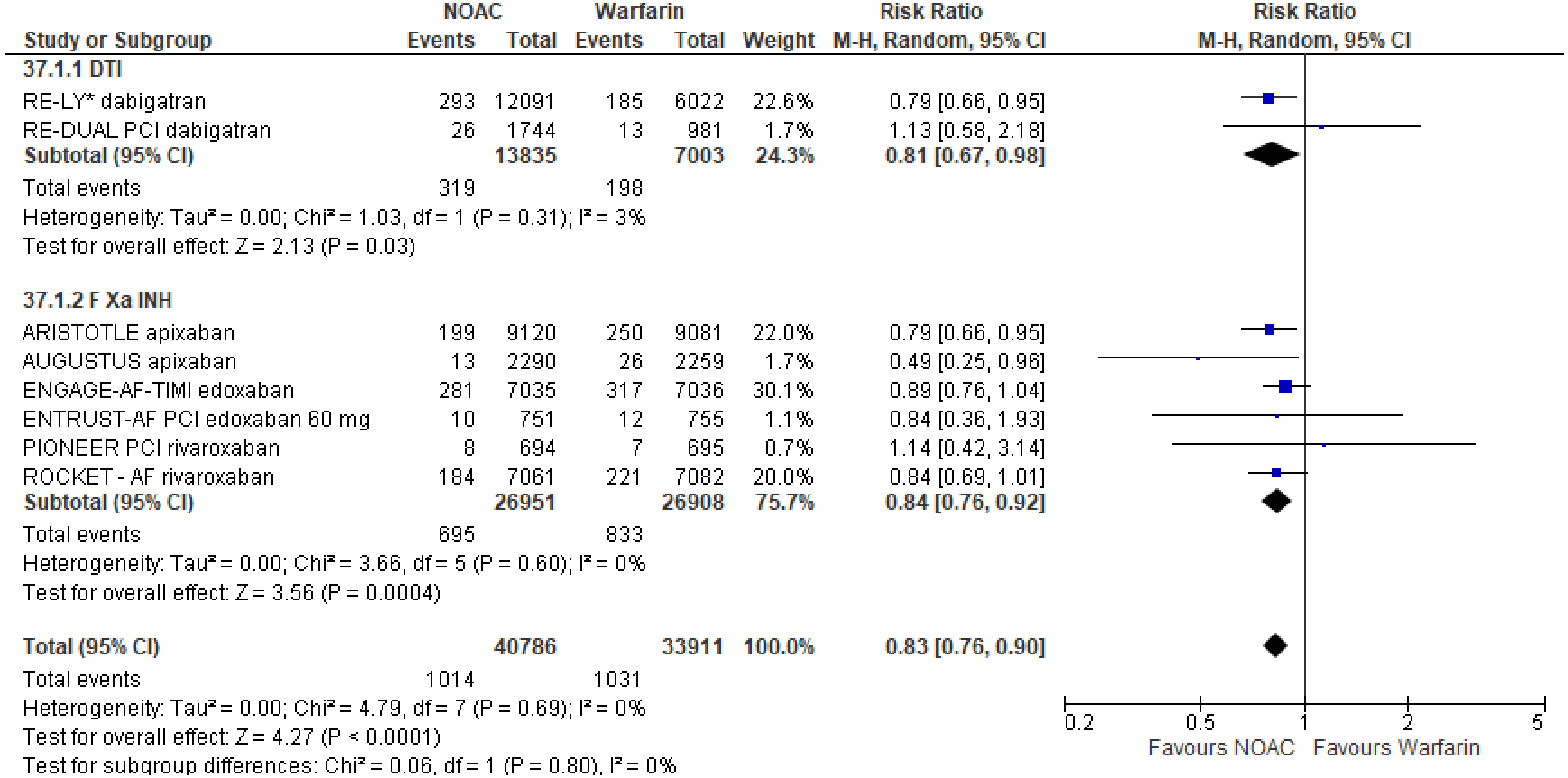

*- original data from RE-LY study [23]

Figure S9. The funnel plot for stroke after excluding ENGAGE AF-TIMI edoxaban 30 mg.

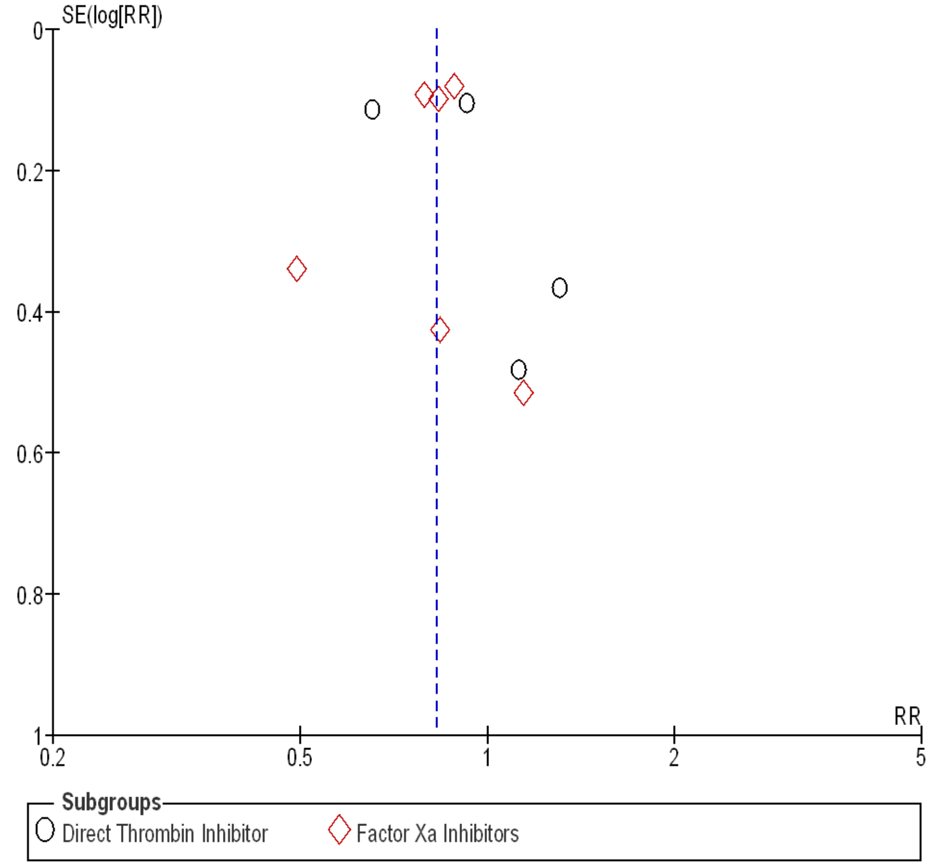

Figure S10. The meta-analysis results for MACE – reanalysis RE-LY [27] -intention to treat data.

Study or Subgroup	NOAC Events	NOAC Total	Warfarin Events	Warfarin Total	Weight	Risk Ratio M-H, Random, 95% CI
**5.1.1 DTI**						
RE-LY** dabigatran 110 mg ITT	567	6015	601	6022	12.6%	0.94 [0.85, 1.05]
RE-LY** dabigatran 150 mg ITT	538	6076	601	6022	12.2%	0.89 [0.79, 0.99]
RE-DUAL PCI dabigatran 110 mg	149	981	131	981	3.7%	1.14 [0.92, 1.41]
RE-DUAL PCI dabigatran 150 mg	90	763	98	764	2.5%	0.92 [0.70, 1.20]
**Subtotal (95% CI)**		**13835**		**13789**	**31.0%**	**0.94 [0.86, 1.03]**
Total events	1344		1431			
Heterogeneity: Tau² = 0.00; Chi² = 4.03, df = 3 (P = 0.26); I² = 25%						
Test for overall effect: Z = 1.29 (P = 0.20)						
**5.1.2 F Xa INH**						
ARISTOTLE apixaban	810	9120	906	9081	16.9%	0.89 [0.81, 0.97]
AUGUSTUS apixaban	154	2306	163	2308	3.8%	0.95 [0.76, 1.17]
ENGAGE AF-TIMI edoxaban 30 mg	913	7034	926	7036	18.3%	0.99 [0.91, 1.07]
ENGAGE AF-TIMI edoxaban 60 mg	827	7035	926	7036	17.6%	0.89 [0.82, 0.98]
ENTRUST-AF PCI edoxaban 60 mg	49	751	46	755	1.2%	1.07 [0.73, 1.58]
PIONEER PCI rivaroxaban	41	694	36	695	1.0%	1.14 [0.74, 1.76]
ROCKET - AF rivaroxaban	433	7061	519	7082	10.3%	0.84 [0.74, 0.95]
**Subtotal (95% CI)**		**34001**		**33993**	**69.0%**	**0.92 [0.87, 0.97]**
Total events	3227		3522			
Heterogeneity: Tau² = 0.00; Chi² = 7.34, df = 6 (P = 0.29); I² = 18%						
Test for overall effect: Z = 3.22 (P = 0.001)						
**Total (95% CI)**		**47836**		**47782**	**100.0%**	**0.92 [0.88, 0.96]**
Total events	4571		4953			
Heterogeneity: Tau² = 0.00; Chi² = 11.64, df = 10 (P = 0.31); I² = 14%						
Test for overall effect: Z = 3.63 (P = 0.0003)						
Test for subgroup differences: Chi² = 0.33, df = 1 (P = 0.57), I² = 0%						

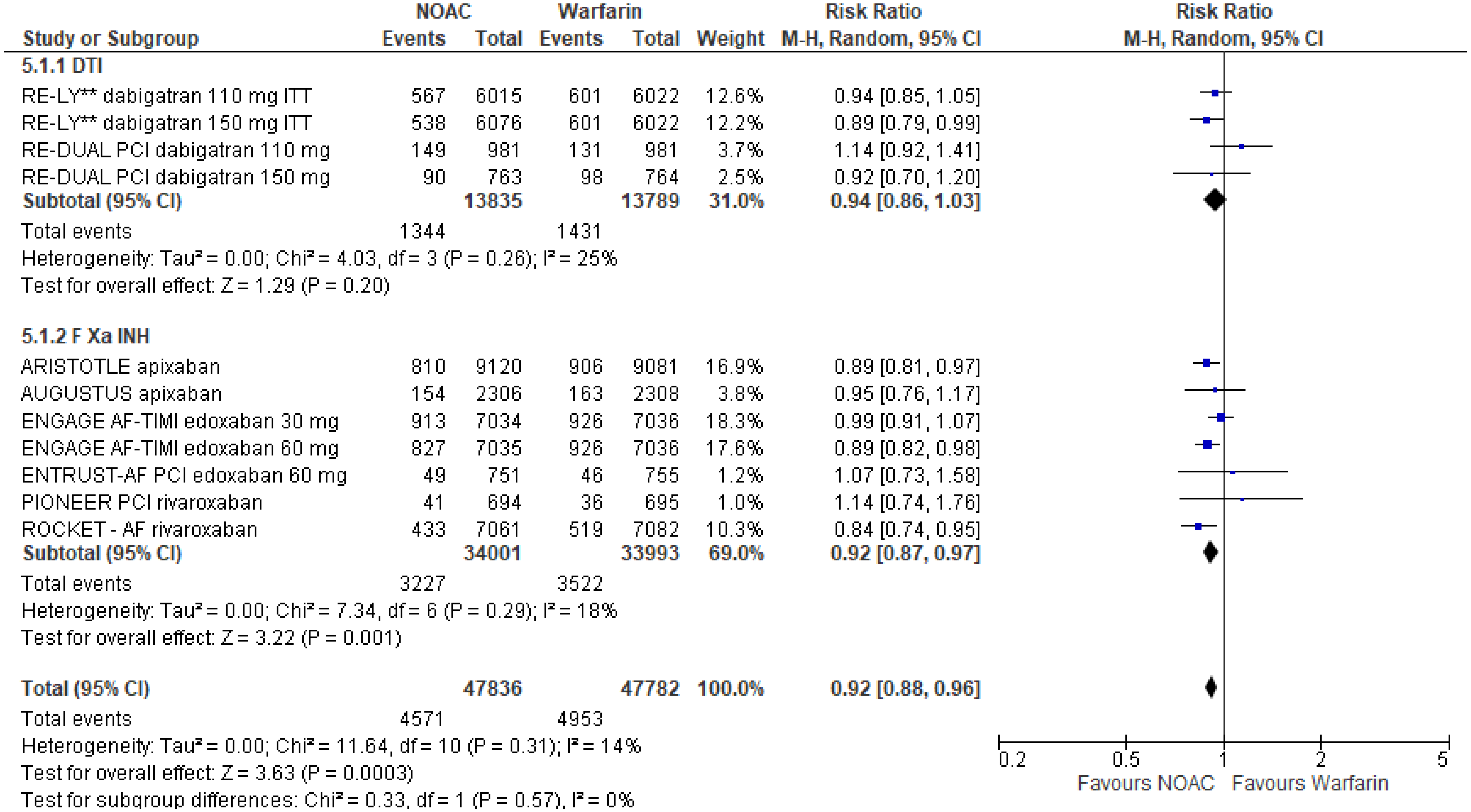

** - results from reanalysis of RE-LY study [27]

Figure S10A. The meta-analysis results for MACE – reanalysis RE-LY [27] -intention to treat data after combining study data with respect to doses.

Study or Subgroup	NOAC Events	NOAC Total	Warfarin Events	Warfarin Total	Weight	Risk Ratio M-H, Random, 95% CI
**38.1.1 DTI**						
RE-LY** dabigatran ITT	1105	12091	601	6022	20.7%	0.92 [0.83, 1.01]
RE-DUAL PCI dabigatran	239	1744	131	981	4.7%	1.03 [0.84, 1.25]
**Subtotal (95% CI)**		**13835**		**7003**	**25.4%**	**0.94 [0.86, 1.02]**
Total events	1344		732			
Heterogeneity: Tau² = 0.00; Chi² = 1.03, df = 1 (P = 0.31); I² = 3%						
Test for overall effect: Z = 1.45 (P = 0.15)						
**38.1.2 F Xa INH**						
ARISTOTLE apixaban	810	9120	906	9081	22.7%	0.89 [0.81, 0.97]
AUGUSTUS apixaban	154	2306	163	2308	4.1%	0.95 [0.76, 1.17]
ENGAGE-AF-TIMI edoxaban	1740	14069	926	7036	33.4%	0.94 [0.87, 1.01]
ENTRUST-AF PCI edoxaban 60 mg	49	751	46	755	1.2%	1.07 [0.73, 1.58]
PIONEER PCI rivaroxaban	41	694	36	695	1.0%	1.14 [0.74, 1.76]
ROCKET - AF rivaroxaban	433	7061	519	7082	12.2%	0.84 [0.74, 0.95]
**Subtotal (95% CI)**		**34001**		**26957**	**74.6%**	**0.91 [0.87, 0.96]**
Total events	3227		2596			
Heterogeneity: Tau² = 0.00; Chi² = 4.55, df = 5 (P = 0.47); I² = 0%						
Test for overall effect: Z = 3.64 (P = 0.0003)						
**Total (95% CI)**		**47836**		**33960**	**100.0%**	**0.92 [0.88, 0.96]**
Total events	4571		3328			
Heterogeneity: Tau² = 0.00; Chi² = 5.85, df = 7 (P = 0.56); I² = 0%						
Test for overall effect: Z = 3.92 (P < 0.0001)						
Test for subgroup differences: Chi² = 0.27, df = 1 (P = 0.60), I² = 0%						

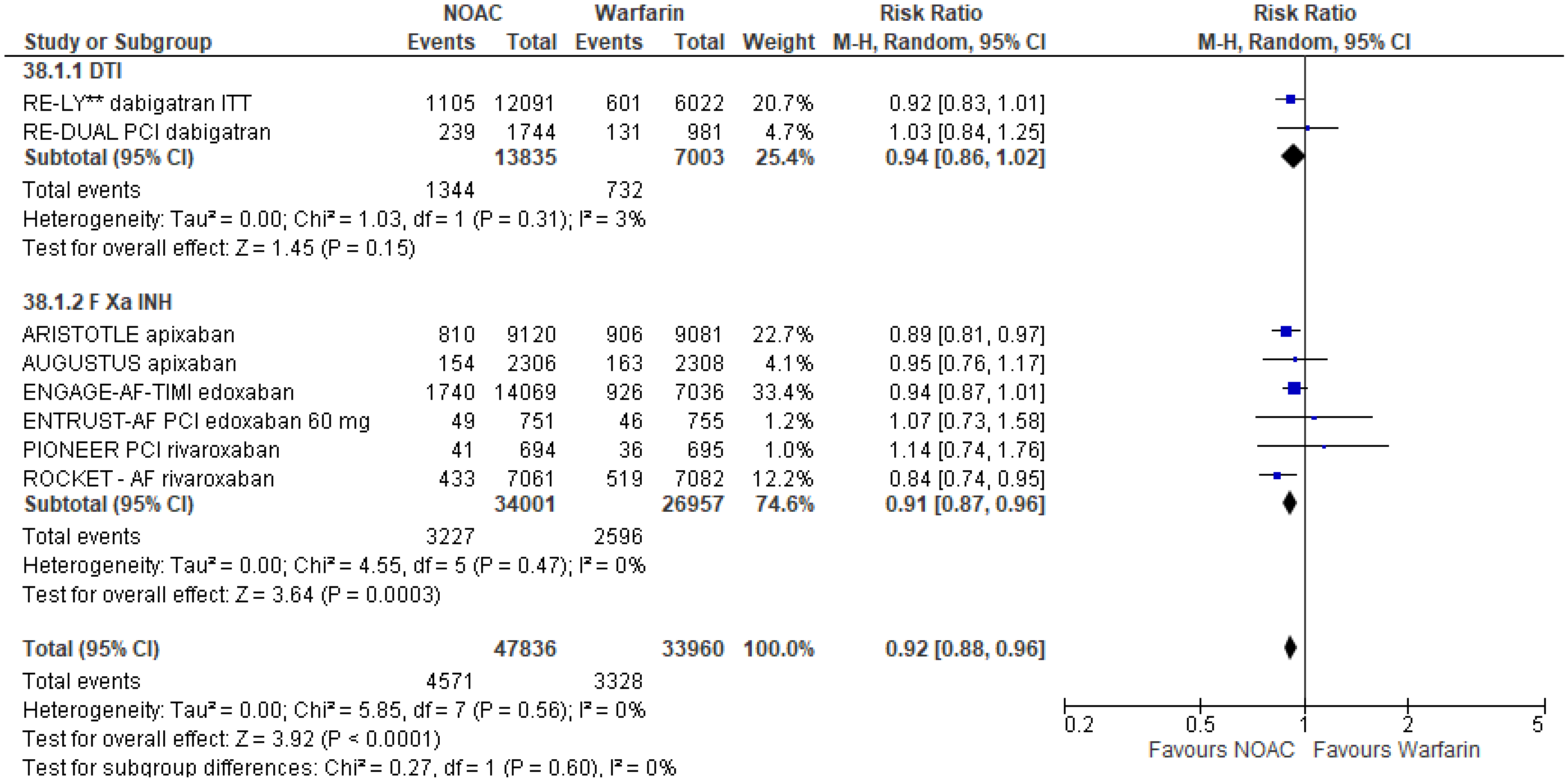

** - results from reanalysis of RE-LY study [27]

Figure S11. The funnel plot for MACE – re-analysis RE-LY [27] -intention to treat data.

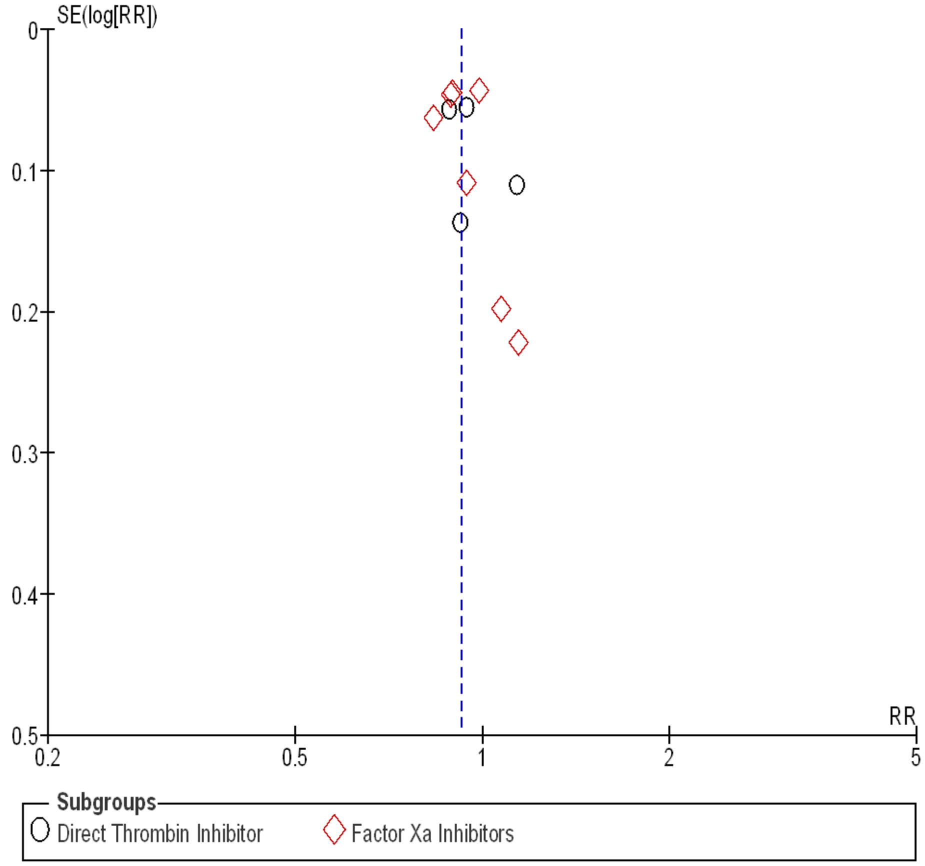

Figure S12. The meta-analysis results for MACE – reanalysis RE-LY [27] -intention to treat data after excluding ENGAGE AF-TIMI edoxaban 30 mg

Study or Subgroup	NOAC Events	NOAC Total	Warfarin Events	Warfarin Total	Weight	Risk Ratio M-H, Random, 95% CI
**6.1.1 DTI**						
RE-LY** dabigatran 110 mg ITT	567	6015	601	6022	15.2%	0.94 [0.85, 1.05]
RE-LY** dabigatran 150 mg ITT	538	6076	601	6022	14.7%	0.89 [0.79, 0.99]
RE-DUAL PCI dabigatran 110 mg	149	981	131	981	3.8%	1.14 [0.92, 1.41]
RE-DUAL PCI dabigatran 150 mg	90	763	98	764	2.5%	0.92 [0.70, 1.20]
**Subtotal (95% CI)**		**13835**		**13789**	**36.3%**	**0.94 [0.86, 1.03]**
Total events	1344		1431			
Heterogeneity: Tau² = 0.00; Chi² = 4.03, df = 3 (P = 0.26); I² = 25%						
Test for overall effect: Z = 1.29 (P = 0.20)						
**6.1.2 F Xa INH**						
ARISTOTLE apixaban	810	9120	906	9081	22.2%	0.89 [0.81, 0.97]
AUGUSTUS apixaban	154	2306	163	2308	4.0%	0.95 [0.76, 1.17]
ENGAGE AF-TIMI edoxaban 60 mg	827	7035	926	7036	23.5%	0.89 [0.82, 0.98]
ENTRUST-AF PCI edoxaban 60 mg	49	751	46	755	1.2%	1.07 [0.73, 1.58]
PIONEER PCI rivaroxaban	41	694	36	695	1.0%	1.14 [0.74, 1.76]
ROCKET - AF rivaroxaban	433	7061	519	7082	11.9%	0.84 [0.74, 0.95]
**Subtotal (95% CI)**		**26967**		**26957**	**63.7%**	**0.89 [0.84, 0.94]**
Total events	2314		2596			
Heterogeneity: Tau² = 0.00; Chi² = 3.39, df = 5 (P = 0.64); I² = 0%						
Test for overall effect: Z = 4.26 (P < 0.0001)						
**Total (95% CI)**		**40802**		**40746**	**100.0%**	**0.91 [0.87, 0.95]**
Total events	3658		4027			
Heterogeneity: Tau² = 0.00; Chi² = 8.69, df = 9 (P = 0.47); I² = 0%						
Test for overall effect: Z = 4.48 (P < 0.00001)						
Test for subgroup differences: Chi² = 1.23, df = 1 (P = 0.27), I² = 19.0%						

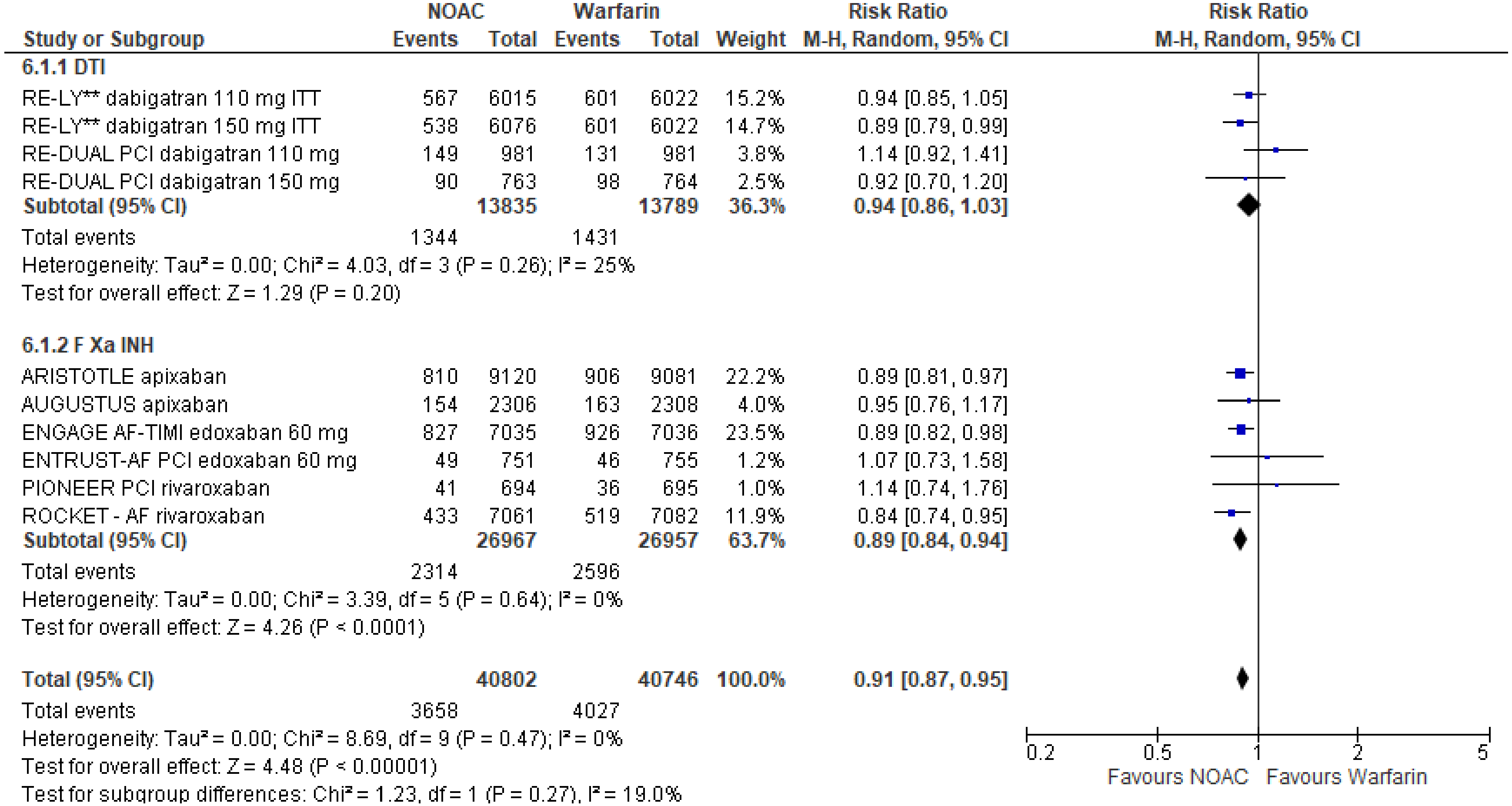

** - results from reanalysis of RE-LY study [27]

Figure S12A. The meta-analysis results for MACE – reanalysis RE-LY [27] -intention to treat data after excluding ENGAGE AF-TIMI edoxaban 30 mg and combining study data with respect to doses.

Study or Subgroup	NOAC Events	NOAC Total	Warfarin Events	Warfarin Total	Weight	Risk Ratio M-H, Random, 95% CI
**39.1.1 DTI**						
RE-LY** dabigatran ITT	1105	12091	601	6022	22.9%	0.92 [0.83, 1.01]
RE-DUAL PCI dabigatran	239	1744	131	981	5.2%	1.03 [0.84, 1.25]
**Subtotal (95% CI)**		**13835**		**7003**	**28.1%**	**0.94 [0.86, 1.02]**
Total events	1344		732			
Heterogeneity: Tau² = 0.00; Chi² = 1.03, df = 1 (P = 0.31); I² = 3%						
Test for overall effect: Z = 1.45 (P = 0.15)						
**39.1.2 F Xa INH**						
ARISTOTLE apixaban	810	9120	906	9081	25.1%	0.89 [0.81, 0.97]
AUGUSTUS apixaban	154	2306	163	2308	4.5%	0.95 [0.76, 1.17]
ENGAGE-AF-TIMI edoxaban	827	7035	926	7036	26.5%	0.89 [0.82, 0.98]
ENTRUST-AF PCI edoxaban 60 mg	49	751	46	755	1.3%	1.07 [0.73, 1.58]
PIONEER PCI rivaroxaban	41	694	36	695	1.1%	1.14 [0.74, 1.76]
ROCKET - AF rivaroxaban	433	7061	519	7082	13.4%	0.84 [0.74, 0.95]
**Subtotal (95% CI)**		**26967**		**26957**	**71.9%**	**0.89 [0.84, 0.94]**
Total events	2314		2596			
Heterogeneity: Tau² = 0.00; Chi² = 3.39, df = 5 (P = 0.64); I² = 0%						
Test for overall effect: Z = 4.26 (P < 0.0001)						
**Total (95% CI)**		**40802**		**33960**	**100.0%**	**0.90 [0.86, 0.94]**
Total events	3658		3328			
Heterogeneity: Tau² = 0.00; Chi² = 5.33, df = 7 (P = 0.62); I² = 0%						
Test for overall effect: Z = 4.43 (P < 0.00001)						
Test for subgroup differences: Chi² = 0.90, df = 1 (P = 0.34), I² = 0%						

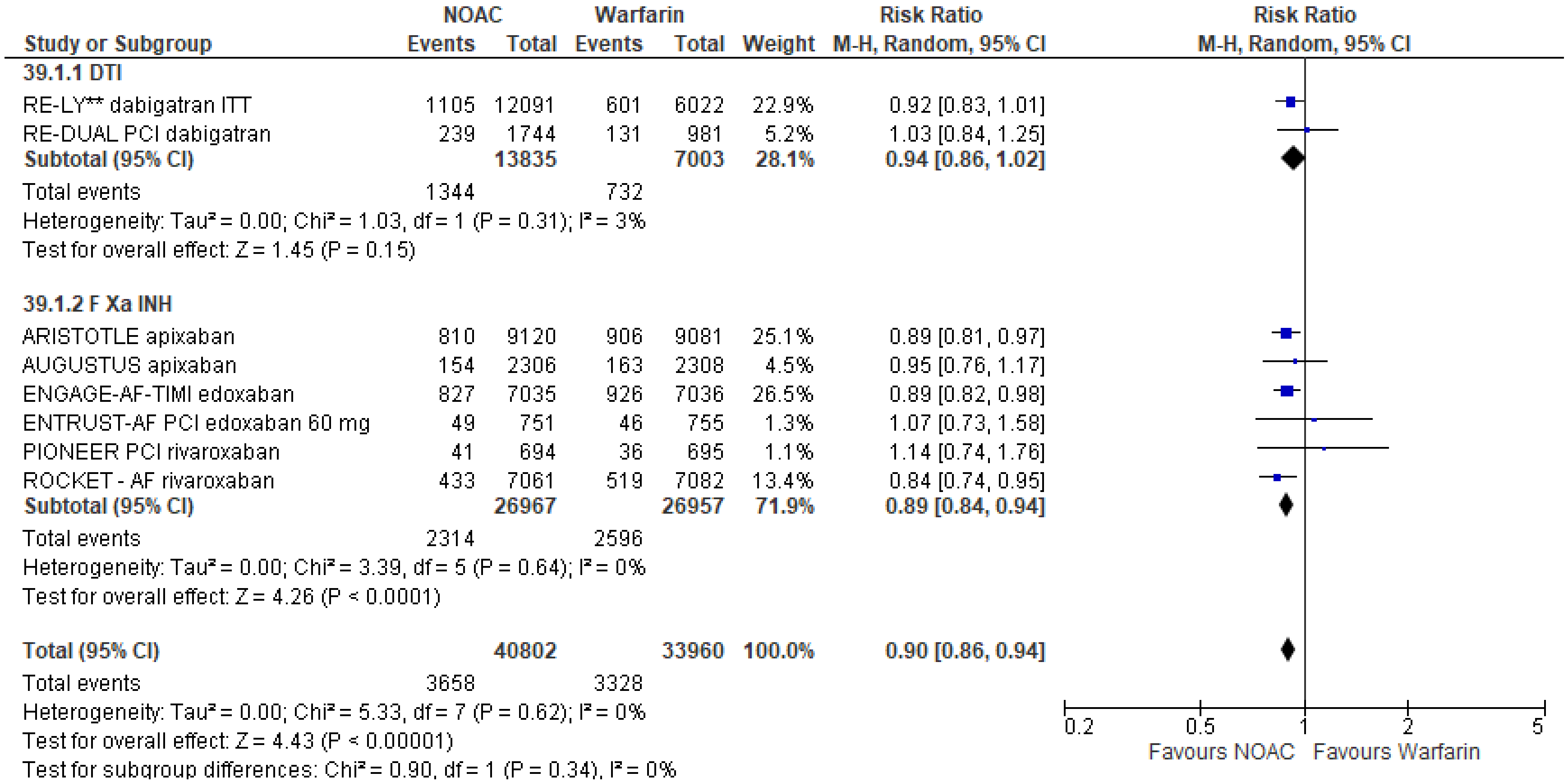

** - results from reanalysis of RE-LY study [27]

Figure S13. The funnel plot for MACE – reanalysis RE-LY [27] -intention to treat data after excluding ENGAGE AF-TIMI edoxaban 30 mg.

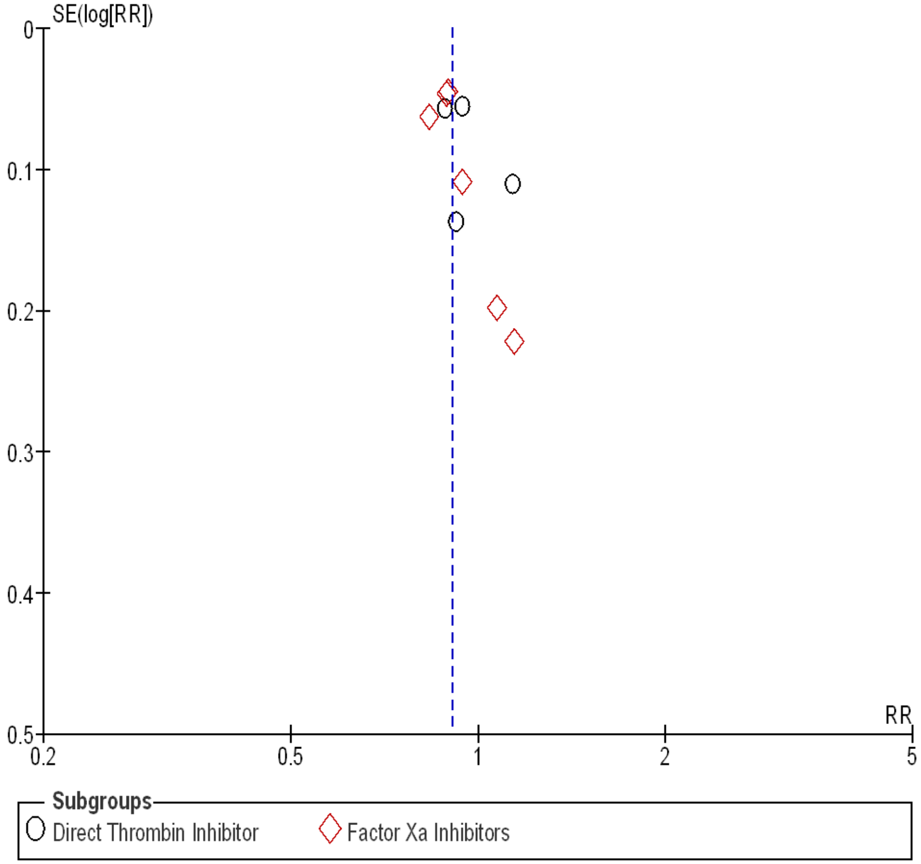

Figure S14. The meta-analysis results for MACE – reanalysis RE-LY [27] -on treatment data.

Study or Subgroup	NOAC Events	NOAC Total	Warfarin Events	Warfarin Total	Weight	Risk Ratio M-H, Random, 95% CI
**7.1.1 DTI**						
RE-LY** dabigatran 110mg on-T	517	5983	545	5998	12.1%	0.95 [0.85, 1.07]
RE-LY** dabigatran 150mg on-T	467	6059	545	5998	11.6%	0.85 [0.75, 0.95]
RE-DUAL PCI dabigatran 110 mg	149	981	131	981	4.3%	1.14 [0.92, 1.41]
RE-DUAL PCI dabigatran 150 mg	90	763	98	764	2.9%	0.92 [0.70, 1.20]
**Subtotal (95% CI)**		**13786**		**13741**	**30.8%**	**0.94 [0.84, 1.05]**
Total events	1223		1319			
Heterogeneity: Tau² = 0.01; Chi² = 5.76, df = 3 (P = 0.12); I² = 48%						
Test for overall effect: Z = 1.07 (P = 0.28)						
**7.1.2 F Xa INH**						
ARISTOTLE apixaban	810	9120	906	9081	16.5%	0.89 [0.81, 0.97]
AUGUSTUS apixaban	154	2306	163	2308	4.4%	0.95 [0.76, 1.17]
ENGAGE AF-TIMI edoxaban 30 mg	913	7034	926	7036	17.7%	0.99 [0.91, 1.07]
ENGAGE AF-TIMI edoxaban 60 mg	827	7035	926	7036	17.1%	0.89 [0.82, 0.98]
ENTRUST-AF PCI edoxaban 60 mg	49	751	46	755	1.4%	1.07 [0.73, 1.58]
PIONEER PCI rivaroxaban	41	694	36	695	1.2%	1.14 [0.74, 1.76]
ROCKET - AF rivaroxaban	433	7061	519	7082	10.9%	0.84 [0.74, 0.95]
**Subtotal (95% CI)**		**34001**		**33993**	**69.2%**	**0.92 [0.87, 0.97]**
Total events	3227		3522			
Heterogeneity: Tau² = 0.00; Chi² = 7.34, df = 6 (P = 0.29); I² = 18%						
Test for overall effect: Z = 3.22 (P = 0.001)						
**Total (95% CI)**		**47787**		**47734**	**100.0%**	**0.92 [0.88, 0.97]**
Total events	4450		4841			
Heterogeneity: Tau² = 0.00; Chi² = 13.15, df = 10 (P = 0.22); I² = 24%						
Test for overall effect: Z = 3.40 (P = 0.0007)						
Test for subgroup differences: Chi² = 0.17, df = 1 (P = 0.68), I² = 0%						

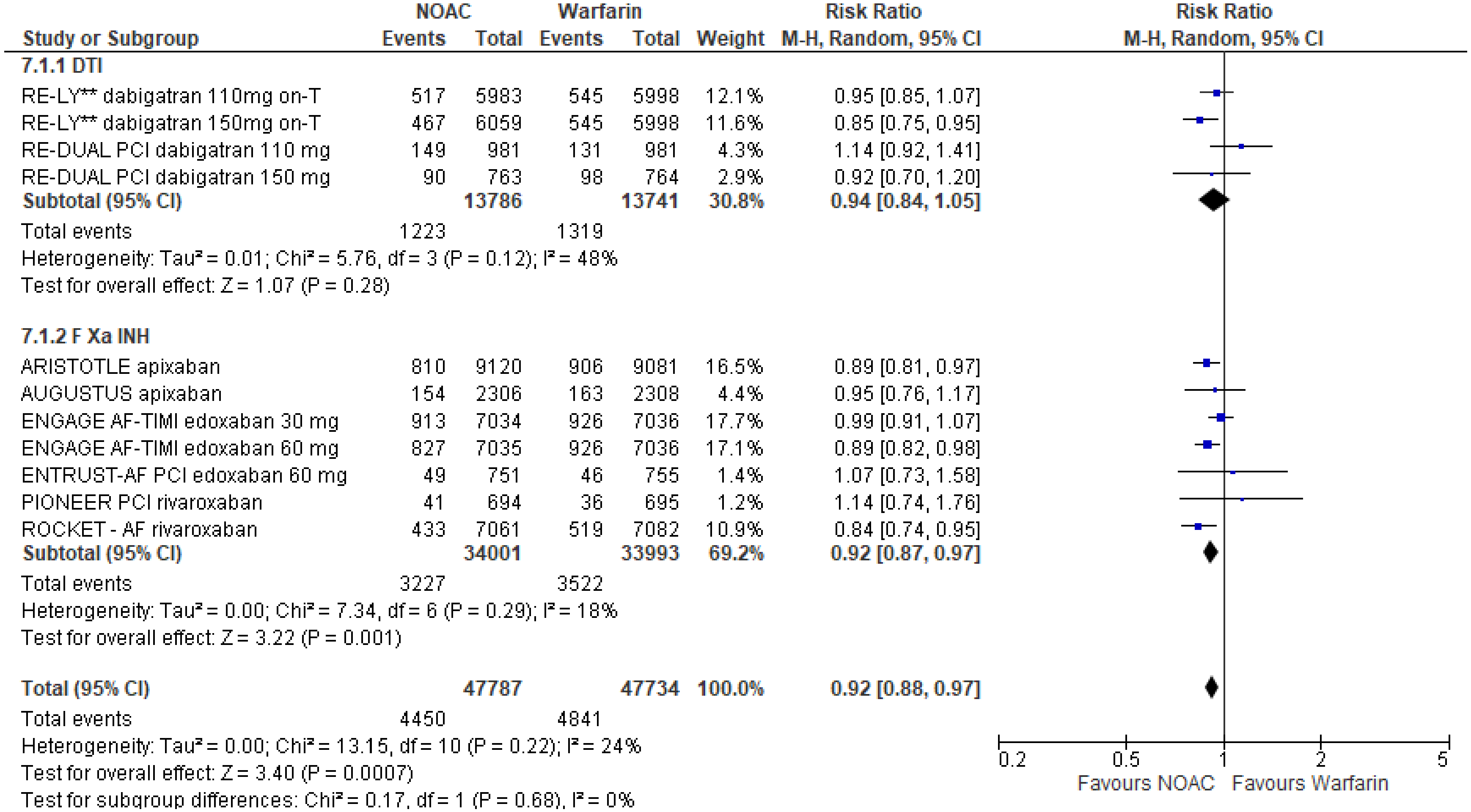

** - results from reanalysis of RE-LY study [27]

Figure S14A. The meta-analysis results for MACE – reanalysis RE-LY [27] -on treatment data after combining study data with respect to doses.

Study or Subgroup	NOAC Events	NOAC Total	Warfarin Events	Warfarin Total	Weight	Risk Ratio M-H, Random, 95% CI
**40.1.1 DTI**						
RE-LY** dabigatran on-T	984	12042	545	5998	18.9%	0.90 [0.81, 0.99]
RE-DUAL PCI dabigatran	239	1744	131	981	4.8%	1.03 [0.84, 1.25]
**Subtotal (95% CI)**		**13786**		**6979**	**23.7%**	**0.93 [0.83, 1.05]**
Total events	1223		676			
Heterogeneity: Tau² = 0.00; Chi² = 1.36, df = 1 (P = 0.24); I² = 26%						
Test for overall effect: Z = 1.16 (P = 0.25)						
**40.1.2 F Xa INH**						
ARISTOTLE apixaban	810	9120	906	9081	23.2%	0.89 [0.81, 0.97]
AUGUSTUS apixaban	154	2306	163	2308	4.2%	0.95 [0.76, 1.17]
ENGAGE-AF-TIMI edoxaban	1740	14069	926	7036	34.1%	0.94 [0.87, 1.01]
ENTRUST-AF PCI edoxaban 60 mg	49	751	46	755	1.2%	1.07 [0.73, 1.58]
PIONEER PCI rivaroxaban	41	694	36	695	1.0%	1.14 [0.74, 1.76]
ROCKET - AF rivaroxaban	433	7061	519	7082	12.5%	0.84 [0.74, 0.95]
**Subtotal (95% CI)**		**34001**		**26957**	**76.3%**	**0.91 [0.87, 0.96]**
Total events	3227		2596			
Heterogeneity: Tau² = 0.00; Chi² = 4.55, df = 5 (P = 0.47); I² = 0%						
Test for overall effect: Z = 3.64 (P = 0.0003)						
**Total (95% CI)**		**47787**		**33936**	**100.0%**	**0.91 [0.88, 0.96]**
Total events	4450		3272			
Heterogeneity: Tau² = 0.00; Chi² = 5.98, df = 7 (P = 0.54); I² = 0%						
Test for overall effect: Z = 4.03 (P < 0.0001)						
Test for subgroup differences: Chi² = 0.13, df = 1 (P = 0.71), I² = 0%						

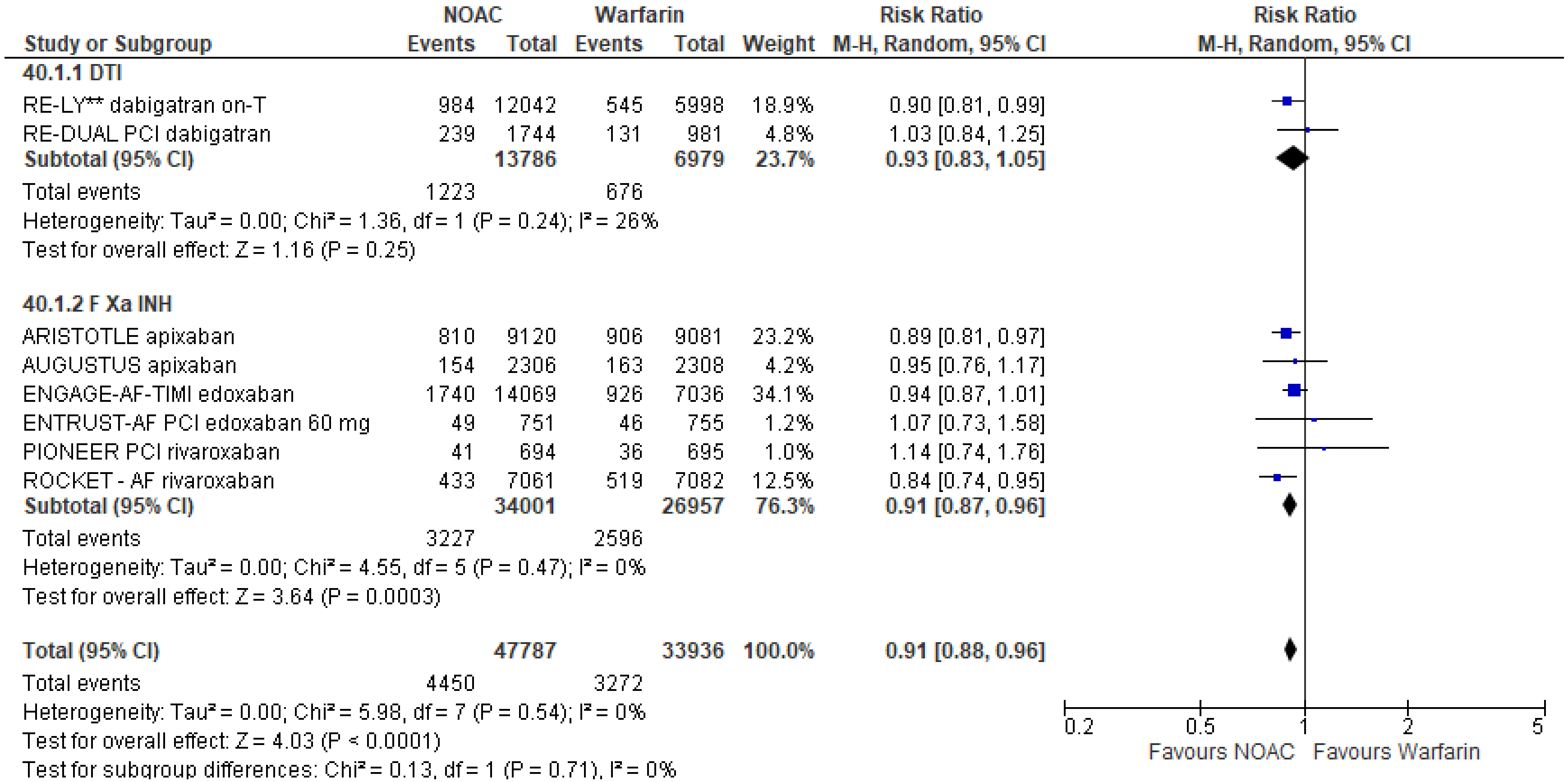

** - results from reanalysis of RE-LY study [27]

Figure S15. The funnel plot for MACE – reanalysis RE-LY [27] - on treatment data.

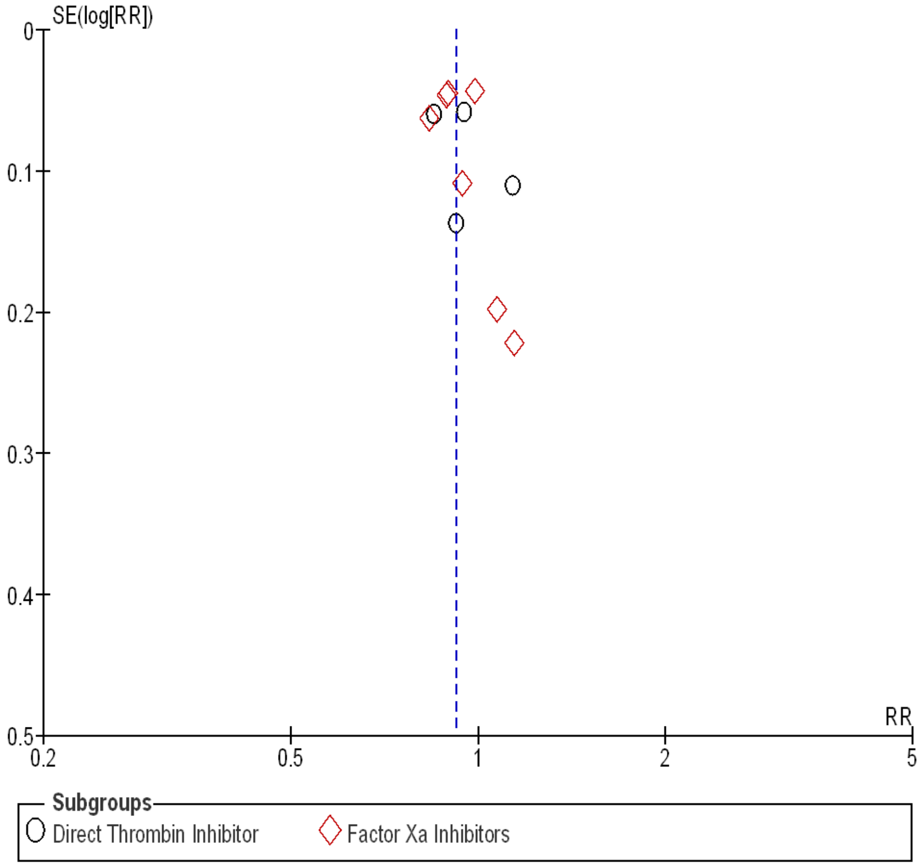

Figure S16. The meta-analysis results for MACE – reanalysis RE-LY [27] -on treatment data after excluding ENGAGE AF-TIMI edoxaban 30 mg.

Study or Subgroup	NOAC Events	NOAC Total	Warfarin Events	Warfarin Total	Weight	Risk Ratio M-H, Random, 95% CI
**8.1.1 DTI**						
RE-LY** dabigatran 110mg on-T	517	5983	545	5998	14.5%	0.95 [0.85, 1.07]
RE-LY** dabigatran 150mg on-T	467	6059	545	5998	13.7%	0.85 [0.75, 0.95]
RE-DUAL PCI dabigatran 110 mg	149	981	131	981	4.4%	1.14 [0.92, 1.41]
RE-DUAL PCI dabigatran 150 mg	90	763	98	764	3.0%	0.92 [0.70, 1.20]
**Subtotal (95% CI)**		**13786**		**13741**	**35.6%**	**0.94 [0.84, 1.05]**
Total events	1223		1319			
Heterogeneity: Tau² = 0.01; Chi² = 5.76, df = 3 (P = 0.12); I² = 48%						
Test for overall effect: Z = 1.07 (P = 0.28)						
**8.1.2 F Xa INH**						
ARISTOTLE apixaban	810	9120	906	9081	21.7%	0.89 [0.81, 0.97]
AUGUSTUS apixaban	154	2306	163	2308	4.6%	0.95 [0.76, 1.17]
ENGAGE AF-TIMI edoxaban 60 mg	827	7035	926	7036	22.7%	0.89 [0.82, 0.98]
ENTRUST-AF PCI edoxaban 60 mg	49	751	46	755	1.4%	1.07 [0.73, 1.58]
PIONEER PCI rivaroxaban	41	694	36	695	1.1%	1.14 [0.74, 1.76]
ROCKET - AF rivaroxaban	433	7061	519	7082	12.8%	0.84 [0.74, 0.95]
**Subtotal (95% CI)**		**26967**		**26957**	**64.4%**	**0.89 [0.84, 0.94]**
Total events	2314		2596			
Heterogeneity: Tau² = 0.00; Chi² = 3.39, df = 5 (P = 0.64); I² = 0%						
Test for overall effect: Z = 4.26 (P < 0.0001)						
**Total (95% CI)**		**40753**		**40698**	**100.0%**	**0.90 [0.86, 0.95]**
Total events	3537		3915			
Heterogeneity: Tau² = 0.00; Chi² = 9.85, df = 9 (P = 0.36); I² = 9%						
Test for overall effect: Z = 4.21 (P < 0.0001)						
Test for subgroup differences: Chi² = 0.73, df = 1 (P = 0.39), I² = 0%						

Risk Ratio M-H, Random, 95% CI

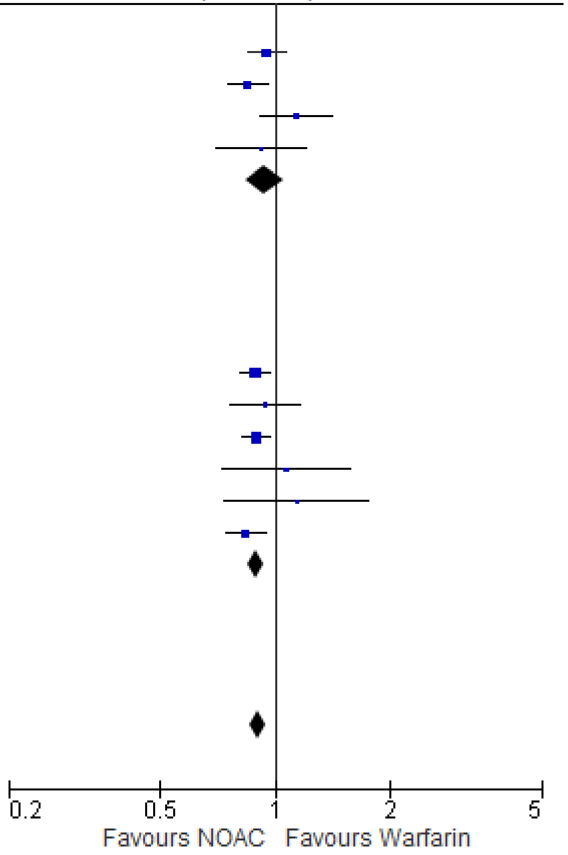

** - results from reanalysis of RE-LY study [27]

Figure S16A. The meta-analysis results for MACE – reanalysis RE-LY [27] -on treatment data after excluding ENGAGE AF-TIMI edoxaban 30 mg and combining study data with respect to doses.

Study or Subgroup	NOAC Events	NOAC Total	Warfarin Events	Warfarin Total	Weight	Risk Ratio M-H, Random, 95% CI
**41.1.1 DTI**						
RE-LY** dabigatran on-T	984	12042	545	5998	20.9%	0.90 [0.81, 0.99]
RE-DUAL PCI dabigatran	239	1744	131	981	5.3%	1.03 [0.84, 1.25]
**Subtotal (95% CI)**		**13786**		**6979**	**26.3%**	**0.93 [0.83, 1.05]**
Total events	1223		676			
Heterogeneity: Tau² = 0.00; Chi² = 1.36, df = 1 (P = 0.24); I² = 26%						
Test for overall effect: Z = 1.16 (P = 0.25)						
**41.1.2 F Xa INH**						
ARISTOTLE apixaban	810	9120	906	9081	25.7%	0.89 [0.81, 0.97]
AUGUSTUS apixaban	154	2306	163	2308	4.6%	0.95 [0.76, 1.17]
ENGAGE-AF-TIMI edoxaban	827	7035	926	7036	27.2%	0.89 [0.82, 0.98]
ENTRUST-AF PCI edoxaban 60 mg	49	751	46	755	1.4%	1.07 [0.73, 1.58]
PIONEER PCI rivaroxaban	41	694	36	695	1.1%	1.14 [0.74, 1.76]
ROCKET - AF rivaroxaban	433	7061	519	7082	13.8%	0.84 [0.74, 0.95]
**Subtotal (95% CI)**		**26967**		**26957**	**73.7%**	**0.89 [0.84, 0.94]**
Total events	2314		2596			
Heterogeneity: Tau² = 0.00; Chi² = 3.39, df = 5 (P = 0.64); I² = 0%						
Test for overall effect: Z = 4.26 (P < 0.0001)						
**Total (95% CI)**		**40753**		**33936**	**100.0%**	**0.90 [0.86, 0.94]**
Total events	3537		3272			
Heterogeneity: Tau² = 0.00; Chi² = 5.22, df = 7 (P = 0.63); I² = 0%						
Test for overall effect: Z = 4.55 (P < 0.00001)						
Test for subgroup differences: Chi² = 0.51, df = 1 (P = 0.47), I² = 0%						

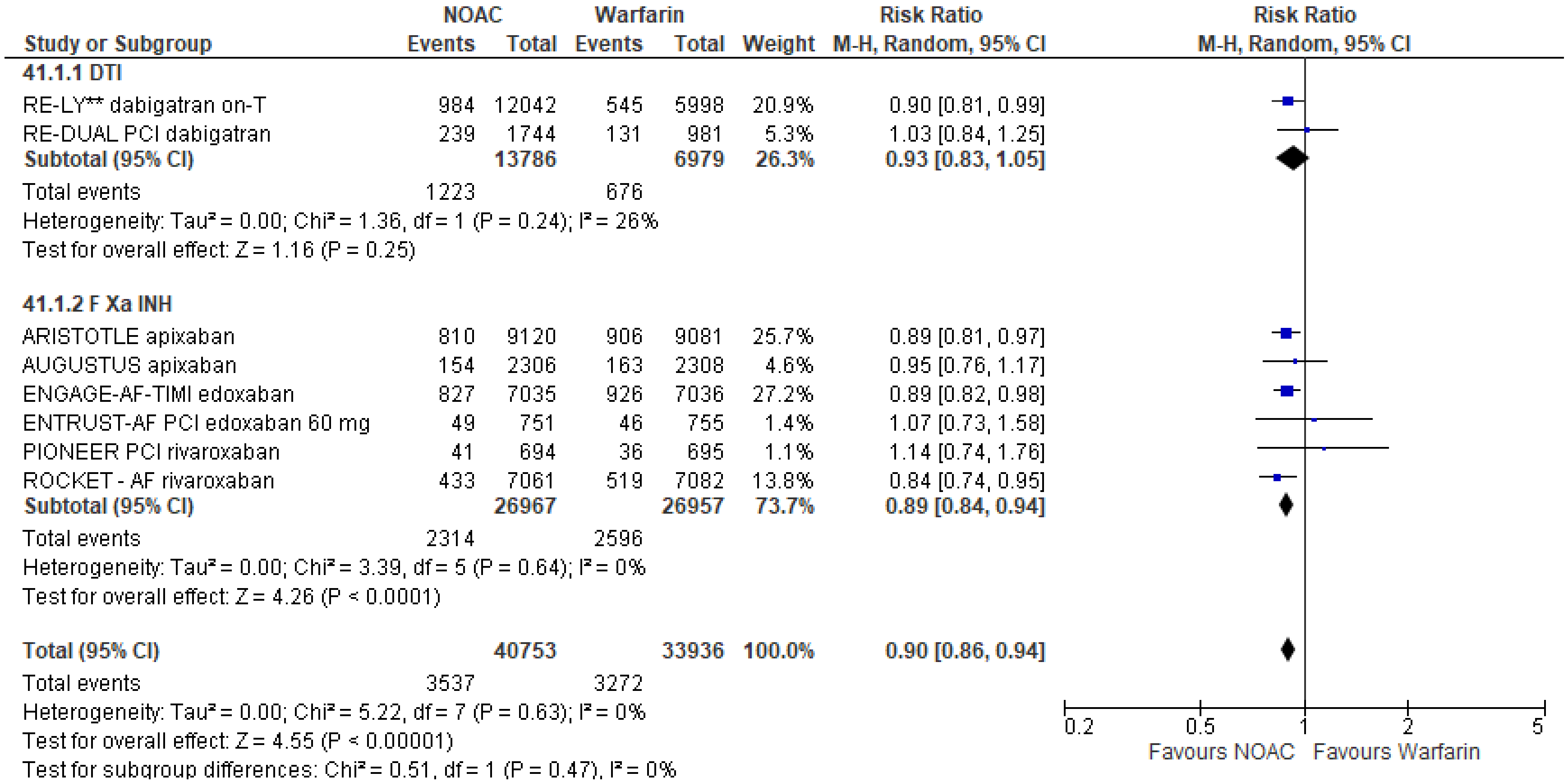

** - results from reanalysis of RE-LY study [27]

Figure S17. The funnel plot for MACE – reanalysis RE-LY [27] on treatment data after excluding ENGAGE AF-TIMI edoxaban 30 mg.

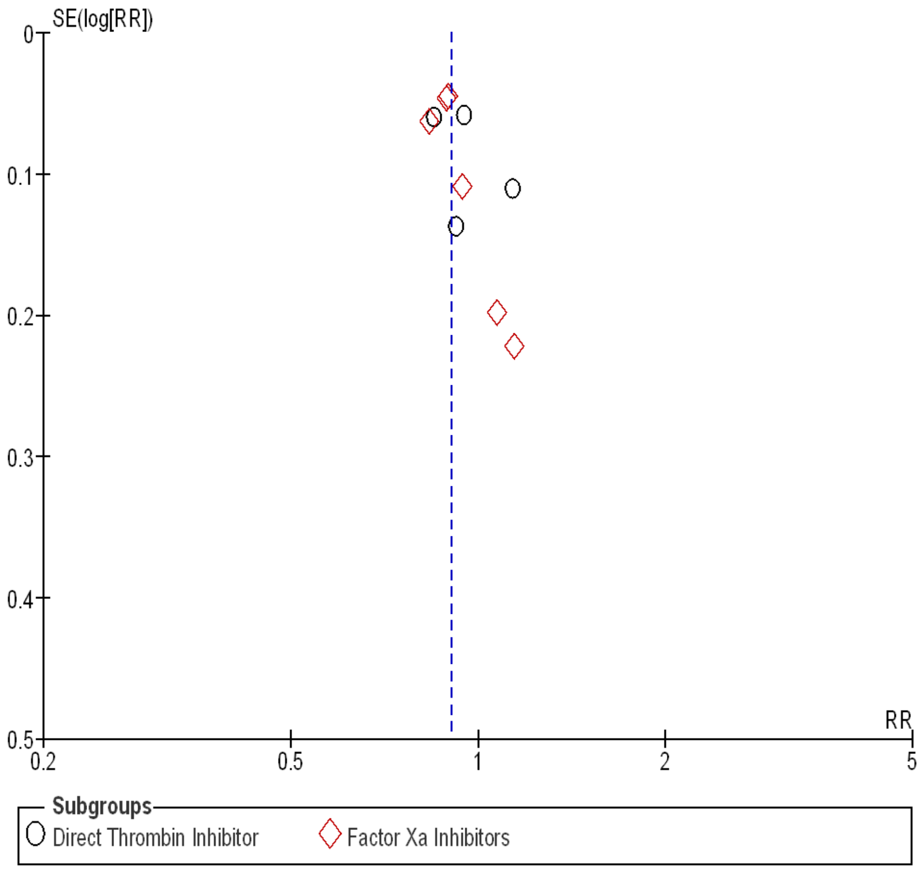

Figure S18. The meta-analysis results for MI.

Study or Subgroup	NOAC Events	NOAC Total	Warfarin Events	Warfarin Total	Weight	Risk Ratio M-H, Random, 95% CI
**9.1.1 DTI**						
RE-LY* dabigatran 110 mg	86	6015	63	6022	9.8%	1.37 [0.99, 1.89]
RE-LY* dabigatran 150 mg	89	6076	63	6022	9.8%	1.40 [1.02, 1.93]
RE-DUAL PCI dabigatran 110 mg	44	981	29	981	6.2%	1.52 [0.96, 2.40]
RE-DUAL PCI dabigatran 150 mg	26	763	22	764	4.6%	1.18 [0.68, 2.07]
**Subtotal (95% CI)**		**13835**		**13789**	**30.4%**	**1.38 [1.14, 1.67]**
Total events	245		177			
Heterogeneity: Tau² = 0.00; Chi² = 0.46, df = 3 (P = 0.93); I² = 0%						
Test for overall effect: Z = 3.29 (P = 0.0010)						
**9.1.2 F Xa INH**						
ARISTOTLE apixaban	90	9120	102	9081	11.3%	0.88 [0.66, 1.16]
AUGUSTUS apixaban	72	2290	80	2259	10.1%	0.89 [0.65, 1.21]
ENGAGE AF-TIMI edoxaban 30 mg	169	7034	141	7036	13.9%	1.20 [0.96, 1.50]
ENGAGE AF-TIMI edoxaban 60 mg	133	7035	141	7036	13.3%	0.94 [0.75, 1.19]
ENTRUST-AF PCI edoxaban 60 mg	29	751	23	755	4.9%	1.27 [0.74, 2.17]
PIONEER PCI rivaroxaban	19	694	21	695	4.0%	0.91 [0.49, 1.67]
ROCKET - AF rivaroxaban	101	7061	126	7082	12.2%	0.80 [0.62, 1.04]
**Subtotal (95% CI)**		**33985**		**33944**	**69.6%**	**0.96 [0.85, 1.09]**
Total events	613		634			
Heterogeneity: Tau² = 0.01; Chi² = 7.36, df = 6 (P = 0.29); I² = 19%						
Test for overall effect: Z = 0.60 (P = 0.55)						
**Total (95% CI)**		**47820**		**47733**	**100.0%**	**1.07 [0.94, 1.23]**
Total events	858		811			
Heterogeneity: Tau² = 0.02; Chi² = 17.85, df = 10 (P = 0.06); I² = 44%						
Test for overall effect: Z = 1.01 (P = 0.31)						
Test for subgroup differences: Chi² = 9.51, df = 1 (P = 0.002), I² = 89.5%						

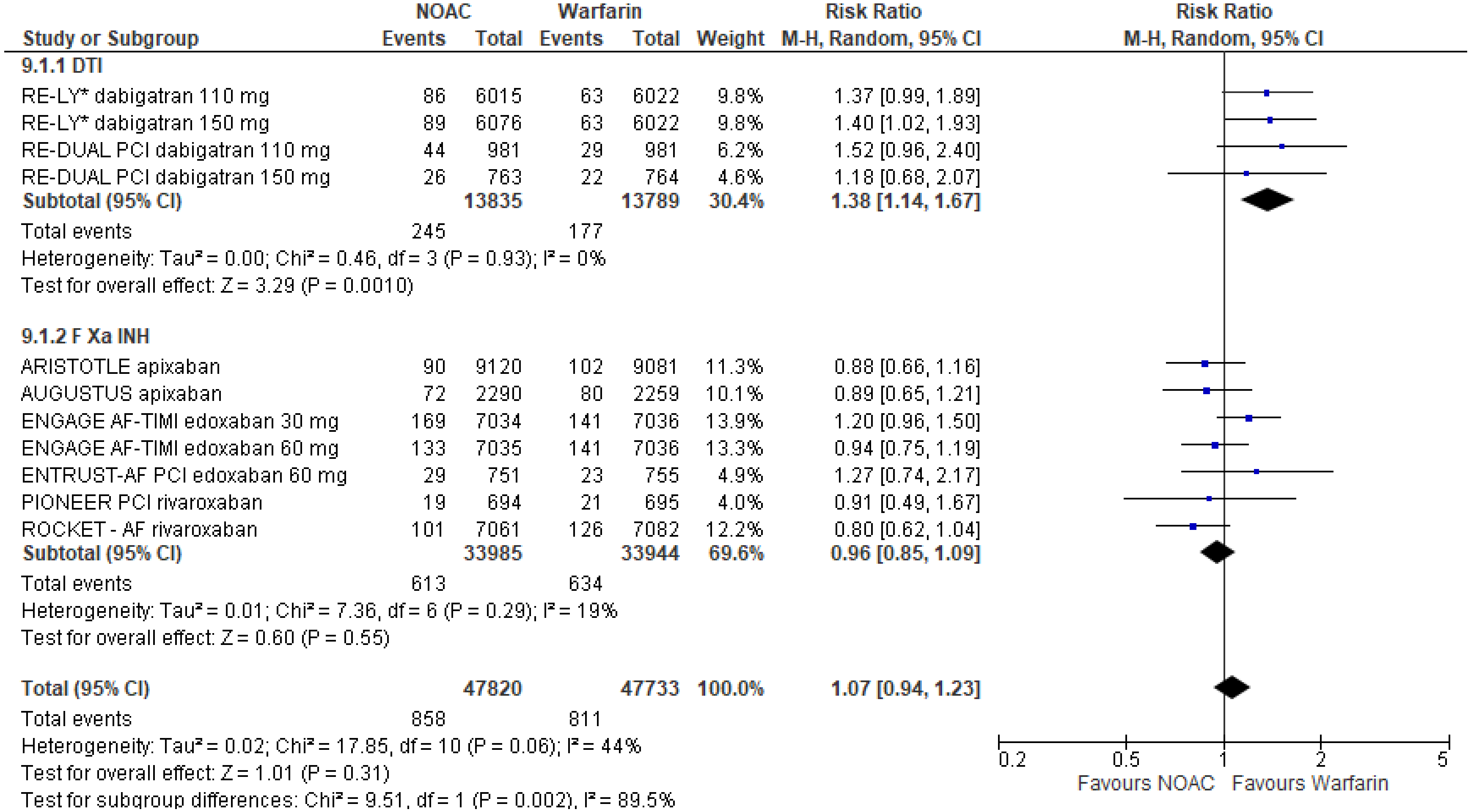

*- original data from RE-LY study [23]

Figure S18A. The meta-analysis results for MI after combining study data with respect to doses.

Study or Subgroup	NOAC Events	NOAC Total	Warfarin Events	Warfarin Total	Weight	Risk Ratio M-H, Random, 95% CI
**28.1.1 DTI**						
RE-LY* dabigatran	175	12091	63	6022	14.7%	1.38 [1.04, 1.84]
RE-DUAL PCI dabigatran	70	1744	29	981	9.0%	1.36 [0.89, 2.08]
**Subtotal (95% CI)**		**13835**		**7003**	**23.7%**	**1.38 [1.08, 1.74]**
Total events	245		92			
Heterogeneity: Tau² = 0.00; Chi² = 0.01, df = 1 (P = 0.94); I² = 0%						
Test for overall effect: Z = 2.63 (P = 0.009)						
**28.1.2 F Xa INH**						
ARISTOTLE apixaban	90	9120	102	9081	15.0%	0.88 [0.66, 1.16]
AUGUSTUS apixaban	72	2290	80	2259	13.3%	0.89 [0.65, 1.21]
ENGAGE-AF-TIMI edoxaban	302	14069	141	7036	20.4%	1.07 [0.88, 1.31]
ENTRUST-AF PCI edoxaban 60 mg	29	751	23	755	6.3%	1.27 [0.74, 2.17]
PIONEER PCI rivaroxaban	19	694	21	695	5.1%	0.91 [0.49, 1.67]
ROCKET - AF rivaroxaban	101	7061	126	7082	16.3%	0.80 [0.62, 1.04]
**Subtotal (95% CI)**		**33985**		**26908**	**76.3%**	**0.95 [0.84, 1.07]**
Total events	613		493			
Heterogeneity: Tau² = 0.00; Chi² = 4.60, df = 5 (P = 0.47); I² = 0%						
Test for overall effect: Z = 0.89 (P = 0.38)						
**Total (95% CI)**		**47820**		**33911**	**100.0%**	**1.03 [0.88, 1.20]**
Total events	858		585			
Heterogeneity: Tau² = 0.02; Chi² = 12.16, df = 7 (P = 0.10); I² = 42%						
Test for overall effect: Z = 0.37 (P = 0.71)						
Test for subgroup differences: Chi² = 7.55, df = 1 (P = 0.006), I² = 86.7%						

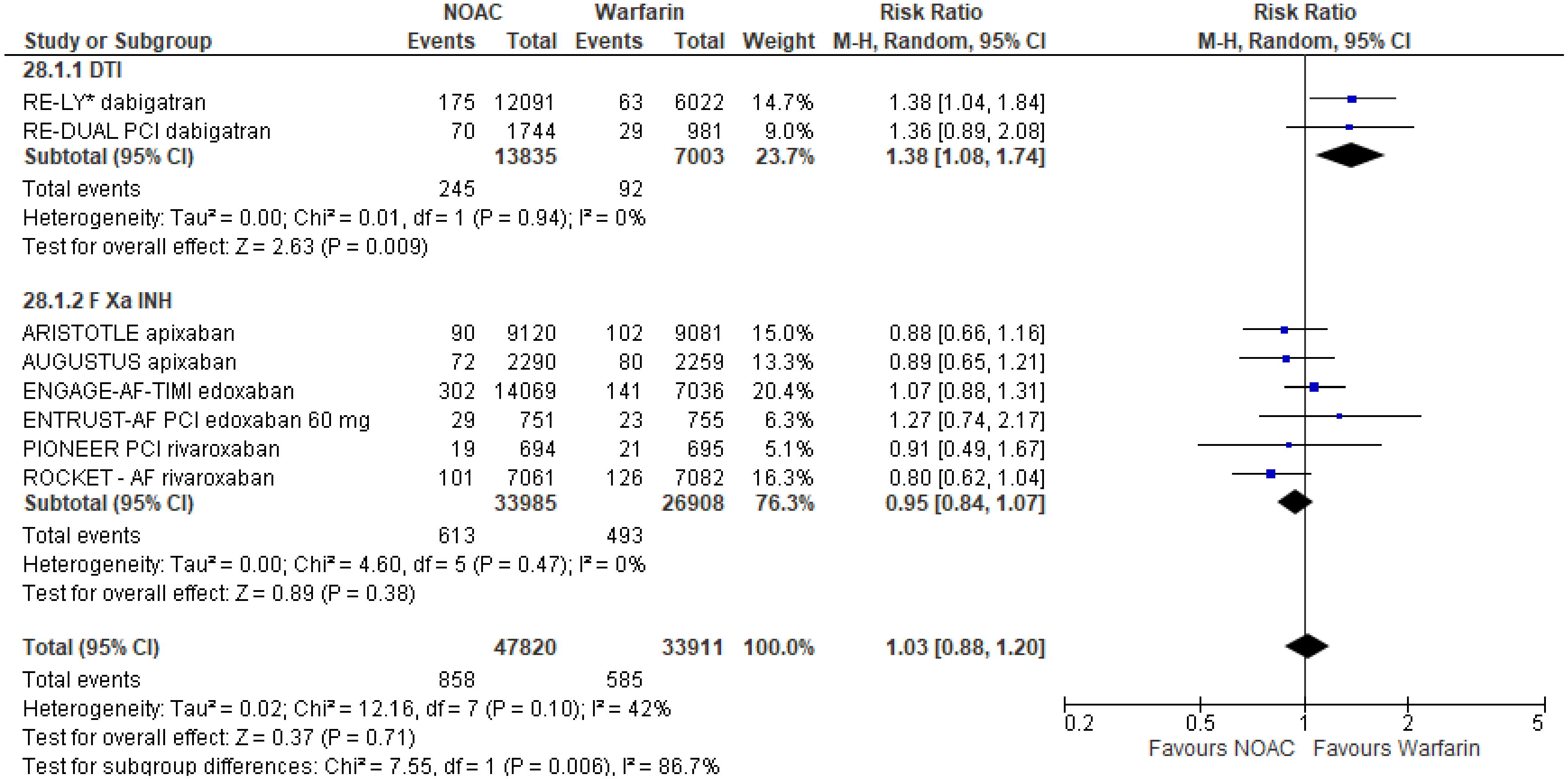

*- original data from RE-LY study [23]

Figure S19. The funnel plot for MI.

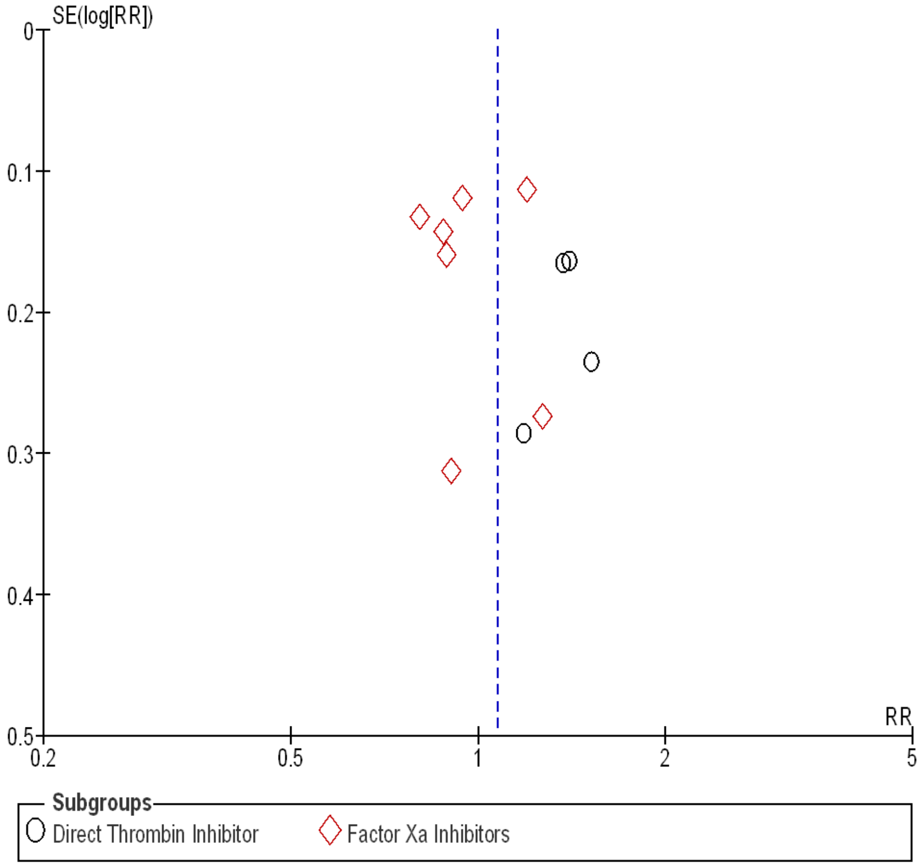

Figure S20. The meta-analysis results for MI after excluding ENGAGE AF-TIMI edoxaban 30 mg.

Study or Subgroup	NOAC Events	NOAC Total	Warfarin Events	Warfarin Total	Weight	Risk Ratio M-H, Random, 95% CI
**10.1.1 DTI**						
RE-LY* dabigatran 110 mg	86	6015	63	6022	11.3%	1.37 [0.99, 1.89]
RE-LY* dabigatran 150 mg	89	6076	63	6022	11.4%	1.40 [1.02, 1.93]
RE-DUAL PCI dabigatran 110 mg	44	981	29	981	7.4%	1.52 [0.96, 2.40]
RE-DUAL PCI dabigatran 150 mg	26	763	22	764	5.5%	1.18 [0.68, 2.07]
**Subtotal (95% CI)**		**13835**		**13789**	**35.6%**	**1.38 [1.14, 1.67]**
Total events	245		177			
Heterogeneity: Tau² = 0.00; Chi² = 0.46, df = 3 (P = 0.93); I² = 0%						
Test for overall effect: Z = 3.29 (P = 0.0010)						
**10.1.2 F Xa INH**						
ARISTOTLE apixaban	90	9120	102	9081	13.0%	0.88 [0.66, 1.16]
AUGUSTUS apixaban	72	2290	80	2259	11.7%	0.89 [0.65, 1.21]
ENGAGE AF-TIMI edoxaban 60 mg	133	7035	141	7036	15.1%	0.94 [0.75, 1.19]
ENTRUST-AF PCI edoxaban 60 mg	29	751	23	755	5.9%	1.27 [0.74, 2.17]
PIONEER PCI rivaroxaban	19	694	21	695	4.8%	0.91 [0.49, 1.67]
ROCKET - AF rivaroxaban	101	7061	126	7082	13.9%	0.80 [0.62, 1.04]
**Subtotal (95% CI)**		**26951**		**26908**	**64.4%**	**0.90 [0.79, 1.02]**
Total events	444		493			
Heterogeneity: Tau² = 0.00; Chi² = 2.47, df = 5 (P = 0.78); I² = 0%						
Test for overall effect: Z = 1.64 (P = 0.10)						
**Total (95% CI)**		**40786**		**40697**	**100.0%**	**1.05 [0.91, 1.23]**
Total events	689		670			
Heterogeneity: Tau² = 0.02; Chi² = 16.27, df = 9 (P = 0.06); I² = 45%						
Test for overall effect: Z = 0.69 (P = 0.49)						
Test for subgroup differences: Chi² = 13.34, df = 1 (P = 0.0003), I² = 92.5%						

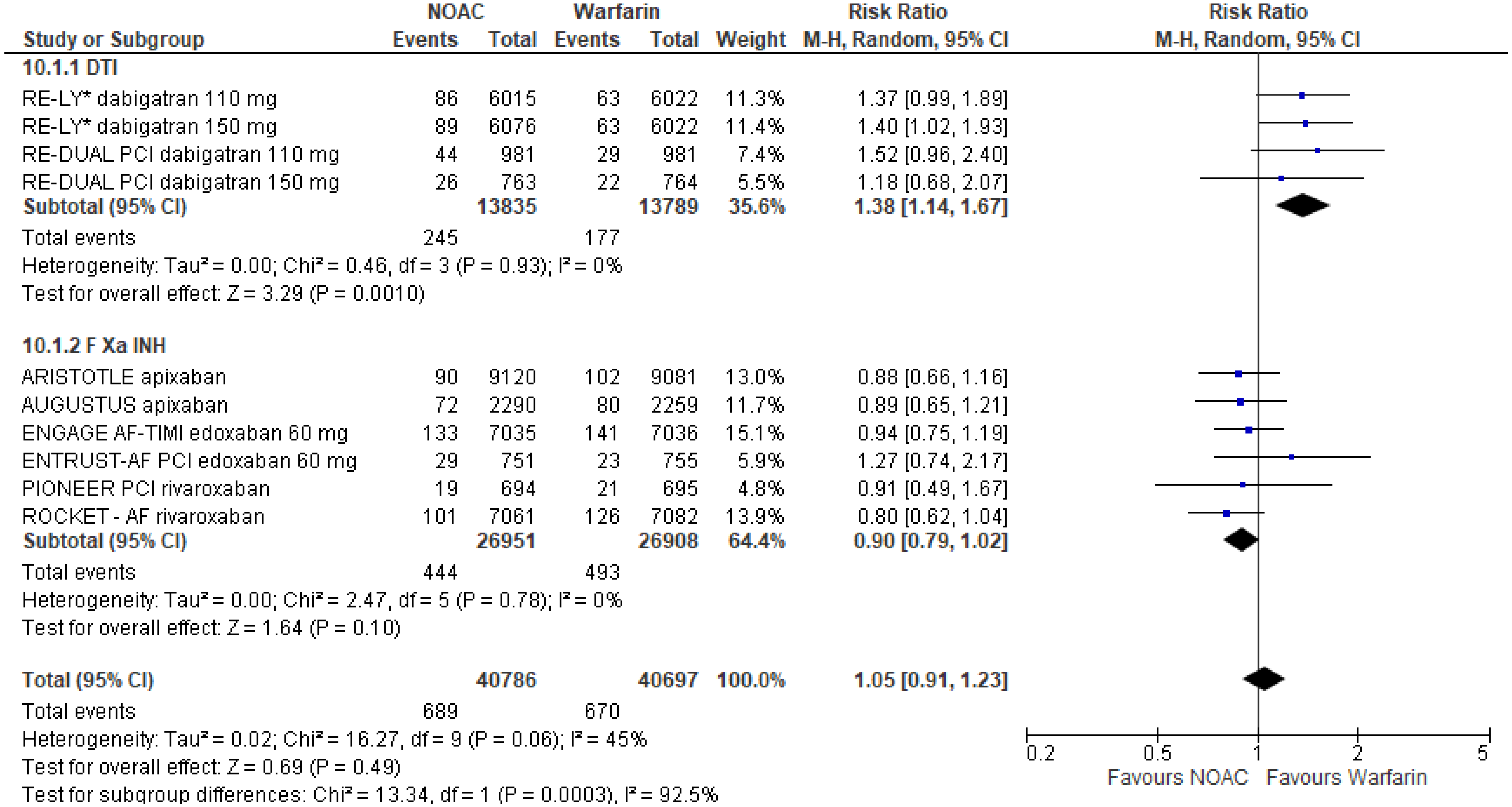

*- original data from RE-LY study [23]

Figure S20A. The meta-analysis results for MI after excluding ENGAGE AF-TIMI edoxaban 30 mg and combining study data with respect to doses.

Study or Subgroup	NOAC Events	NOAC Total	Warfarin Events	Warfarin Total	Weight	Risk Ratio M-H, Random, 95% CI
**29.1.1 DTI**						
RE-LY* dabigatran	175	12091	63	6022	15.1%	1.38 [1.04, 1.84]
RE-DUAL PCI dabigatran	70	1744	29	981	9.3%	1.36 [0.89, 2.08]
**Subtotal (95% CI)**		**13835**		**7003**	**24.3%**	**1.38 [1.08, 1.74]**
Total events	245		92			
Heterogeneity: Tau² = 0.00; Chi² = 0.01, df = 1 (P = 0.94); I² = 0%						
Test for overall effect: Z = 2.63 (P = 0.009)						
**29.1.2 F Xa INH**						
ARISTOTLE apixaban	90	9120	102	9081	15.3%	0.88 [0.66, 1.16]
AUGUSTUS apixaban	72	2290	80	2259	13.7%	0.89 [0.65, 1.21]
ENGAGE AF-TIMI edoxaban 60 mg	133	7035	141	7036	18.2%	0.94 [0.75, 1.19]
ENTRUST-AF PCI edoxaban 60 mg	29	751	23	755	6.5%	1.27 [0.74, 2.17]
PIONEER PCI rivaroxaban	19	694	21	695	5.3%	0.91 [0.49, 1.67]
ROCKET - AF rivaroxaban	101	7061	126	7082	16.6%	0.80 [0.62, 1.04]
**Subtotal (95% CI)**		**26951**		**26908**	**75.7%**	**0.90 [0.79, 1.02]**
Total events	444		493			
Heterogeneity: Tau² = 0.00; Chi² = 2.47, df = 5 (P = 0.78); I² = 0%						
Test for overall effect: Z = 1.64 (P = 0.10)						
**Total (95% CI)**		**40786**		**33911**	**100.0%**	**1.00 [0.86, 1.17]**
Total events	689		585			
Heterogeneity: Tau² = 0.02; Chi² = 12.05, df = 7 (P = 0.10); I² = 42%						
Test for overall effect: Z = 0.06 (P = 0.95)						
Test for subgroup differences: Chi² = 9.56, df = 1 (P = 0.002), I² = 89.5%						

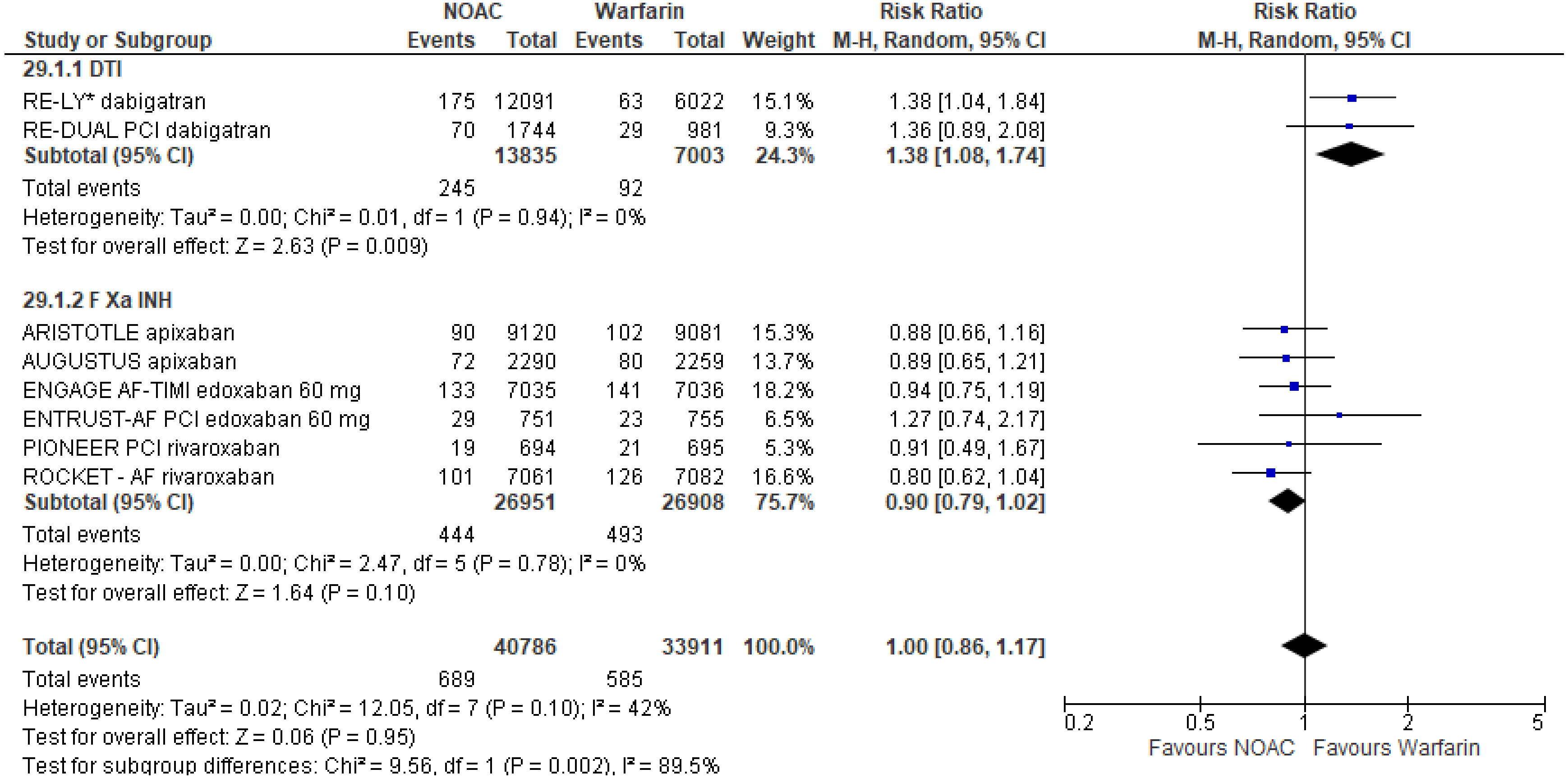

*- original data from RE-LY study [23]

Figure S21. The funnel plot for MI after excluding ENGAGE AF-TIMI edoxaban 30 mg.

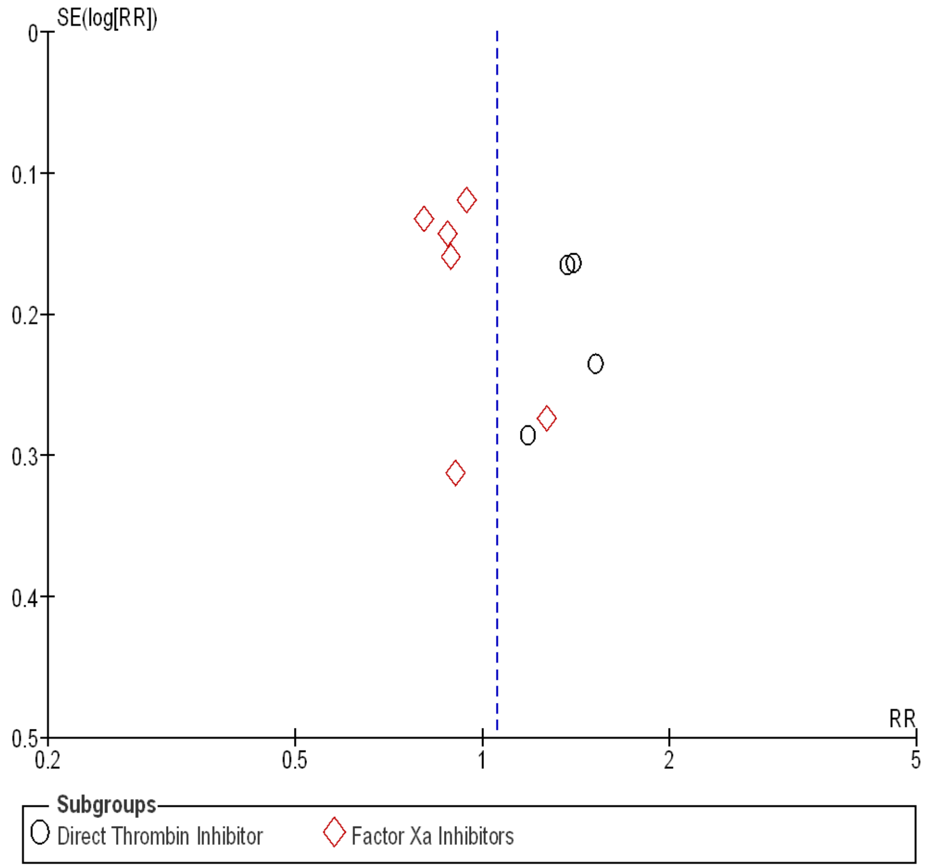

Figure S22. The meta-analysis results for MI – reanalysis RE-LY [27] -intention to treat data.

Study or Subgroup	NOAC Events	NOAC Total	Warfarin Events	Warfarin Total	Weight	Risk Ratio M-H, Random, 95% CI
**11.1.1 DTI**						
RE-LY** dabigatran 110 mg ITT	98	6015	75	6022	10.5%	1.31 [0.97, 1.76]
RE-LY** dabigatran 150 mg ITT	97	6076	75	6022	10.4%	1.28 [0.95, 1.73]
RE-DUAL PCI dabigatran 110 mg	44	981	29	981	5.7%	1.52 [0.96, 2.40]
RE-DUAL PCI dabigatran 150 mg	26	763	22	764	4.2%	1.18 [0.68, 2.07]
**Subtotal (95% CI)**		**13835**		**13789**	**30.8%**	**1.31 [1.10, 1.58]**
Total events	265		201			
Heterogeneity: Tau² = 0.00; Chi² = 0.54, df = 3 (P = 0.91); I² = 0%						
Test for overall effect: Z = 2.95 (P = 0.003)						
**11.1.2 F Xa INH**						
ARISTOTLE apixaban	90	9120	102	9081	11.2%	0.88 [0.66, 1.16]
AUGUSTUS apixaban	72	2290	80	2259	9.9%	0.89 [0.65, 1.21]
ENGAGE AF-TIMI edoxaban 30 mg	169	7034	141	7036	14.3%	1.20 [0.96, 1.50]
ENGAGE AF-TIMI edoxaban 60 mg	133	7035	141	7036	13.5%	0.94 [0.75, 1.19]
ENTRUST-AF PCI edoxaban 60 mg	29	751	23	755	4.5%	1.27 [0.74, 2.17]
PIONEER PCI rivaroxaban	19	694	21	695	3.6%	0.91 [0.49, 1.67]
ROCKET - AF rivaroxaban	101	7061	126	7082	12.2%	0.80 [0.62, 1.04]
**Subtotal (95% CI)**		**33985**		**33944**	**69.2%**	**0.96 [0.85, 1.09]**
Total events	613		634			
Heterogeneity: Tau² = 0.01; Chi² = 7.36, df = 6 (P = 0.29); I² = 19%						
Test for overall effect: Z = 0.60 (P = 0.55)						
**Total (95% CI)**		**47820**		**47733**	**100.0%**	**1.06 [0.93, 1.20]**
Total events	878		835			
Heterogeneity: Tau² = 0.02; Chi² = 16.00, df = 10 (P = 0.10); I² = 37%						
Test for overall effect: Z = 0.90 (P = 0.37)						
Test for subgroup differences: Chi² = 7.66, df = 1 (P = 0.006), I² = 86.9%						

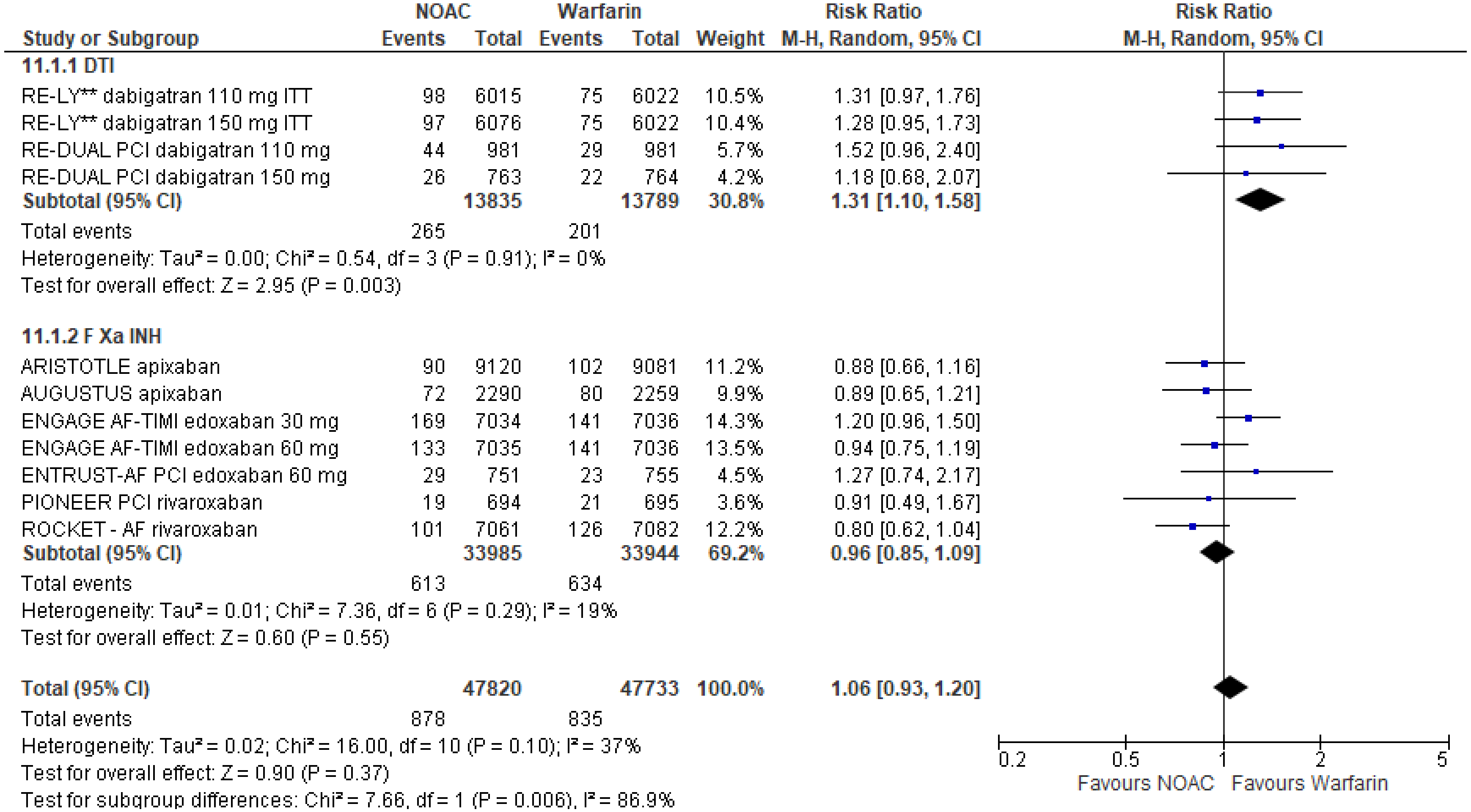

** - results from reanalysis of RE-LY study [27]

Figure S22A. The meta-analysis results for MI – reanalysis RE-LY [27] -intention to treat data after combining study data with respect to doses.

Study or Subgroup	NOAC Events	NOAC Total	Warfarin Events	Warfarin Total	Weight	Risk Ratio M-H, Random, 95% CI
**30.1.1 DTI**						
RE-LY** dabigatran ITT	195	12091	75	6022	15.9%	1.29 [0.99, 1.69]
RE-DUAL PCI dabigatran	70	1744	29	981	8.4%	1.36 [0.89, 2.08]
**Subtotal (95% CI)**		**13835**		**7003**	**24.3%**	**1.31 [1.05, 1.64]**
Total events	265		104			
Heterogeneity: Tau² = 0.00; Chi² = 0.03, df = 1 (P = 0.85); I² = 0%						
Test for overall effect: Z = 2.37 (P = 0.02)						
**30.1.2 F Xa INH**						
ARISTOTLE apixaban	90	9120	102	9081	14.8%	0.88 [0.66, 1.16]
AUGUSTUS apixaban	72	2290	80	2259	13.0%	0.89 [0.65, 1.21]
ENGAGE-AF-TIMI edoxaban	302	14069	141	7036	21.3%	1.07 [0.88, 1.31]
ENTRUST-AF PCI edoxaban 60 mg	29	751	23	755	5.7%	1.27 [0.74, 2.17]
PIONEER PCI rivaroxaban	19	694	21	695	4.6%	0.91 [0.49, 1.67]
ROCKET - AF rivaroxaban	101	7061	126	7082	16.3%	0.80 [0.62, 1.04]
**Subtotal (95% CI)**		**33985**		**26908**	**75.7%**	**0.95 [0.84, 1.07]**
Total events	613		493			
Heterogeneity: Tau² = 0.00; Chi² = 4.60, df = 5 (P = 0.47); I² = 0%						
Test for overall effect: Z = 0.89 (P = 0.38)						
**Total (95% CI)**		**47820**		**33911**	**100.0%**	**1.02 [0.89, 1.17]**
Total events	878		597			
Heterogeneity: Tau² = 0.01; Chi² = 10.93, df = 7 (P = 0.14); I² = 36%						
Test for overall effect: Z = 0.29 (P = 0.78)						
Test for subgroup differences: Chi² = 6.29, df = 1 (P = 0.01), I² = 84.1%						

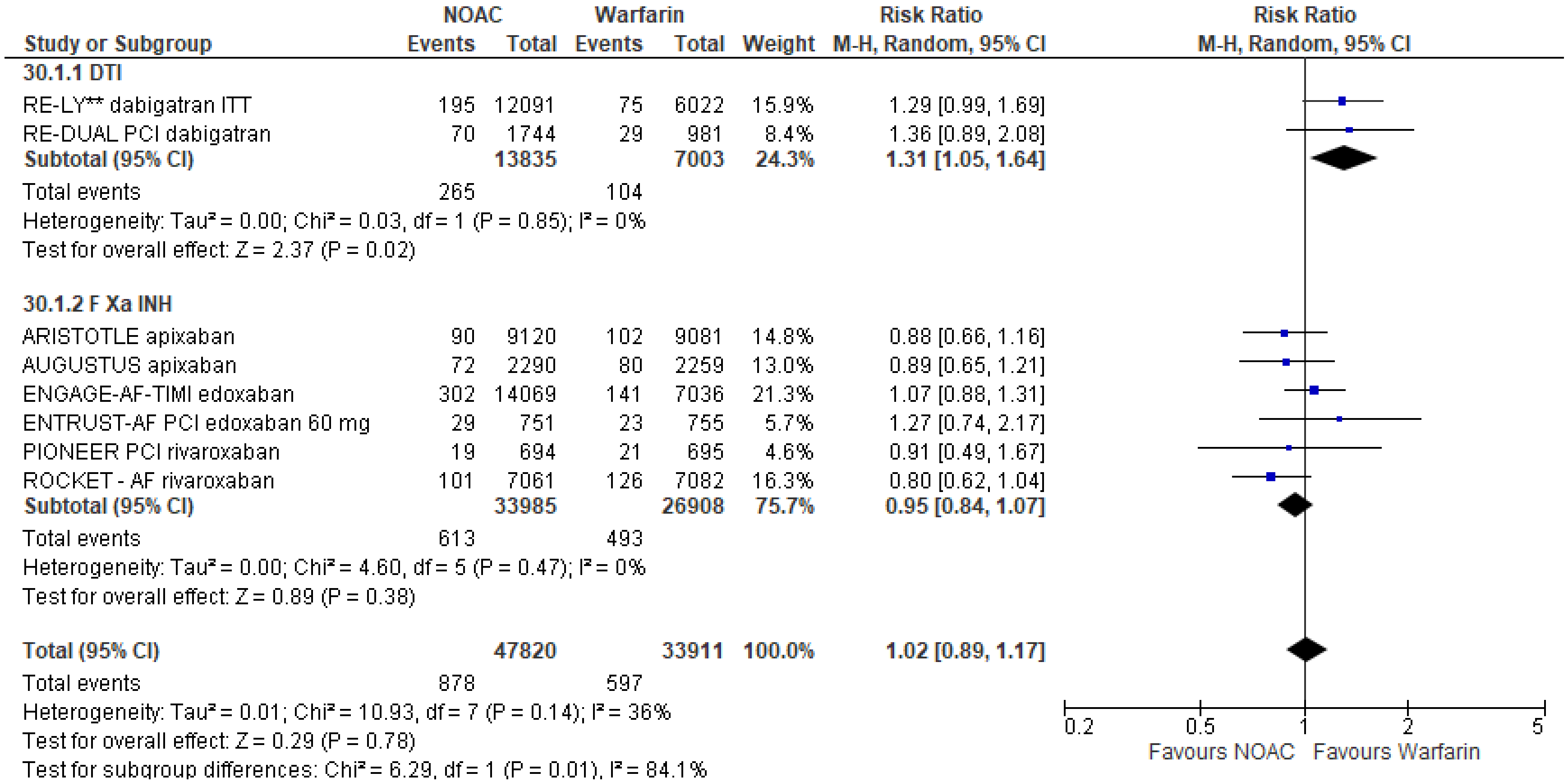

** - results from reanalysis of RE-LY study [27]

Figure S23. The funnel plot for MI – reanalysis RE-LY [27] -intention to treat data.

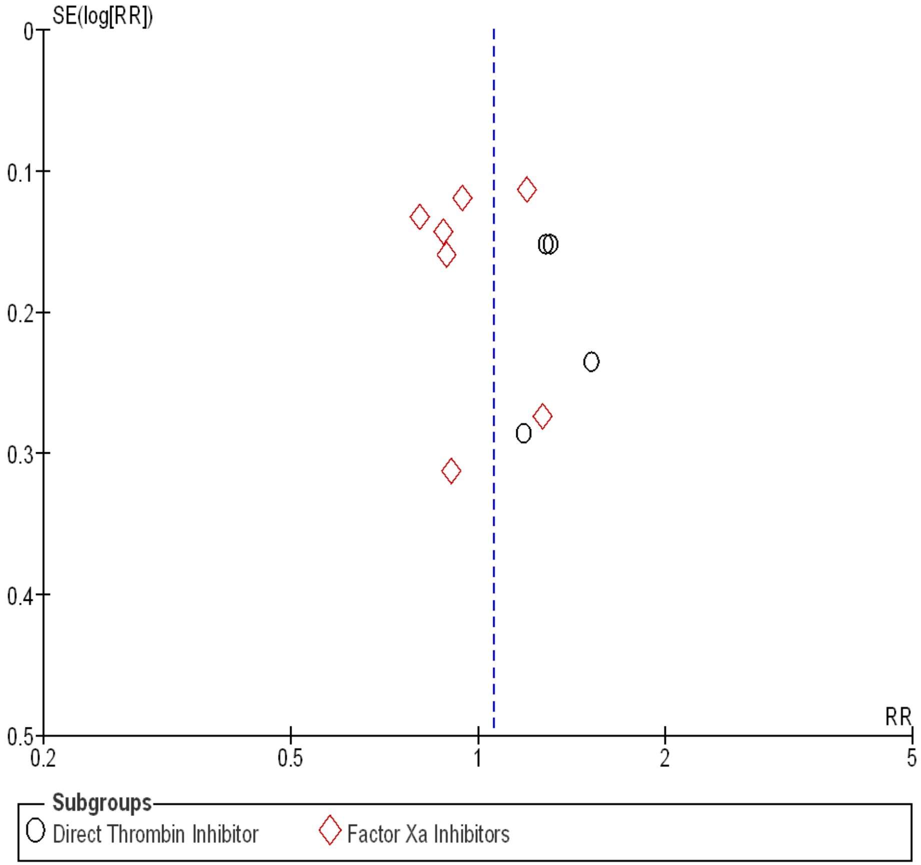

Figure S24. The meta-analysis results for MI – reanalysis RE-LY [27] - intention to treat data after excluding ENGAGE AF-TIMI edoxaban 30 mg.

Study or Subgroup	NOAC Events	NOAC Total	Warfarin Events	Warfarin Total	Weight	Risk Ratio M-H, Random, 95% CI
**12.1.1 DTI**						
RE-LY** dabigatran 110 mg ITT	98	6015	75	6022	12.2%	1.31 [0.97, 1.76]
RE-LY** dabigatran 150 mg ITT	97	6076	75	6022	12.1%	1.28 [0.95, 1.73]
RE-DUAL PCI dabigatran 110 mg	44	981	29	981	6.8%	1.52 [0.96, 2.40]
RE-DUAL PCI dabigatran 150 mg	26	763	22	764	5.0%	1.18 [0.68, 2.07]
**Subtotal (95% CI)**		**13835**		**13789**	**36.1%**	**1.31 [1.10, 1.58]**
Total events	265		201			
Heterogeneity: Tau² = 0.00; Chi² = 0.54, df = 3 (P = 0.91); I² = 0%						
Test for overall effect: Z = 2.95 (P = 0.003)						
**12.1.2 F Xa INH**						
ARISTOTLE apixaban	90	9120	102	9081	13.0%	0.88 [0.66, 1.16]
AUGUSTUS apixaban	72	2290	80	2259	11.5%	0.89 [0.65, 1.21]
ENGAGE AF-TIMI edoxaban 60 mg	133	7035	141	7036	15.6%	0.94 [0.75, 1.19]
ENTRUST-AF PCI edoxaban 60 mg	29	751	23	755	5.3%	1.27 [0.74, 2.17]
PIONEER PCI rivaroxaban	19	694	21	695	4.3%	0.91 [0.49, 1.67]
ROCKET - AF rivaroxaban	101	7061	126	7082	14.2%	0.80 [0.62, 1.04]
**Subtotal (95% CI)**		**26951**		**26908**	**63.9%**	**0.90 [0.79, 1.02]**
Total events	444		493			
Heterogeneity: Tau² = 0.00; Chi² = 2.47, df = 5 (P = 0.78); I² = 0%						
Test for overall effect: Z = 1.64 (P = 0.10)						
**Total (95% CI)**		**40786**		**40697**	**100.0%**	**1.04 [0.90, 1.19]**
Total events	709		694			
Heterogeneity: Tau² = 0.02; Chi² = 14.29, df = 9 (P = 0.11); I² = 37%						
Test for overall effect: Z = 0.53 (P = 0.59)						
Test for subgroup differences: Chi² = 11.28, df = 1 (P = 0.0008), I² = 91.1%						

Risk Ratio
M-H, Random, 95% CI

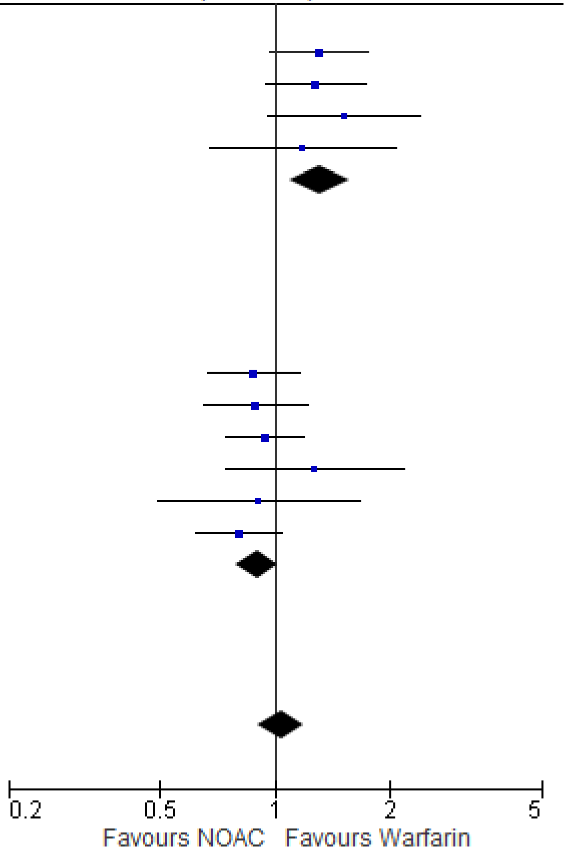

** - results from reanalysis of RE-LY study [27]

Figure S24A. The meta-analysis results for MI – reanalysis RE-LY [27] - intention to treat data after excluding ENGAGE AF-TIMI edoxaban 30 mg and combining study data with respect to doses.

Study or Subgroup	NOAC Events	NOAC Total	Warfarin Events	Warfarin Total	Weight	Risk Ratio M-H, Random, 95% CI
**31.1.1 DTI**						
RE-LY** dabigatran ITT	195	12091	75	6022	16.4%	1.29 [0.99, 1.69]
RE-DUAL PCI dabigatran	70	1744	29	981	8.7%	1.36 [0.89, 2.08]
**Subtotal (95% CI)**		**13835**		**7003**	**25.1%**	**1.31 [1.05, 1.64]**
Total events	265		104			
Heterogeneity: Tau² = 0.00; Chi² = 0.03, df = 1 (P = 0.85); I² = 0%						
Test for overall effect: Z = 2.37 (P = 0.02)						
**31.1.2 F Xa INH**						
ARISTOTLE apixaban	90	9120	102	9081	15.3%	0.88 [0.66, 1.16]
AUGUSTUS apixaban	72	2290	80	2259	13.4%	0.89 [0.65, 1.21]
ENGAGE AF-TIMI edoxaban 60 mg	133	7035	141	7036	18.7%	0.94 [0.75, 1.19]
ENTRUST-AF PCI edoxaban 60 mg	29	751	23	755	6.0%	1.27 [0.74, 2.17]
PIONEER PCI rivaroxaban	19	694	21	695	4.8%	0.91 [0.49, 1.67]
ROCKET - AF rivaroxaban	101	7061	126	7082	16.8%	0.80 [0.62, 1.04]
**Subtotal (95% CI)**		**26951**		**26908**	**74.9%**	**0.90 [0.79, 1.02]**
Total events	444		493			
Heterogeneity: Tau² = 0.00; Chi² = 2.47, df = 5 (P = 0.78); I² = 0%						
Test for overall effect: Z = 1.64 (P = 0.10)						
**Total (95% CI)**		**40786**		**33911**	**100.0%**	**1.00 [0.86, 1.15]**
Total events	709		597			
Heterogeneity: Tau² = 0.01; Chi² = 10.75, df = 7 (P = 0.15); I² = 35%						
Test for overall effect: Z = 0.07 (P = 0.95)						
Test for subgroup differences: Chi² = 8.24, df = 1 (P = 0.004), I² = 87.9%						

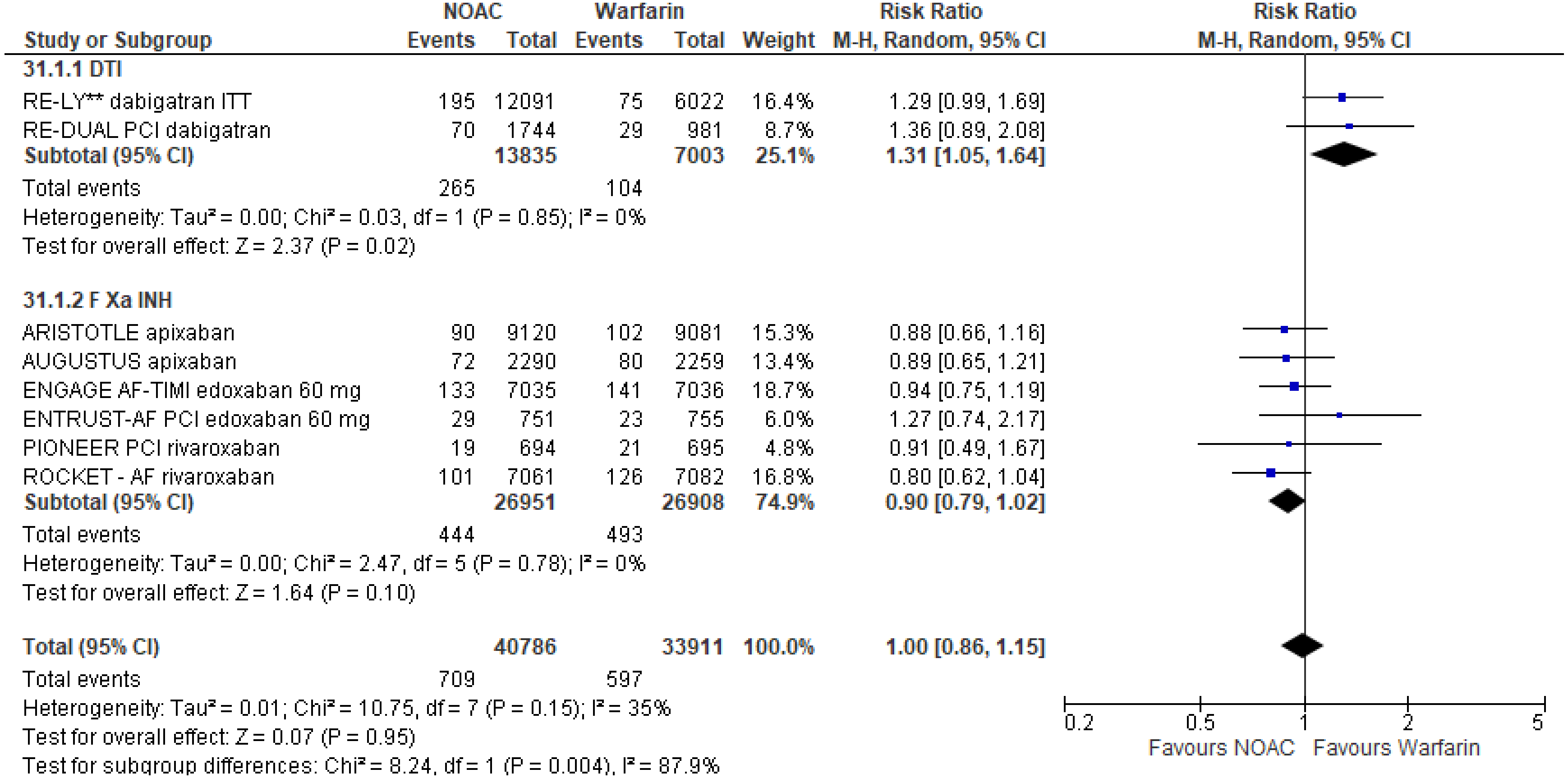

Figure S25. The funnel plot for MI – reanalysis RE-LY [27] -intention to treat data after excluding ENGAGE AF-TIMI edoxaban 30 mg.

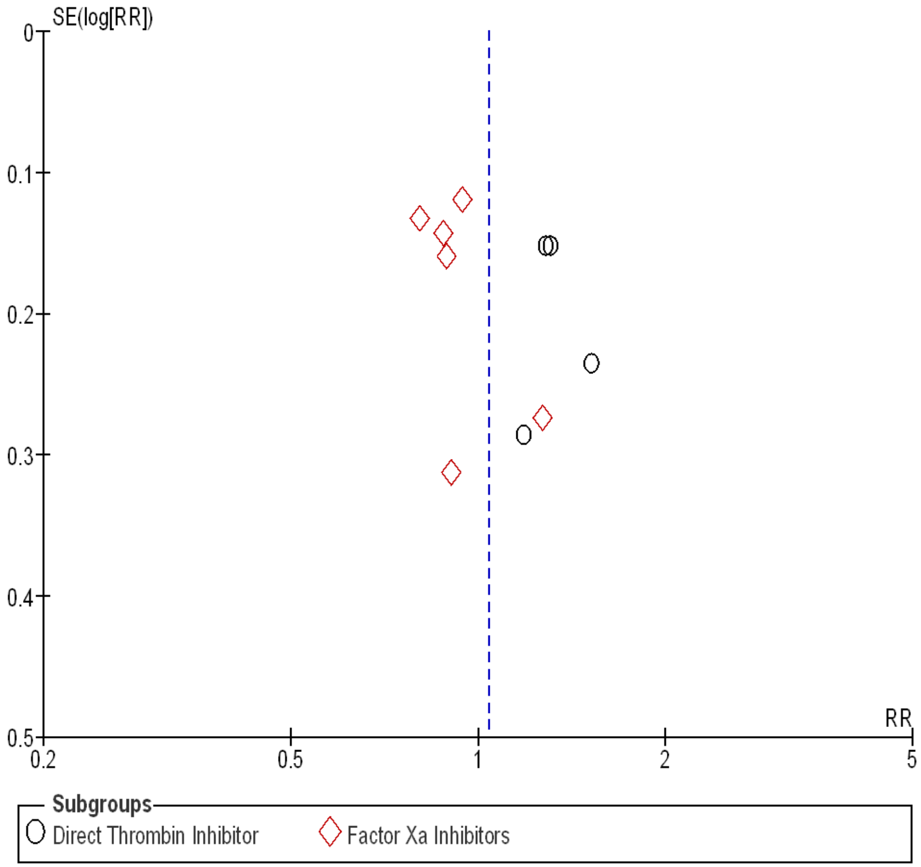

Figure S26. The meta-analysis results for MI – reanalysis RE-LY [27] - on treatment data.

Study or Subgroup	NOAC Events	NOAC Total	Warfarin Events	Warfarin Total	Weight	Risk Ratio M-H, Random, 95% CI
**13.1.1 DTI**						
RE-LY** dabigatran 110mg on-T	80	5983	63	5998	9.4%	1.27 [0.92, 1.77]
RE-LY** dabigatran 150mg on-T	79	6059	63	5998	9.4%	1.24 [0.89, 1.73]
RE-DUAL PCI dabigatran 110 mg	44	981	29	981	5.6%	1.52 [0.96, 2.40]
RE-DUAL PCI dabigatran 150 mg	26	763	22	764	4.0%	1.18 [0.68, 2.07]
**Subtotal (95% CI)**		**13786**		**13741**	**28.4%**	**1.29 [1.06, 1.57]**
Total events	229		177			
Heterogeneity: Tau² = 0.00; Chi² = 0.63, df = 3 (P = 0.89); I² = 0%						
Test for overall effect: Z = 2.57 (P = 0.01)						
**13.1.2 F Xa INH**						
ARISTOTLE apixaban	90	9120	102	9081	11.5%	0.88 [0.66, 1.16]
AUGUSTUS apixaban	72	2290	80	2259	10.0%	0.89 [0.65, 1.21]
ENGAGE AF-TIMI edoxaban 30 mg	169	7034	141	7036	15.2%	1.20 [0.96, 1.50]
ENGAGE AF-TIMI edoxaban 60 mg	133	7035	141	7036	14.3%	0.94 [0.75, 1.19]
ENTRUST-AF PCI edoxaban 60 mg	29	751	23	755	4.3%	1.27 [0.74, 2.17]
PIONEER PCI rivaroxaban	19	694	21	695	3.5%	0.91 [0.49, 1.67]
ROCKET - AF rivaroxaban	101	7061	126	7082	12.7%	0.80 [0.62, 1.04]
**Subtotal (95% CI)**		**33985**		**33944**	**71.6%**	**0.96 [0.85, 1.09]**
Total events	613		634			
Heterogeneity: Tau² = 0.01; Chi² = 7.36, df = 6 (P = 0.29); I² = 19%						
Test for overall effect: Z = 0.60 (P = 0.55)						
**Total (95% CI)**		**47771**		**47685**	**100.0%**	**1.05 [0.93, 1.18]**
Total events	842		811			
Heterogeneity: Tau² = 0.01; Chi² = 14.45, df = 10 (P = 0.15); I² = 31%						
Test for overall effect: Z = 0.73 (P = 0.47)						
Test for subgroup differences: Chi² = 6.17, df = 1 (P = 0.01), I² = 83.8%						

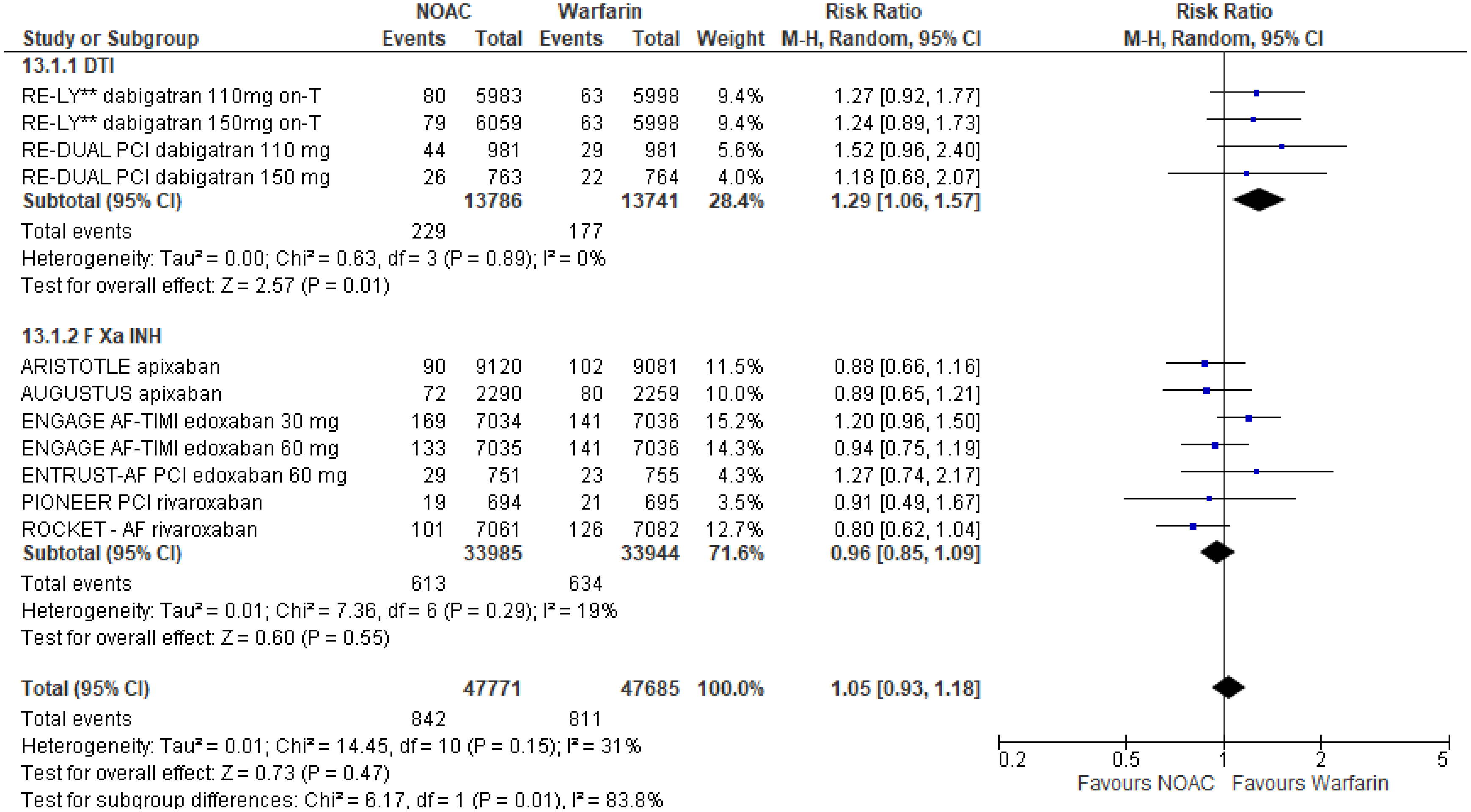

** - results from reanalysis of RE-LY study [27]

Figure S26A. The meta-analysis results for MI – reanalysis RE-LY [27] - on treatment data after combining study data with respect to doses.

Study or Subgroup	NOAC Events	NOAC Total	Warfarin Events	Warfarin Total	Weight	Risk Ratio M-H, Random, 95% CI
**32.1.1 DTI**						
RE-LY** dabigatran on-T	159	12042	63	5998	14.5%	1.26 [0.94, 1.68]
RE-DUAL PCI dabigatran	70	1744	29	981	8.1%	1.36 [0.89, 2.08]
**Subtotal (95% CI)**		**13786**		**6979**	**22.6%**	**1.29 [1.01, 1.64]**
Total events	229		92			
Heterogeneity: Tau² = 0.00; Chi² = 0.09, df = 1 (P = 0.77); I² = 0%						
Test for overall effect: Z = 2.07 (P = 0.04)						
**32.1.2 F Xa INH**						
ARISTOTLE apixaban	90	9120	102	9081	15.1%	0.88 [0.66, 1.16]
AUGUSTUS apixaban	72	2290	80	2259	13.0%	0.89 [0.65, 1.21]
ENGAGE-AF-TIMI edoxaban	302	14069	141	7036	22.9%	1.07 [0.88, 1.31]
ENTRUST-AF PCI edoxaban 60 mg	29	751	23	755	5.4%	1.27 [0.74, 2.17]
PIONEER PCI rivaroxaban	19	694	21	695	4.3%	0.91 [0.49, 1.67]
ROCKET - AF rivaroxaban	101	7061	126	7082	16.8%	0.80 [0.62, 1.04]
**Subtotal (95% CI)**		**33985**		**26908**	**77.4%**	**0.95 [0.84, 1.07]**
Total events	613		493			
Heterogeneity: Tau² = 0.00; Chi² = 4.60, df = 5 (P = 0.47); I² = 0%						
Test for overall effect: Z = 0.89 (P = 0.38)						
**Total (95% CI)**		**47771**		**33887**	**100.0%**	**1.01 [0.88, 1.15]**
Total events	842		585			
Heterogeneity: Tau² = 0.01; Chi² = 9.74, df = 7 (P = 0.20); I² = 28%						
Test for overall effect: Z = 0.16 (P = 0.88)						
Test for subgroup differences: Chi² = 5.05, df = 1 (P = 0.02), I² = 80.2%						

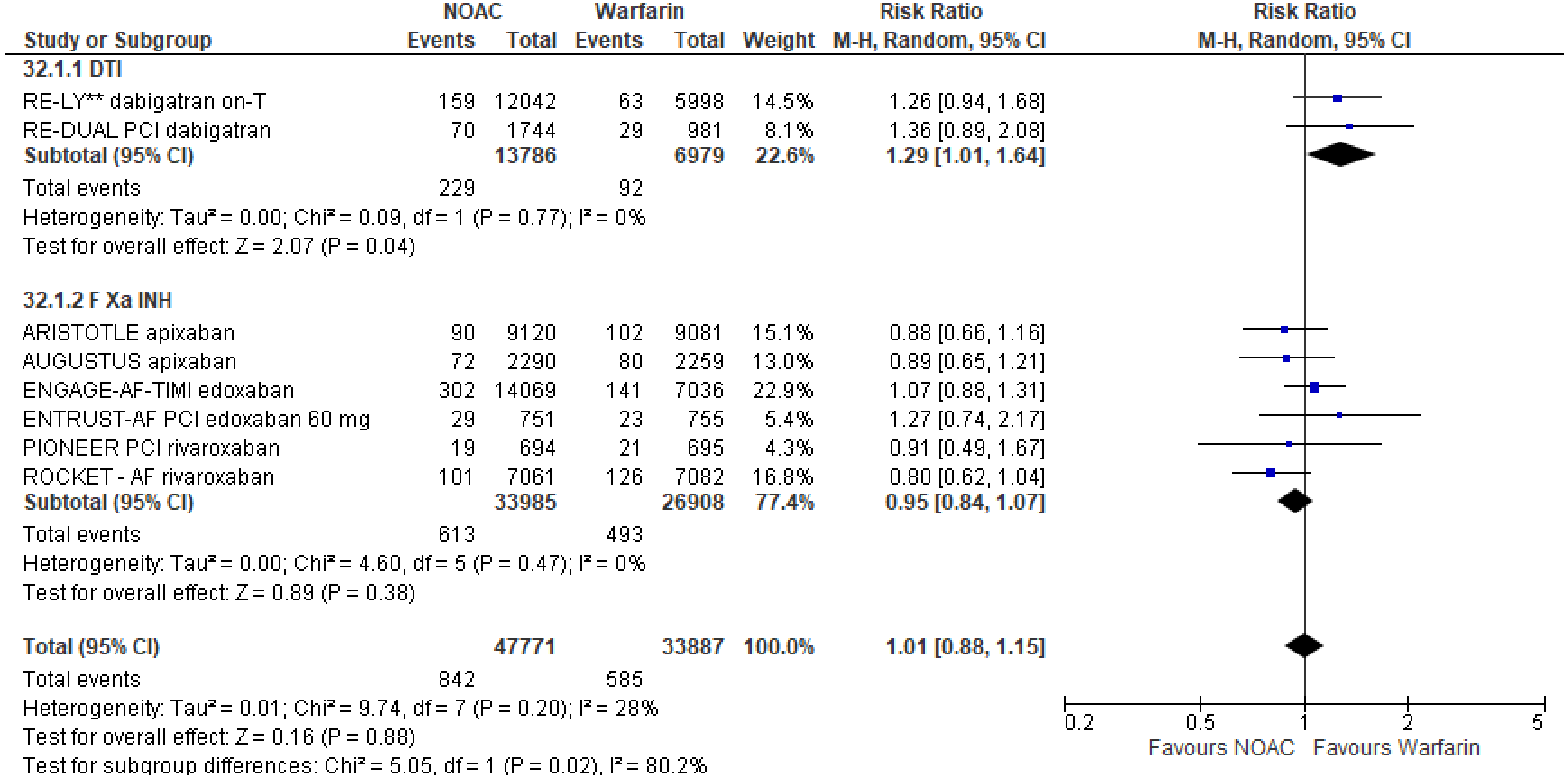

** - results from reanalysis of RE-LY study [27]

Figure S27. The funnel plot for MI – reanalysis RE-LY [27] - on treatment data.

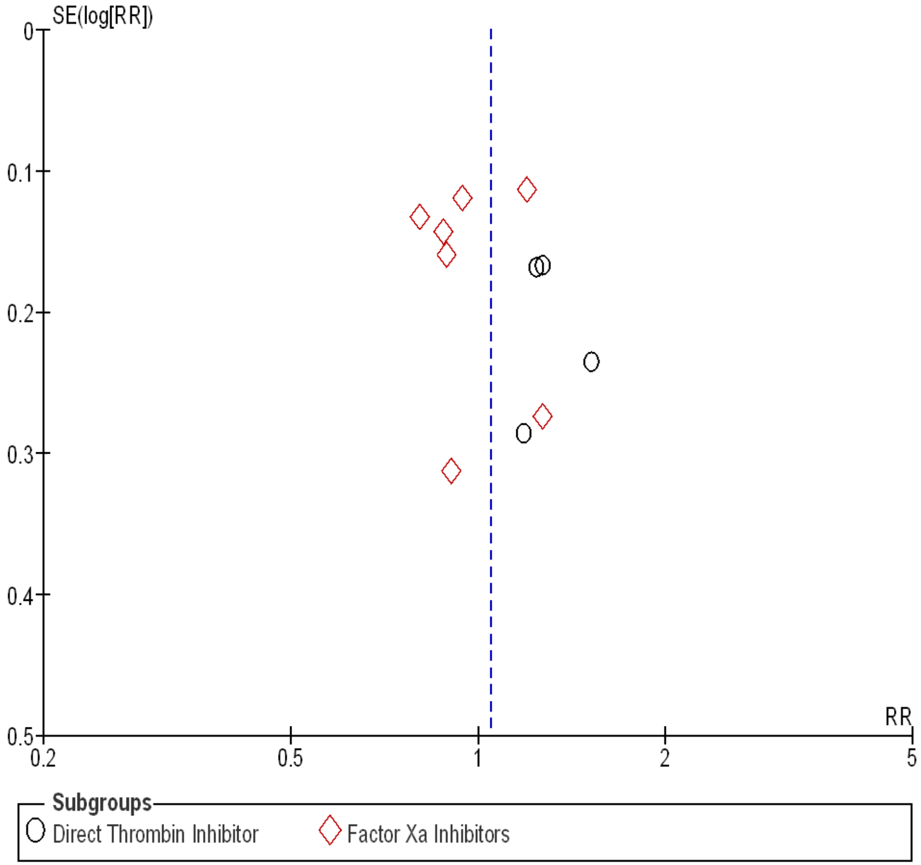

Figure S28. The meta-analysis results for MI – reanalysis RE-LY [27] - on treatment data after excluding ENGAGE AF-TIMI edoxaban 30 mg.

Study or Subgroup	NOAC Events	NOAC Total	Warfarin Events	Warfarin Total	Weight	Risk Ratio M-H, Random, 95% CI
**14.1.1 DTI**						
RE-LY** dabigatran 110mg on-T	80	5983	63	5998	11.1%	1.27 [0.92, 1.77]
RE-LY** dabigatran 150mg on-T	79	6059	63	5998	11.0%	1.24 [0.89, 1.73]
RE-DUAL PCI dabigatran 110 mg	44	981	29	981	6.6%	1.52 [0.96, 2.40]
RE-DUAL PCI dabigatran 150 mg	26	763	22	764	4.7%	1.18 [0.68, 2.07]
**Subtotal (95% CI)**		**13786**		**13741**	**33.4%**	**1.29 [1.06, 1.57]**
Total events	229		177			
Heterogeneity: Tau² = 0.00; Chi² = 0.63, df = 3 (P = 0.89); I² = 0%						
Test for overall effect: Z = 2.57 (P = 0.01)						
**14.1.2 F Xa INH**						
ARISTOTLE apixaban	90	9120	102	9081	13.6%	0.88 [0.66, 1.16]
AUGUSTUS apixaban	72	2290	80	2259	11.8%	0.89 [0.65, 1.21]
ENGAGE AF-TIMI edoxaban 60 mg	133	7035	141	7036	17.0%	0.94 [0.75, 1.19]
ENTRUST-AF PCI edoxaban 60 mg	29	751	23	755	5.1%	1.27 [0.74, 2.17]
PIONEER PCI rivaroxaban	19	694	21	695	4.0%	0.91 [0.49, 1.67]
ROCKET - AF rivaroxaban	101	7061	126	7082	15.1%	0.80 [0.62, 1.04]
**Subtotal (95% CI)**		**26951**		**26908**	**66.6%**	**0.90 [0.79, 1.02]**
Total events	444		493			
Heterogeneity: Tau² = 0.00; Chi² = 2.47, df = 5 (P = 0.78); I² = 0%						
Test for overall effect: Z = 1.64 (P = 0.10)						
**Total (95% CI)**		**40737**		**40649**	**100.0%**	**1.02 [0.90, 1.16]**
Total events	673		670			
Heterogeneity: Tau² = 0.01; Chi² = 12.39, df = 9 (P = 0.19); I² = 27%						
Test for overall effect: Z = 0.29 (P = 0.77)						
Test for subgroup differences: Chi² = 9.29, df = 1 (P = 0.002), I² = 89.2%						

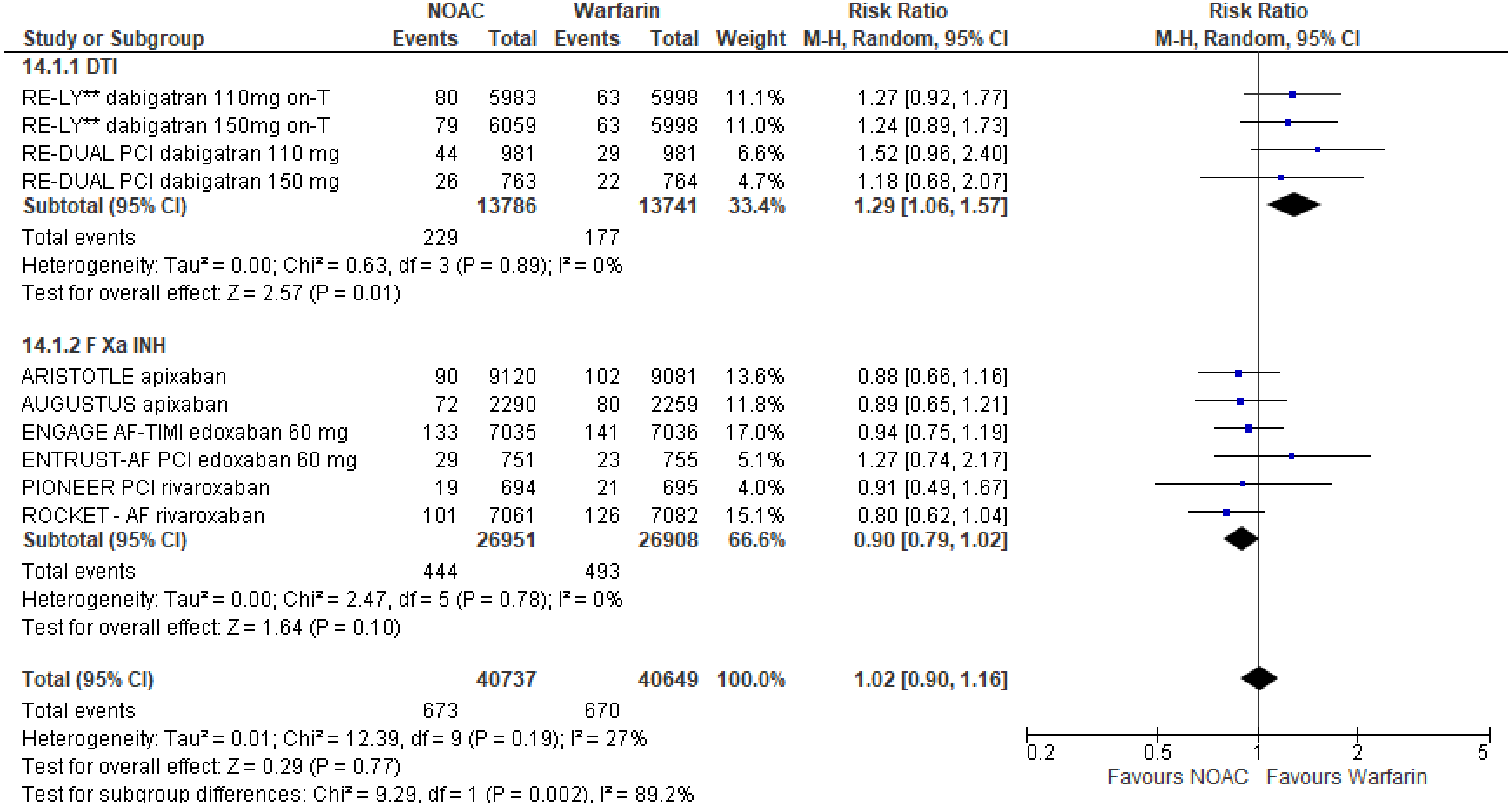

** - results from reanalysis of RE-LY study [27]

Figure S28A. The meta-analysis results for MI – reanalysis RE-LY [27] - on treatment data after excluding ENGAGE AF-TIMI edoxaban 30 mg and combining study data with respect to doses.

Study or Subgroup	NOAC Events	NOAC Total	Warfarin Events	Warfarin Total	Weight	Risk Ratio M-H, Random, 95% CI
**33.1.1 DTI**						
RE-LY** dabigatran on-T	159	12042	63	5998	15.1%	1.26 [0.94, 1.68]
RE-DUAL PCI dabigatran	70	1744	29	981	8.3%	1.36 [0.89, 2.08]
**Subtotal (95% CI)**		**13786**		**6979**	**23.4%**	**1.29 [1.01, 1.64]**
Total events	229		92			
Heterogeneity: Tau² = 0.00; Chi² = 0.09, df = 1 (P = 0.77); I² = 0%						
Test for overall effect: Z = 2.07 (P = 0.04)						
**33.1.2 F Xa INH**						
ARISTOTLE apixaban	90	9120	102	9081	15.7%	0.88 [0.66, 1.16]
AUGUSTUS apixaban	72	2290	80	2259	13.5%	0.89 [0.65, 1.21]
ENGAGE AF-TIMI edoxaban 60 mg	133	7035	141	7036	20.0%	0.94 [0.75, 1.19]
ENTRUST-AF PCI edoxaban 60 mg	29	751	23	755	5.5%	1.27 [0.74, 2.17]
PIONEER PCI rivaroxaban	19	694	21	695	4.4%	0.91 [0.49, 1.67]
ROCKET - AF rivaroxaban	101	7061	126	7082	17.5%	0.80 [0.62, 1.04]
**Subtotal (95% CI)**		**26951**		**26908**	**76.6%**	**0.90 [0.79, 1.02]**
Total events	444		493			
Heterogeneity: Tau² = 0.00; Chi² = 2.47, df = 5 (P = 0.78); I² = 0%						
Test for overall effect: Z = 1.64 (P = 0.10)						
**Total (95% CI)**		**40737**		**33887**	**100.0%**	**0.98 [0.86, 1.12]**
Total events	673		585			
Heterogeneity: Tau² = 0.01; Chi² = 9.30, df = 7 (P = 0.23); I² = 25%						
Test for overall effect: Z = 0.26 (P = 0.80)						
Test for subgroup differences: Chi² = 6.74, df = 1 (P = 0.009), I² = 85.2%						

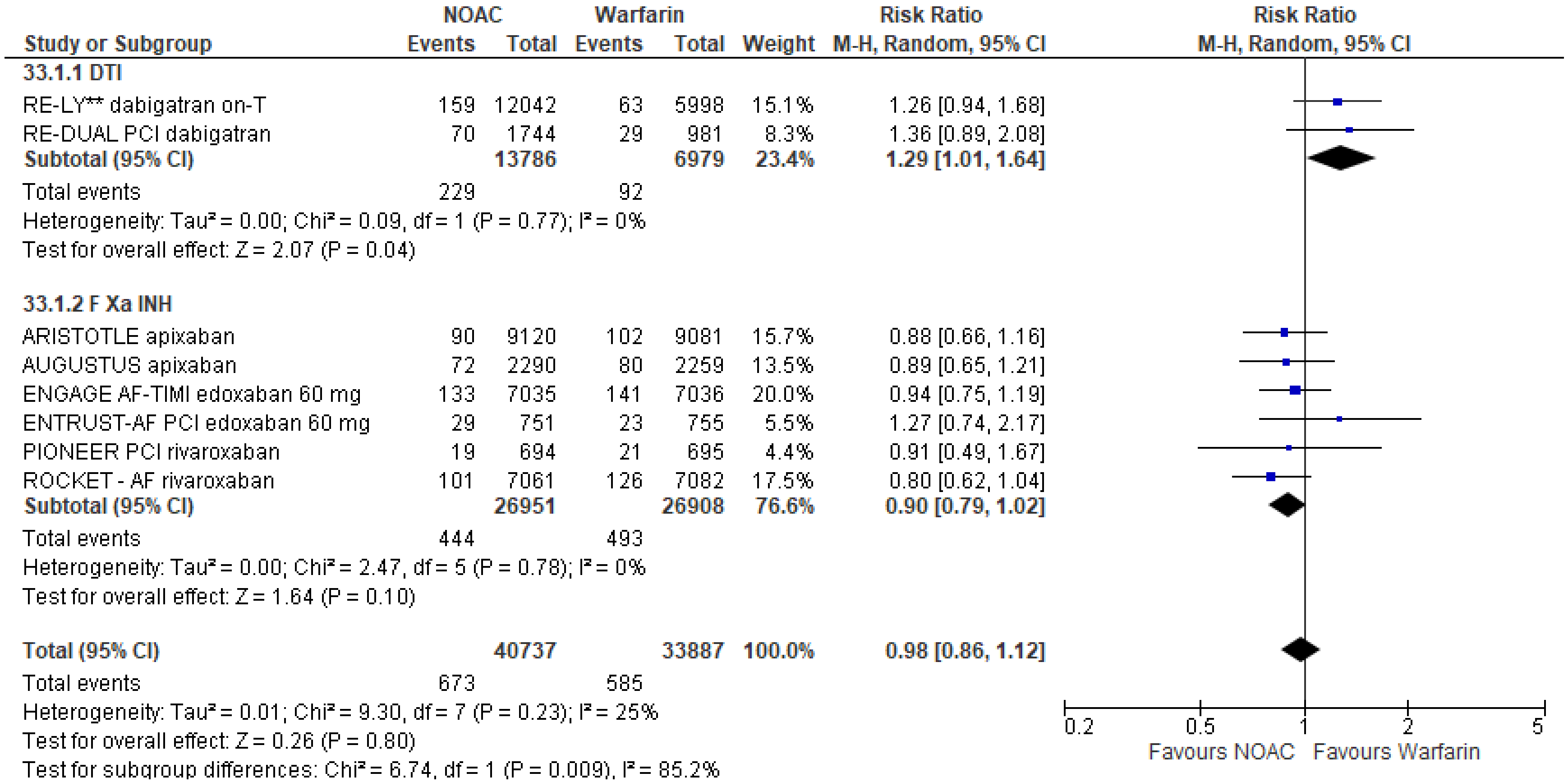

** - results from reanalysis of RE-LY study [27]

Figure S29. The funnel plot for MI – reanalysis RE-LY [27] - on treatment data after excluding ENGAGE AF-TIMI edoxaban 30 mg.

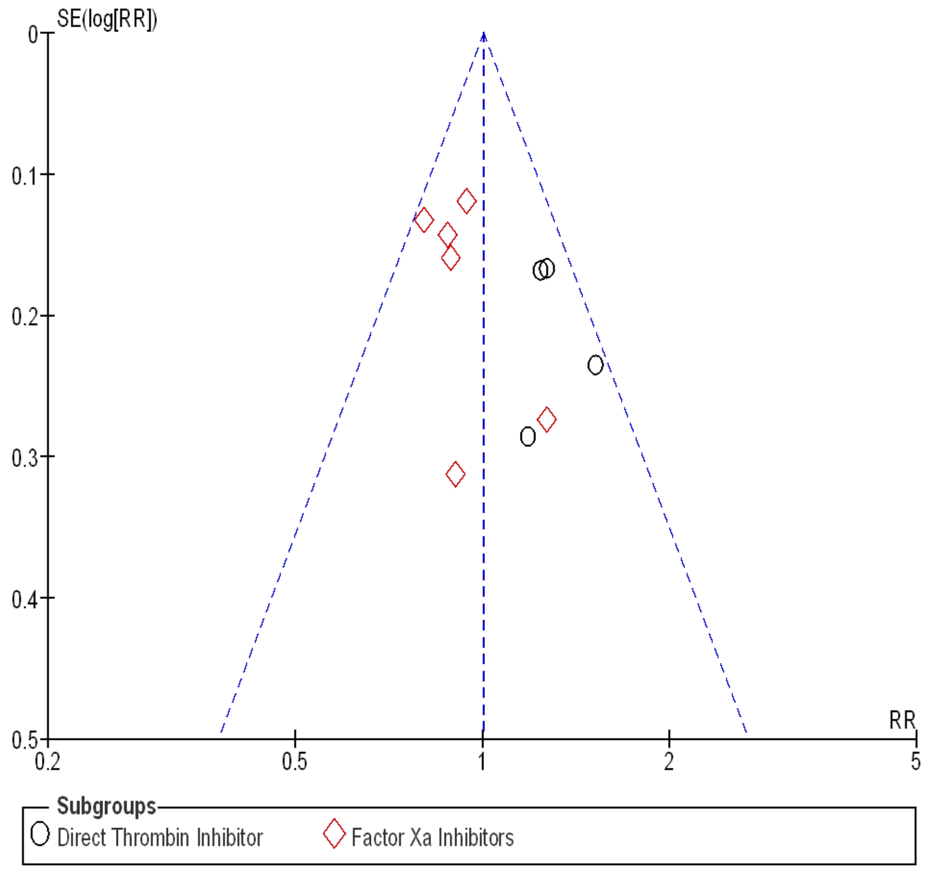

Figure S30. The meta-analysis results for stent thrombosis.

Study or Subgroup	NOAC Events	NOAC Total	Warfarin Events	Warfarin Total	Weight	Risk Ratio M-H, Random, 95% CI
**15.1.1 DTI**						
RE-DUAL PCI dabigatran 110 mg	15	981	8	981	23.5%	1.88 [0.80, 4.40]
RE-DUAL PCI dabigatran 150 mg	7	763	7	764	15.7%	1.00 [0.35, 2.84]
**Subtotal (95% CI)**		**1744**		**1745**	**39.3%**	**1.46 [0.75, 2.82]**
Total events	22		15			
Heterogeneity: Tau² = 0.00; Chi² = 0.83, df = 1 (P = 0.36); I² = 0%						
Test for overall effect: Z = 1.12 (P = 0.26)						
**15.1.2 F Xa INH**						
AUGUSTUS apixaban	14	2290	18	2259	35.4%	0.77 [0.38, 1.54]
ENTRUST-AF PCI edoxaban 60 mg	8	751	6	755	15.4%	1.34 [0.47, 3.84]
PIONEER PCI rivaroxaban	5	694	4	695	10.0%	1.25 [0.34, 4.64]
**Subtotal (95% CI)**		**3735**		**3709**	**60.7%**	**0.96 [0.56, 1.63]**
Total events	27		28			
Heterogeneity: Tau² = 0.00; Chi² = 0.94, df = 2 (P = 0.62); I² = 0%						
Test for overall effect: Z = 0.16 (P = 0.87)						
**Total (95% CI)**		**5479**		**5454**	**100.0%**	**1.13 [0.75, 1.71]**
Total events	49		43			
Heterogeneity: Tau² = 0.00; Chi² = 2.72, df = 4 (P = 0.61); I² = 0%						
Test for overall effect: Z = 0.58 (P = 0.56)						
Test for subgroup differences: Chi² = 0.94, df = 1 (P = 0.33), I² = 0%						

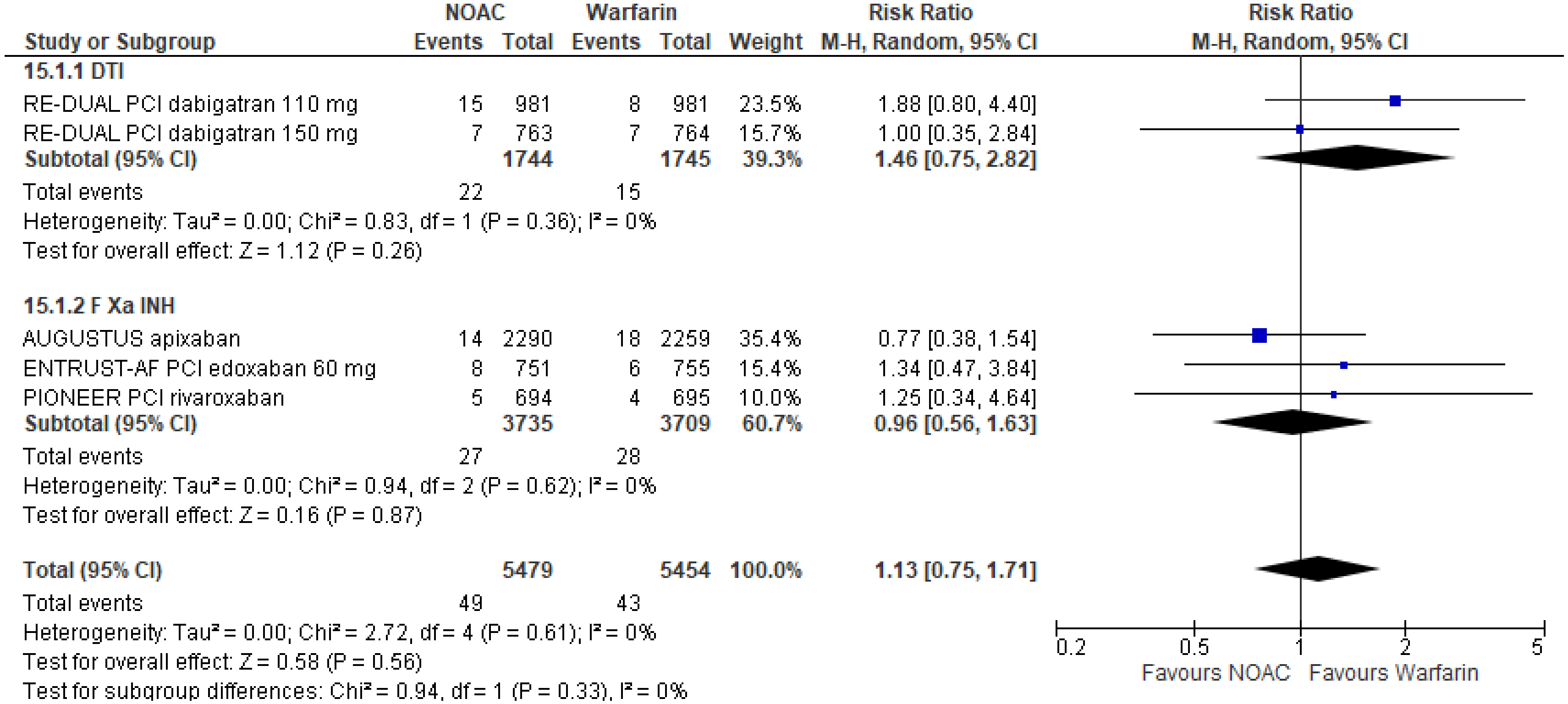

Figure S30A. The meta-analysis results for stent thrombosis after combining study data with respect to doses.

Study or Subgroup	NOAC Events	NOAC Total	Warfarin Events	Warfarin Total	Weight	Risk Ratio M-H, Random, 95% CI
**42.1.1 DTI**						
RE-DUAL PCI dabigatran	22	1744	8	981	30.3%	1.55 [0.69, 3.46]
**Subtotal (95% CI)**		**1744**		**981**	**30.3%**	**1.55 [0.69, 3.46]**
Total events	22		8			
Heterogeneity: Not applicable						
Test for overall effect: Z = 1.06 (P = 0.29)						
**42.1.2 F Xa INH**						
AUGUSTUS apixaban	14	2290	18	2259	40.6%	0.77 [0.38, 1.54]
ENTRUST-AF PCI edoxaban 60 mg	8	751	6	755	17.7%	1.34 [0.47, 3.84]
PIONEER PCI rivaroxaban	5	694	4	695	11.4%	1.25 [0.34, 4.64]
**Subtotal (95% CI)**		**3735**		**3709**	**69.7%**	**0.96 [0.56, 1.63]**
Total events	27		28			
Heterogeneity: Tau² = 0.00; Chi² = 0.94, df = 2 (P = 0.62); I² = 0%						
Test for overall effect: Z = 0.16 (P = 0.87)						
**Total (95% CI)**		**5479**		**4690**	**100.0%**	**1.11 [0.71, 1.73]**
Total events	49		36			
Heterogeneity: Tau² = 0.00; Chi² = 1.89, df = 3 (P = 0.60); I² = 0%						
Test for overall effect: Z = 0.45 (P = 0.65)						
Test for subgroup differences: Chi² = 0.95, df = 1 (P = 0.33), I² = 0%						

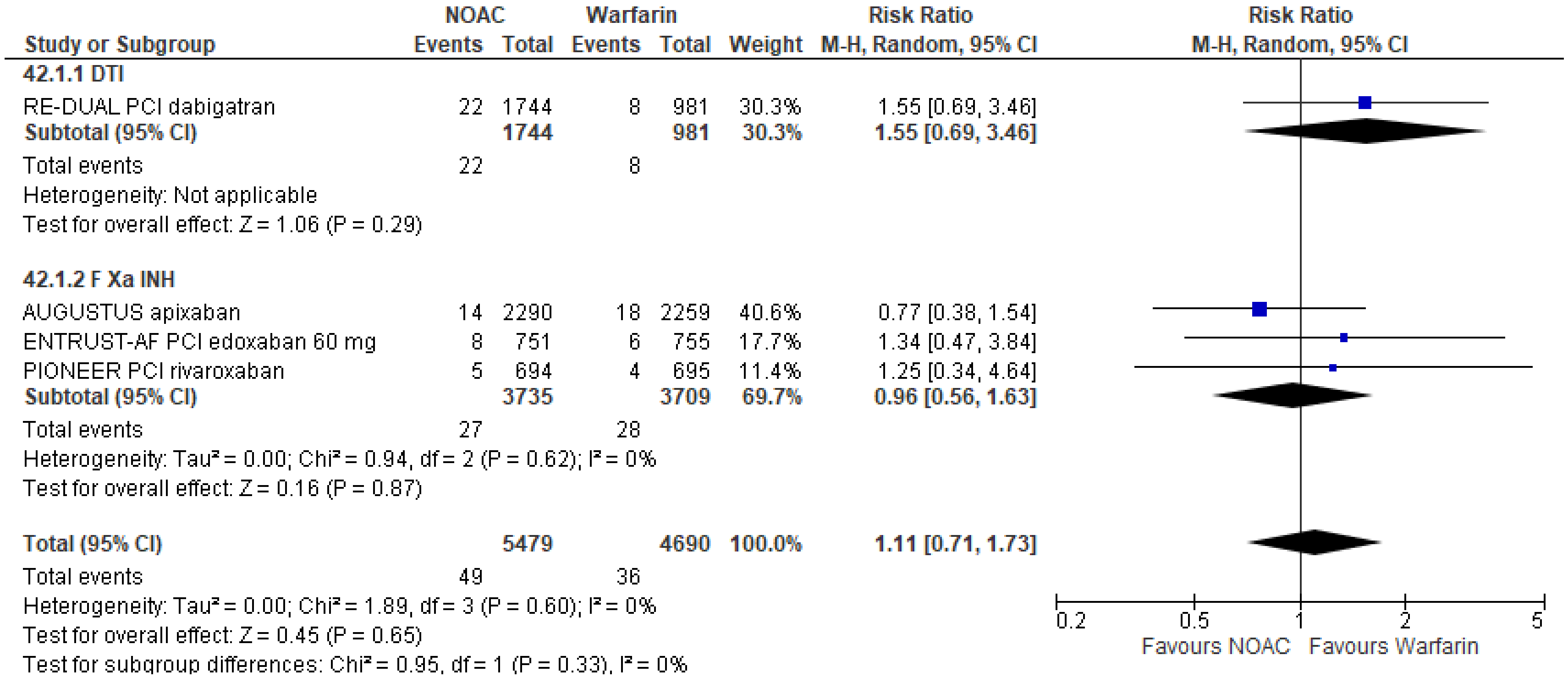

Figure S31. The funnel plot for stent thrombosis.

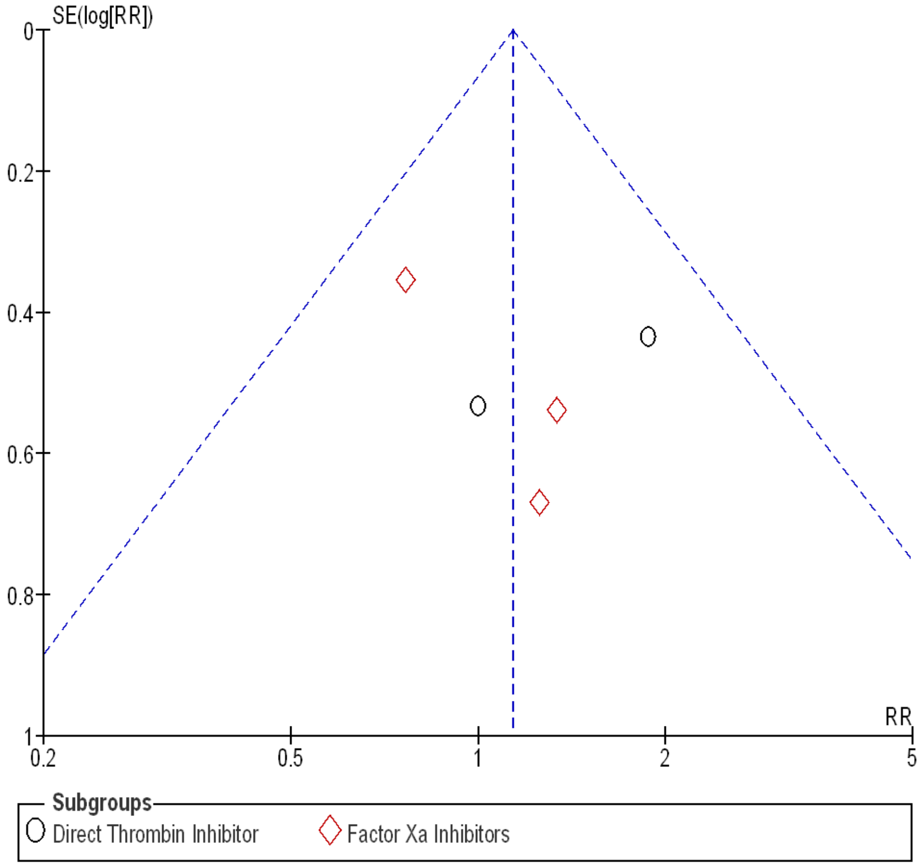

Figure S32. The meta-analysis results for cardiovascular mortality.

Study or Subgroup	NOAC Events	NOAC Total	Warfarin Events	Warfarin Total	Weight	Risk Ratio M-H, Random, 95% CI
**16.1.1 DTI**						
RE-LY* dabigatran 110 mg	289	6015	317	6022	14.9%	0.91 [0.78, 1.07]
RE-LY* dabigatran 150 mg	274	6076	317	6022	14.5%	0.86 [0.73, 1.00]
**Subtotal (95% CI)**		**12091**		**12044**	**29.4%**	**0.88 [0.79, 0.99]**
Total events	563		634			
Heterogeneity: Tau² = 0.00; Chi² = 0.31, df = 1 (P = 0.57); I² = 0%						
Test for overall effect: Z = 2.17 (P = 0.03)						
**16.1.2 F Xa INH**						
AUGUSTUS apixaban	57	2290	54	2259	2.7%	1.04 [0.72, 1.50]
ENGAGE AF-TIMI edoxaban 30 mg	527	7034	611	7036	28.9%	0.86 [0.77, 0.96]
ENGAGE AF-TIMI edoxaban 60 mg	530	7035	611	7036	29.0%	0.87 [0.78, 0.97]
ENTRUST-AF PCI edoxaban 60 mg	17	751	16	755	0.8%	1.07 [0.54, 2.10]
PIONEER PCI rivaroxaban	15	694	11	695	0.6%	1.37 [0.63, 2.95]
ROCKET - AF rivaroxaban	170	7061	193	7082	8.7%	0.88 [0.72, 1.08]
**Subtotal (95% CI)**		**24865**		**24863**	**70.6%**	**0.88 [0.82, 0.94]**
Total events	1316		1496			
Heterogeneity: Tau² = 0.00; Chi² = 2.55, df = 5 (P = 0.77); I² = 0%						
Test for overall effect: Z = 3.54 (P = 0.0004)						
**Total (95% CI)**		**36956**		**36907**	**100.0%**	**0.88 [0.83, 0.94]**
Total events	1879		2130			
Heterogeneity: Tau² = 0.00; Chi² = 2.88, df = 7 (P = 0.90); I² = 0%						
Test for overall effect: Z = 4.15 (P < 0.0001)						
Test for subgroup differences: Chi² = 0.01, df = 1 (P = 0.92), I² = 0%						

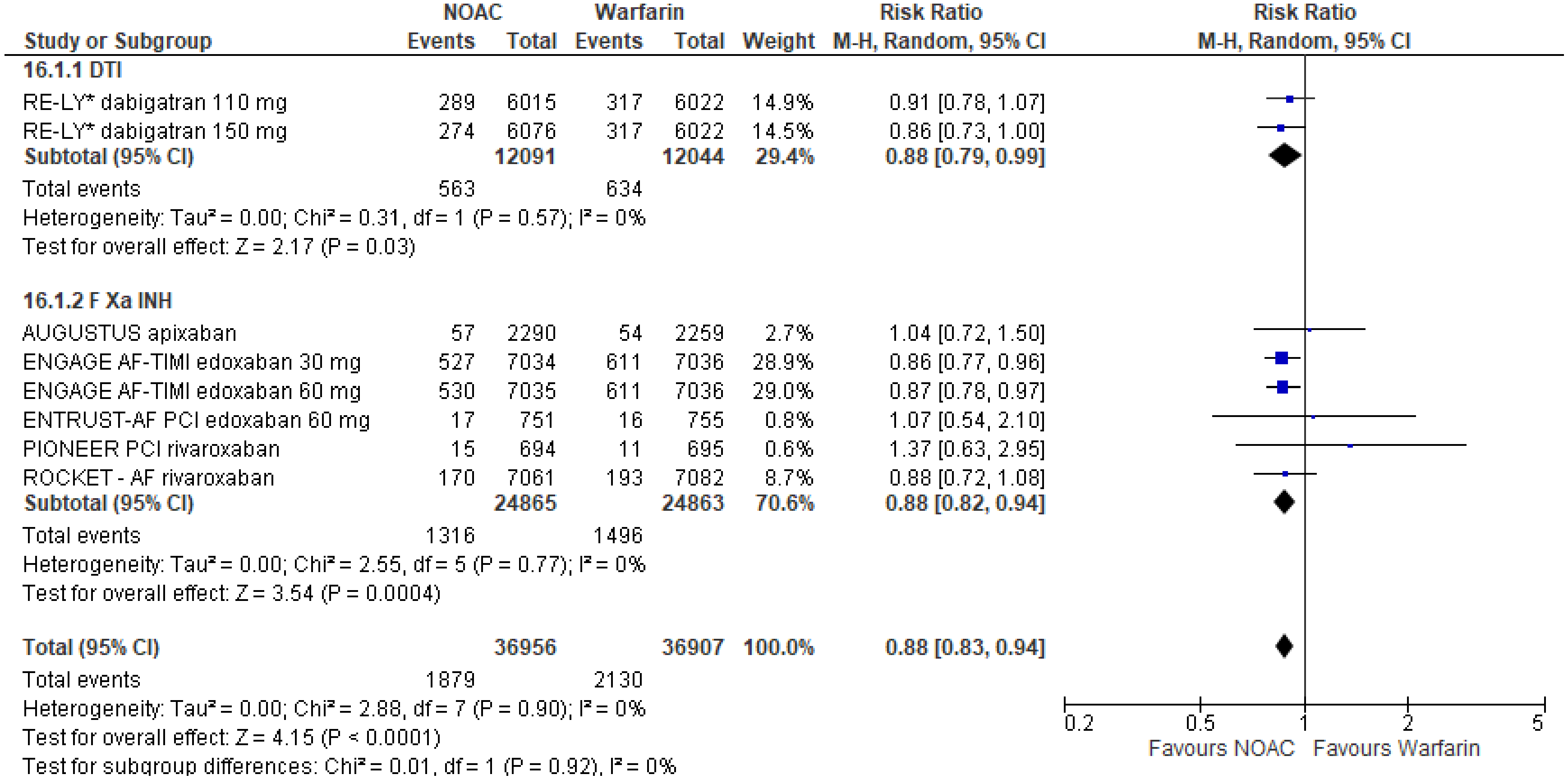

* - Defined as death from vascular causes in RE-LY study [23]

Figure S32A. The meta-analysis results for cardiovascular mortality after combining study data with respect to doses.

Study or Subgroup	NOAC Events	NOAC Total	Warfarin Events	Warfarin Total	Weight	Risk Ratio M-H, Random, 95% CI
**43.1.1 DTI**						
RE-LY* dabigatran	563	12091	317	6022	27.7%	0.88 [0.77, 1.01]
**Subtotal (95% CI)**		**12091**		**6022**	**27.7%**	**0.88 [0.77, 1.01]**
Total events	563		317			
Heterogeneity: Not applicable						
Test for overall effect: Z = 1.79 (P = 0.07)						
**43.1.2 F Xa INH**						
AUGUSTUS apixaban	57	2290	54	2259	3.7%	1.04 [0.72, 1.50]
ENGAGE-AF-TIMI edoxaban	1057	14069	611	7036	54.7%	0.87 [0.79, 0.95]
ENTRUST-AF PCI edoxaban 60 mg	17	751	16	755	1.1%	1.07 [0.54, 2.10]
PIONEER PCI rivaroxaban	15	694	11	695	0.8%	1.37 [0.63, 2.95]
ROCKET - AF rivaroxaban	170	7061	193	7082	12.0%	0.88 [0.72, 1.08]
**Subtotal (95% CI)**		**24865**		**17827**	**72.3%**	**0.88 [0.81, 0.96]**
Total events	1316		885			
Heterogeneity: Tau² = 0.00; Chi² = 2.48, df = 4 (P = 0.65); I² = 0%						
Test for overall effect: Z = 2.92 (P = 0.004)						
**Total (95% CI)**		**36956**		**23849**	**100.0%**	**0.88 [0.82, 0.95]**
Total events	1879		1202			
Heterogeneity: Tau² = 0.00; Chi² = 2.48, df = 5 (P = 0.78); I² = 0%						
Test for overall effect: Z = 3.42 (P = 0.0006)						
Test for subgroup differences: Chi² = 0.00, df = 1 (P = 0.99), I² = 0%						

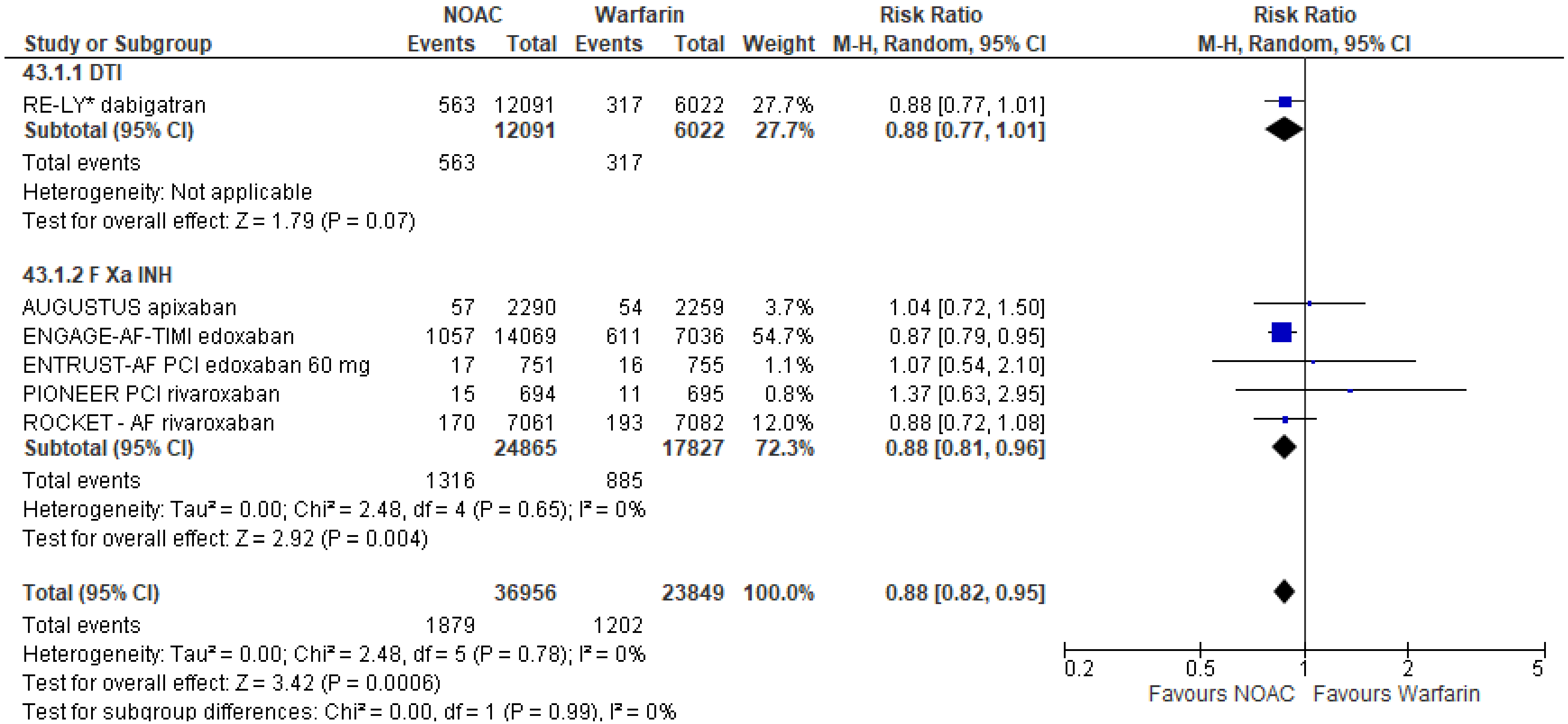

* - Defined as death from vascular causes in RE-LY study [20]

Figure S33. The funnel plot for cardiovascular mortality.

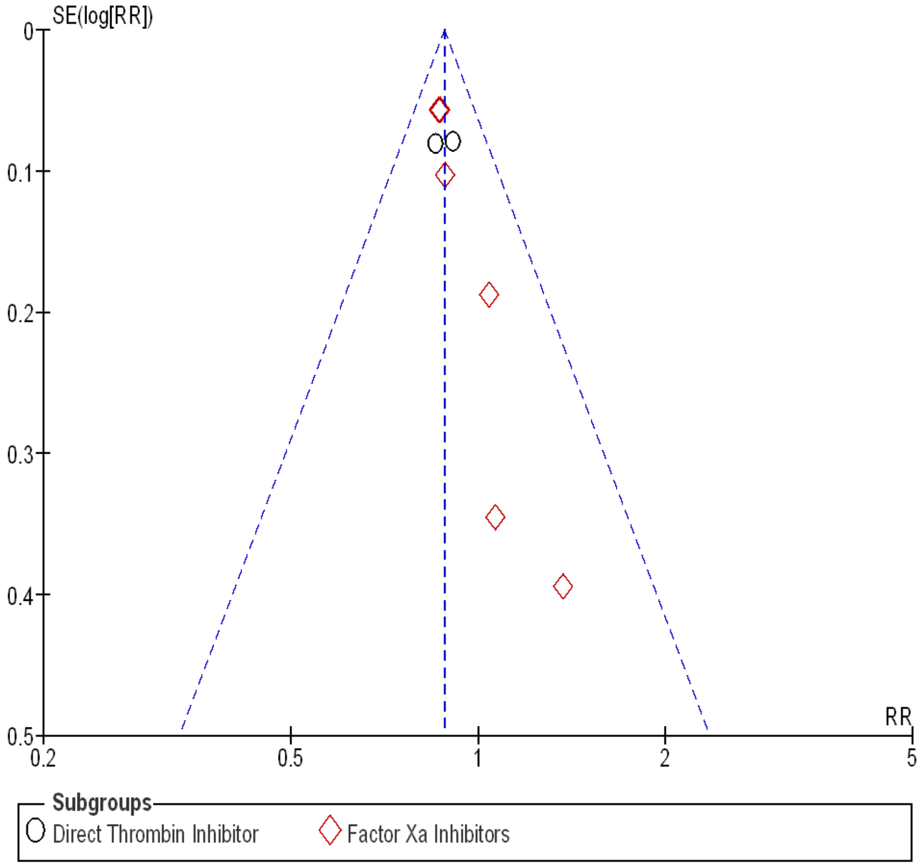

Figure S34. The meta-analysis results for cardiovascular mortality after excluding ENGAGE AF-TIMI edoxaban 30 mg.

	NOAC		Warfarin			Risk Ratio
Study or Subgroup	Events	Total	Events	Total	Weight	M-H, Random, 95% CI
**17.1.1 DTI**						
RE-LY* dabigatran 110 mg	289	6015	317	6022	21.0%	0.91 [0.78, 1.07]
RE-LY* dabigatran 150 mg	274	6076	317	6022	20.4%	0.86 [0.73, 1.00]
**Subtotal (95% CI)**		**12091**		**12044**	**41.4%**	**0.88 [0.79, 0.99]**
Total events	563		634			
Heterogeneity: Tau² = 0.00; Chi² = 0.31, df = 1 (P = 0.57); I² = 0%						
Test for overall effect: Z = 2.17 (P = 0.03)						
**17.1.2 F Xa INH**						
AUGUSTUS apixaban	57	2290	54	2259	3.7%	1.04 [0.72, 1.50]
ENGAGE AF-TIMI edoxaban 60 mg	530	7035	611	7036	40.7%	0.87 [0.78, 0.97]
ENTRUST-AF PCI edoxaban 60 mg	17	751	16	755	1.1%	1.07 [0.54, 2.10]
PIONEER PCI rivaroxaban	15	694	11	695	0.9%	1.37 [0.63, 2.95]
ROCKET - AF rivaroxaban	170	7061	193	7082	12.2%	0.88 [0.72, 1.08]
**Subtotal (95% CI)**		**17831**		**17827**	**58.6%**	**0.89 [0.81, 0.98]**
Total events	789		885			
Heterogeneity: Tau² = 0.00; Chi² = 2.37, df = 4 (P = 0.67); I² = 0%						
Test for overall effect: Z = 2.45 (P = 0.01)						
**Total (95% CI)**		**29922**		**29871**	**100.0%**	**0.89 [0.83, 0.95]**
Total events	1352		1519			
Heterogeneity: Tau² = 0.00; Chi² = 2.70, df = 6 (P = 0.85); I² = 0%						
Test for overall effect: Z = 3.27 (P = 0.001)						
Test for subgroup differences: Chi² = 0.01, df = 1 (P = 0.93), I² = 0%						

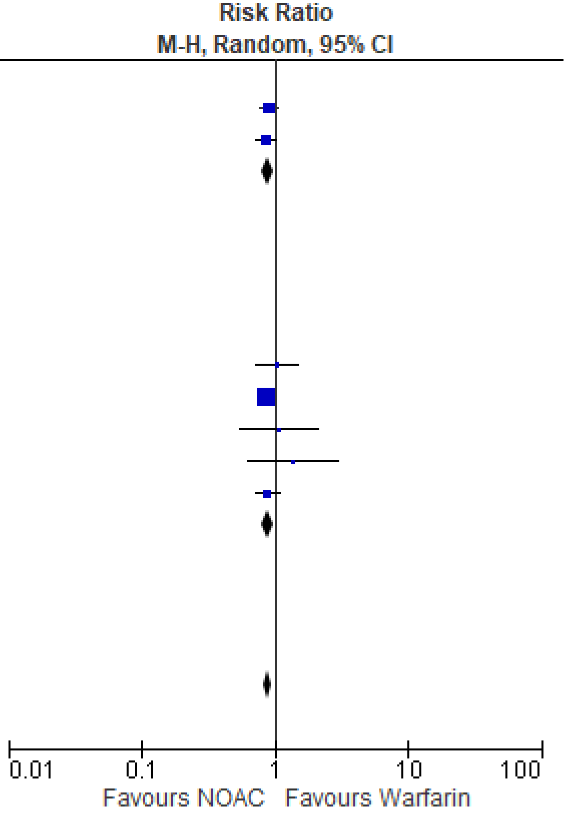

*- Defined as death from vascular causes in RE-LY study [23]

Figure S34A. The meta-analysis results for cardiovascular mortality after excluding ENGAGE AF-TIMI edoxaban 30 mg and combining study data with respect to doses.

Study or Subgroup	NOAC Events	NOAC Total	Warfarin Events	Warfarin Total	Weight	Risk Ratio M-H, Random, 95% CI
**44.1.1 DTI**						
RE-LY* dabigatran	563	12091	317	6022	32.4%	0.88 [0.77, 1.01]
**Subtotal (95% CI)**		**12091**		**6022**	**32.4%**	**0.88 [0.77, 1.01]**
Total events	563		317			
Heterogeneity: Not applicable						
Test for overall effect: Z = 1.79 (P = 0.07)						
**44.1.2 F Xa INH**						
AUGUSTUS apixaban	57	2290	54	2259	4.3%	1.04 [0.72, 1.50]
ENGAGE-AF-TIMI edoxaban	530	7035	611	7036	46.9%	0.87 [0.78, 0.97]
ENTRUST-AF PCI edoxaban 60 mg	17	751	16	755	1.3%	1.07 [0.54, 2.10]
PIONEER PCI rivaroxaban	15	694	11	695	1.0%	1.37 [0.63, 2.95]
ROCKET - AF rivaroxaban	170	7061	193	7082	14.1%	0.88 [0.72, 1.08]
**Subtotal (95% CI)**		**17831**		**17827**	**67.6%**	**0.89 [0.81, 0.98]**
Total events	789		885			
Heterogeneity: Tau² = 0.00; Chi² = 2.37, df = 4 (P = 0.67); I² = 0%						
Test for overall effect: Z = 2.45 (P = 0.01)						
**Total (95% CI)**		**29922**		**23849**	**100.0%**	**0.89 [0.82, 0.96]**
Total events	1352		1202			
Heterogeneity: Tau² = 0.00; Chi² = 2.38, df = 5 (P = 0.79); I² = 0%						
Test for overall effect: Z = 3.03 (P = 0.002)						
Test for subgroup differences: Chi² = 0.01, df = 1 (P = 0.94), I² = 0%						

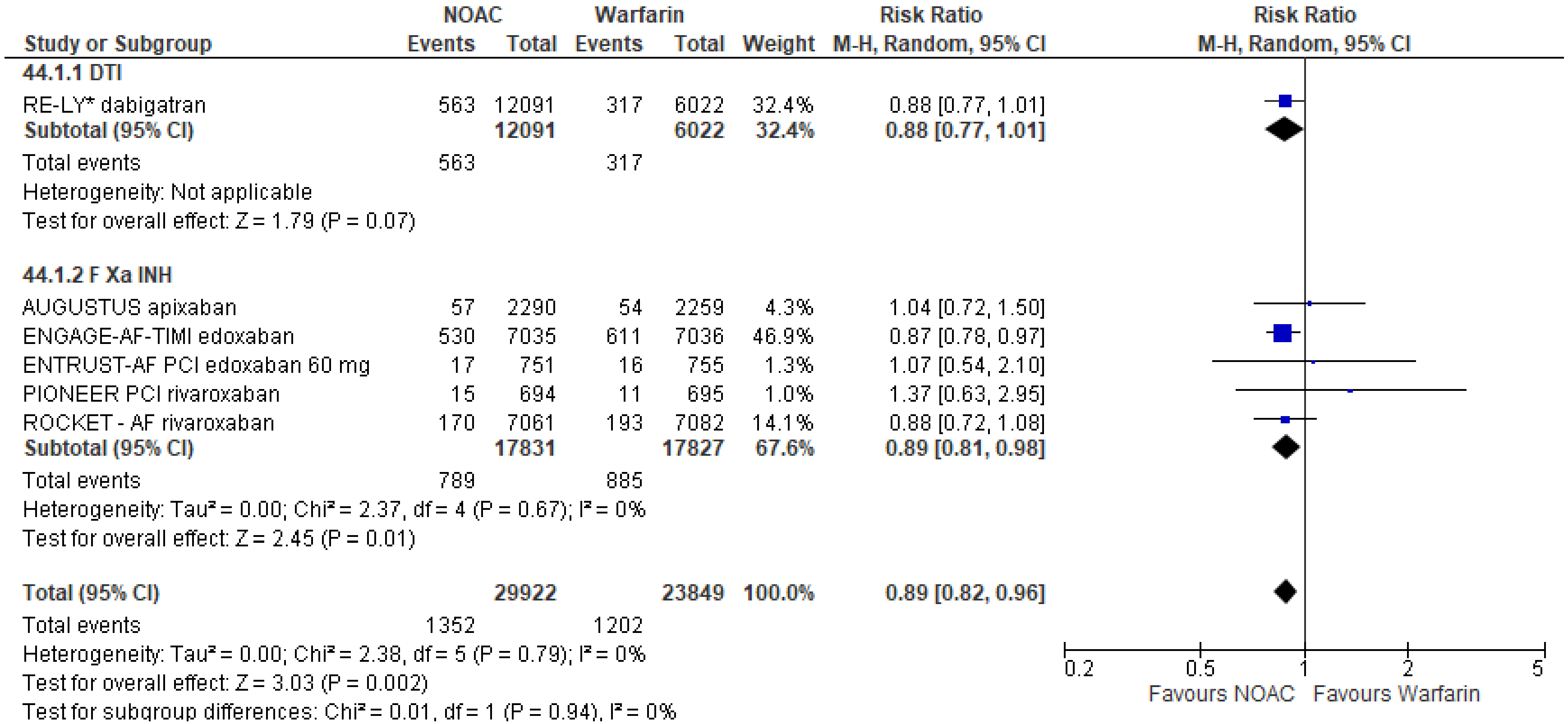

*- Defined as death from vascular causes in RE-LY study [23]

Figure S35. The funnel plot for cardiovascular mortality after excluding ENGAGE AF-TIMI edoxaban 30 mg.

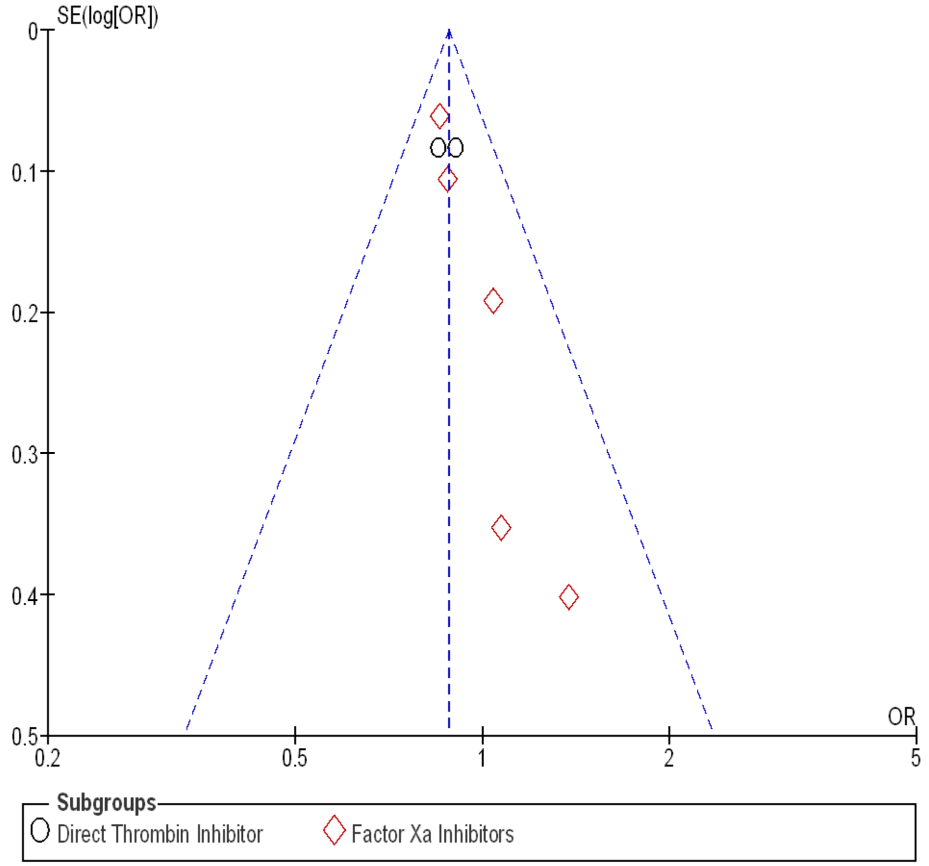

Figure S36. The meta-analysis results for cardiovascular mortality reanalysis RE-LY [27]– intention to treat data.

Study or Subgroup	NOAC Events	NOAC Total	Warfarin Events	Warfarin Total	Weight	Risk Ratio M-H, Random, 95% CI
**18.1.1 DTI**						
RE-LY** dabigatran 110 mg ITT	177	6015	174	6022	9.7%	1.02 [0.83, 1.25]
RE-LY** dabigatran 150 mg ITT	161	6076	174	6022	9.3%	0.92 [0.74, 1.13]
**Subtotal (95% CI)**		**12091**		**12044**	**19.0%**	**0.97 [0.83, 1.12]**
Total events	338		348			
Heterogeneity: Tau² = 0.00; Chi² = 0.48, df = 1 (P = 0.49); I² = 0%						
Test for overall effect: Z = 0.44 (P = 0.66)						
**18.1.2 F Xa INH**						
AUGUSTUS apixaban	57	2290	54	2259	3.1%	1.04 [0.72, 1.50]
ENGAGE AF-TIMI edoxaban 30 mg	527	7034	611	7036	33.1%	0.86 [0.77, 0.96]
ENGAGE AF-TIMI edoxaban 60 mg	530	7035	611	7036	33.2%	0.87 [0.78, 0.97]
ENTRUST-AF PCI edoxaban 60 mg	17	751	16	755	0.9%	1.07 [0.54, 2.10]
PIONEER PCI rivaroxaban	15	694	11	695	0.7%	1.37 [0.63, 2.95]
ROCKET - AF rivaroxaban	170	7061	193	7082	10.0%	0.88 [0.72, 1.08]
**Subtotal (95% CI)**		**24865**		**24863**	**81.0%**	**0.88 [0.82, 0.94]**
Total events	1316		1496			
Heterogeneity: Tau² = 0.00; Chi² = 2.55, df = 5 (P = 0.77); I² = 0%						
Test for overall effect: Z = 3.54 (P = 0.0004)						
**Total (95% CI)**		**36956**		**36907**	**100.0%**	**0.90 [0.84, 0.95]**
Total events	1654		1844			
Heterogeneity: Tau² = 0.00; Chi² = 4.36, df = 7 (P = 0.74); I² = 0%						
Test for overall effect: Z = 3.37 (P = 0.0007)						
Test for subgroup differences: Chi² = 1.32, df = 1 (P = 0.25), I² = 24.3%						

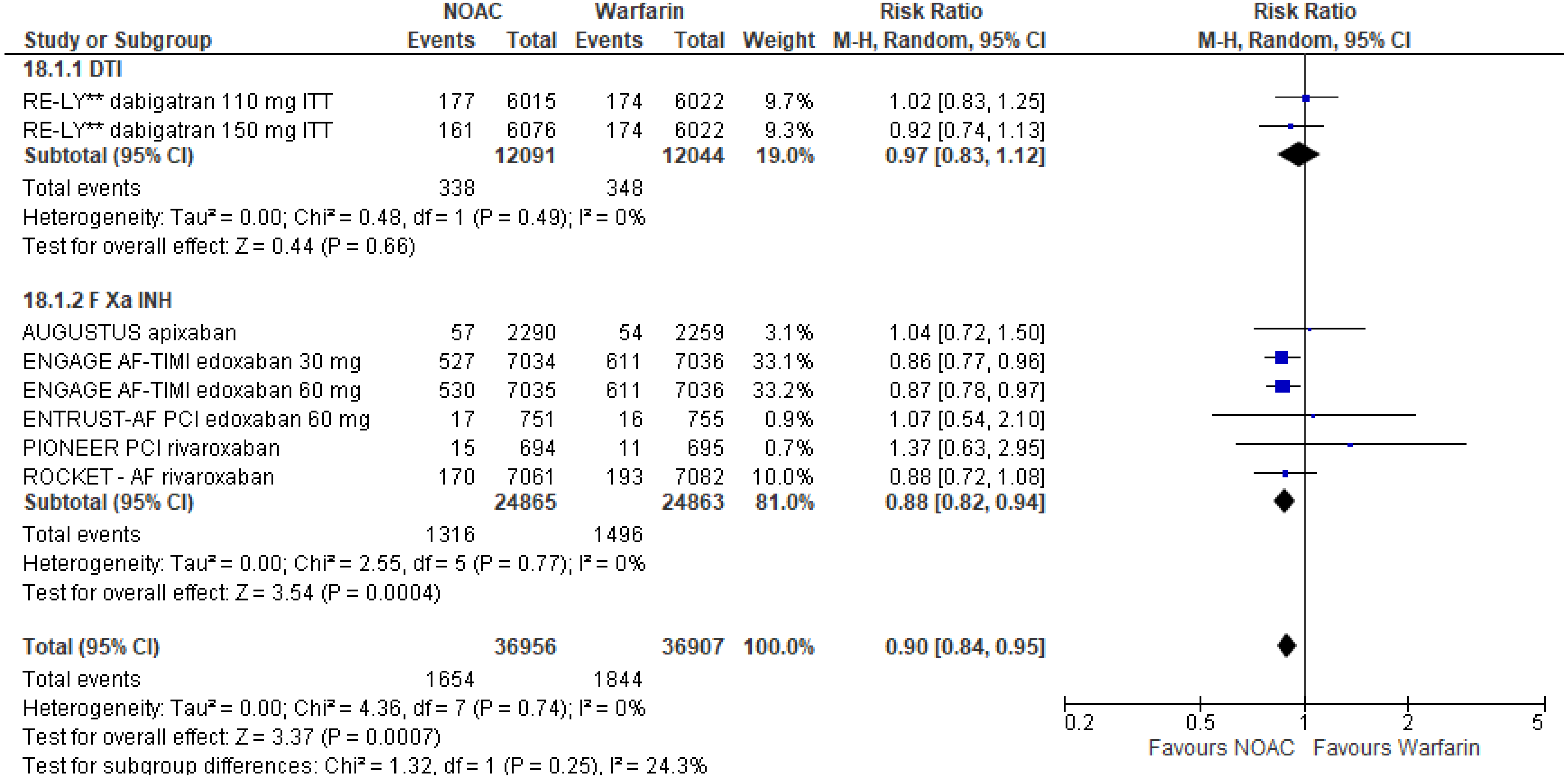

**- Defined as cardiac death in reanalysis of RE-LY study [27]

Figure S36A. The meta-analysis results for cardiovascular mortality reanalysis RE-LY [27]– intention to treat data after combining study data with respect to doses.

Study or Subgroup	NOAC Events	NOAC Total	Warfarin Events	Warfarin Total	Weight	Risk Ratio M-H, Random, 95% CI
**45.1.1 DTI**						
RE-LY** dabigatran	338	12091	174	6022		0.97 [0.81, 1.16]
**Subtotal (95% CI)**		**12091**		**6022**	**17.5%**	**0.97 [0.81, 1.16]**
Total events	338		174			
Heterogeneity: Not applicable						
Test for overall effect: Z = 0.36 (P = 0.72)						
**45.1.2 F Xa INH**						
AUGUSTUS apixaban	57	2290	54	2259	4.2%	1.04 [0.72, 1.50]
ENGAGE-AF-TIMI edoxaban	1057	14069	611	7036	62.4%	0.87 [0.79, 0.95]
ENTRUST-AF PCI edoxaban 60 mg	17	751	16	755	1.2%	1.07 [0.54, 2.10]
PIONEER PCI rivaroxaban	15	694	11	695	1.0%	1.37 [0.63, 2.95]
ROCKET - AF rivaroxaban	170	7061	193	7082	13.7%	0.88 [0.72, 1.08]
**Subtotal (95% CI)**		**24865**		**17827**	**82.5%**	**0.88 [0.81, 0.96]**
Total events	1316		885			
Heterogeneity: Tau² = 0.00; Chi² = 2.48, df = 4 (P = 0.65); I² = 0%						
Test for overall effect: Z = 2.92 (P = 0.004)						
**Total (95% CI)**		**36956**		**23849**	**100.0%**	**0.90 [0.83, 0.97]**
Total events	1654		1059			
Heterogeneity: Tau² = 0.00; Chi² = 3.28, df = 5 (P = 0.66); I² = 0%						
Test for overall effect: Z = 2.80 (P = 0.005)						
Test for subgroup differences: Chi² = 0.80, df = 1 (P = 0.37), I² = 0%						

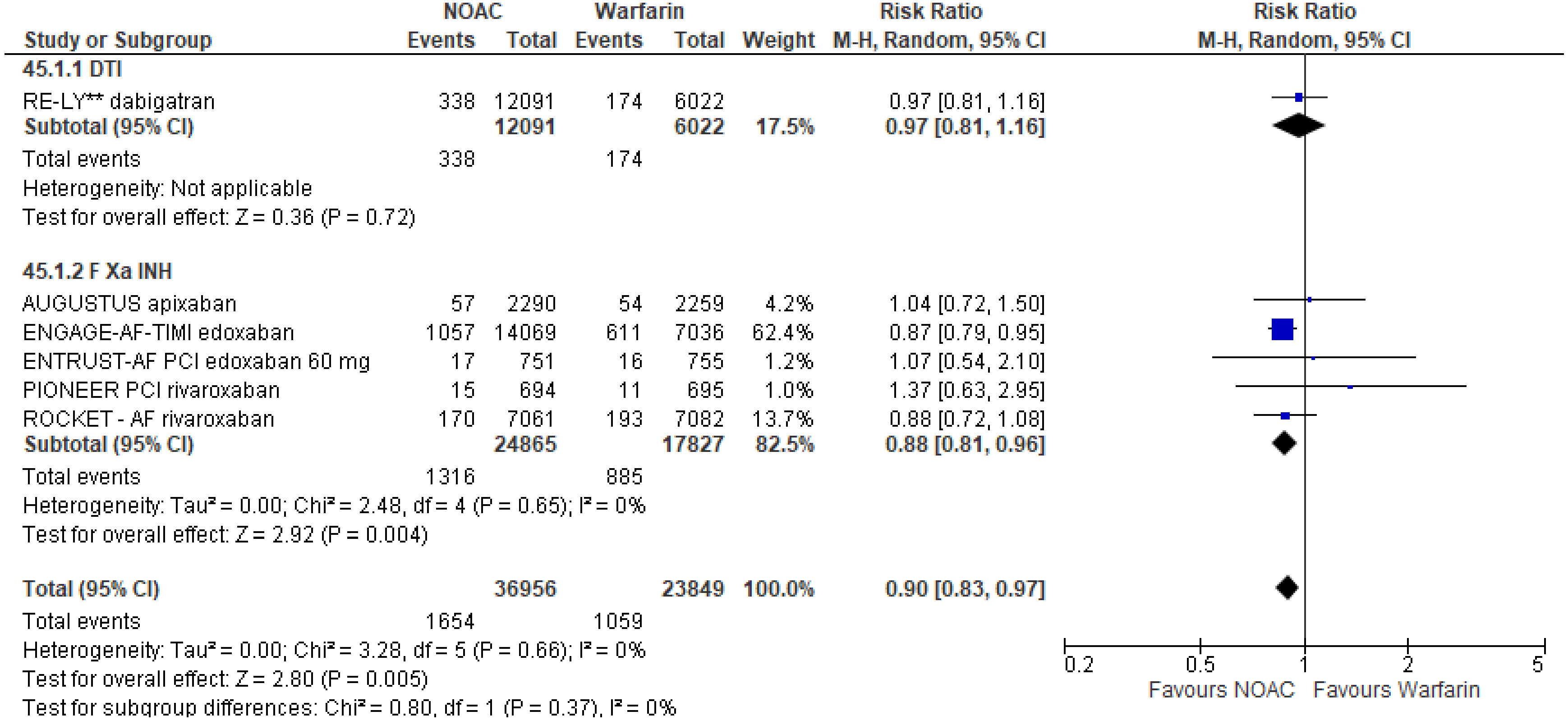

**- Defined as cardiac death in reanalysis of RE-LY study [27]

Figure S37. The funnel plot for cardiovascular mortality reanalysis RE-LY [27] – intention to treat data.

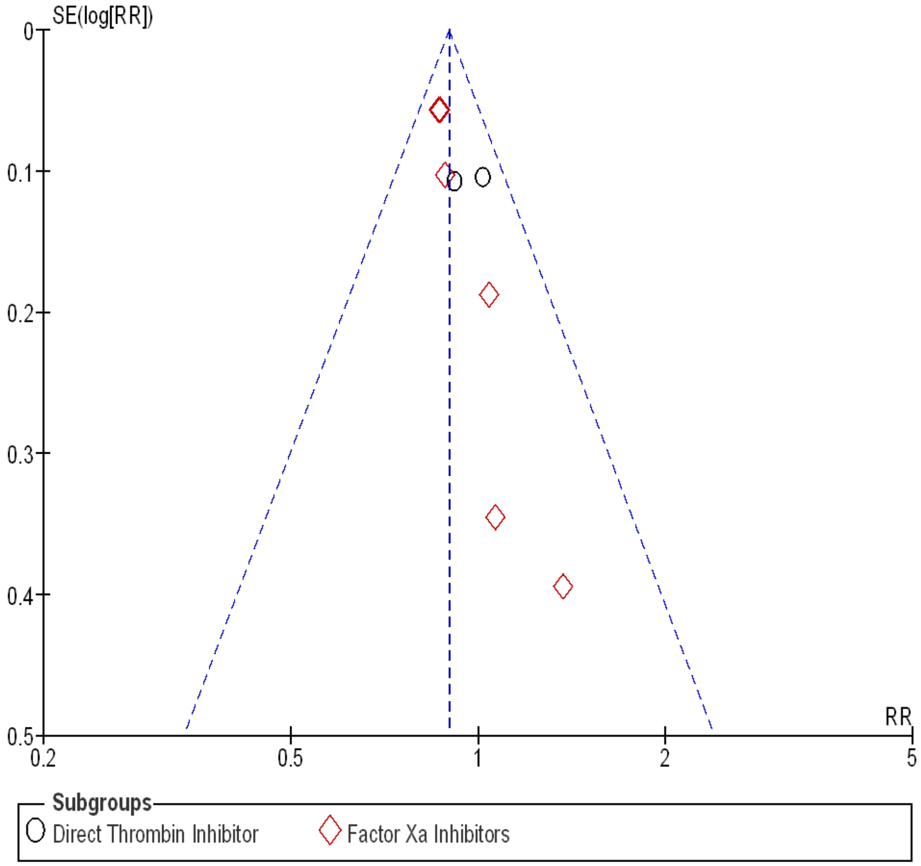

Figure S38. The meta-analysis results for cardiovascular mortality reanalysis RE-LY [27]– intention to treat data after excluding ENGAGE AF-TIMI edoxaban 30 mg.

Study or Subgroup	NOAC Events	NOAC Total	Warfarin Events	Warfarin Total	Weight	Risk Ratio M-H, Random, 95% CI
**19.1.1 DTI**						
RE-LY** dabigatran 110 mg ITT	177	6015	174	6022	14.5%	1.02 [0.83, 1.25]
RE-LY** dabigatran 150 mg ITT	161	6076	174	6022	13.8%	0.92 [0.74, 1.13]
**Subtotal (95% CI)**		**12091**		**12044**	**28.4%**	**0.97 [0.83, 1.12]**
Total events	338		348			
Heterogeneity: Tau² = 0.00; Chi² = 0.48, df = 1 (P = 0.49); I² = 0%						
Test for overall effect: Z = 0.44 (P = 0.66)						
**19.1.2 F Xa INH**						
AUGUSTUS apixaban	57	2290	54	2259	4.6%	1.04 [0.72, 1.50]
ENGAGE AF-TIMI edoxaban 60 mg	530	7035	611	7036	49.7%	0.87 [0.78, 0.97]
ENTRUST-AF PCI edoxaban 60 mg	17	751	16	755	1.4%	1.07 [0.54, 2.10]
PIONEER PCI rivaroxaban	15	694	11	695	1.0%	1.37 [0.63, 2.95]
ROCKET - AF rivaroxaban	170	7061	193	7082	14.9%	0.88 [0.72, 1.08]
**Subtotal (95% CI)**		**17831**		**17827**	**71.6%**	**0.89 [0.81, 0.98]**
Total events	789		885			
Heterogeneity: Tau² = 0.00; Chi² = 2.37, df = 4 (P = 0.67); I² = 0%						
Test for overall effect: Z = 2.45 (P = 0.01)						
**Total (95% CI)**		**29922**		**29871**	**100.0%**	**0.91 [0.84, 0.99]**
Total events	1127		1233			
Heterogeneity: Tau² = 0.00; Chi² = 3.73, df = 6 (P = 0.71); I² = 0%						
Test for overall effect: Z = 2.30 (P = 0.02)						
Test for subgroup differences: Chi² = 0.87, df = 1 (P = 0.35), I² = 0%						

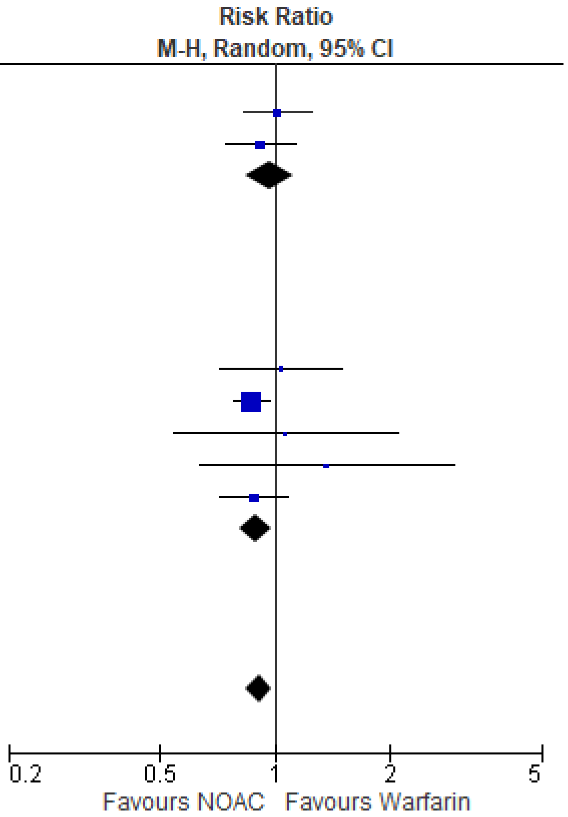

** - Defined as cardiac death in reanalysis of RE-LY study [27]

Figure S38A. The meta-analysis results for cardiovascular mortality reanalysis RE-LY [27]– intention to treat data after excluding ENGAGE AF-TIMI edoxaban 30 mg and combining study data with respect to doses.

Study or Subgroup	NOAC Events	NOAC Total	Warfarin Events	Warfarin Total	Weight	Risk Ratio M-H, Random, 95% CI
**46.1.1 DTI**						
RE-LY** dabigatran ITT	338	12091	174	6022	21.0%	0.97 [0.81, 1.16]
**Subtotal (95% CI)**		**12091**		**6022**	**21.0%**	**0.97 [0.81, 1.16]**
Total events	338		174			
Heterogeneity: Not applicable						
Test for overall effect: Z = 0.36 (P = 0.72)						
**46.1.2 F Xa INH**						
AUGUSTUS apixaban	57	2290	54	2259	5.0%	1.04 [0.72, 1.50]
ENGAGE-AF-TIMI edoxaban	530	7035	611	7036	54.8%	0.87 [0.78, 0.97]
ENTRUST-AF PCI edoxaban 60 mg	17	751	16	755	1.5%	1.07 [0.54, 2.10]
PIONEER PCI rivaroxaban	15	694	11	695	1.1%	1.37 [0.63, 2.95]
ROCKET - AF rivaroxaban	170	7061	193	7082	16.5%	0.88 [0.72, 1.08]
**Subtotal (95% CI)**		**17831**		**17827**	**79.0%**	**0.89 [0.81, 0.98]**
Total events	789		885			
Heterogeneity: Tau² = 0.00; Chi² = 2.37, df = 4 (P = 0.67); I² = 0%						
Test for overall effect: Z = 2.45 (P = 0.01)						
**Total (95% CI)**		**29922**		**23849**	**100.0%**	**0.91 [0.83, 0.98]**
Total events	1127		1059			
Heterogeneity: Tau² = 0.00; Chi² = 3.02, df = 5 (P = 0.70); I² = 0%						
Test for overall effect: Z = 2.34 (P = 0.02)						
Test for subgroup differences: Chi² = 0.64, df = 1 (P = 0.42), I² = 0%						

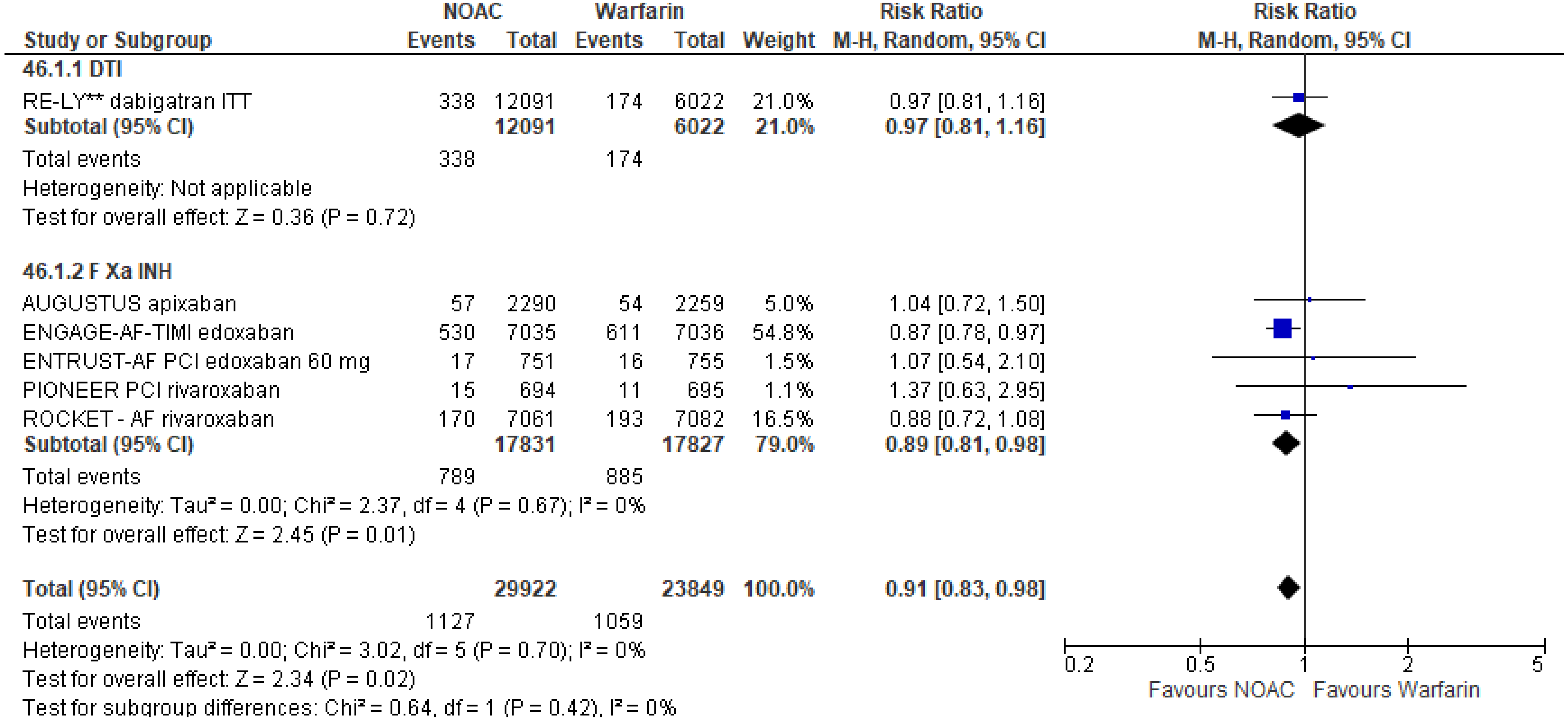

** - Defined as cardiac death in reanalysis of RE-LY study [27]

Figure S39. The funnel plot for cardiovascular mortality reanalysis RE-LY [27] – intention to treat data after excluding ENGAGE AF-TIMI edoxaban 30 mg.

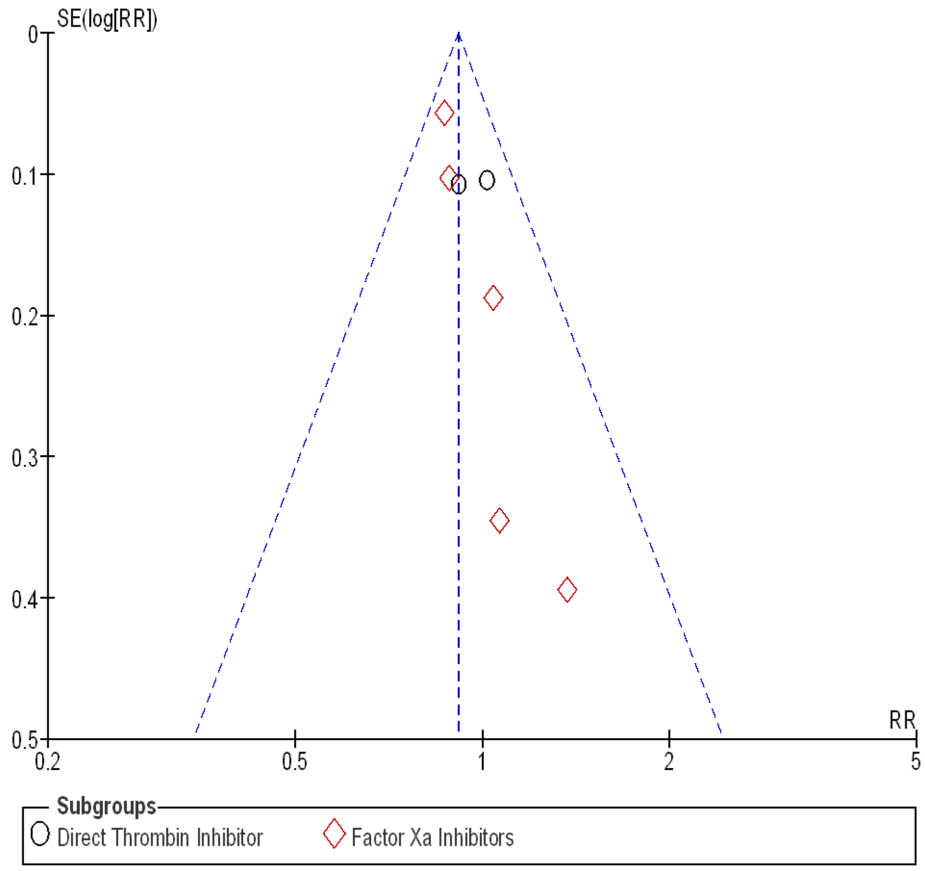

Figure S40. The meta-analysis results for cardiovascular mortality reanalysis RE-LY [27] – on treatment data.

Study or Subgroup	NOAC Events	NOAC Total	Warfarin Events	Warfarin Total	Weight	Risk Ratio M-H, Random, 95% CI
**20.1.1 DTI**						
RE-LY** dabigatran 110mg on-T	123	5983	126	5998	7.3%	0.98 [0.77, 1.25]
RE-LY** dabigatran 150mg on-T	101	6059	126	5998	6.5%	0.79 [0.61, 1.03]
**Subtotal (95% CI)**		**12042**		**11996**	**13.8%**	**0.88 [0.72, 1.09]**
Total events	224		252			
Heterogeneity: Tau² = 0.01; Chi² = 1.32, df = 1 (P = 0.25); I² = 24%						
Test for overall effect: Z = 1.17 (P = 0.24)						
**20.1.2 F Xa INH**						
AUGUSTUS apixaban	57	2090	54	2259	3.3%	1.14 [0.79, 1.65]
ENGAGE AF-TIMI edoxaban 30 mg	527	7034	611	7036	35.2%	0.86 [0.77, 0.96]
ENGAGE AF-TIMI edoxaban 60 mg	530	7035	611	7036	35.4%	0.87 [0.78, 0.97]
ENTRUST-AF PCI edoxaban 60 mg	17	751	16	755	1.0%	1.07 [0.54, 2.10]
PIONEER PCI rivaroxaban	15	694	11	695	0.7%	1.37 [0.63, 2.95]
ROCKET - AF rivaroxaban	170	7061	193	7082	10.6%	0.88 [0.72, 1.08]
**Subtotal (95% CI)**		**24665**		**24863**	**86.2%**	**0.88 [0.82, 0.95]**
Total events	1316		1496			
Heterogeneity: Tau² = 0.00; Chi² = 3.66, df = 5 (P = 0.60); I² = 0%						
Test for overall effect: Z = 3.44 (P = 0.0006)						
**Total (95% CI)**		**36707**		**36859**	**100.0%**	**0.88 [0.83, 0.94]**
Total events	1540		1748			
Heterogeneity: Tau² = 0.00; Chi² = 4.99, df = 7 (P = 0.66); I² = 0%						
Test for overall effect: Z = 3.69 (P = 0.0002)						
Test for subgroup differences: Chi² = 0.00, df = 1 (P = 0.98), I² = 0%						

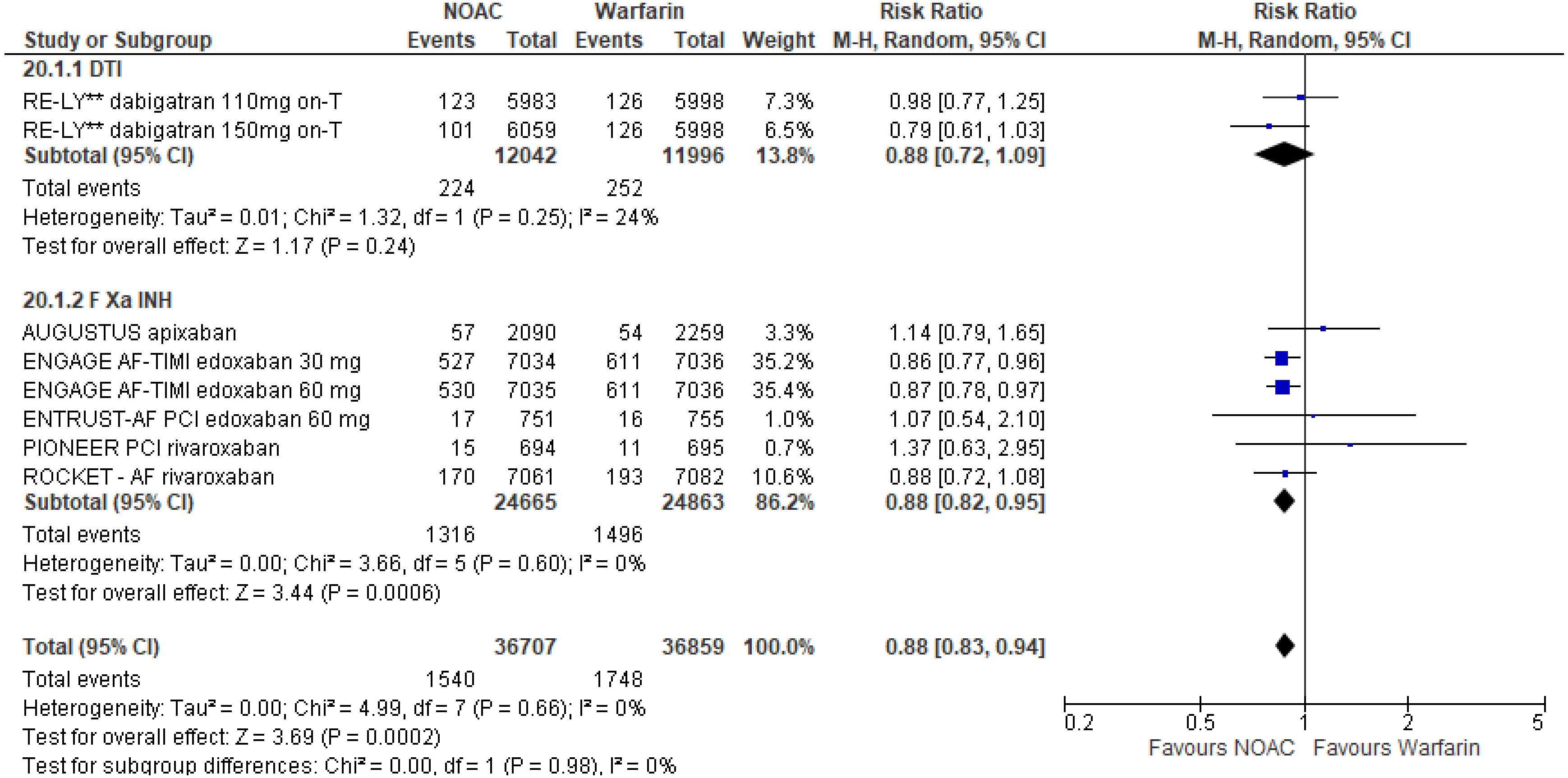

**- Defined as cardiac death in reanalysis of RE-LY study [27]

Figure S40A. The meta-analysis results for cardiovascular mortality reanalysis RE-LY [27] – on treatment data after combining study data with respect to doses.

	NOAC		Warfarin			Risk Ratio
**Study or Subgroup**	**Events**	**Total**	**Events**	**Total**	**Weight**	**M-H, Random, 95% CI**
**47.1.1 DTI**						
RE-LY** dabigatran on-T	224	12042	126	5998	12.9%	0.89 [0.71, 1.10]
**Subtotal (95% CI)**		**12042**		**5998**	**12.9%**	**0.89 [0.71, 1.10]**
Total events	224		126			
Heterogeneity: Not applicable						
Test for overall effect: Z = 1.10 (P = 0.27)						
**47.1.2 F Xa INH**						
AUGUSTUS apixaban	57	2090	54	2259	4.4%	1.14 [0.79, 1.65]
ENGAGE-AF-TIMI edoxaban	1057	14069	611	7036	65.9%	0.87 [0.79, 0.95]
ENTRUST-AF PCI edoxaban 60 mg	17	751	16	755	1.3%	1.07 [0.54, 2.10]
PIONEER PCI rivaroxaban	15	694	11	695	1.0%	1.37 [0.63, 2.95]
ROCKET - AF rivaroxaban	170	7061	193	7082	14.5%	0.88 [0.72, 1.08]
**Subtotal (95% CI)**		**24665**		**17827**	**87.1%**	**0.89 [0.82, 0.96]**
Total events	1316		885			
Heterogeneity: Tau² = 0.00; Chi² = 3.56, df = 4 (P = 0.47); I² = 0%						
Test for overall effect: Z = 2.81 (P = 0.005)						
**Total (95% CI)**		**36707**		**23825**	**100.0%**	**0.89 [0.82, 0.96]**
Total events	1540		1011			
Heterogeneity: Tau² = 0.00; Chi² = 3.56, df = 5 (P = 0.61); I² = 0%						
Test for overall effect: Z = 3.02 (P = 0.003)						
Test for subgroup differences: Chi² = 0.00, df = 1 (P = 0.98), I² = 0%						

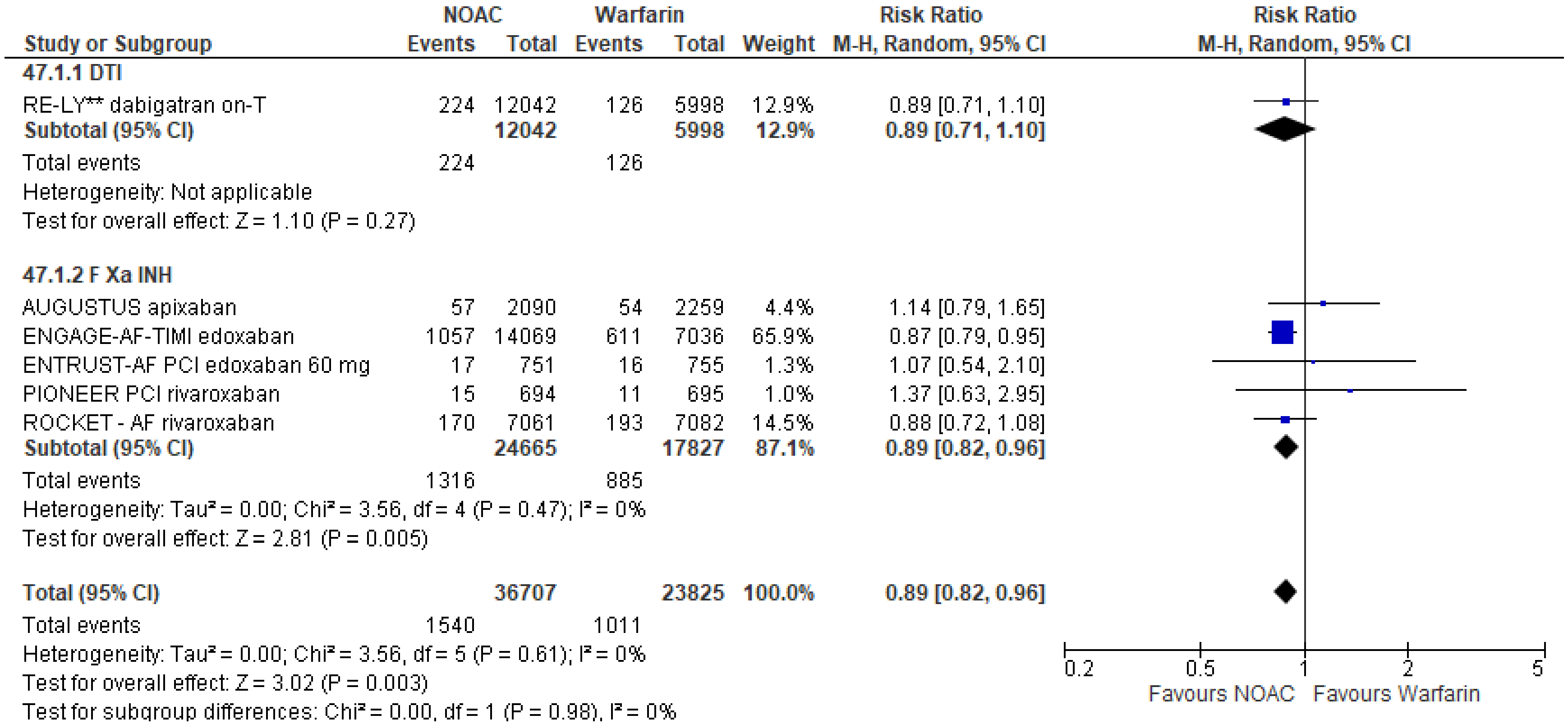

**- Defined as cardiac death in reanalysis of RE-LY study [27]

Figure S41. The funnel plot for cardiovascular mortality - re-analysis RE-LY [27] – on treatment data.

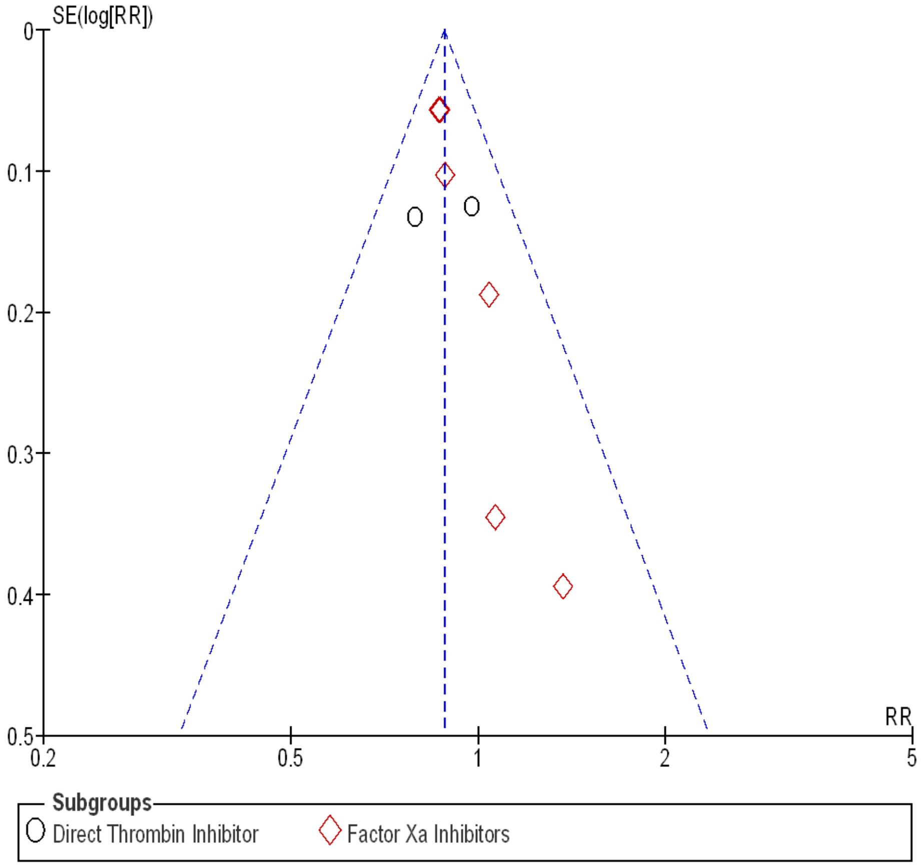

Figure S42. The meta-analysis results for cardiovascular mortality reanalysis RE-LY [27] – on treatment data after excluding ENGAGE AF-TIMI edoxaban 30 mg.

Study or Subgroup	NOAC Events	NOAC Total	Warfarin Events	Warfarin Total	Weight	Risk Ratio M-H, Random, 95% CI
**21.1.1 DTI**						
RE-LY** dabigatran 110mg on-T	123	5983	126	5998	11.2%	0.98 [0.77, 1.25]
RE-LY** dabigatran 150mg on-T	101	6059	126	5998	10.1%	0.79 [0.61, 1.03]
**Subtotal (95% CI)**		**12042**		**11996**	**21.3%**	**0.88 [0.72, 1.09]**
Total events	224		252			
Heterogeneity: Tau² = 0.01; Chi² = 1.32, df = 1 (P = 0.25); I² = 24%						
Test for overall effect: Z = 1.17 (P = 0.24)						
**21.1.2 F Xa INH**						
AUGUSTUS apixaban	57	2290	54	2259	5.0%	1.04 [0.72, 1.50]
ENGAGE AF-TIMI edoxaban 60 mg	530	7035	611	7036	54.6%	0.87 [0.78, 0.97]
ENTRUST-AF PCI edoxaban 60 mg	17	751	16	755	1.5%	1.07 [0.54, 2.10]
PIONEER PCI rivaroxaban	15	694	11	695	1.1%	1.37 [0.63, 2.95]
ROCKET - AF rivaroxaban	170	7061	193	7082	16.4%	0.88 [0.72, 1.08]
**Subtotal (95% CI)**		**17831**		**17827**	**78.7%**	**0.89 [0.81, 0.98]**
Total events	789		885			
Heterogeneity: Tau² = 0.00; Chi² = 2.37, df = 4 (P = 0.67); I² = 0%						
Test for overall effect: Z = 2.45 (P = 0.01)						
**Total (95% CI)**		**29873**		**29823**	**100.0%**	**0.89 [0.82, 0.97]**
Total events	1013		1137			
Heterogeneity: Tau² = 0.00; Chi² = 3.70, df = 6 (P = 0.72); I² = 0%						
Test for overall effect: Z = 2.78 (P = 0.005)						
Test for subgroup differences: Chi² = 0.00, df = 1 (P = 0.96), I² = 0%						

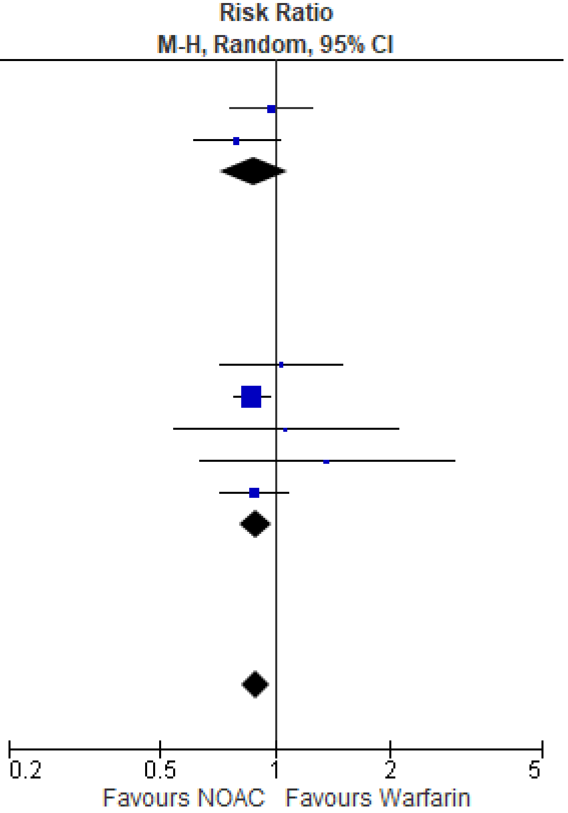

** - Defined as cardiac death in reanalysis of RE-LY study [27]

Figure S42A. The meta-analysis results for cardiovascular mortality reanalysis RE-LY [27] – on treatment data after excluding ENGAGE AF-TIMI edoxaban 30 mg and combining study data with respect to doses.

Study or Subgroup	NOAC Events	NOAC Total	Warfarin Events	Warfarin Total	Weight	Risk Ratio M-H, Random, 95% CI
**48.1.1 DTI**						
RE-LY** dabigatran on-T	224	12042	126	5998	15.6%	0.89 [0.71, 1.10]
**Subtotal (95% CI)**		**12042**		**5998**	**15.6%**	**0.89 [0.71, 1.10]**
Total events	224		126			
Heterogeneity: Not applicable						
Test for overall effect: Z = 1.10 (P = 0.27)						
**48.1.2 F Xa INH**						
AUGUSTUS apixaban	57	2090	54	2259	5.4%	1.14 [0.79, 1.65]
ENGAGE-AF-TIMI edoxaban	530	7035	611	7036	58.6%	0.87 [0.78, 0.97]
ENTRUST-AF PCI edoxaban 60 mg	17	751	16	755	1.6%	1.07 [0.54, 2.10]
PIONEER PCI rivaroxaban	15	694	11	695	1.2%	1.37 [0.63, 2.95]
ROCKET - AF rivaroxaban	170	7061	193	7082	17.6%	0.88 [0.72, 1.08]
**Subtotal (95% CI)**		**17631**		**17827**	**84.4%**	**0.90 [0.82, 0.98]**
Total events	789		885			
Heterogeneity: Tau² = 0.00; Chi² = 3.41, df = 4 (P = 0.49); I² = 0%						
Test for overall effect: Z = 2.32 (P = 0.02)						
**Total (95% CI)**		**29673**		**23825**	**100.0%**	**0.89 [0.82, 0.97]**
Total events	1013		1011			
Heterogeneity: Tau² = 0.00; Chi² = 3.42, df = 5 (P = 0.64); I² = 0%						
Test for overall effect: Z = 2.57 (P = 0.01)						
Test for subgroup differences: Chi² = 0.01, df = 1 (P = 0.92), I² = 0%						

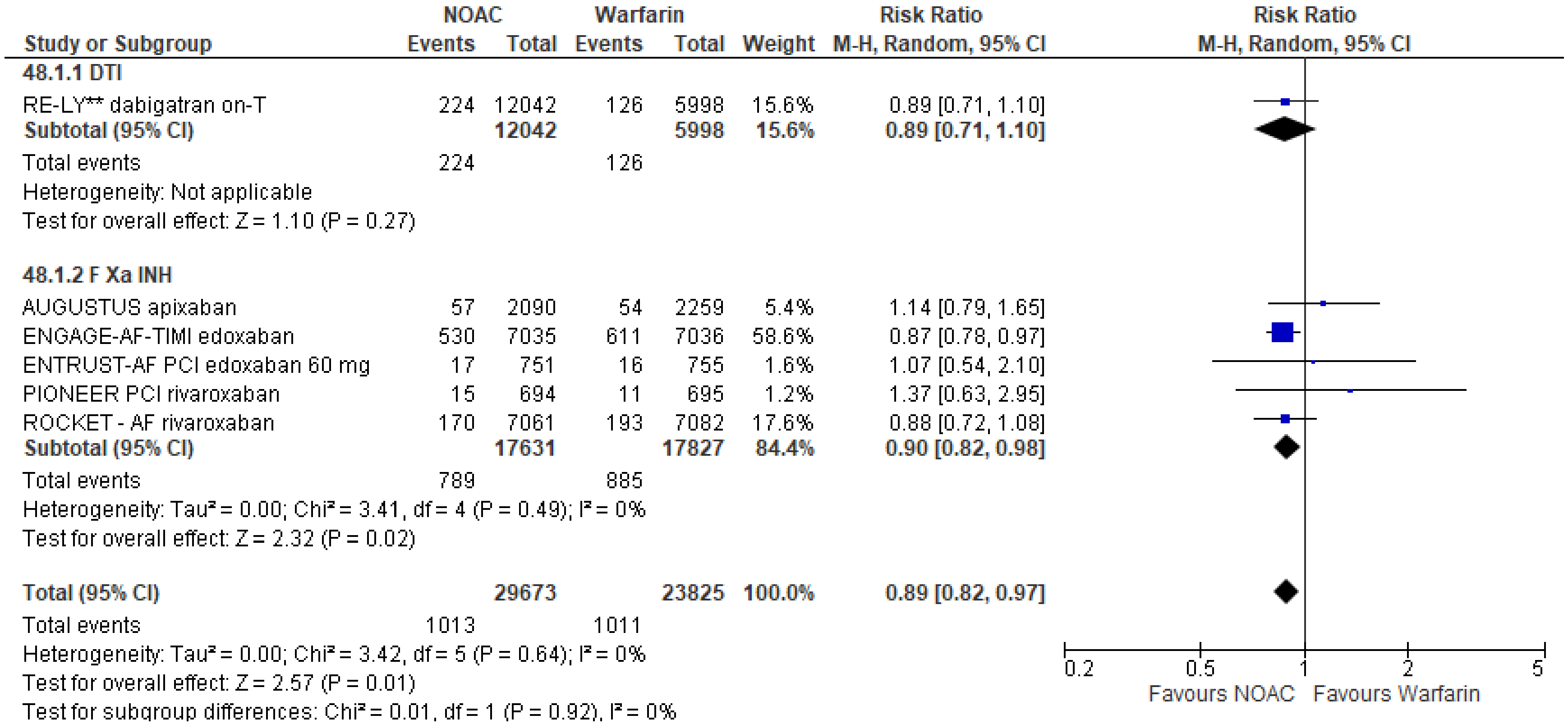

** - Defined as cardiac death in reanalysis of RE-LY study [27]

Figure S43. The funnel plot for cardiovascular mortality reanalysis RE-LY [27] – on treatment data after excluding ENGAGE AF-TIMI edoxaban 30 mg.

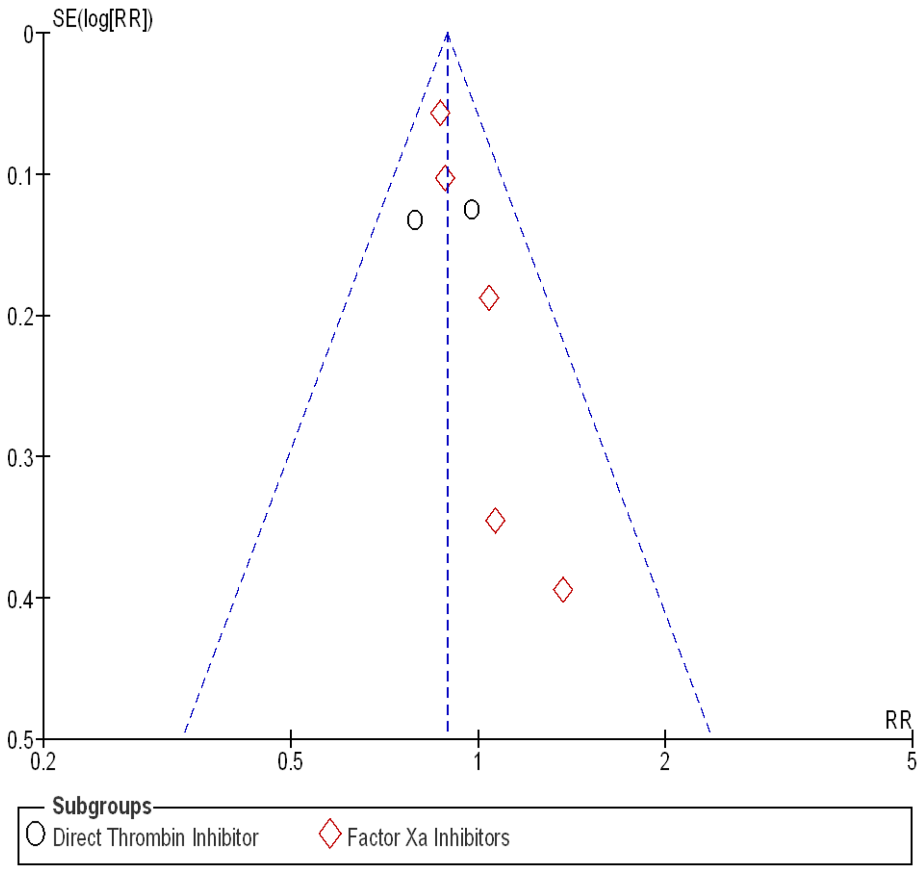

Figure S44. The network plot of compared NOAC's and warfarin.

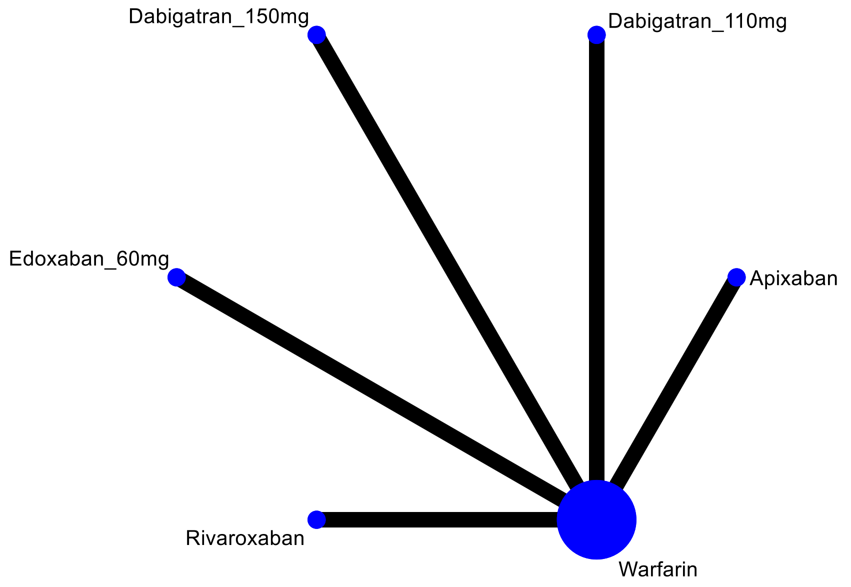

Table S3. Direct and indirect comparison between warfarin and NOAC's – MACE– intention to treat data.**

Dabigatran_110mg RR (95%CI)					
1,13 (0,93 - 1,39)	Dabigatran_150mg RR (95%CI)				
1,13 (0,93 - 1,37)	0,99 (0,87 - 1,13)	Apixaban RR (95%CI)			
1,12 (0,92 - 1,36)	0,99 (0,87 - 1,13)	1 (0,88 - 1,12)	Edoxaban_60mg RR (95%CI)		
1,11 (0,84 - 1,48)	0,98 (0,74 - 1,31)	0,99 (0,75 - 1,31)	1 (0,88 - 1,12)	Rivaroxaban RR (95%CI)	
1,01 (0,85 - 1,21)	**0,89 (0,81 - 0,99)**	**0,9 (0,83 - 0,98)**	**0,9 (0,83 - 0,98)**	0,91 (0,7 - 1,19)	Warfarin RR (95%CI)

Treatment 1	
RR (95%CI)	Treatment 2

** results from the reanalysis of RE-LY study [31]

Table S4. Treatment hierarchy assessed by surface under the cumulative ranking (SUCRA) curves. – MACE – intention to treat data.**

Treatment	SUCRA
Warfarin	7,6
Apixaban	62,9
Dabigatran_110mg	19,3
Dabigatran_150mg	66,3
Edoxaban_60mg	60,9
Rivaroxaban	83,0

**- results from the reanalysis of RE-LY study [31]

Figure S45. Rankograms for the drugs network showing the probability every treatment being at particular order– MACE – intention to treat data.**

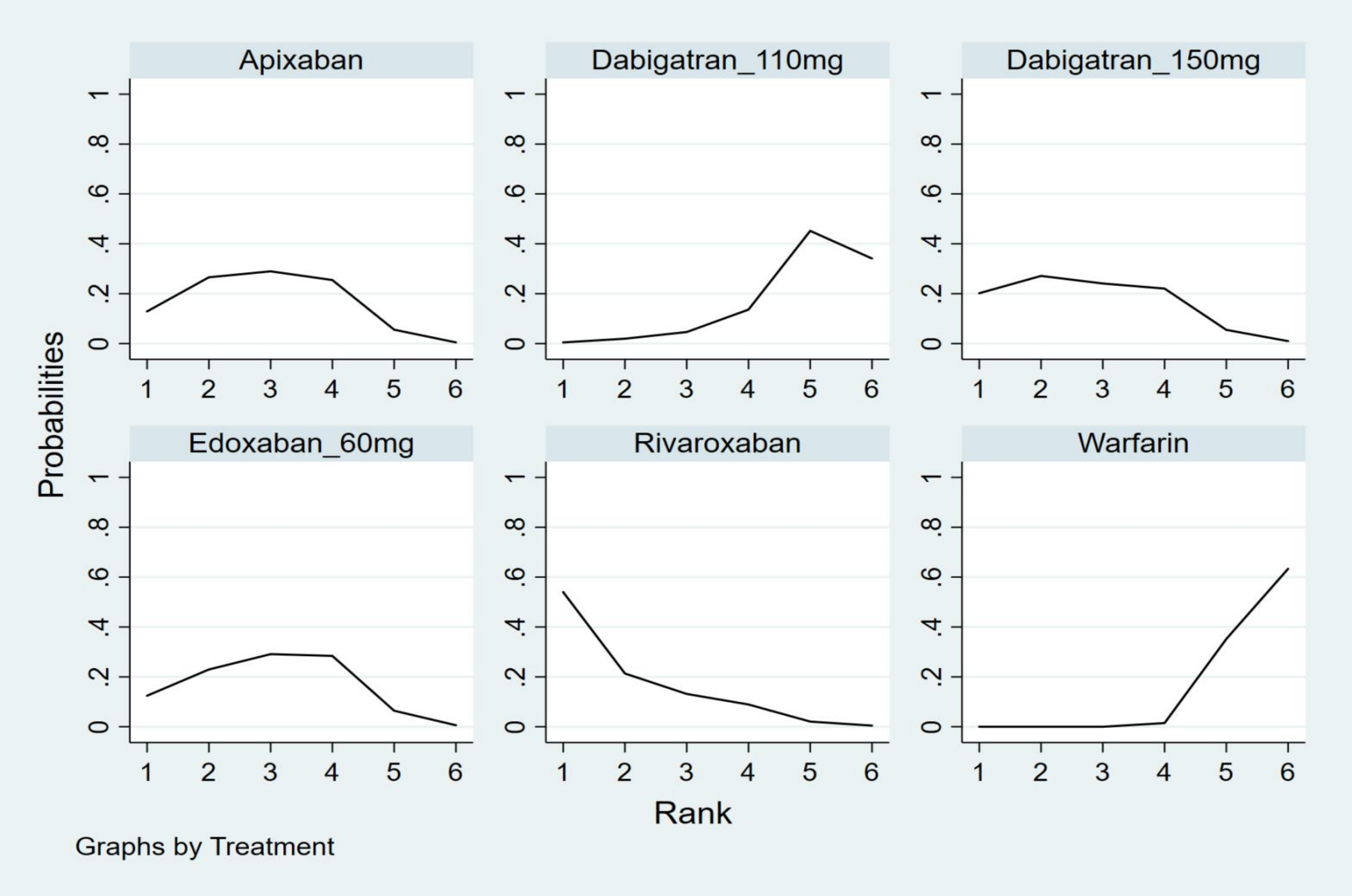

** - results from the reanalysis of RE-LY study [31]

Table S5. Direct and indirect comparison between warfarin and NOAC's – MACE** – on treatment data.

Dabigatran_110mg RR (95%CI)					
1,18 (0,97 - 1,43)	Dabigatran_150mg RR (95%CI)				
1,13 (0,94 - 1,36)	0,96 (0,84 - 1,1)	Apixaban RR (95%CI)			
1,13 (0,94 - 1,36)	0,96 (0,83 - 1,1)	1 (0,88 - 1,12)	Edoxaban_60mg RR (95%CI)		
1,12 (0,84 - 1,49)	0,95 (0,71 - 1,27)	0,99 (0,75 - 1,31)	1 (0,88 - 1,12)	Rivaroxaban RR (95%CI)	
1,02 (0,86 - 1,2)	**0,86 (0,77 - 0,96)**	**0,9 (0,83 - 0,98)**	**0,9 (0,83 - 0,98)**	0,91 (0,7 - 1,19)	Warfarin RR (95%CI)

Treatment 1	
RR (95%CI)	Treatment 2

** - results from the reanalysis of RE-LY study [31]

Table S6. Treatment hierarchy assessed by surface under the cumulative ranking (SUCRA) curves. – MACE on treatment data.**

Treatment	SUCRA
Warfarin	9,1
Apixaban	59,7
Dabigatran_110mg	15,5
Dabigatran_150mg	78,0
Edoxaban_60mg	57,8
Rivaroxaban	79,9

** - results from the reanalysis of RE-LY study [31]

Figure S46. Rankograms for the drugs network showing the probability every treatment being at particular order– MACE– on treatment data.**

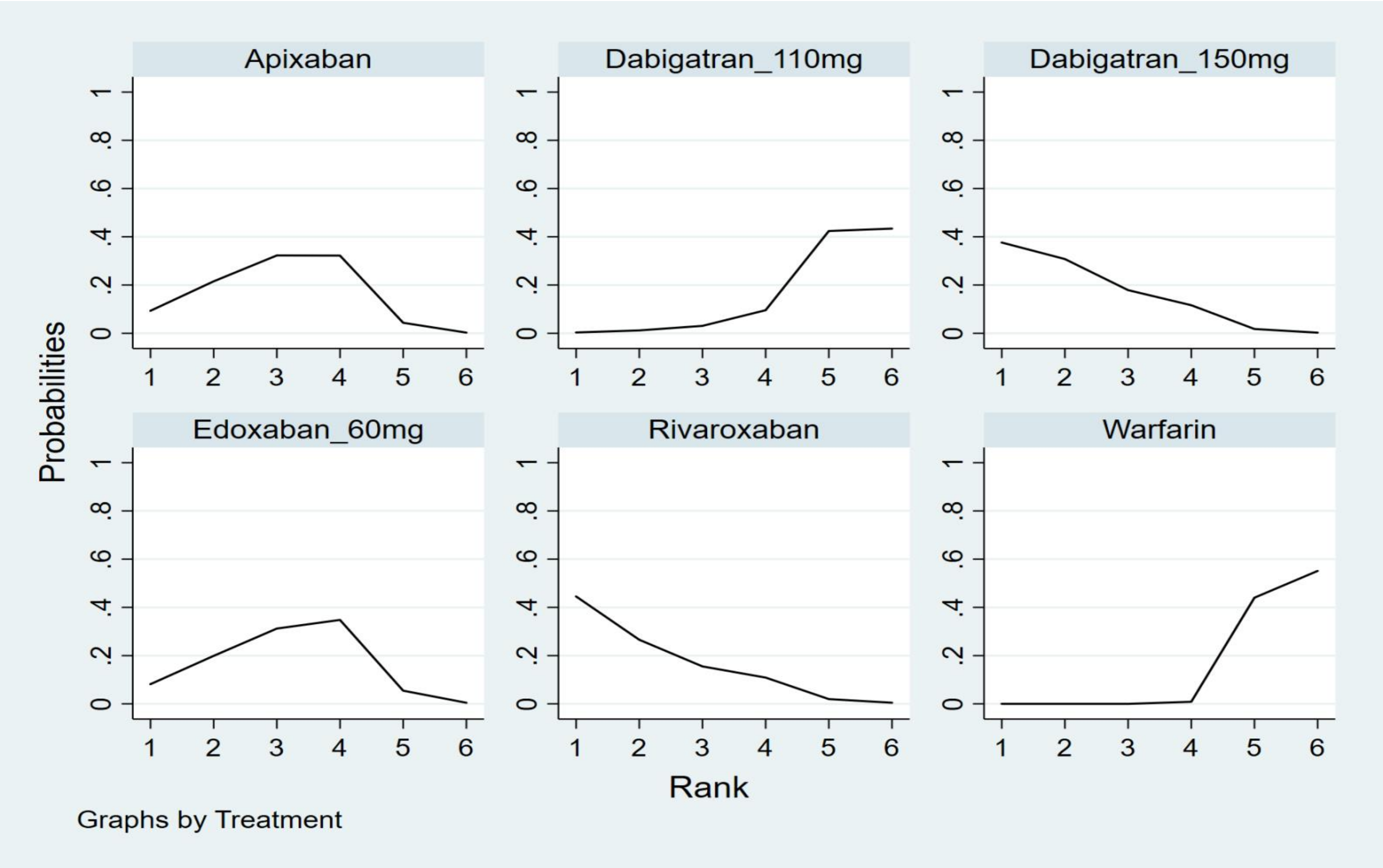

** - results from the reanalysis of RE-LY study [31]

Table S7. Direct and indirect comparison between warfarin and NOAC's – MI* data.

Dabigatran_110mg RR (95%CI)					
1,05 (0,72 - 1,55)	Dabigatran_150mg RR (95%CI)				
**1,6 (1,14 - 2,24)**	**1,52 (1,07 - 2,15)**	Apixaban RR (95%CI)			
**1,43 (1,02 - 2,01)**	1,36 (0,96 - 1,93)	0,89 (0,66 - 1,2)	Edoxaban_60mg RR (95%CI)		
**1,73 (1,2 - 2,49)**	**1,64 (1,14 - 2,37)**	1,08 (0,79 - 1,48)	0,89 (0,66 - 1,2)	Rivaroxaban RR (95%CI)	
**1,42 (1,09 - 1,84)**	**1,34 (1,02 - 1,77)**	0,88 (0,72 - 1,09)	0,99 (0,79 - 1,23)	0,82 (0,65 - 1,04)	Warfarin RR (95%CI)

Treatment 1	
RR (95%CI)	Treatment 2

* - results from the original RE-LY study [20]

Table S8. Treatment hierarchy assessed by surface under the cumulative ranking (SUCRA) curves. – MI* data.

Treatment	SUCRA
Warfarin	52,2
Apixaban	79,1
Dabigatran_110mg	8,2
Dabigatran_150mg	14,0
Edoxaban_60mg	56,4
Rivaroxaban	90,1

* - results from the original RE-LY study [20]

Figure S47. Rankograms for the drugs network showing the probability every treatment being at particular order– MI* data.

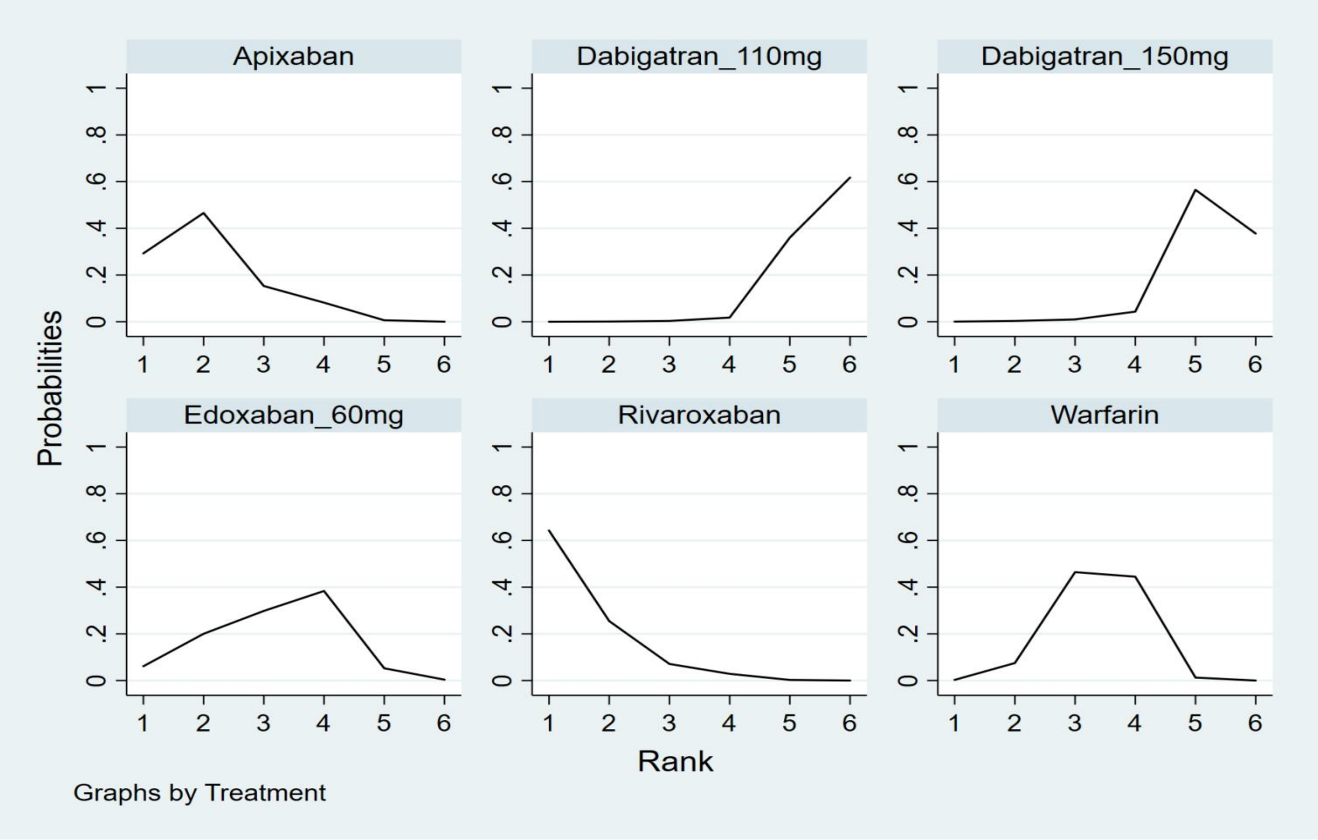

* - results from the original RE-LY study [20]

Table S9. Direct and indirect comparison between warfarin and NOAC's – MI- intention to treat data.**

Dabigatran_110mg RR (95%CI)				Treatment 1	
1,09 (0,75 - 1,56)	Dabigatran_150mg RR (95%CI)			RR (95%CI)	Treatment 2
**1,55 (1,12 - 2,14)**	**1,43 (1,02 - 2)**	Apixaban RR (95%CI)			
**1,38 (0,99 - 1,92)**	1,27 (0,91 - 1,79)	0,89 (0,66 - 1,2)	Edoxaban_60mg RR (95%CI)		
**1,67 (1,17 - 2,38)**	**1,54 (1,08 - 2,19)**	1,08 (0,79 - 1,48)	0,89 (0,66 - 1,2)	Rivaroxaban RR (95%CI)	
**1,37 (1,06 - 1,76)**	1,26 (0,97 - 1,64)	0,88 (0,72 - 1,09)	0,99 (0,79 - 1,23)	0,82 (0,65 - 1,04)	Warfarin RR (95%CI)

** - results from the reanalysis of RE-LY study [31]

Table S10. Treatment hierarchy assessed by surface under the cumulative ranking (SUCRA) curves. – MI - intention to treat data.**

Treatment	SUCRA
Warfarin	51,5
Apixaban	78,8
Dabigatran_110mg	7,4
Dabigatran_150mg	16,4
Edoxaban_60mg	55,7
Rivaroxaban	90,3

** - results from the reanalysis of RE-LY study [31]

Figure S48. Rankograms for the drugs network showing the probability every treatment being at particular order– MI - intention to treat data.**

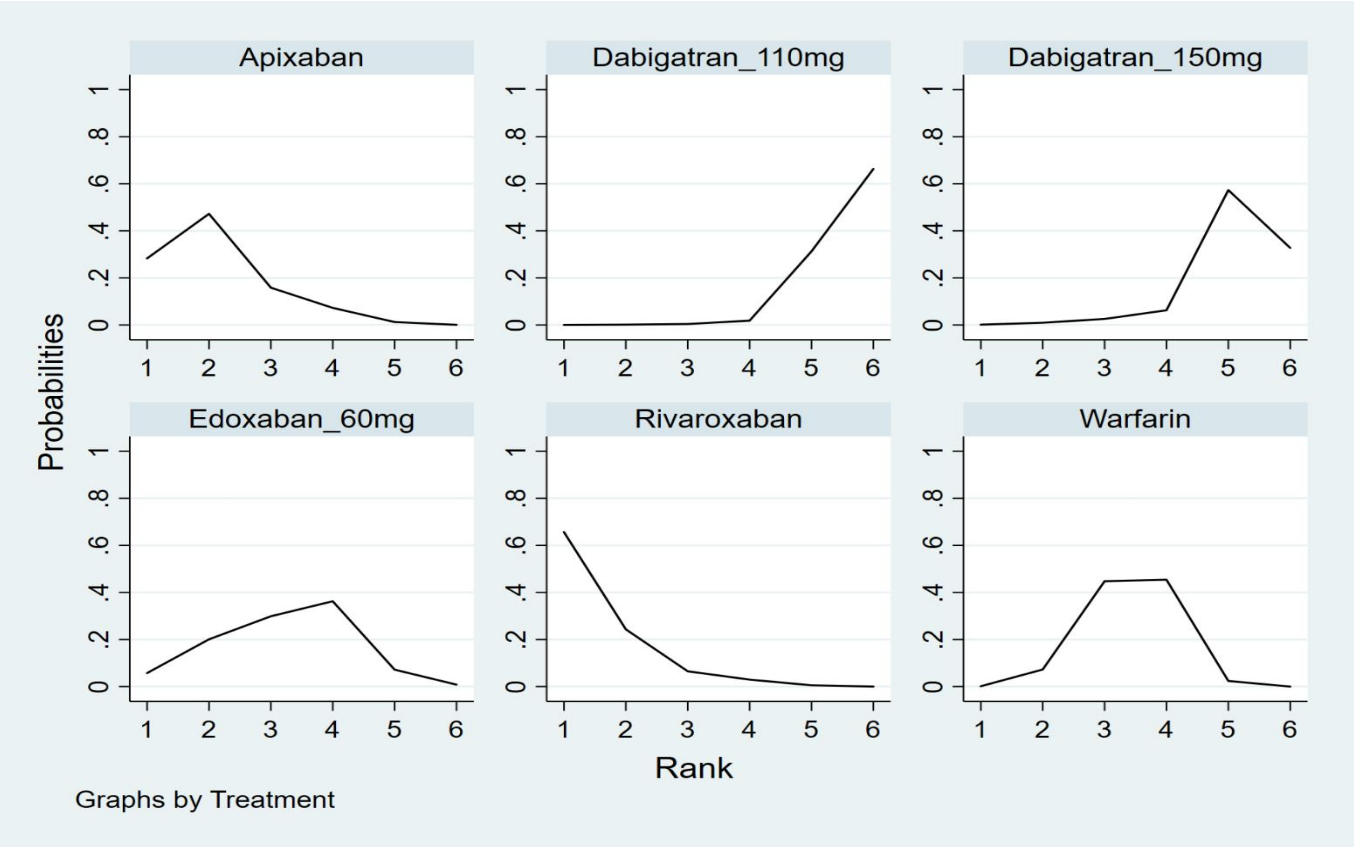

** - results from the reanalysis of RE-LY study [31]

Table S11. Direct and indirect comparison between warfarin and NOAC's – MI – on treatment data.**

Dabigatran_110mg RR (95%CI)					
1,1 (0,75 - 1,63)	Dabigatran_150mg RR (95%CI)				
**1,53 (1,09 - 2,15)**	1,39 (0,98 - 1,97)	Apixaban RR (95%CI)			
1,37 (0,97 - 1,92)	1,24 (0,87 - 1,77)	0,89 (0,66 - 1,2)	Edoxaban_60mg RR (95%CI)		
**1,65 (1,14 - 2,39)**	**1,5 (1,03 - 2,17)**	1,08 (0,79 - 1,48)	0,89 (0,66 - 1,2)	Rivaroxaban RR (95%CI)	
**1,35 (1,03 - 1,76)**	1,23 (0,92 - 1,63)	0,88 (0,72 - 1,09)	0,99 (0,79 - 1,23)	0,82 (0,65 - 1,04)	Warfarin RR (95%CI)

Treatment 1	
RR (95%CI)	Treatment 2

** - results from the reanalysis of RE-LY study [31]

Table S12. Treatment hierarchy assessed by surface under the cumulative ranking (SUCRA) curves. – MI – on treatment data.**

Treatment	SUCRA
Warfarin	50,9
Apixaban	78,5
Dabigatran_110mg	7,3
Dabigatran_150mg	18,5
Edoxaban_60mg	54,9
Rivaroxaban	90,0

** - results from the reanalysis of RE-LY study [31]

Figure S49. Rankograms for the drugs network showing the probability every treatment being at particular order– MI– on treatment data.**

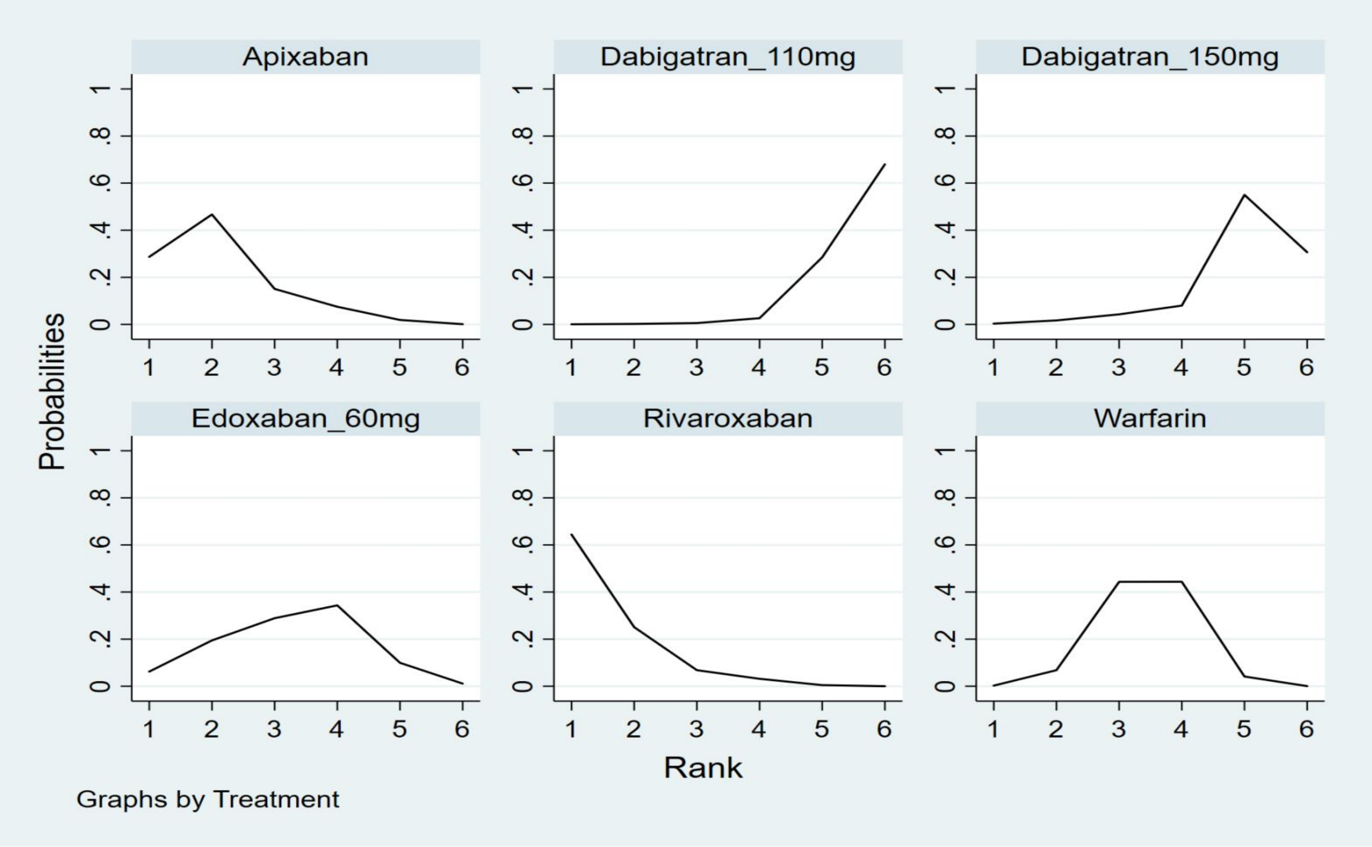

** - results from the reanalysis of RE-LY study [31]

Table S13. Direct and indirect comparison between warfarin and NOAC's – Stroke data.

Dabigatran_110mg RR (95%CI)					
1,36 (0,9 - 2,04)	Dabigatran_150mg RR (95%CI)				
1,36 (0,86 - 2,14)	1 (0,58 - 1,73)	Apixaban RR (95%CI)			
1,07 (0,84 - 1,38)	0,79 (0,54 - 1,17)	0,79 (0,51 - 1,22)	Edoxaban_60mg RR (95%CI)		
1,13 (0,75 - 1,69)	0,83 (0,55 - 1,24)	0,83 (0,53 - 1,3)	0,79 (0,51 - 1,22)	Rivaroxaban RR (95%CI)	
0,95 (0,78 - 1,16)	0,7 (0,49 - 1)	0,7 (0,46 - 1,05)	0,89 (0,76 - 1,03)	0,84 (0,7 - 1,02)	Warfarin RR (95%CI)

Treatment 1	
RR (95%CI)	Treatment 2

Table S14. Treatment hierarchy assessed by surface under the cumulative ranking (SUCRA) curves. – Stroke data.

Treatment	SUCRA
Warfarin	8,4
Apixaban	76,0
Dabigatran_110mg	24,5
Dabigatran_150mg	94,1
Edoxaban_60mg	43,1
Rivaroxaban	53,9

Figure S50. Rankograms for the drugs network showing the probability every treatment being at particular order - Stroke data.

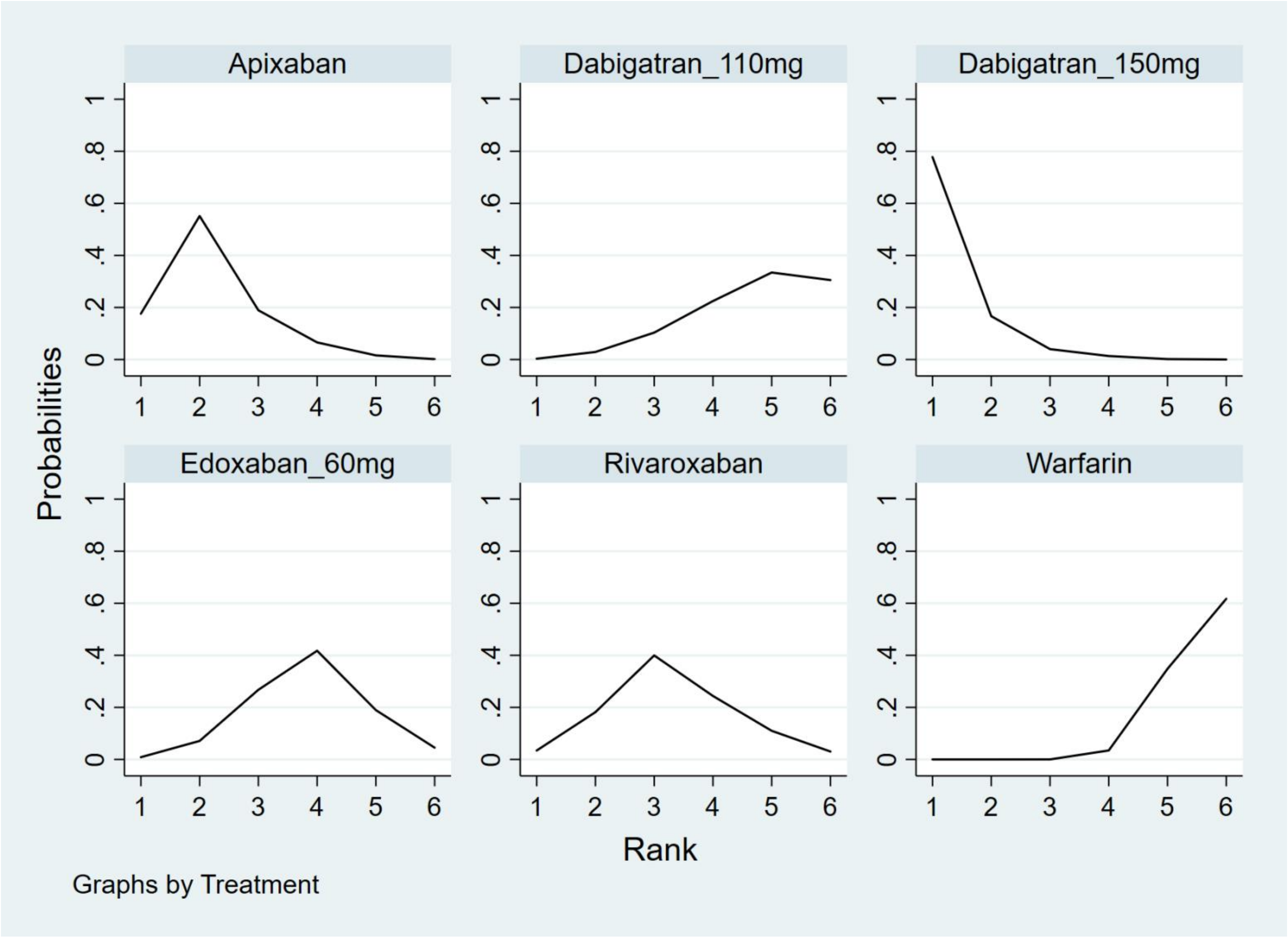

Table S15. Direct and indirect comparison between warfarin and NOAC's – overall mortality data.

Dabigatran_110mg RR (95%CI)					
1,07 (0,87 - 1,31)	Dabigatran_150mg RR (95%CI)				
1,06 (0,87 - 1,28)	0,99 (0,84 - 1,16)	Apixaban RR (95%CI)			
0,95 (0,7 - 1,3)	0,89 (0,67 - 1,19)	0,9 (0,68 - 1,2)	Edoxaban_60mg RR (95%CI)		
1,13 (0,92 - 1,4)	1,06 (0,86 - 1,31)	1,07 (0,88 - 1,31)	0,9 (0,68 - 1,2)	Rivaroxaban RR (95%CI)	
0,95 (0,81 - 1,12)	0,89 (0,79 - 1)	0,9 (0,81 - 1)	1 (0,77 - 1,3)	0,84 (0,71 - 1)	Warfin RR (95%CI)

Treatment 1	
RR (95%CI)	Treatment 2

Table S16. Treatment hierarchy assessed by surface under the cumulative ranking (SUCRA) curves. – overall mortality data.

Treatment	SUCRA
Warfarin	6,1
Apixaban	55,6
Dabigatran_110mg	42,9
Dabigatran_150mg	67,7
Edoxaban_60mg	44,4
Rivaroxaban	83,5

Figure S51. Rankograms for the drugs network showing the probability every treatment being at particular order – overall mortality data.

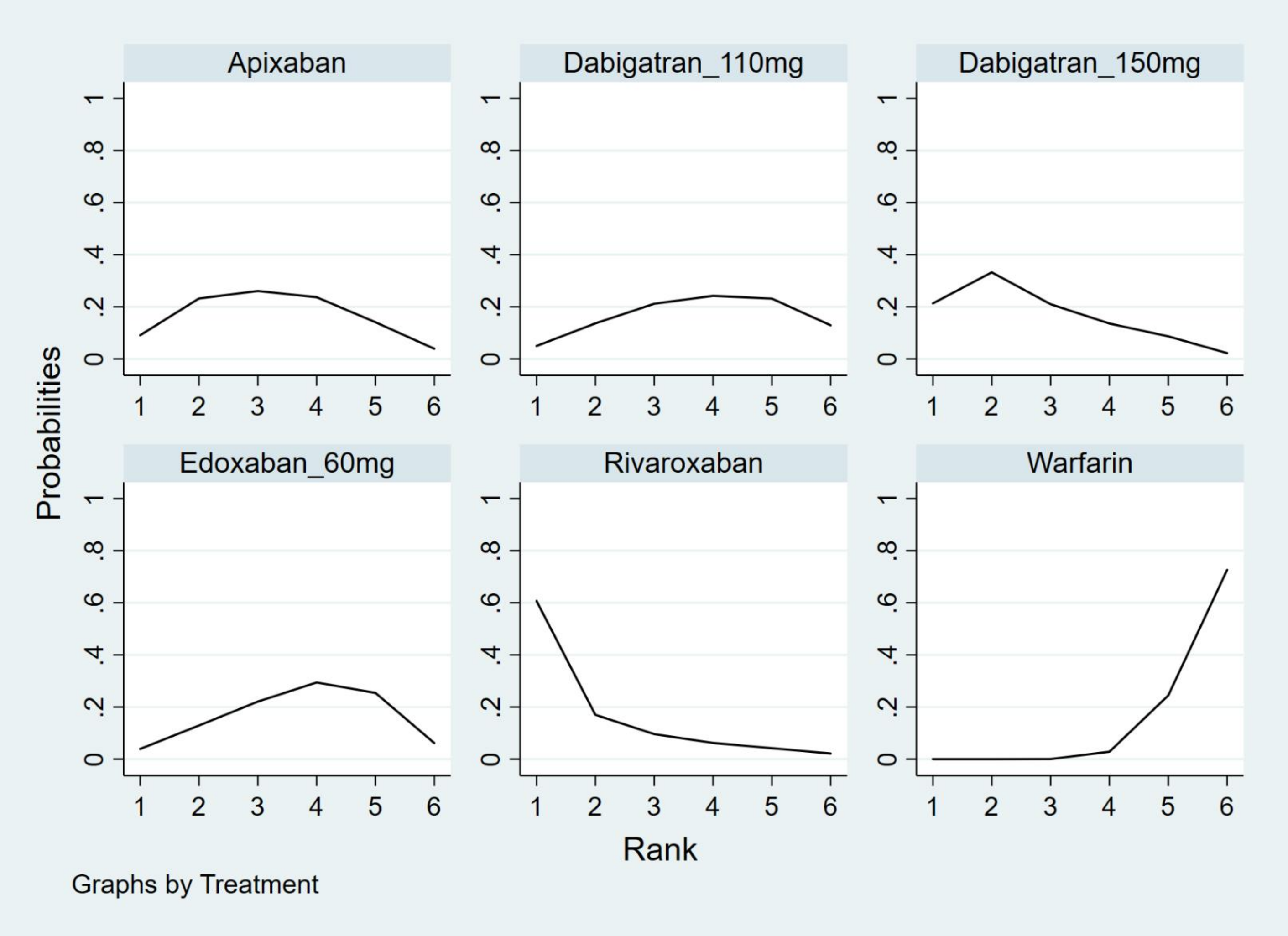

Supplement: Supplementary file 1 [file jpm-11-01013-s001.zip › jpm-1363450-supplementary.pdf]
